# Supplementary material for: Carvedilol suppresses ryanodine receptor-dependent Ca2+ bursts in human neurons bearing PSEN1 variants found in early onset Alzheimer’s disease
Source: PLoS One. 2024 Aug 22;19(8):e0291887. doi: 10.1371/journal.pone.0291887 (PMC11341060; doi:10.1371/journal.pone.0291887)
Supplement: S1 Fig — Immunocytochemical staining of WT and PSEN1 p.A246E neurons. Images obtained from WT neurons (A) and PSEN1 p.A246E neurons (B). The far-left panels are negative controls without using primary antibodies. Blue, DAPI; Red, β-tubulin Ⅲ; Green, MAP2; Scale bar, 10 μm. (DOCX) [file pone.0291887.s001.docx]

**
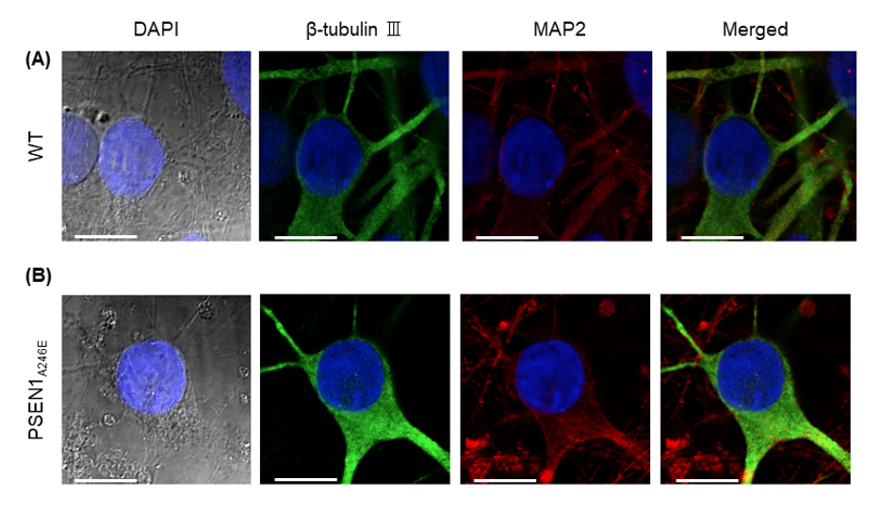
**

**S1 Fig**. **Immunocytochemical staining of WT and PSEN1 p.A246E neurons.** Images obtained from WT neurons (A) and PSEN1 p.A246E neurons (B). The far-left panels are negative controls without using primary antibodies. Blue, DAPI; Red, β-tubulin Ⅲ; Green, MAP2; Scale bar, 10 µm.
