# Supplementary figures and images for: Carvedilol suppresses ryanodine receptor-dependent Ca2+ bursts in human neurons bearing PSEN1 variants found in early onset Alzheimer’s disease
Source: PLoS One. 2024 Aug 22;19(8):e0291887. doi: 10.1371/journal.pone.0291887 (PMC11341060; doi:10.1371/journal.pone.0291887)

**
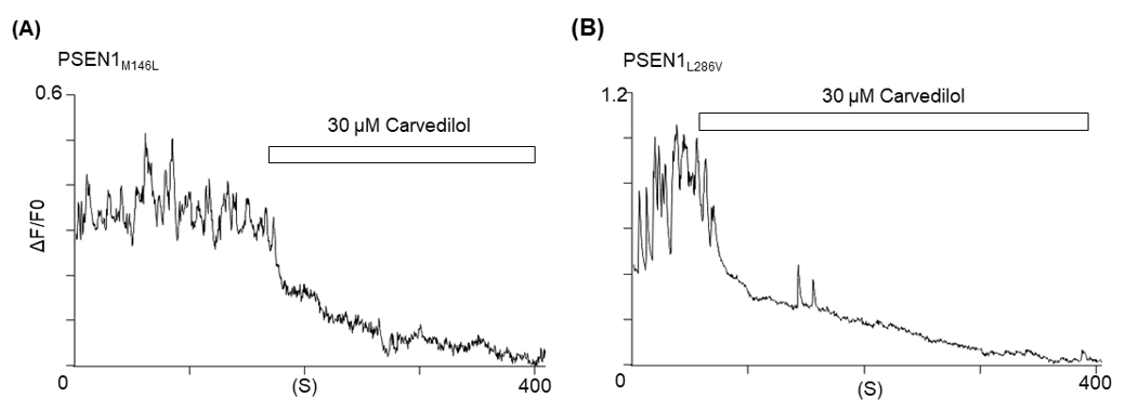
**

**S2 Fig. Effects of carvedilol on the Ca2+-bursts in the PSEN1M146L and PSEN1L286V neurons.**

Supplement: S2 Fig — (DOCX) [file pone.0291887.s002.docx]
