# Supplementary material for: Carvedilol suppresses ryanodine receptor-dependent Ca2+ bursts in human neurons bearing PSEN1 variants found in early onset Alzheimer’s disease
Source: PLoS One. 2024 Aug 22;19(8):e0291887. doi: 10.1371/journal.pone.0291887 (PMC11341060; doi:10.1371/journal.pone.0291887)
Supplement: S1 Table — NS: not significant, **p<0.01, CI: confidence interval, LL: lower limit, UL: upper limit. (DOCX) [file pone.0291887.s003.docx]

**S1 Table. Statistics for Ca^2+^-oscillation in Fig 2.**

| Ca oscillation | WT | A246E | M146L | L286V |
| --- | --- | --- | --- | --- |
| n | 592 | 353 | 155 | 363 |
| Ratio (%) | 9.1 | 34.6 | 52.9 | 59.5 |

| PSEN1  variants | PSEN1 variants | rates1 | rates 2 | difference | significance | 95%CI  LL | 95%CI  UL | 95%CI  LL | 95%CI  UL |
| --- | --- | --- | --- | --- | --- | --- | --- | --- | --- |
| WT | A246E | 0.091 | 0.346 | 9.717 | ** | -0.314 | -0.195 | -0.329 | -0.180 |
| WT | M146L | 0.091 | 0.529 | 11.452 | ** | -0.532 | -0.344 | -0.553 | -0.323 |
| WT | L286V | 0.091 | 0.595 | 16.149 | ** | -0.584 | -0.424 | -0.601 | -0.407 |
| A246E | M146L | 0.346 | 0.529 | 4.008 | ** | -0.290 | -0.076 | -0.318 | -0.049 |
| A246E | L286V | 0.346 | 0.595 | 6.842 | ** | -0.341 | -0.158 | -0.362 | -0.137 |
| M146L | L286V | 0.529 | 0.595 | 1.393 | NS | -0.173 | 0.041 | -0.201 | 0.069 |

NS: not significant, **p<0.01, CI: confidence interval, LL: lower limit, UL: upper limit
