# Supplementary material for: Carvedilol suppresses ryanodine receptor-dependent Ca2+ bursts in human neurons bearing PSEN1 variants found in early onset Alzheimer’s disease
Source: PLoS One. 2024 Aug 22;19(8):e0291887. doi: 10.1371/journal.pone.0291887 (PMC11341060; doi:10.1371/journal.pone.0291887)
Supplement: S2 Table — NS: not significant, **p<0.01, CI: confidence interval, LL: lower limit, UL: upper limit. (DOCX) [file pone.0291887.s004.docx]

**S2 Table. Statistics for Ca^2+^-bursts in Fig 2.**

| Ca burst | WT | A246E | M146L | L286V |
| --- | --- | --- | --- | --- |
| n | 592 | 353 | 155 | 363 |
| Ratio (%) | 2.0 | 43.1 | 14.2 | 6.1 |

| PSEN1  variants | PSEN1 variants | rates1 | rates 2 | difference | significance | 95%CI  LL | 95%CI  UL | 95%CI  LL | 95%CI  UL |
| --- | --- | --- | --- | --- | --- | --- | --- | --- | --- |
| WT | A246E | 0.020 | 0.431 | -0.410 | ** | -0.468 | -0.353 | -0.483 | -0.338 |
| WT | M146L | 0.020 | 0.142 | -0.122 | ** | -0.205 | -0.039 | -0.224 | -0.020 |
| WT | L286V | 0.020 | 0.061 | -0.040 | NS | -0.100 | 0.019 | -0.113 | 0.032 |
| A246E | M146L | 0.431 | 0.142 | 0.289 | ** | 0.185 | 0.392 | 0.159 | 0.419 |
| A246E | L286V | 0.431 | 0.061 | 0.370 | ** | 0.293 | 0.447 | 0.276 | 0.464 |
| M146L | L286V | 0.142 | 0.061 | 0.081 | ** | 0.021 | 0.142 | 0.005 | 0.157 |

NS: not significant, **p<0.01, CI: confidence interval, LL: lower limit, UL: upper limit
