# Supplementary material for: Carvedilol suppresses ryanodine receptor-dependent Ca2+ bursts in human neurons bearing PSEN1 variants found in early onset Alzheimer’s disease
Source: PLoS One. 2024 Aug 22;19(8):e0291887. doi: 10.1371/journal.pone.0291887 (PMC11341060; doi:10.1371/journal.pone.0291887)
Supplement: S3 Table — (DOCX) [file pone.0291887.s005.docx]

**S3 Table. A list of the top 10,000 genes that were affected in the PSEN1A246E neurons compared to the WT neuron.**

| **Gene** |  | **logFC** | **logCPM** | **PValue** | **FDR** |
| --- | --- | --- | --- | --- | --- |
| ZNF208 | 7757 | 10.037133 | 1.2424698 | 1.74E-05 | 2.57E-04 |
| BSX | 390259 | 8.9152557 | 0.1330032 | 1.04E-20 | 2.33E-18 |
| FAM177B | 400823 | 8.3229063 | -0.23866 | 1.66E-17 | 2.61E-15 |
| BMP4 | 652 | 7.9700751 | 1.6781897 | 3.08E-07 | 7.66E-06 |
| ZNF728 | 388523 | 7.6545153 | -0.962882 | 9.93E-13 | 7.34E-11 |
| ZNF578 | 147660 | 7.4850051 | 1.2894411 | 8.21E-31 | 4.72E-28 |
| HK2 | 3099 | 7.0944351 | 3.2546302 | 2.28E-04 | 2.21E-03 |
| OTX2 | 5015 | 6.8558279 | 4.1333135 | 1.24E-40 | 1.65E-37 |
| ADRB2 | 154 | 6.6036246 | 0.7356424 | 9.83E-08 | 2.80E-06 |
| TENT5C | 54855 | 6.5380505 | 0.7968204 | 9.41E-05 | 1.07E-03 |
| CXCL1 | 2919 | 6.5069077 | -0.308494 | 2.47E-05 | 3.43E-04 |
| INSM2 | 84684 | 6.4163182 | 2.5704149 | 7.19E-08 | 2.15E-06 |
| IRS4 | 8471 | 6.3045582 | 4.1045655 | 6.89E-08 | 2.07E-06 |
| ZNF667 | 63934 | 6.0866151 | 3.3260879 | 2.38E-40 | 2.87E-37 |
| CRYAB | 1410 | 5.6433504 | 3.6934468 | 8.95E-13 | 6.73E-11 |
| PCDHA5 | 56143 | 5.415891 | 2.0840626 | 4.67E-06 | 8.21E-05 |
| ACVR1C | 130399 | 5.4017175 | 0.5731498 | 5.31E-05 | 6.65E-04 |
| FOXF2 | 2295 | 5.3997531 | 1.2644367 | 3.83E-03 | 2.16E-02 |
| STON1-GTF2A1L | 286749 | 5.397593 | 1.2234483 | 1.73E-02 | 6.80E-02 |
| DEPDC1 | 55635 | 5.3763108 | -0.059198 | 5.37E-03 | 2.84E-02 |
| PGR | 5241 | 5.3217302 | -0.407129 | 9.51E-04 | 7.10E-03 |
| AKR1C4 | 1109 | 5.2678962 | 0.1670711 | 1.88E-08 | 6.45E-07 |
| CTSV | 1515 | 5.1939447 | 4.8551192 | 1.38E-06 | 2.85E-05 |
| RASSF9 | 9182 | 5.1911554 | 0.6515558 | 3.33E-02 | 1.10E-01 |
| PNMA5 | 114824 | 4.9686249 | 2.1687052 | 1.35E-13 | 1.18E-11 |
| IRF7 | 3665 | 4.9571975 | 0.9247552 | 5.57E-07 | 1.27E-05 |
| MT1E | 4493 | 4.8986191 | 1.8155159 | 1.31E-07 | 3.62E-06 |
| SLC18A1 | 6570 | 4.8770761 | 2.8055131 | 1.03E-06 | 2.23E-05 |
| ELF4 | 2000 | 4.8614497 | -0.624492 | 1.23E-03 | 8.73E-03 |
| SLFN12 | 55106 | 4.8075954 | 1.3555564 | 1.26E-02 | 5.36E-02 |
| HDC | 3067 | 4.6954141 | 1.7506414 | 1.59E-07 | 4.28E-06 |
| MAP3K19 | 80122 | 4.6813415 | -0.503605 | 8.61E-03 | 4.05E-02 |
| PROCR | 10544 | 4.6563902 | 1.7294945 | 7.31E-08 | 2.17E-06 |
| ZIC5 | 85416 | 4.6472811 | 4.2004499 | 5.04E-58 | 2.22E-54 |
| CEACAM21 | 90273 | 4.6083187 | 3.434021 | 7.78E-12 | 4.92E-10 |
| POU5F1B | 5462 | 4.6016806 | 0.1543935 | 9.20E-14 | 8.46E-12 |
| RPE65 | 6121 | 4.5854448 | 1.3477037 | 5.10E-08 | 1.57E-06 |
| MT1G | 4495 | 4.5314513 | 0.1855618 | 1.85E-02 | 7.14E-02 |
| HPD | 3242 | 4.5070176 | 3.8782312 | 2.48E-12 | 1.74E-10 |
| FGL2 | 10875 | 4.4970676 | 0.3168433 | 7.94E-06 | 1.31E-04 |
| CXCL12 | 6387 | 4.4904105 | -0.432872 | 1.54E-02 | 6.24E-02 |
| IL22 | 50616 | 4.3815328 | 2.5566724 | 3.82E-05 | 4.96E-04 |
| RBP7 | 116362 | 4.3579397 | 1.2141748 | 9.41E-04 | 7.03E-03 |
| CRYBA2 | 1412 | 4.289904 | -0.416138 | 2.20E-04 | 2.15E-03 |
| SLC9A3R1 | 9368 | 4.2694164 | 4.8159866 | 2.70E-11 | 1.53E-09 |
| MT2A | 4502 | 4.2552198 | 5.0425193 | 4.05E-11 | 2.25E-09 |
| ONECUT3 | 390874 | 4.2469882 | 2.9099503 | 4.49E-03 | 2.44E-02 |
| OTX1 | 5013 | 4.2251165 | 2.644343 | 5.49E-09 | 2.14E-07 |
| ATP8B3 | 148229 | 4.2005237 | -0.098272 | 9.87E-04 | 7.32E-03 |
| HSD17B2 | 3294 | 4.1904904 | 2.9734812 | 8.57E-07 | 1.89E-05 |
| C9orf64 | 84267 | 4.1767975 | 1.7687821 | 3.60E-03 | 2.06E-02 |
| HKDC1 | 80201 | 4.1610871 | 0.7244021 | 8.23E-02 | 2.09E-01 |
| MYH6 | 4624 | 4.1093127 | 0.4209623 | 3.47E-14 | 3.43E-12 |
| VWA5B1 | 127731 | 4.1073408 | 0.6562745 | 1.21E-12 | 8.92E-11 |
| SERPINF1 | 5176 | 4.0558981 | 3.8880079 | 5.89E-15 | 6.33E-13 |
| TNNI1 | 7135 | 4.049322 | 4.3130616 | 2.42E-30 | 1.23E-27 |
| SMPDL3B | 27293 | 4.0246481 | 0.40107 | 3.74E-16 | 4.59E-14 |
| TMEM191B | 728229 | 4.0242241 | 2.0470897 | 3.97E-24 | 1.38E-21 |
| HLA-DQA2 | 3118 | 4.0185562 | 3.2946144 | 7.85E-09 | 2.92E-07 |
| LRIG3 | 121227 | 3.9028636 | 0.8583965 | 1.17E-01 | 2.68E-01 |
| MYO1H | 283446 | 3.9002925 | 0.5881251 | 7.44E-10 | 3.40E-08 |
| CDKN2B | 1030 | 3.8842558 | 2.2384331 | 7.80E-05 | 9.19E-04 |
| MAMDC2 | 256691 | 3.8719041 | 4.6776818 | 2.43E-05 | 3.39E-04 |
| PLEKHD1 | 400224 | 3.8563787 | 0.0658788 | 4.24E-08 | 1.35E-06 |
| PLCD4 | 84812 | 3.8429742 | -0.307612 | 8.51E-05 | 9.85E-04 |
| WNT7B | 7477 | 3.7926762 | 2.1679541 | 3.14E-18 | 5.47E-16 |
| ANKFN1 | 162282 | 3.783355 | 0.7697469 | 8.88E-08 | 2.58E-06 |
| EMX2 | 2018 | 3.7108226 | 4.2564448 | 2.35E-05 | 3.31E-04 |
| DIO2 | 1734 | 3.7076137 | 1.1499875 | 3.54E-08 | 1.14E-06 |
| ONECUT1 | 3175 | 3.6467551 | 3.6786677 | 9.16E-14 | 8.46E-12 |
| SLC39A12 | 221074 | 3.6294851 | -0.049178 | 1.86E-01 | 3.65E-01 |
| TTC29 | 83894 | 3.6189736 | 1.7249365 | 5.84E-12 | 3.85E-10 |
| GCNT3 | 9245 | 3.6090594 | 1.74542 | 3.00E-16 | 3.78E-14 |
| VWA3A | 146177 | 3.6024616 | -0.468383 | 6.44E-05 | 7.84E-04 |
| ZIC4 | 84107 | 3.5906644 | 5.216254 | 9.74E-37 | 9.21E-34 |
| ULBP1 | 80329 | 3.573883 | 2.9939237 | 1.36E-09 | 5.94E-08 |
| ZPLD1 | 131368 | 3.5718521 | -0.089962 | 1.60E-02 | 6.40E-02 |
| CHRNB3 | 1142 | 3.5526837 | 0.2548754 | 3.35E-05 | 4.44E-04 |
| ERN2 | 10595 | 3.5430032 | 0.1464023 | 1.61E-05 | 2.40E-04 |
| S100A4 | 6275 | 3.513848 | 1.5176387 | 4.70E-11 | 2.59E-09 |
| APLN | 8862 | 3.4899156 | 3.320622 | 3.54E-03 | 2.02E-02 |
| RTKN | 6242 | 3.4729653 | 3.1999152 | 1.18E-14 | 1.23E-12 |
| BOLL | 66037 | 3.46018 | -0.928205 | 5.74E-08 | 1.74E-06 |
| FGF19 | 9965 | 3.4448215 | 5.3910026 | 9.59E-03 | 4.37E-02 |
| TMEM255A | 55026 | 3.4309612 | 5.1526575 | 1.63E-22 | 4.49E-20 |
| CDC14A | 8556 | 3.419207 | -0.129735 | 2.97E-03 | 1.75E-02 |
| TDO2 | 6999 | 3.4023002 | 2.7884676 | 1.37E-11 | 8.04E-10 |
| NECAB1 | 64168 | 3.382429 | 4.4389667 | 6.04E-36 | 5.00E-33 |
| PRSS35 | 167681 | 3.3114411 | 2.1523753 | 4.24E-04 | 3.70E-03 |
| ACOX2 | 8309 | 3.2978194 | 0.919268 | 8.07E-05 | 9.40E-04 |
| NPBWR1 | 2831 | 3.2869316 | 1.3499788 | 1.93E-03 | 1.25E-02 |
| FBP1 | 2203 | 3.2623469 | 0.6323407 | 4.61E-07 | 1.07E-05 |
| PODNL1 | 79883 | 3.2450216 | 1.4421066 | 4.33E-07 | 1.02E-05 |
| TXLNB | 167838 | 3.2449382 | 0.4080457 | 1.10E-11 | 6.71E-10 |
| TMEM244 | 253582 | 3.2330757 | -0.947775 | 2.60E-04 | 2.46E-03 |
| NDST4 | 64579 | 3.2179074 | 2.7876701 | 3.21E-19 | 5.98E-17 |
| MB | 4151 | 3.2047713 | -0.052756 | 1.60E-02 | 6.41E-02 |
| PLAAT3 | 11145 | 3.1962534 | 5.0601879 | 2.88E-33 | 2.01E-30 |
| FREM2 | 341640 | 3.1606396 | -0.20805 | 2.59E-02 | 9.16E-02 |
| BMP3 | 651 | 3.1554385 | 3.829794 | 7.32E-06 | 1.22E-04 |
| CPA4 | 51200 | 3.1462824 | 3.8009158 | 3.71E-02 | 1.19E-01 |
| KCTD4 | 386618 | 3.1239248 | 1.8560724 | 8.62E-11 | 4.60E-09 |
| PLCG2 | 5336 | 3.1221098 | 0.3369421 | 1.32E-08 | 4.68E-07 |
| ZNF474 | 133923 | 3.1017167 | 0.1482194 | 4.01E-05 | 5.16E-04 |
| MT1X | 4501 | 3.0949374 | 2.8273093 | 9.64E-20 | 1.99E-17 |
| CFAP47 | 286464 | 3.0825416 | 0.8391333 | 1.29E-04 | 1.39E-03 |
| SSH3 | 54961 | 3.0651774 | -0.020052 | 5.66E-03 | 2.96E-02 |
| TLX1 | 3195 | 3.0582492 | 0.522506 | 9.28E-03 | 4.27E-02 |
| S100A6 | 6277 | 3.0572299 | 5.6026842 | 3.29E-21 | 8.22E-19 |
| ASIC4 | 55515 | 3.0544626 | 1.4750803 | 1.72E-05 | 2.55E-04 |
| GNRH1 | 2796 | 3.0382343 | 6.3795786 | 7.38E-03 | 3.60E-02 |
| PPP1R17 | 10842 | 3.0223115 | 6.4362075 | 3.82E-05 | 4.96E-04 |
| AOX1 | 316 | 3.0216858 | 0.5806334 | 1.32E-04 | 1.41E-03 |
| NPFFR1 | 64106 | 3.0177939 | 0.0150042 | 1.81E-03 | 1.18E-02 |
| AMDHD1 | 144193 | 3.0108204 | -0.104083 | 1.52E-04 | 1.58E-03 |
| AHNAK | 79026 | 2.9961763 | 3.109749 | 5.98E-04 | 4.88E-03 |
| RTP1 | 132112 | 2.989688 | 0.9062431 | 3.32E-07 | 8.12E-06 |
| H1-4 | 3008 | 2.9885523 | 3.1051791 | 6.01E-09 | 2.29E-07 |
| TBR1 | 10716 | 2.9794357 | 4.8825357 | 2.09E-02 | 7.82E-02 |
| EVA1A | 84141 | 2.9716953 | 2.1356825 | 8.49E-03 | 4.01E-02 |
| SERPINB1 | 1992 | 2.9505218 | 2.2388618 | 1.90E-06 | 3.75E-05 |
| HEPHL1 | 341208 | 2.9489984 | 0.159609 | 5.28E-06 | 9.09E-05 |
| SYNE4 | 163183 | 2.9397828 | 3.2960893 | 2.61E-06 | 4.91E-05 |
| LHX9 | 56956 | 2.9294962 | 5.7129932 | 3.13E-03 | 1.83E-02 |
| CHRNA3 | 1136 | 2.9282976 | 2.6470916 | 4.73E-13 | 3.75E-11 |
| PLEKHB1 | 58473 | 2.9203456 | 5.4074955 | 1.56E-30 | 8.25E-28 |
| PCSK1 | 5122 | 2.9075609 | 7.3785499 | 4.06E-04 | 3.58E-03 |
| ZNF528 | 84436 | 2.8998706 | 2.5781511 | 6.73E-11 | 3.63E-09 |
| TFAP2E | 339488 | 2.8816496 | -0.315058 | 3.82E-02 | 1.21E-01 |
| ALDOB | 229 | 2.8726957 | 0.0328973 | 7.86E-05 | 9.23E-04 |
| PKP2 | 5318 | 2.8631379 | 1.7566909 | 9.10E-06 | 1.48E-04 |
| ORC1 | 4998 | 2.8523224 | 0.0089302 | 4.45E-04 | 3.85E-03 |
| ZIC1 | 7545 | 2.8454272 | 4.558188 | 1.37E-26 | 5.87E-24 |
| RORB | 6096 | 2.8439298 | 2.2161565 | 4.67E-08 | 1.48E-06 |
| AK4 | 205 | 2.8365697 | 4.3361937 | 2.41E-06 | 4.58E-05 |
| FEV | 54738 | 2.8228172 | -0.767042 | 1.88E-02 | 7.25E-02 |
| TNIP3 | 79931 | 2.7994744 | 2.2520985 | 2.17E-13 | 1.82E-11 |
| MAD1L1 | 8379 | 2.7993731 | 1.0602382 | 2.09E-10 | 1.05E-08 |
| SUCLG2 | 8801 | 2.7976815 | 2.7007812 | 4.92E-04 | 4.17E-03 |
| SOX5 | 6660 | 2.7933308 | 2.9088851 | 8.81E-12 | 5.50E-10 |
| CHEK1 | 1111 | 2.7819253 | 3.6051057 | 2.11E-25 | 8.72E-23 |
| H3C10 | 8357 | 2.767805 | 0.7742898 | 1.68E-03 | 1.12E-02 |
| TEKT1 | 83659 | 2.7544898 | -0.183487 | 1.70E-03 | 1.13E-02 |
| DGKG | 1608 | 2.7511892 | 2.12677 | 1.17E-17 | 1.89E-15 |
| CDCA2 | 157313 | 2.7336721 | 0.4189801 | 5.47E-04 | 4.53E-03 |
| LRRIQ3 | 127255 | 2.7305278 | -0.298603 | 3.11E-03 | 1.82E-02 |
| SPAG6 | 9576 | 2.7161401 | 1.8872107 | 1.57E-08 | 5.47E-07 |
| C22orf42 | 150297 | 2.7151831 | 6.9939823 | 3.92E-02 | 1.24E-01 |
| CREB3L1 | 90993 | 2.711871 | 2.3679857 | 7.92E-13 | 6.03E-11 |
| DGKK | 139189 | 2.7018918 | 2.0331129 | 2.76E-10 | 1.36E-08 |
| ANXA13 | 312 | 2.6997917 | -0.767833 | 5.10E-01 | 6.93E-01 |
| INPP5D | 3635 | 2.6992511 | -0.581181 | 1.07E-03 | 7.83E-03 |
| EPAS1 | 2034 | 2.6879684 | 2.5216575 | 1.00E-01 | 2.40E-01 |
| MAB21L2 | 10586 | 2.6872919 | -0.356348 | 1.72E-03 | 1.14E-02 |
| SALL1 | 6299 | 2.6856407 | 2.2556306 | 4.31E-06 | 7.65E-05 |
| PRDM16 | 63976 | 2.6690641 | 1.5569948 | 7.73E-10 | 3.51E-08 |
| GABRQ | 55879 | 2.6684622 | 2.507254 | 4.97E-08 | 1.54E-06 |
| SNCG | 6623 | 2.6658045 | 4.3264899 | 4.19E-04 | 3.67E-03 |
| VAX2 | 25806 | 2.6612091 | 0.4811926 | 2.73E-05 | 3.76E-04 |
| NMS | 129521 | 2.6590509 | 1.7378789 | 5.98E-03 | 3.09E-02 |
| GPR6 | 2830 | 2.6501238 | 2.7417179 | 2.00E-02 | 7.56E-02 |
| CENPI | 2491 | 2.6452478 | 0.4874695 | 2.60E-05 | 3.59E-04 |
| TROAP | 10024 | 2.6350412 | -0.092209 | 3.23E-02 | 1.08E-01 |
| NPR3 | 4883 | 2.6344975 | 0.7695141 | 5.00E-03 | 2.68E-02 |
| BTBD17 | 388419 | 2.6188506 | 3.5820927 | 1.15E-15 | 1.30E-13 |
| LAMA3 | 3909 | 2.6164069 | -0.07701 | 3.54E-04 | 3.18E-03 |
| TKTL1 | 8277 | 2.6127476 | 0.6415083 | 8.37E-03 | 3.96E-02 |
| UNC5B | 219699 | 2.6087477 | 1.2889769 | 1.94E-09 | 8.28E-08 |
| ZNF471 | 57573 | 2.6007507 | 1.825137 | 1.12E-08 | 4.06E-07 |
| FGF20 | 26281 | 2.5991688 | 1.1642421 | 5.49E-04 | 4.55E-03 |
| KIF14 | 9928 | 2.5958159 | -0.206419 | 2.81E-03 | 1.69E-02 |
| DDIT4L | 115265 | 2.594309 | 2.0902899 | 8.37E-03 | 3.96E-02 |
| EPB41L4A | 64097 | 2.5906835 | 2.7782647 | 2.56E-17 | 3.94E-15 |
| NEURL3 | 93082 | 2.5857303 | -0.114728 | 9.27E-03 | 4.27E-02 |
| OMG | 4974 | 2.5846387 | 1.8722073 | 2.85E-02 | 9.80E-02 |
| KRT8 | 3856 | 2.583451 | 1.3680293 | 3.71E-07 | 8.95E-06 |
| AKAP3 | 10566 | 2.579757 | 2.5142824 | 1.50E-13 | 1.29E-11 |
| LSR | 51599 | 2.5603301 | 0.9348125 | 1.03E-06 | 2.22E-05 |
| ZNF835 | 90485 | 2.5552464 | 0.7722786 | 8.11E-04 | 6.26E-03 |
| CA14 | 23632 | 2.5392096 | 3.4811978 | 5.75E-09 | 2.21E-07 |
| HLA-DRB5 | 3127 | 2.5389769 | 3.7873988 | 1.06E-04 | 1.18E-03 |
| STRA6 | 64220 | 2.5350115 | -0.393691 | 3.13E-05 | 4.20E-04 |
| ZXDA | 7789 | 2.5269979 | 1.103355 | 4.69E-04 | 4.01E-03 |
| ARMC3 | 219681 | 2.526427 | 1.9936366 | 2.36E-06 | 4.52E-05 |
| CALB2 | 794 | 2.5230118 | 7.304608 | 1.28E-06 | 2.69E-05 |
| SH3BGRL2 | 83699 | 2.5223409 | 5.1369848 | 1.27E-16 | 1.71E-14 |
| MKRN2OS | 100129480 | 2.5205342 | 1.6964824 | 9.81E-04 | 7.29E-03 |
| ILDR2 | 387597 | 2.5176643 | 2.0886271 | 4.18E-06 | 7.44E-05 |
| STON2 | 85439 | 2.511288 | 3.3600338 | 3.37E-16 | 4.20E-14 |
| FYCO1 | 79443 | 2.5095273 | 0.4862543 | 7.34E-03 | 3.60E-02 |
| C1orf53 | 388722 | 2.5078025 | 3.6206739 | 1.24E-19 | 2.41E-17 |
| PRTN3 | 5657 | 2.5041413 | -0.097779 | 2.85E-02 | 9.80E-02 |
| NHLH2 | 4808 | 2.5002867 | 4.7911903 | 3.34E-06 | 6.11E-05 |
| SGPP2 | 130367 | 2.4931356 | 1.4904628 | 3.49E-05 | 4.59E-04 |
| RD3L | 647286 | 2.485644 | 1.866406 | 1.90E-03 | 1.24E-02 |
| FOXR1 | 283150 | 2.4847595 | -0.7003 | 1.04E-05 | 1.66E-04 |
| ZMYND12 | 84217 | 2.4792536 | 1.9572554 | 1.55E-07 | 4.18E-06 |
| HMMR | 3161 | 2.4771319 | 1.0247583 | 4.35E-03 | 2.38E-02 |
| RHOU | 58480 | 2.4757445 | 3.054841 | 5.35E-12 | 3.54E-10 |
| APOLD1 | 81575 | 2.4743387 | 2.4236287 | 1.81E-03 | 1.19E-02 |
| CFAP57 | 149465 | 2.4715665 | 0.5332405 | 1.98E-06 | 3.90E-05 |
| EDA2R | 60401 | 2.4707985 | 2.9505496 | 5.21E-04 | 4.36E-03 |
| KLF10 | 7071 | 2.4457194 | 3.3368825 | 6.74E-09 | 2.53E-07 |
| PARP9 | 83666 | 2.445244 | 1.7186433 | 8.81E-02 | 2.18E-01 |
| DDX60 | 55601 | 2.4450015 | -0.221194 | 9.00E-02 | 2.22E-01 |
| ESAM | 90952 | 2.4359536 | 0.3589497 | 4.00E-02 | 1.26E-01 |
| OCLN | 100506658 | 2.4345476 | 2.5354829 | 3.41E-05 | 4.50E-04 |
| SAMD3 | 154075 | 2.4328124 | 4.7134215 | 3.77E-02 | 1.21E-01 |
| LDLRAD3 | 143458 | 2.4322001 | 5.0830513 | 1.49E-06 | 3.04E-05 |
| CRYBG3 | 131544 | 2.4255222 | 1.1780739 | 1.26E-04 | 1.36E-03 |
| CXXC5 | 51523 | 2.4215975 | 7.1111587 | 5.84E-15 | 6.33E-13 |
| KDR | 3791 | 2.4211585 | 0.2123217 | 3.04E-02 | 1.03E-01 |
| LHX2 | 9355 | 2.4180224 | 3.9245449 | 3.25E-07 | 8.04E-06 |
| FGF3 | 2248 | 2.4095081 | 1.9679049 | 2.27E-02 | 8.31E-02 |
| ZFPM2 | 23414 | 2.405201 | 2.226044 | 2.74E-11 | 1.55E-09 |
| EPS8L1 | 54869 | 2.4008103 | 4.3840534 | 6.17E-05 | 7.58E-04 |
| CGA | 1081 | 2.3999052 | 2.208512 | 8.25E-02 | 2.09E-01 |
| DHRS2 | 10202 | 2.3982827 | 2.1717339 | 5.92E-13 | 4.58E-11 |
| RBM47 | 54502 | 2.3720472 | 2.076747 | 3.05E-02 | 1.03E-01 |
| CLDN7 | 1366 | 2.3720458 | 0.0208512 | 2.41E-05 | 3.37E-04 |
| DRD2 | 1813 | 2.3712947 | 3.3094898 | 1.51E-05 | 2.27E-04 |
| PMEL | 6490 | 2.3625687 | 2.7697572 | 1.84E-03 | 1.20E-02 |
| ODF3B | 440836 | 2.3588466 | 0.6990726 | 7.51E-05 | 8.91E-04 |
| SP100 | 6672 | 2.3561494 | 0.887551 | 2.10E-01 | 3.96E-01 |
| LOXL1 | 4016 | 2.3534248 | 3.4741145 | 3.13E-04 | 2.88E-03 |
| TBX19 | 9095 | 2.3527372 | 0.1898539 | 4.01E-02 | 1.26E-01 |
| KLHL41 | 10324 | 2.3438275 | 1.8062856 | 2.47E-07 | 6.24E-06 |
| FRMD7 | 90167 | 2.342716 | -0.316669 | 4.06E-02 | 1.27E-01 |
| EFCAB12 | 90288 | 2.3374812 | 2.3791436 | 3.68E-03 | 2.09E-02 |
| LDLRAP1 | 26119 | 2.3369118 | 2.0492941 | 5.37E-02 | 1.55E-01 |
| ARL17B | 100996709 | 2.3321065 | 2.0926664 | 5.17E-13 | 4.05E-11 |
| CCND1 | 595 | 2.3301603 | 5.175133 | 1.46E-04 | 1.53E-03 |
| TRH | 7200 | 2.3282687 | 4.6053453 | 7.57E-04 | 5.93E-03 |
| RADX | 55086 | 2.323682 | 5.8605766 | 6.06E-13 | 4.66E-11 |
| EXO1 | 9156 | 2.3195306 | 0.2742277 | 2.09E-03 | 1.33E-02 |
| ZNF600 | 162966 | 2.3151275 | 1.2481688 | 1.30E-03 | 9.13E-03 |
| CD38 | 952 | 2.306276 | 2.2607861 | 1.74E-02 | 6.82E-02 |
| SKA1 | 220134 | 2.3016777 | 0.4985946 | 1.22E-02 | 5.24E-02 |
| DDC | 1644 | 2.3000811 | 3.9416339 | 2.95E-02 | 1.01E-01 |
| LMX1A | 4009 | 2.2835302 | -0.50256 | 1.94E-02 | 7.41E-02 |
| PTPN3 | 5774 | 2.2786993 | 1.3357519 | 3.82E-05 | 4.96E-04 |
| ST18 | 9705 | 2.2632318 | 2.3910422 | 2.42E-03 | 1.49E-02 |
| AMPD3 | 272 | 2.2616145 | 0.8058626 | 2.95E-02 | 1.01E-01 |
| AEN | 64782 | 2.2602809 | 5.1276144 | 4.71E-06 | 8.27E-05 |
| C6 | 729 | 2.2542569 | 0.2961074 | 3.53E-05 | 4.62E-04 |
| ABCD2 | 225 | 2.2538524 | 1.0664912 | 4.89E-08 | 1.53E-06 |
| PIPOX | 51268 | 2.2535889 | 4.3338773 | 3.26E-12 | 2.27E-10 |
| PHLDB2 | 90102 | 2.2423665 | 2.3977054 | 2.20E-02 | 8.14E-02 |
| MCM10 | 55388 | 2.2411878 | 0.8604852 | 5.84E-03 | 3.04E-02 |
| ZIC2 | 7546 | 2.2356353 | 3.8922103 | 9.87E-06 | 1.59E-04 |
| MAP1LC3C | 440738 | 2.2346941 | 1.7953575 | 6.96E-05 | 8.36E-04 |
| ATF3 | 467 | 2.2339139 | 3.5944256 | 2.38E-03 | 1.47E-02 |
| RET | 5979 | 2.230333 | 2.6128596 | 4.31E-05 | 5.51E-04 |
| METRN | 79006 | 2.2274682 | 4.9698904 | 2.33E-05 | 3.29E-04 |
| C21orf62 | 56245 | 2.2271185 | 5.5391398 | 2.37E-19 | 4.48E-17 |
| ARHGAP9 | 64333 | 2.2261764 | 1.0371871 | 2.80E-07 | 7.04E-06 |
| USH2A | 7399 | 2.22278 | -0.578688 | 5.96E-03 | 3.09E-02 |
| CCDC85A | 114800 | 2.2142438 | 2.8500785 | 8.69E-03 | 4.07E-02 |
| NLRP2 | 55655 | 2.2138442 | 2.9335275 | 6.43E-09 | 2.43E-07 |
| IL34 | 146433 | 2.2127396 | 0.0277925 | 7.38E-03 | 3.60E-02 |
| ZNF730 | 100129543 | 2.2114596 | 1.9921757 | 2.53E-05 | 3.51E-04 |
| STYK1 | 55359 | 2.2080506 | 2.1320517 | 9.68E-03 | 4.40E-02 |
| EFCAB11 | 90141 | 2.2031254 | 2.3152201 | 4.21E-08 | 1.34E-06 |
| PKD1L2 | 114780 | 2.201188 | 0.8195444 | 1.08E-01 | 2.53E-01 |
| FAM81B | 153643 | 2.1882306 | 1.1516696 | 2.52E-02 | 9.00E-02 |
| RNF135 | 84282 | 2.1773931 | 0.0911337 | 4.54E-04 | 3.91E-03 |
| PRTG | 283659 | 2.1712512 | 1.7564473 | 5.13E-03 | 2.74E-02 |
| H2AC6 | 8334 | 2.1706067 | 5.2297983 | 1.46E-08 | 5.15E-07 |
| TMOD1 | 7111 | 2.1693376 | 1.1566711 | 1.03E-04 | 1.15E-03 |
| SHISA6 | 388336 | 2.1614782 | 3.2681218 | 1.20E-02 | 5.18E-02 |
| KCNJ12 | 3768 | 2.1573747 | 2.5939922 | 3.39E-04 | 3.07E-03 |
| ZNF630 | 57232 | 2.1507388 | 3.3022959 | 2.30E-06 | 4.43E-05 |
| RTL9 | 57529 | 2.1492971 | 1.8574835 | 5.46E-03 | 2.88E-02 |
| CHGB | 1114 | 2.1479356 | 8.8090075 | 2.36E-02 | 8.56E-02 |
| FRZB | 2487 | 2.1463404 | 4.7441975 | 9.79E-06 | 1.58E-04 |
| PIK3AP1 | 118788 | 2.1459497 | 0.1151367 | 1.41E-04 | 1.49E-03 |
| CA10 | 56934 | 2.124616 | 5.1460472 | 6.59E-03 | 3.31E-02 |
| CALHM5 | 254228 | 2.1245244 | 1.1559075 | 1.51E-05 | 2.27E-04 |
| CTXN3 | 613212 | 2.122458 | 2.0711141 | 1.47E-02 | 6.02E-02 |
| CFAP58 | 159686 | 2.1216177 | -0.177723 | 9.23E-02 | 2.25E-01 |
| ART3 | 419 | 2.1202777 | 1.8588448 | 3.75E-02 | 1.20E-01 |
| MDGA1 | 266727 | 2.1183634 | 3.6682319 | 3.44E-06 | 6.25E-05 |
| KLK10 | 5655 | 2.1153161 | 2.8786073 | 8.71E-02 | 2.16E-01 |
| CBLN4 | 140689 | 2.1132706 | 1.4660384 | 2.78E-05 | 3.81E-04 |
| TSTD1 | 100131187 | 2.110108 | 2.6776158 | 8.43E-12 | 5.28E-10 |
| CAPSL | 133690 | 2.1100223 | -0.050259 | 9.39E-02 | 2.28E-01 |
| SAMD11 | 148398 | 2.1063994 | 1.3599316 | 2.13E-05 | 3.05E-04 |
| RBMS1 | 5937 | 2.0955229 | 4.926409 | 1.12E-06 | 2.40E-05 |
| SUSD5 | 26032 | 2.0940615 | 1.0638148 | 4.32E-03 | 2.37E-02 |
| SYT2 | 127833 | 2.0813435 | -0.201601 | 1.72E-03 | 1.14E-02 |
| GDPD2 | 54857 | 2.0796021 | 3.6726841 | 6.18E-09 | 2.34E-07 |
| CHRNA2 | 1135 | 2.0793019 | -0.091727 | 2.85E-02 | 9.79E-02 |
| PDE11A | 50940 | 2.0712747 | -0.035601 | 8.30E-03 | 3.93E-02 |
| H2AC19 | 723790 | 2.0678653 | -0.287709 | 1.05E-02 | 4.69E-02 |
| MYBPC1 | 4604 | 2.0642308 | -0.258194 | 3.75E-03 | 2.12E-02 |
| ARHGEF16 | 27237 | 2.049366 | 0.4572398 | 1.31E-04 | 1.40E-03 |
| CPXM2 | 119587 | 2.0442729 | 1.0907758 | 8.22E-06 | 1.36E-04 |
| RNF182 | 221687 | 2.0420629 | 4.5422568 | 3.21E-05 | 4.31E-04 |
| TAL2 | 6887 | 2.036972 | -0.26068 | 1.58E-01 | 3.26E-01 |
| RRAD | 6236 | 2.0368051 | 1.492468 | 3.37E-06 | 6.16E-05 |
| PELI2 | 57161 | 2.0280724 | 2.3534034 | 5.36E-04 | 4.46E-03 |
| MAOA | 4128 | 2.024249 | 5.2586938 | 9.15E-16 | 1.05E-13 |
| RHOH | 399 | 2.0242247 | 0.2725199 | 4.26E-02 | 1.31E-01 |
| CNTN6 | 27255 | 2.0206303 | 1.5283145 | 1.77E-05 | 2.61E-04 |
| MYO15A | 51168 | 2.0169152 | 0.2221628 | 3.45E-06 | 6.27E-05 |
| CECR2 | 27443 | 2.0148908 | 0.7235616 | 2.81E-04 | 2.62E-03 |
| ESRP1 | 54845 | 2.013279 | 1.0406834 | 8.94E-06 | 1.45E-04 |
| PCDHAC1 | 56135 | 2.0124523 | 0.278089 | 3.94E-03 | 2.21E-02 |
| TPPP3 | 51673 | 2.0106426 | 4.3549644 | 1.14E-22 | 3.28E-20 |
| IL15 | 3600 | 2.0074435 | 0.754831 | 7.11E-03 | 3.50E-02 |
| MDK | 4192 | 2.0067755 | 7.5375194 | 2.63E-16 | 3.35E-14 |
| IFI44L | 10964 | 1.9895248 | -0.030666 | 1.02E-01 | 2.42E-01 |
| CCNB3 | 85417 | 1.9873407 | 1.3399537 | 7.84E-02 | 2.02E-01 |
| VANGL1 | 81839 | 1.9863146 | 3.5002904 | 8.78E-11 | 4.66E-09 |
| SEMA3B | 7869 | 1.9836632 | 0.7558613 | 2.93E-04 | 2.72E-03 |
| TMEM154 | 201799 | 1.9819573 | 0.3908698 | 7.45E-02 | 1.95E-01 |
| MYH7 | 4625 | 1.9798711 | 3.329007 | 1.87E-07 | 4.90E-06 |
| SLC44A3 | 126969 | 1.9794775 | 2.1232889 | 2.14E-06 | 4.17E-05 |
| VAX1 | 11023 | 1.9745103 | 1.7903123 | 1.79E-05 | 2.63E-04 |
| TLL2 | 7093 | 1.9734917 | 2.1621815 | 1.46E-03 | 9.97E-03 |
| C20orf204 | 284739 | 1.9699784 | 0.2882035 | 4.32E-02 | 1.33E-01 |
| MAL2 | 114569 | 1.9680876 | 4.5340534 | 1.48E-03 | 1.01E-02 |
| RIT2 | 6014 | 1.9671431 | 4.5505023 | 1.97E-02 | 7.48E-02 |
| ANXA5 | 308 | 1.964223 | 7.6646825 | 6.20E-04 | 5.01E-03 |
| SYT17 | 51760 | 1.9614387 | 3.5179875 | 1.84E-14 | 1.88E-12 |
| SULF1 | 23213 | 1.9528931 | 1.0105179 | 1.92E-03 | 1.24E-02 |
| MFAP4 | 4239 | 1.950572 | 0.9612054 | 1.01E-01 | 2.41E-01 |
| NEBL | 10529 | 1.9491841 | 5.0650663 | 3.89E-09 | 1.55E-07 |
| STING1 | 340061 | 1.9462255 | 3.5082906 | 4.27E-04 | 3.73E-03 |
| PRR11 | 55771 | 1.9403352 | 1.9693671 | 4.99E-04 | 4.22E-03 |
| PDLIM2 | 64236 | 1.9389307 | 4.6279749 | 8.55E-08 | 2.50E-06 |
| H1-3 | 3007 | 1.9377372 | 1.0685323 | 1.34E-02 | 5.61E-02 |
| C8G | 733 | 1.9346205 | -0.048944 | 1.10E-02 | 4.86E-02 |
| THAP5 | 168451 | 1.9260506 | 4.3439937 | 1.84E-09 | 7.95E-08 |
| NAT1 | 9 | 1.9244198 | 0.571865 | 2.12E-02 | 7.88E-02 |
| COL5A1 | 1289 | 1.9154334 | 1.2817842 | 3.41E-02 | 1.12E-01 |
| LRRC75B | 388886 | 1.9136532 | 5.5610959 | 3.87E-17 | 5.81E-15 |
| ZNF680 | 340252 | 1.9081255 | 2.4668794 | 2.74E-07 | 6.90E-06 |
| SLC2A14 | 144195 | 1.9062962 | 1.6642897 | 4.33E-03 | 2.38E-02 |
| LRRC37A2 | 474170 | 1.9026661 | 1.0329246 | 7.22E-04 | 5.71E-03 |
| BFSP1 | 631 | 1.8989549 | 1.6841638 | 3.42E-06 | 6.24E-05 |
| EML5 | 161436 | 1.8881256 | 1.8215456 | 5.18E-04 | 4.34E-03 |
| ANXA1 | 301 | 1.886789 | 6.52743 | 4.08E-03 | 2.27E-02 |
| CFAP61 | 26074 | 1.8850762 | 1.4466097 | 2.12E-05 | 3.05E-04 |
| PADI2 | 11240 | 1.8771553 | 0.3552106 | 1.32E-03 | 9.20E-03 |
| PLK4 | 10733 | 1.8760263 | 0.9192023 | 1.23E-05 | 1.92E-04 |
| ZEB1 | 6935 | 1.8741162 | 6.293086 | 5.48E-16 | 6.42E-14 |
| BHLHE40 | 8553 | 1.8740347 | 3.650144 | 2.47E-02 | 8.85E-02 |
| NEK7 | 140609 | 1.8679153 | 3.4155663 | 9.90E-05 | 1.12E-03 |
| ZNF114 | 163071 | 1.8610286 | 3.4533669 | 2.93E-03 | 1.74E-02 |
| SLC35F2 | 54733 | 1.8602607 | 1.6235072 | 2.12E-05 | 3.05E-04 |
| KRT17 | 3872 | 1.8522336 | 0.027777 | 2.38E-02 | 8.61E-02 |
| CA9 | 768 | 1.8509091 | -0.035094 | 1.54E-02 | 6.24E-02 |
| H2BC6 | 8344 | 1.8493976 | 0.429917 | 1.41E-04 | 1.48E-03 |
| RGS16 | 6004 | 1.8469063 | 4.3191294 | 3.19E-04 | 2.92E-03 |
| HS3ST2 | 9956 | 1.8448758 | 4.1649094 | 8.19E-07 | 1.81E-05 |
| MYL9 | 10398 | 1.844541 | 1.0331112 | 1.75E-02 | 6.85E-02 |
| LRRC55 | 219527 | 1.84312 | 1.5029199 | 6.29E-02 | 1.73E-01 |
| CPEB1 | 64506 | 1.8427656 | 2.6968551 | 1.92E-08 | 6.52E-07 |
| FAM47E | 100129583 | 1.8423284 | 1.3790838 | 2.92E-05 | 3.98E-04 |
| PLP1 | 5354 | 1.8395322 | 4.257479 | 2.02E-09 | 8.54E-08 |
| ULBP3 | 79465 | 1.8388184 | 3.2681568 | 5.64E-09 | 2.18E-07 |
| RGS5 | 8490 | 1.8364224 | 4.2767229 | 5.31E-02 | 1.53E-01 |
| RTN4RL2 | 349667 | 1.8354375 | 0.6914324 | 3.22E-03 | 1.88E-02 |
| ANO2 | 57101 | 1.834327 | -0.345225 | 6.31E-02 | 1.73E-01 |
| TDRD10 | 126668 | 1.8318604 | -0.335523 | 9.40E-02 | 2.28E-01 |
| CRACR2B | 283229 | 1.82761 | 2.7419091 | 3.72E-06 | 6.72E-05 |
| ZNF85 | 7639 | 1.8270222 | 2.7650393 | 2.99E-05 | 4.05E-04 |
| UCP2 | 7351 | 1.8163649 | 4.1717459 | 1.02E-07 | 2.90E-06 |
| SYT9 | 143425 | 1.8149339 | 2.5541368 | 1.06E-09 | 4.73E-08 |
| JADE3 | 9767 | 1.8135196 | 2.1099936 | 3.72E-03 | 2.11E-02 |
| CBS | 875 | 1.8092278 | 1.6903485 | 2.65E-04 | 2.49E-03 |
| KIF20A | 10112 | 1.802826 | 0.3526641 | 1.59E-02 | 6.38E-02 |
| POU2F2 | 5452 | 1.8001361 | 4.4171083 | 2.09E-02 | 7.83E-02 |
| CAVIN1 | 284119 | 1.799762 | 5.6018744 | 6.96E-02 | 1.86E-01 |
| CCDC121 | 79635 | 1.7984933 | 2.0764971 | 3.73E-07 | 9.00E-06 |
| CDC25A | 993 | 1.7924064 | 1.6880074 | 9.46E-08 | 2.71E-06 |
| GFRA3 | 2676 | 1.792042 | 0.6517182 | 1.42E-04 | 1.49E-03 |
| GNG8 | 94235 | 1.7920014 | 5.7544489 | 1.70E-01 | 3.44E-01 |
| MSMB | 4477 | 1.7877124 | 0.1133947 | 1.55E-01 | 3.21E-01 |
| CD6 | 923 | 1.7822468 | 0.4154046 | 1.24E-02 | 5.31E-02 |
| GDF10 | 2662 | 1.7794702 | 4.1139872 | 5.32E-06 | 9.16E-05 |
| GGACT | 87769 | 1.7780028 | 0.8428936 | 3.31E-05 | 4.40E-04 |
| BAIAP2L2 | 80115 | 1.7732079 | 0.8703117 | 8.88E-02 | 2.19E-01 |
| GFAP | 2670 | 1.770916 | 3.1193661 | 6.86E-02 | 1.84E-01 |
| CENPVL3 | 347549 | 1.7597861 | 4.4818557 | 1.24E-11 | 7.41E-10 |
| BMP7 | 655 | 1.7568316 | 5.6492122 | 4.63E-03 | 2.51E-02 |
| RASGRP1 | 10125 | 1.745821 | 2.5200499 | 1.06E-10 | 5.58E-09 |
| H1-2 | 3006 | 1.7433524 | 4.3966162 | 1.18E-04 | 1.29E-03 |
| ZNF423 | 23090 | 1.7334086 | 4.9883758 | 4.50E-10 | 2.14E-08 |
| INSM1 | 3642 | 1.7322718 | 3.4119018 | 4.78E-02 | 1.42E-01 |
| SCPEP1 | 59342 | 1.7296486 | 5.0370163 | 5.82E-04 | 4.77E-03 |
| BVES | 11149 | 1.7289668 | 1.8498076 | 2.70E-02 | 9.44E-02 |
| LRRC17 | 10234 | 1.7276089 | 1.7855661 | 1.59E-04 | 1.65E-03 |
| PTH1R | 5745 | 1.7238138 | -0.130557 | 2.04E-01 | 3.89E-01 |
| RAD9B | 144715 | 1.7225523 | 0.002876 | 2.86E-03 | 1.71E-02 |
| CLDN3 | 1365 | 1.7163105 | 1.4735357 | 1.00E-02 | 4.52E-02 |
| MICAL2 | 84953 | 1.7153322 | 1.1055446 | 1.12E-02 | 4.92E-02 |
| PAPSS2 | 9060 | 1.7058716 | 2.755136 | 2.52E-02 | 8.99E-02 |
| SDC4 | 6385 | 1.7054083 | 1.8555969 | 1.89E-01 | 3.69E-01 |
| FAM135B | 51059 | 1.7032934 | 1.9274726 | 1.04E-05 | 1.66E-04 |
| ESCO2 | 157570 | 1.7030983 | 0.2588697 | 9.36E-03 | 4.29E-02 |
| STC2 | 8614 | 1.699247 | 2.9793804 | 1.06E-01 | 2.49E-01 |
| SCD | 6319 | 1.6933027 | 8.13838 | 2.69E-04 | 2.53E-03 |
| TP53I3 | 9540 | 1.6930359 | 5.3739596 | 9.37E-21 | 2.14E-18 |
| CDCA5 | 113130 | 1.6909764 | 1.4532153 | 5.74E-03 | 2.99E-02 |
| TMEM191C | 645426 | 1.6861616 | 1.1702463 | 1.50E-02 | 6.12E-02 |
| TMEM31 | 203562 | 1.6856216 | 1.9273438 | 8.04E-02 | 2.05E-01 |
| TNFRSF10A | 8797 | 1.6793 | -0.128833 | 8.29E-02 | 2.09E-01 |
| FAM43A | 131583 | 1.6780877 | 3.593667 | 3.60E-02 | 1.17E-01 |
| SEC24D | 9871 | 1.6774908 | 4.4369317 | 1.23E-03 | 8.71E-03 |
| CD36 | 948 | 1.6753328 | 2.2845695 | 1.01E-04 | 1.14E-03 |
| CHST9 | 83539 | 1.6733873 | 1.2737689 | 1.83E-02 | 7.08E-02 |
| PDE2A | 5138 | 1.6713392 | 3.7938003 | 1.77E-04 | 1.79E-03 |
| RMI2 | 116028 | 1.6696532 | 1.553951 | 4.34E-02 | 1.33E-01 |
| SDSL | 113675 | 1.6691916 | 3.4976187 | 8.23E-04 | 6.33E-03 |
| FBXW4 | 6468 | 1.6691318 | 1.9456982 | 5.48E-07 | 1.25E-05 |
| GPR68 | 8111 | 1.6645468 | 1.7362661 | 6.61E-02 | 1.79E-01 |
| ARHGAP28 | 79822 | 1.6643658 | 1.8100391 | 4.89E-04 | 4.15E-03 |
| EMB | 133418 | 1.6620335 | 2.96105 | 1.12E-07 | 3.14E-06 |
| NR4A2 | 4929 | 1.6615734 | 3.0676166 | 1.56E-02 | 6.28E-02 |
| PCP4L1 | 654790 | 1.6614831 | 6.651729 | 2.38E-01 | 4.30E-01 |
| DUSP16 | 80824 | 1.6614068 | 4.1129779 | 1.93E-02 | 7.38E-02 |
| TMEM45A | 55076 | 1.660449 | 2.0733915 | 1.11E-02 | 4.88E-02 |
| TTC12 | 54970 | 1.6583707 | 2.1514759 | 4.09E-03 | 2.27E-02 |
| TRPC4 | 7223 | 1.6553301 | 2.4216641 | 2.07E-06 | 4.06E-05 |
| STK33 | 65975 | 1.6514026 | 3.7831149 | 3.13E-04 | 2.88E-03 |
| CCNG1 | 900 | 1.6423303 | 7.0376678 | 8.77E-07 | 1.93E-05 |
| RFPL2 | 10739 | 1.6415738 | 2.9412163 | 5.49E-02 | 1.57E-01 |
| OPN1SW | 611 | 1.63804 | 0.1448457 | 1.35E-01 | 2.94E-01 |
| LONRF3 | 79836 | 1.6379022 | 1.1947682 | 1.17E-01 | 2.68E-01 |
| GLIPR1L2 | 144321 | 1.6345023 | 1.1357313 | 1.01E-01 | 2.41E-01 |
| ZNF214 | 7761 | 1.6337862 | 1.7467253 | 1.42E-07 | 3.88E-06 |
| CNGB1 | 1258 | 1.6296157 | 1.1759037 | 2.14E-04 | 2.10E-03 |
| INSIG1 | 3638 | 1.6283956 | 8.1186095 | 3.41E-10 | 1.64E-08 |
| SLC17A6 | 57084 | 1.6253733 | 6.2158299 | 7.72E-02 | 2.00E-01 |
| PTPRM | 5797 | 1.6232258 | 5.17809 | 8.23E-17 | 1.15E-14 |
| ABHD4 | 63874 | 1.6225059 | 5.8013403 | 8.13E-03 | 3.88E-02 |
| MCUB | 55013 | 1.6215007 | 5.4668886 | 4.58E-13 | 3.65E-11 |
| CBX2 | 84733 | 1.6203301 | 2.2788556 | 1.09E-04 | 1.21E-03 |
| ANKRD6 | 22881 | 1.6195524 | 3.620289 | 4.73E-12 | 3.16E-10 |
| H1-0 | 3005 | 1.6188066 | 5.3354086 | 1.19E-02 | 5.15E-02 |
| NSUN7 | 79730 | 1.6136969 | 2.7152422 | 2.25E-06 | 4.35E-05 |
| GTDC1 | 79712 | 1.613468 | 3.5908928 | 1.54E-08 | 5.37E-07 |
| C1S | 716 | 1.6131487 | 3.6498048 | 5.83E-02 | 1.64E-01 |
| RFTN1 | 23180 | 1.6086899 | 6.0875016 | 2.86E-13 | 2.33E-11 |
| DNAH8 | 1769 | 1.6076161 | 0.6956244 | 2.31E-03 | 1.44E-02 |
| RBM24 | 221662 | 1.6026399 | 4.5238518 | 2.43E-07 | 6.18E-06 |
| AFAP1L2 | 84632 | 1.5945776 | 2.9386537 | 9.84E-02 | 2.36E-01 |
| UTS2 | 10911 | 1.5930971 | 2.1898637 | 2.53E-02 | 9.01E-02 |
| SYTL2 | 54843 | 1.5920685 | 3.9277268 | 5.73E-03 | 2.99E-02 |
| C1QTNF1 | 114897 | 1.5893066 | -0.656556 | 5.59E-02 | 1.59E-01 |
| CCDC168 | 643677 | 1.5889366 | 0.0257912 | 1.76E-01 | 3.51E-01 |
| FBN2 | 2201 | 1.5883259 | 1.6408411 | 3.81E-02 | 1.21E-01 |
| H2BC5 | 3017 | 1.5849375 | 4.5806153 | 1.08E-06 | 2.32E-05 |
| SLC15A2 | 6565 | 1.5829817 | 1.9422626 | 3.71E-02 | 1.19E-01 |
| HNF1B | 6928 | 1.5823507 | 0.9519049 | 4.21E-02 | 1.31E-01 |
| KLHDC8B | 200942 | 1.5798404 | 6.0208639 | 2.82E-14 | 2.81E-12 |
| KRT18 | 3875 | 1.5796533 | 4.5521799 | 7.49E-12 | 4.79E-10 |
| ZFYVE9 | 9372 | 1.5790167 | 5.0741068 | 3.91E-11 | 2.18E-09 |
| ADA2 | 51816 | 1.5753282 | 1.9404143 | 2.13E-06 | 4.16E-05 |
| PALM3 | 342979 | 1.5749253 | 2.090893 | 1.46E-01 | 3.10E-01 |
| GNAI1 | 2770 | 1.5728832 | 7.9430658 | 5.39E-17 | 7.75E-15 |
| AGBL4 | 84871 | 1.5722379 | 1.6316639 | 2.77E-03 | 1.67E-02 |
| PLCL1 | 5334 | 1.570647 | 2.9623679 | 3.89E-03 | 2.18E-02 |
| CHRNA5 | 1138 | 1.5624052 | 0.7278689 | 2.71E-03 | 1.64E-02 |
| PPP1R32 | 220004 | 1.5610087 | 1.4530983 | 3.24E-05 | 4.31E-04 |
| RCBTB2 | 1102 | 1.5592537 | 4.2084393 | 1.51E-11 | 8.82E-10 |
| AQP4 | 361 | 1.5576958 | 1.8719349 | 1.57E-02 | 6.32E-02 |
| P4HA3 | 283208 | 1.5556225 | 0.3896288 | 1.45E-01 | 3.08E-01 |
| PPP1R13L | 10848 | 1.5507445 | 0.7122015 | 6.32E-02 | 1.73E-01 |
| H2BC21 | 8349 | 1.5487276 | 5.4091062 | 3.94E-09 | 1.56E-07 |
| TMEM163 | 81615 | 1.5465713 | 5.796209 | 2.33E-05 | 3.29E-04 |
| RPH3A | 22895 | 1.5453168 | 3.0830738 | 1.04E-03 | 7.64E-03 |
| S100B | 6285 | 1.5447307 | 4.1436155 | 1.75E-04 | 1.77E-03 |
| BICC1 | 80114 | 1.543817 | 1.9153094 | 3.60E-02 | 1.16E-01 |
| COL13A1 | 1305 | 1.5432239 | -0.203812 | 6.35E-02 | 1.74E-01 |
| KIF2C | 11004 | 1.5375653 | 1.1187221 | 1.87E-02 | 7.22E-02 |
| UBE2E2 | 7325 | 1.536533 | 7.3468052 | 2.70E-09 | 1.10E-07 |
| HMOX1 | 3162 | 1.5355766 | 6.6190383 | 1.09E-01 | 2.54E-01 |
| CDC6 | 990 | 1.5343346 | 2.2639454 | 6.15E-03 | 3.15E-02 |
| LGI3 | 203190 | 1.5319167 | 1.2245798 | 4.06E-02 | 1.27E-01 |
| ARHGAP12 | 94134 | 1.5273967 | 5.7148603 | 1.80E-08 | 6.18E-07 |
| TMEM178A | 130733 | 1.5269209 | 4.3337558 | 9.13E-04 | 6.88E-03 |
| GHRH | 2691 | 1.5267483 | 1.6468588 | 6.38E-02 | 1.74E-01 |
| CHN2 | 1124 | 1.526018 | 2.0803598 | 4.03E-03 | 2.24E-02 |
| UGT8 | 7368 | 1.5258499 | 1.4046467 | 1.37E-05 | 2.08E-04 |
| SIDT1 | 54847 | 1.5255637 | 0.8939206 | 2.17E-03 | 1.36E-02 |
| LIPG | 9388 | 1.5206878 | 2.8861216 | 3.00E-02 | 1.02E-01 |
| GPR149 | 344758 | 1.5203948 | 1.1804583 | 4.32E-02 | 1.33E-01 |
| CNTN4 | 152330 | 1.5171056 | 3.8188225 | 1.68E-02 | 6.63E-02 |
| DPF3 | 8110 | 1.514811 | 2.2307743 | 6.31E-04 | 5.09E-03 |
| H2BC4 | 8347 | 1.5132189 | 0.0799278 | 4.03E-02 | 1.26E-01 |
| NES | 10763 | 1.5094412 | 4.7577919 | 7.53E-12 | 4.79E-10 |
| SV2B | 9899 | 1.5088723 | 1.194795 | 2.94E-03 | 1.74E-02 |
| EXOC3 | 11336 | 1.5070801 | 3.8045376 | 9.01E-05 | 1.04E-03 |
| COPZ2 | 51226 | 1.5069477 | 1.7318353 | 1.02E-02 | 4.57E-02 |
| EGFL6 | 25975 | 1.5050558 | 3.2862049 | 1.45E-01 | 3.08E-01 |
| EIF5A | 1984 | 1.5017037 | 6.8783032 | 1.04E-11 | 6.37E-10 |
| MFAP5 | 8076 | 1.5009885 | 1.7580308 | 4.28E-01 | 6.25E-01 |
| THAP9 | 79725 | 1.5009767 | 2.0009157 | 3.10E-06 | 5.72E-05 |
| ACTC1 | 70 | 1.498946 | 2.613659 | 2.21E-01 | 4.09E-01 |
| DLX1 | 1745 | 1.4980898 | 6.1940962 | 2.43E-01 | 4.35E-01 |
| CFAP53 | 220136 | 1.4977633 | 2.3166734 | 8.78E-08 | 2.56E-06 |
| CCDC69 | 26112 | 1.495733 | -0.173144 | 6.00E-02 | 1.67E-01 |
| DIAPH3 | 81624 | 1.4945987 | 0.6139584 | 5.94E-02 | 1.66E-01 |
| GPR176 | 11245 | 1.4939786 | 4.8730617 | 4.38E-07 | 1.03E-05 |
| RIOX2 | 84864 | 1.4914262 | 3.8715024 | 9.50E-05 | 1.08E-03 |
| SAMD13 | 148418 | 1.4910399 | 1.7557695 | 9.09E-03 | 4.21E-02 |
| CDHR1 | 92211 | 1.4905119 | 1.9423945 | 4.76E-04 | 4.06E-03 |
| NTRK1 | 4914 | 1.4888906 | 0.0676072 | 7.66E-03 | 3.71E-02 |
| S100PBP | 64766 | 1.4883659 | 4.5826084 | 2.48E-12 | 1.74E-10 |
| YES1 | 7525 | 1.4874008 | 4.2250195 | 2.15E-05 | 3.07E-04 |
| MERTK | 10461 | 1.484942 | 0.1602524 | 1.67E-01 | 3.40E-01 |
| SOS2 | 6655 | 1.4848818 | 4.3555287 | 1.18E-11 | 7.10E-10 |
| PPL | 5493 | 1.4847349 | 1.7035273 | 5.01E-04 | 4.24E-03 |
| DHCR24 | 1718 | 1.4809888 | 7.4586048 | 2.53E-03 | 1.55E-02 |
| GSN | 2934 | 1.4791805 | 5.3384195 | 3.95E-03 | 2.21E-02 |
| GIPC2 | 54810 | 1.4756451 | -0.524153 | 5.35E-02 | 1.54E-01 |
| MEI1 | 150365 | 1.47381 | 1.1431248 | 1.20E-02 | 5.19E-02 |
| SLC7A3 | 84889 | 1.4737714 | 3.240889 | 4.98E-02 | 1.46E-01 |
| TXNRD1 | 7296 | 1.4729679 | 7.7564179 | 3.26E-02 | 1.08E-01 |
| FUT1 | 2523 | 1.4720258 | 3.5089158 | 1.50E-01 | 3.15E-01 |
| SETD9 | 133383 | 1.4716308 | 3.8967771 | 3.87E-11 | 2.17E-09 |
| EAF2 | 55840 | 1.470714 | 2.3049115 | 1.32E-05 | 2.02E-04 |
| NAP1L5 | 266812 | 1.465589 | 8.0153867 | 8.13E-03 | 3.88E-02 |
| EDN1 | 1906 | 1.4654043 | 4.5966224 | 2.29E-01 | 4.20E-01 |
| ST8SIA4 | 7903 | 1.4649009 | 4.4787407 | 6.40E-03 | 3.25E-02 |
| ELOVL5 | 60481 | 1.4602804 | 7.2901241 | 5.72E-10 | 2.68E-08 |
| VAT1L | 57687 | 1.4587115 | 8.2297917 | 2.47E-09 | 1.03E-07 |
| WEE1 | 7465 | 1.4582263 | 4.7844423 | 7.69E-02 | 1.99E-01 |
| TP53BP1 | 7158 | 1.4538346 | 6.2543534 | 2.38E-13 | 1.98E-11 |
| STARD4 | 134429 | 1.4533675 | 4.9017498 | 1.17E-04 | 1.28E-03 |
| ZNF229 | 7772 | 1.4525074 | 2.2036744 | 6.18E-05 | 7.59E-04 |
| ICA1L | 130026 | 1.4508412 | 4.8780901 | 1.12E-04 | 1.24E-03 |
| TP53BP2 | 7159 | 1.449984 | 4.5840844 | 1.98E-09 | 8.44E-08 |
| ZBTB7C | 201501 | 1.4474623 | 1.1210011 | 7.18E-03 | 3.53E-02 |
| LHX8 | 431707 | 1.4472418 | -0.172957 | 3.38E-01 | 5.39E-01 |
| EIF5AL1 | 143244 | 1.4464806 | 1.90138 | 1.13E-03 | 8.17E-03 |
| E2F5 | 1875 | 1.4462693 | 3.8016175 | 6.83E-09 | 2.56E-07 |
| CCT6B | 10693 | 1.443882 | 1.4153678 | 2.86E-05 | 3.91E-04 |
| PRSS23 | 11098 | 1.443871 | 5.6785898 | 1.21E-02 | 5.21E-02 |
| SKAP2 | 8935 | 1.4433769 | 4.1797415 | 1.03E-11 | 6.37E-10 |
| TTF2 | 8458 | 1.4428146 | 1.6426909 | 5.73E-03 | 2.99E-02 |
| PDPN | 10630 | 1.4426023 | 3.8803772 | 1.43E-02 | 5.89E-02 |
| BRCA2 | 675 | 1.442337 | 0.6517905 | 6.81E-02 | 1.83E-01 |
| PCOLCE | 5118 | 1.4422041 | 3.5520762 | 1.71E-02 | 6.73E-02 |
| HRH3 | 11255 | 1.4418796 | 0.8073453 | 4.20E-02 | 1.30E-01 |
| ZNF25 | 219749 | 1.4406941 | 5.7300639 | 5.78E-05 | 7.18E-04 |
| ID3 | 3399 | 1.439565 | 3.7751527 | 4.83E-06 | 8.41E-05 |
| SDC2 | 6383 | 1.4380485 | 8.1635227 | 1.37E-02 | 5.71E-02 |
| CCNJL | 79616 | 1.4351955 | 1.8949665 | 3.59E-02 | 1.16E-01 |
| PLK5 | 126520 | 1.4346807 | 0.4981058 | 9.03E-03 | 4.19E-02 |
| LRRC34 | 151827 | 1.4319091 | 1.62454 | 1.37E-04 | 1.45E-03 |
| TAFA1 | 407738 | 1.4302816 | 2.8925391 | 1.83E-01 | 3.62E-01 |
| DNMT3B | 1789 | 1.4290213 | 1.1270044 | 1.37E-01 | 2.97E-01 |
| DNMT3A | 1788 | 1.4271589 | 4.8924251 | 1.62E-03 | 1.09E-02 |
| SCN4B | 6330 | 1.4246965 | 0.109078 | 4.63E-03 | 2.51E-02 |
| UBXN10 | 127733 | 1.4236177 | 1.5453562 | 4.57E-04 | 3.92E-03 |
| MYO5C | 55930 | 1.4194618 | 2.2404439 | 1.60E-02 | 6.40E-02 |
| MSN | 4478 | 1.4176327 | 6.02986 | 9.94E-03 | 4.49E-02 |
| TNS3 | 64759 | 1.4161429 | 1.4030713 | 1.49E-01 | 3.13E-01 |
| USP47 | 55031 | 1.414252 | 6.3639266 | 8.06E-14 | 7.56E-12 |
| H2BU1 | 128312 | 1.409441 | 1.6308341 | 9.83E-03 | 4.45E-02 |
| MR1 | 3140 | 1.4073671 | 0.1412204 | 3.93E-01 | 5.93E-01 |
| H2BC11 | 8970 | 1.4068915 | 0.121189 | 8.16E-03 | 3.89E-02 |
| ZNF442 | 79973 | 1.4067178 | 2.1298884 | 3.71E-04 | 3.32E-03 |
| MTARC1 | 64757 | 1.4053245 | 3.1237671 | 1.93E-05 | 2.81E-04 |
| ZNF33A | 7581 | 1.4031039 | 5.573628 | 2.41E-06 | 4.58E-05 |
| NARS2 | 79731 | 1.4021171 | 5.0372275 | 1.91E-09 | 8.20E-08 |
| ENPP2 | 5168 | 1.3996908 | 3.4536818 | 1.29E-02 | 5.43E-02 |
| SLC35D2 | 11046 | 1.3990004 | 0.1849355 | 8.94E-02 | 2.20E-01 |
| PLS1 | 5357 | 1.3956904 | 2.016486 | 2.18E-02 | 8.06E-02 |
| KIF15 | 56992 | 1.3953953 | 1.4334641 | 8.39E-03 | 3.97E-02 |
| ENOSF1 | 55556 | 1.3930699 | 1.8766745 | 3.97E-02 | 1.25E-01 |
| RNF39 | 80352 | 1.3927319 | -0.274908 | 1.58E-01 | 3.25E-01 |
| ZDBF2 | 57683 | 1.391031 | 4.3695646 | 2.14E-10 | 1.07E-08 |
| GBP1 | 2633 | 1.3896208 | 1.9978125 | 4.70E-01 | 6.60E-01 |
| KISS1R | 84634 | 1.3872509 | 2.3468373 | 8.99E-02 | 2.21E-01 |
| C2orf74 | 339804 | 1.386543 | 1.3640771 | 4.31E-04 | 3.76E-03 |
| WNT4 | 54361 | 1.385491 | 4.9541278 | 5.98E-02 | 1.67E-01 |
| LIN28B | 389421 | 1.3840397 | 2.193918 | 9.66E-03 | 4.39E-02 |
| ANXA2R | 389289 | 1.3813517 | 2.0462395 | 2.21E-02 | 8.15E-02 |
| XK | 7504 | 1.3807046 | 0.7690523 | 1.43E-02 | 5.88E-02 |
| TAGLN | 6876 | 1.3803788 | 6.589186 | 8.55E-02 | 2.14E-01 |
| DMRTA1 | 63951 | 1.3793256 | 0.9903832 | 8.51E-02 | 2.13E-01 |
| NT5E | 4907 | 1.3774562 | 3.773144 | 2.51E-01 | 4.45E-01 |
| TLCD1 | 116238 | 1.3746559 | 1.7908818 | 1.68E-01 | 3.40E-01 |
| SNX16 | 64089 | 1.3733453 | 3.2757104 | 6.83E-06 | 1.15E-04 |
| PAQR6 | 79957 | 1.3732042 | -0.391232 | 4.67E-03 | 2.52E-02 |
| S100A11 | 6282 | 1.3728309 | 5.8378322 | 3.52E-03 | 2.01E-02 |
| PROCA1 | 147011 | 1.3712887 | 2.2836437 | 1.95E-02 | 7.44E-02 |
| SOX6 | 55553 | 1.3707453 | 2.6164994 | 1.05E-05 | 1.67E-04 |
| KRT19 | 3880 | 1.3705899 | 2.025917 | 5.43E-04 | 4.51E-03 |
| SPATA18 | 132671 | 1.3695593 | 0.9307701 | 1.58E-01 | 3.25E-01 |
| DIS3L | 115752 | 1.3648058 | 4.2438837 | 7.20E-08 | 2.15E-06 |
| SMARCAD1 | 56916 | 1.3640796 | 5.7080102 | 1.35E-05 | 2.06E-04 |
| DSC2 | 1824 | 1.3633493 | 2.3554171 | 1.11E-04 | 1.23E-03 |
| CENPS | 378708 | 1.3623177 | 1.0427552 | 5.50E-03 | 2.90E-02 |
| NPIPB6 | 728741 | 1.3565429 | -0.279461 | 1.05E-01 | 2.48E-01 |
| FAM183A | 440585 | 1.356405 | 1.6170935 | 7.30E-03 | 3.58E-02 |
| GNG2 | 54331 | 1.3557788 | 8.1200183 | 1.87E-02 | 7.21E-02 |
| MORN5 | 254956 | 1.3549392 | 1.5988358 | 3.25E-04 | 2.95E-03 |
| TUBB6 | 84617 | 1.3548629 | 5.6182634 | 7.92E-03 | 3.81E-02 |
| GBE1 | 2632 | 1.354648 | 3.8842918 | 4.17E-02 | 1.30E-01 |
| ZNF248 | 57209 | 1.3507288 | 4.4263607 | 7.20E-10 | 3.31E-08 |
| DRC1 | 92749 | 1.3496541 | 2.4986838 | 7.03E-04 | 5.60E-03 |
| BTK | 695 | 1.3487983 | -0.615614 | 1.74E-01 | 3.49E-01 |
| ADCYAP1 | 116 | 1.3480842 | 4.7990787 | 3.03E-01 | 5.01E-01 |
| EFCAB6 | 64800 | 1.3469518 | 1.3055183 | 3.52E-04 | 3.17E-03 |
| SCN7A | 6332 | 1.3457166 | 0.1974435 | 2.16E-02 | 8.00E-02 |
| TMIE | 259236 | 1.3450109 | -0.099864 | 7.77E-03 | 3.76E-02 |
| C16orf86 | 388284 | 1.3437788 | 1.0909247 | 4.40E-03 | 2.40E-02 |
| P2RX5 | 5026 | 1.3435726 | 2.2774346 | 1.27E-02 | 5.38E-02 |
| CAMK1G | 57172 | 1.3434219 | 4.0577915 | 6.55E-02 | 1.77E-01 |
| LYPD5 | 284348 | 1.3420019 | 1.3586611 | 1.10E-01 | 2.55E-01 |
| MID1IP1 | 58526 | 1.3416936 | 3.1105443 | 1.51E-05 | 2.27E-04 |
| BEND4 | 389206 | 1.3413512 | 2.0390596 | 2.02E-01 | 3.87E-01 |
| AMOT | 154796 | 1.3397822 | 3.3125279 | 1.10E-02 | 4.86E-02 |
| PPP1R3D | 5509 | 1.3375813 | 2.6626205 | 2.47E-06 | 4.68E-05 |
| OSTF1 | 26578 | 1.3372629 | 4.3149746 | 2.63E-08 | 8.75E-07 |
| ODF3L1 | 161753 | 1.3369932 | 0.4928384 | 1.23E-01 | 2.76E-01 |
| SMIM17 | 147670 | 1.3343339 | 3.147337 | 1.01E-02 | 4.54E-02 |
| SLCO1C1 | 53919 | 1.3334227 | 4.9106758 | 1.52E-03 | 1.03E-02 |
| IFI44 | 10561 | 1.3318225 | -0.00838 | 2.87E-01 | 4.85E-01 |
| CCDC175 | 729665 | 1.3296675 | 0.0493623 | 1.40E-01 | 3.02E-01 |
| BBS10 | 79738 | 1.327036 | 3.8393164 | 1.63E-07 | 4.36E-06 |
| ONECUT2 | 9480 | 1.3262655 | 4.4154428 | 1.08E-01 | 2.52E-01 |
| PIK3R1 | 5295 | 1.3256685 | 6.7193725 | 3.48E-04 | 3.13E-03 |
| TSHZ3 | 57616 | 1.3231863 | 2.7640506 | 1.16E-03 | 8.37E-03 |
| PRIM1 | 5557 | 1.3199813 | 4.2836923 | 2.72E-03 | 1.64E-02 |
| CYP27A1 | 1593 | 1.3199717 | 2.3938589 | 8.80E-03 | 4.11E-02 |
| PTPN13 | 5783 | 1.3191646 | 2.21413 | 1.55E-02 | 6.27E-02 |
| ORAI3 | 93129 | 1.3179124 | 2.5167695 | 1.09E-02 | 4.85E-02 |
| DYNLT3 | 6990 | 1.3173498 | 6.9082701 | 1.08E-09 | 4.79E-08 |
| ZNF75D | 7626 | 1.3145171 | 2.8697266 | 1.07E-05 | 1.70E-04 |
| KDM3A | 55818 | 1.3114576 | 4.3993225 | 3.72E-03 | 2.11E-02 |
| GREB1L | 80000 | 1.3111226 | 3.3593214 | 4.39E-07 | 1.03E-05 |
| THNSL2 | 55258 | 1.3107683 | 2.4380025 | 7.48E-05 | 8.90E-04 |
| SC5D | 6309 | 1.3102939 | 8.3821445 | 4.13E-10 | 1.98E-08 |
| SPARCL1 | 8404 | 1.3098296 | 5.0034687 | 3.63E-04 | 3.25E-03 |
| CGNL1 | 84952 | 1.3096442 | 0.9167576 | 2.78E-01 | 4.75E-01 |
| LAYN | 143903 | 1.308847 | 2.5511754 | 4.65E-02 | 1.40E-01 |
| KIAA0319 | 9856 | 1.3076021 | 3.9192167 | 3.99E-03 | 2.23E-02 |
| TMEM132D | 121256 | 1.3072377 | 1.7059439 | 1.62E-02 | 6.46E-02 |
| MXRA8 | 54587 | 1.3066634 | 2.2309451 | 3.16E-04 | 2.90E-03 |
| CERS6 | 253782 | 1.305398 | 5.821337 | 1.47E-03 | 1.00E-02 |
| MCM2 | 4171 | 1.3050466 | 1.8915857 | 2.27E-04 | 2.20E-03 |
| ERC1 | 23085 | 1.302302 | 6.5484145 | 1.53E-07 | 4.14E-06 |
| PRODH | 5625 | 1.3013609 | 0.1553246 | 9.90E-02 | 2.38E-01 |
| CNN3 | 1266 | 1.3004037 | 8.9703413 | 4.77E-06 | 8.34E-05 |
| TNFRSF1A | 7132 | 1.2999612 | 4.4488074 | 6.08E-03 | 3.13E-02 |
| GREB1 | 9687 | 1.2993537 | 2.9182919 | 2.12E-04 | 2.08E-03 |
| DHRS12 | 79758 | 1.2984294 | 1.7796937 | 3.76E-04 | 3.35E-03 |
| FSTL3 | 10272 | 1.2978994 | 3.4440996 | 1.39E-04 | 1.47E-03 |
| ISL1 | 3670 | 1.2956938 | 6.3136496 | 5.51E-02 | 1.58E-01 |
| IQUB | 154865 | 1.294504 | 1.2054393 | 2.75E-03 | 1.66E-02 |
| MT1F | 4494 | 1.2934797 | 4.707206 | 9.29E-03 | 4.27E-02 |
| PPIP5K2 | 23262 | 1.2933543 | 6.3722275 | 8.35E-04 | 6.40E-03 |
| TLR5 | 7100 | 1.2912668 | -0.156975 | 1.48E-01 | 3.12E-01 |
| MYCL | 4610 | 1.2880926 | 2.4254186 | 8.88E-06 | 1.45E-04 |
| POLI | 11201 | 1.2871167 | 3.9709528 | 2.15E-07 | 5.54E-06 |
| RASD1 | 51655 | 1.2853735 | 5.4684465 | 1.09E-02 | 4.84E-02 |
| KLHL4 | 56062 | 1.2847327 | 4.0856954 | 9.57E-07 | 2.09E-05 |
| BCLAF1 | 9774 | 1.2842739 | 7.4360417 | 7.32E-12 | 4.70E-10 |
| VTCN1 | 79679 | 1.2824908 | 0.6067764 | 1.92E-02 | 7.36E-02 |
| AIF1L | 83543 | 1.2819954 | 0.3848847 | 2.38E-02 | 8.61E-02 |
| DNASE1L1 | 1774 | 1.2781171 | 1.6948234 | 1.30E-03 | 9.13E-03 |
| ADAMTS8 | 11095 | 1.2780538 | 0.3031737 | 5.13E-02 | 1.49E-01 |
| PIGP | 51227 | 1.2777202 | 5.7506854 | 2.49E-09 | 1.03E-07 |
| SIK1 | 150094 | 1.2775106 | 0.6557768 | 1.38E-02 | 5.74E-02 |
| CHST15 | 51363 | 1.2768909 | 3.0551134 | 1.77E-03 | 1.17E-02 |
| RCAN2 | 10231 | 1.2765072 | 8.1301533 | 4.23E-04 | 3.70E-03 |
| OTOGL | 283310 | 1.2759376 | 0.6195518 | 4.94E-02 | 1.45E-01 |
| ECT2 | 1894 | 1.2742911 | 2.5852083 | 9.10E-04 | 6.86E-03 |
| KCNH7 | 90134 | 1.2739126 | 3.6618392 | 2.77E-01 | 4.74E-01 |
| DKK2 | 27123 | 1.2728265 | 2.0691781 | 7.24E-02 | 1.91E-01 |
| SYNC | 81493 | 1.2720233 | 3.1851275 | 6.00E-02 | 1.67E-01 |
| DMGDH | 29958 | 1.270936 | -0.209955 | 8.96E-03 | 4.16E-02 |
| HMGCR | 3156 | 1.2702417 | 8.1282244 | 5.99E-12 | 3.90E-10 |
| MBTPS2 | 51360 | 1.2700236 | 5.0786806 | 2.54E-09 | 1.04E-07 |
| KCNN3 | 3782 | 1.2687666 | 4.0884359 | 6.37E-05 | 7.79E-04 |
| VWA3B | 200403 | 1.2671169 | 0.2017118 | 8.55E-02 | 2.14E-01 |
| ZNF112 | 7771 | 1.2625726 | 3.1925534 | 2.26E-04 | 2.20E-03 |
| HLA-DQB1 | 3119 | 1.2616212 | 3.0699502 | 4.09E-02 | 1.28E-01 |
| TMEM97 | 27346 | 1.2610077 | 6.2480865 | 1.15E-08 | 4.16E-07 |
| TMCO3 | 55002 | 1.258939 | 6.2998433 | 1.65E-04 | 1.70E-03 |
| SOBP | 55084 | 1.2588656 | 5.6842234 | 1.25E-02 | 5.33E-02 |
| RNF128 | 79589 | 1.258616 | 2.7629782 | 3.25E-03 | 1.89E-02 |
| FBXL13 | 222235 | 1.2583905 | 1.6389442 | 5.56E-05 | 6.94E-04 |
| FABP7 | 2173 | 1.2539125 | 8.034372 | 6.03E-04 | 4.91E-03 |
| MAP4K3 | 8491 | 1.2534708 | 4.9081446 | 2.40E-09 | 1.00E-07 |
| LRRC37A3 | 374819 | 1.2519044 | 3.1950455 | 1.97E-03 | 1.27E-02 |
| STAT5A | 6776 | 1.251724 | 0.5140898 | 1.44E-02 | 5.92E-02 |
| CHST8 | 64377 | 1.250813 | 4.4616551 | 2.54E-01 | 4.48E-01 |
| C1D | 10438 | 1.2479389 | 4.8444841 | 2.39E-08 | 8.02E-07 |
| NT5C1A | 84618 | 1.2442981 | 0.1526219 | 1.12E-01 | 2.59E-01 |
| DPH3 | 285381 | 1.243708 | 6.7884378 | 1.32E-07 | 3.64E-06 |
| LRRC9 | 341883 | 1.2427582 | 0.4788728 | 3.14E-02 | 1.05E-01 |
| ZNF521 | 25925 | 1.2418118 | 5.4196436 | 3.35E-08 | 1.09E-06 |
| RASGEF1B | 153020 | 1.2382776 | 5.2957016 | 2.15E-02 | 7.98E-02 |
| HTRA1 | 5654 | 1.2377405 | 6.7277833 | 4.13E-02 | 1.29E-01 |
| PRDM12 | 59335 | 1.2364574 | 2.2536923 | 2.14E-01 | 4.02E-01 |
| PIK3IP1 | 113791 | 1.234208 | 2.5079668 | 1.06E-02 | 4.74E-02 |
| PAWR | 5074 | 1.2322468 | 4.4588258 | 2.38E-02 | 8.60E-02 |
| RPS6KA5 | 9252 | 1.2321309 | 2.6871845 | 2.32E-05 | 3.27E-04 |
| PIAS3 | 10401 | 1.2310128 | 3.9843692 | 5.70E-03 | 2.98E-02 |
| AR | 367 | 1.2308015 | 0.9543696 | 5.87E-02 | 1.65E-01 |
| KCTD11 | 147040 | 1.2307734 | 3.3835436 | 7.84E-04 | 6.09E-03 |
| MFSD4A | 148808 | 1.2288726 | 2.6354304 | 5.23E-02 | 1.51E-01 |
| MYO10 | 4651 | 1.2286934 | 5.1791498 | 5.64E-09 | 2.18E-07 |
| GABRE | 2564 | 1.2286145 | 2.0873334 | 9.77E-04 | 7.26E-03 |
| FBXO7 | 25793 | 1.2253111 | 7.1197288 | 1.34E-06 | 2.79E-05 |
| NEB | 4703 | 1.2247845 | 1.0954562 | 8.89E-02 | 2.20E-01 |
| CCNT2 | 905 | 1.2234265 | 5.2166394 | 1.91E-08 | 6.51E-07 |
| CERS5 | 91012 | 1.2227197 | 6.5870093 | 3.04E-04 | 2.81E-03 |
| IQCN | 80726 | 1.2222823 | 0.7000071 | 9.48E-02 | 2.30E-01 |
| CDYL2 | 124359 | 1.2203088 | 3.3680418 | 2.01E-05 | 2.91E-04 |
| NMRK2 | 27231 | 1.2193291 | 1.0053894 | 2.18E-01 | 4.06E-01 |
| ETS2 | 2114 | 1.2187905 | 4.4191574 | 4.90E-06 | 8.52E-05 |
| CA7 | 766 | 1.2171723 | 0.487692 | 2.11E-01 | 3.98E-01 |
| MAGI3 | 260425 | 1.2169698 | 4.5079252 | 5.75E-06 | 9.81E-05 |
| PGAM4 | 441531 | 1.2163648 | -0.114655 | 8.47E-02 | 2.12E-01 |
| TSPAN1 | 10103 | 1.2136641 | 2.5963257 | 1.00E-02 | 4.51E-02 |
| RHPN2 | 85415 | 1.2124574 | 2.7095316 | 3.39E-02 | 1.12E-01 |
| MVP | 9961 | 1.2124059 | 3.9202317 | 1.31E-01 | 2.89E-01 |
| OAZ3 | 51686 | 1.2114886 | -0.128339 | 7.40E-02 | 1.94E-01 |
| TGFBR1 | 7046 | 1.2111779 | 4.6232487 | 2.31E-04 | 2.23E-03 |
| TRIP13 | 9319 | 1.2104464 | 4.934879 | 3.74E-04 | 3.34E-03 |
| SCN9A | 6335 | 1.2099166 | 4.8447116 | 3.73E-02 | 1.20E-01 |
| TEAD1 | 7003 | 1.2093042 | 4.6751564 | 1.46E-03 | 9.97E-03 |
| RHEBL1 | 121268 | 1.2085128 | 2.6884654 | 9.16E-02 | 2.24E-01 |
| POLE2 | 5427 | 1.2081549 | 1.8344927 | 1.41E-03 | 9.69E-03 |
| CRYZ | 1429 | 1.2075866 | 5.0363699 | 1.32E-05 | 2.03E-04 |
| BCAT1 | 586 | 1.2074597 | 6.0058966 | 8.64E-10 | 3.89E-08 |
| TMEM255B | 348013 | 1.2074299 | 1.6200362 | 6.66E-02 | 1.80E-01 |
| CLCN5 | 1184 | 1.2070317 | 3.6721271 | 6.37E-04 | 5.13E-03 |
| RCAN3 | 11123 | 1.2066515 | 4.9112138 | 1.15E-02 | 5.03E-02 |
| VASN | 114990 | 1.2061043 | 2.1427485 | 5.27E-01 | 7.07E-01 |
| CD72 | 971 | 1.2055971 | -0.456697 | 1.37E-01 | 2.97E-01 |
| SESN1 | 27244 | 1.2051387 | 4.4288588 | 1.69E-03 | 1.13E-02 |
| ANKRD13A | 88455 | 1.2034508 | 4.295539 | 2.15E-05 | 3.07E-04 |
| RMI1 | 80010 | 1.2025269 | 4.3699252 | 4.90E-07 | 1.13E-05 |
| NPPB | 4879 | 1.2024494 | 0.631484 | 1.35E-02 | 5.65E-02 |
| ALCAM | 214 | 1.2020384 | 6.7689823 | 2.91E-05 | 3.97E-04 |
| TEX45 | 374877 | 1.2015802 | 0.5117448 | 2.22E-01 | 4.11E-01 |
| CKAP2L | 150468 | 1.2005113 | 1.0051914 | 1.01E-01 | 2.41E-01 |
| ZNF19 | 7567 | 1.1988157 | 2.7131936 | 3.04E-06 | 5.63E-05 |
| ARAP1 | 116985 | 1.1978574 | 1.1045351 | 1.08E-01 | 2.53E-01 |
| MAP2K4 | 6416 | 1.1966849 | 6.9731311 | 6.32E-11 | 3.44E-09 |
| ZNF233 | 353355 | 1.1961618 | 2.6004635 | 3.85E-03 | 2.17E-02 |
| RHCE | 6006 | 1.1952722 | 1.6665362 | 7.39E-04 | 5.81E-03 |
| DACH2 | 117154 | 1.1945914 | 6.4234363 | 2.88E-01 | 4.87E-01 |
| GSC2 | 2928 | 1.1939626 | -0.545848 | 2.85E-01 | 4.83E-01 |
| GZF1 | 64412 | 1.1936419 | 3.619823 | 1.13E-05 | 1.78E-04 |
| PSMD5 | 5711 | 1.1924112 | 4.8162902 | 5.00E-05 | 6.31E-04 |
| FSTL1 | 11167 | 1.1914449 | 6.7258948 | 5.65E-02 | 1.60E-01 |
| POU6F2 | 11281 | 1.1914176 | 4.4031414 | 9.14E-04 | 6.89E-03 |
| COL4A6 | 1288 | 1.1896462 | 2.9818845 | 1.02E-01 | 2.42E-01 |
| TCN2 | 6948 | 1.1892527 | 4.7968181 | 1.97E-07 | 5.11E-06 |
| FADS2 | 9415 | 1.1888332 | 7.7525514 | 6.41E-03 | 3.25E-02 |
| CFC1 | 55997 | 1.18826 | 3.5445122 | 2.42E-03 | 1.49E-02 |
| WNT10B | 7480 | 1.1875916 | 2.7401342 | 8.68E-03 | 4.07E-02 |
| SLC50A1 | 55974 | 1.1875195 | 5.1726574 | 5.50E-04 | 4.55E-03 |
| TJP3 | 27134 | 1.1874048 | 1.6862069 | 4.42E-02 | 1.35E-01 |
| MASP1 | 5648 | 1.187402 | 2.5495478 | 1.19E-04 | 1.30E-03 |
| AMFR | 267 | 1.1868222 | 5.7577232 | 1.64E-03 | 1.10E-02 |
| SLC25A30 | 253512 | 1.186058 | 0.7241448 | 8.93E-03 | 4.16E-02 |
| SHB | 6461 | 1.1860493 | 1.6641433 | 6.11E-04 | 4.96E-03 |
| STEAP1 | 26872 | 1.1830234 | 0.9269473 | 9.76E-03 | 4.43E-02 |
| JUN | 3725 | 1.1830112 | 5.8319782 | 2.54E-06 | 4.81E-05 |
| PDE1A | 5136 | 1.1783995 | 3.5913659 | 3.48E-02 | 1.14E-01 |
| TIMELESS | 8914 | 1.177137 | 2.6341596 | 1.49E-04 | 1.56E-03 |
| ELN | 2006 | 1.1757872 | -0.053013 | 1.76E-01 | 3.52E-01 |
| CALCOCO2 | 10241 | 1.1754145 | 5.0623947 | 2.51E-03 | 1.54E-02 |
| TMEM150C | 441027 | 1.1750532 | 4.3757323 | 4.27E-03 | 2.35E-02 |
| CRB2 | 286204 | 1.1742177 | 0.9361698 | 1.74E-01 | 3.49E-01 |
| NRP1 | 8829 | 1.1735658 | 4.8204778 | 7.09E-05 | 8.48E-04 |
| NAP1L4 | 4676 | 1.1735148 | 6.9127048 | 1.07E-09 | 4.75E-08 |
| SELENOP | 6414 | 1.1720978 | 1.6530929 | 7.69E-02 | 1.99E-01 |
| UBE4A | 9354 | 1.1715603 | 5.0654757 | 1.42E-08 | 5.02E-07 |
| CPNE9 | 151835 | 1.1711709 | 0.7958079 | 2.11E-01 | 3.98E-01 |
| CRYBG2 | 55057 | 1.1688966 | 1.3322395 | 1.34E-02 | 5.61E-02 |
| SMPDL3A | 10924 | 1.1688064 | 4.5355307 | 5.95E-09 | 2.28E-07 |
| ZNF608 | 57507 | 1.1685775 | 4.6251462 | 1.41E-04 | 1.49E-03 |
| PNMA2 | 10687 | 1.1683986 | 6.8944181 | 3.22E-05 | 4.31E-04 |
| RAD17 | 5884 | 1.1676161 | 5.0220402 | 7.83E-08 | 2.31E-06 |
| ATOH8 | 84913 | 1.166774 | 1.2437867 | 2.35E-01 | 4.27E-01 |
| LURAP1 | 541468 | 1.1665838 | 1.0860709 | 8.28E-03 | 3.93E-02 |
| CCNK | 8812 | 1.166017 | 2.8331818 | 1.38E-04 | 1.46E-03 |
| ELOVL1 | 64834 | 1.165845 | 3.9274489 | 5.12E-02 | 1.49E-01 |
| PLA2G3 | 50487 | 1.1657421 | 0.8022795 | 4.82E-02 | 1.43E-01 |
| CENPK | 64105 | 1.1650094 | 2.273748 | 3.37E-02 | 1.11E-01 |
| EHD2 | 30846 | 1.164656 | 1.4382696 | 1.25E-01 | 2.79E-01 |
| KNL1 | 57082 | 1.16356 | 0.0045744 | 5.71E-02 | 1.61E-01 |
| IGFBP7 | 3490 | 1.1633182 | 5.1899759 | 1.96E-01 | 3.79E-01 |
| INKA2 | 55924 | 1.1631333 | 1.6900119 | 2.31E-02 | 8.41E-02 |
| C3orf80 | 401097 | 1.1618251 | 0.6965488 | 3.24E-02 | 1.08E-01 |
| KCNT2 | 343450 | 1.16048 | 1.280392 | 8.59E-03 | 4.05E-02 |
| FUCA2 | 2519 | 1.1588419 | 5.4721109 | 1.14E-04 | 1.25E-03 |
| DZIP3 | 9666 | 1.1581181 | 6.6820853 | 5.72E-06 | 9.77E-05 |
| KCNIP2 | 30819 | 1.1577253 | 4.3045738 | 7.63E-02 | 1.98E-01 |
| SNX4 | 8723 | 1.1545225 | 7.3978098 | 4.75E-08 | 1.49E-06 |
| ZNF568 | 374900 | 1.1530897 | 4.5021734 | 1.34E-07 | 3.67E-06 |
| PTPRR | 5801 | 1.1530155 | 4.491017 | 7.35E-02 | 1.93E-01 |
| KCNJ6 | 3763 | 1.1517446 | 4.7712316 | 7.17E-03 | 3.53E-02 |
| STAU1 | 6780 | 1.1511147 | 7.1539003 | 7.04E-10 | 3.25E-08 |
| TTC39A | 22996 | 1.1505968 | 3.7622403 | 1.29E-01 | 2.86E-01 |
| CAST | 831 | 1.150555 | 5.2834161 | 4.11E-06 | 7.31E-05 |
| UNC5A | 90249 | 1.1498177 | 2.6088925 | 1.55E-01 | 3.21E-01 |
| CCNDBP1 | 23582 | 1.1497584 | 5.9071082 | 6.03E-08 | 1.82E-06 |
| KCTD8 | 386617 | 1.1496387 | 3.0867308 | 3.53E-02 | 1.15E-01 |
| TNS1 | 7145 | 1.1496151 | 1.4520515 | 9.64E-04 | 7.18E-03 |
| TTK | 7272 | 1.1483313 | 0.7731882 | 6.63E-02 | 1.79E-01 |
| CPM | 1368 | 1.1482232 | 0.3863742 | 3.63E-01 | 5.62E-01 |
| FIG4 | 9896 | 1.148153 | 6.1136997 | 4.35E-10 | 2.07E-08 |
| FBXO38 | 81545 | 1.1472858 | 5.0531694 | 1.11E-08 | 4.01E-07 |
| HUNK | 30811 | 1.1471577 | 4.0871124 | 3.81E-02 | 1.21E-01 |
| RNF32 | 140545 | 1.146848 | 2.0073474 | 1.15E-03 | 8.30E-03 |
| EIF3A | 8661 | 1.1449614 | 6.7561857 | 2.82E-05 | 3.86E-04 |
| C5AR1 | 728 | 1.1438544 | -0.005063 | 3.66E-02 | 1.18E-01 |
| GXYLT1 | 283464 | 1.1434841 | 2.2995814 | 1.41E-02 | 5.84E-02 |
| NMRK1 | 54981 | 1.1428848 | 4.2714517 | 1.79E-06 | 3.56E-05 |
| BBC3 | 27113 | 1.142707 | 2.0370644 | 2.02E-02 | 7.62E-02 |
| DTNA | 1837 | 1.1425966 | 5.9163682 | 5.77E-10 | 2.69E-08 |
| HIF1A | 3091 | 1.1423835 | 7.0262717 | 2.73E-05 | 3.76E-04 |
| MYH15 | 22989 | 1.1395655 | 1.2051549 | 3.84E-03 | 2.16E-02 |
| CPNE7 | 27132 | 1.1370786 | 2.736034 | 9.21E-03 | 4.24E-02 |
| FBXO27 | 126433 | 1.1327247 | 3.669212 | 2.26E-05 | 3.20E-04 |
| CLSTN2 | 64084 | 1.132215 | 3.3050972 | 2.21E-06 | 4.29E-05 |
| TMEM64 | 169200 | 1.1308017 | 4.2101981 | 1.41E-02 | 5.82E-02 |
| TLE6 | 79816 | 1.1287472 | 0.9310271 | 1.81E-02 | 7.03E-02 |
| TAF1 | 6872 | 1.1281529 | 2.5351099 | 1.66E-04 | 1.70E-03 |
| KLHL9 | 55958 | 1.1277416 | 6.4165963 | 1.59E-09 | 6.89E-08 |
| PWWP3B | 139221 | 1.1269932 | 3.2213748 | 2.25E-06 | 4.35E-05 |
| FAM13B | 51306 | 1.1262224 | 5.3641382 | 6.38E-08 | 1.92E-06 |
| SIAE | 54414 | 1.1251712 | 4.8618096 | 1.25E-05 | 1.93E-04 |
| SOCS3 | 9021 | 1.1249386 | 1.3248858 | 1.33E-01 | 2.92E-01 |
| LIMK2 | 3985 | 1.1249269 | 4.9708036 | 3.35E-05 | 4.44E-04 |
| TSPYL4 | 23270 | 1.1245862 | 5.8374707 | 1.39E-02 | 5.78E-02 |
| ZCCHC18 | 644353 | 1.1233883 | 5.1276829 | 1.21E-02 | 5.19E-02 |
| PRLR | 5618 | 1.1229223 | 0.9518371 | 6.26E-02 | 1.72E-01 |
| GPR61 | 83873 | 1.1228857 | 2.6241596 | 9.23E-02 | 2.25E-01 |
| AMPH | 273 | 1.1226607 | 5.9352071 | 2.30E-03 | 1.43E-02 |
| C10orf82 | 143379 | 1.121574 | 3.0855363 | 1.03E-01 | 2.45E-01 |
| AVEN | 57099 | 1.1214748 | 4.1316678 | 3.88E-07 | 9.30E-06 |
| FAM110C | 642273 | 1.1205795 | -0.051639 | 1.15E-01 | 2.64E-01 |
| DTX4 | 23220 | 1.1202283 | 4.6318899 | 2.82E-03 | 1.69E-02 |
| POPDC3 | 64208 | 1.1195094 | 2.7719253 | 3.87E-04 | 3.44E-03 |
| PHF19 | 26147 | 1.1190247 | 1.0773404 | 9.02E-04 | 6.82E-03 |
| MPP7 | 143098 | 1.1180871 | 2.3516921 | 8.56E-04 | 6.52E-03 |
| NRN1 | 51299 | 1.1180232 | 5.9645824 | 7.81E-06 | 1.29E-04 |
| ZBTB18 | 10472 | 1.1170886 | 2.0163139 | 2.51E-04 | 2.38E-03 |
| FRAT1 | 10023 | 1.1157171 | 1.0474337 | 6.54E-03 | 3.29E-02 |
| CTBS | 1486 | 1.114768 | 2.035546 | 6.07E-02 | 1.69E-01 |
| NBPF10 | 100132406 | 1.1131077 | -0.535161 | 2.52E-01 | 4.46E-01 |
| ARL15 | 54622 | 1.1128314 | 5.7695079 | 4.80E-06 | 8.38E-05 |
| ENKUR | 219670 | 1.112767 | 3.3819857 | 5.66E-03 | 2.96E-02 |
| SSX2IP | 117178 | 1.1118699 | 4.3454406 | 1.58E-04 | 1.63E-03 |
| GPX8 | 493869 | 1.1117114 | 4.3483283 | 7.24E-02 | 1.91E-01 |
| MBP | 4155 | 1.1113079 | 4.8203284 | 1.27E-04 | 1.37E-03 |
| ZNF100 | 163227 | 1.1108076 | 1.6172807 | 1.93E-02 | 7.39E-02 |
| HSPB8 | 26353 | 1.1106653 | 1.6278525 | 1.34E-02 | 5.61E-02 |
| TEAD2 | 8463 | 1.1100467 | 0.0220008 | 4.90E-01 | 6.77E-01 |
| DDIT4 | 54541 | 1.1100117 | 5.4922069 | 2.09E-02 | 7.82E-02 |
| ZNF273 | 10793 | 1.1093265 | 3.4296948 | 1.93E-02 | 7.37E-02 |
| TTC8 | 123016 | 1.1082013 | 4.876345 | 2.06E-04 | 2.03E-03 |
| ACYP2 | 98 | 1.1080579 | 5.8599581 | 3.85E-06 | 6.91E-05 |
| MAFF | 23764 | 1.1071331 | 4.2905263 | 7.64E-03 | 3.71E-02 |
| VPS41 | 27072 | 1.1062355 | 6.3270298 | 1.25E-07 | 3.48E-06 |
| ZFAND4 | 93550 | 1.105438 | 3.6649318 | 3.03E-05 | 4.10E-04 |
| EPS8 | 2059 | 1.1051654 | 6.2567851 | 1.46E-02 | 5.98E-02 |
| CHST7 | 56548 | 1.1046539 | 3.5954095 | 9.61E-03 | 4.37E-02 |
| RSPH4A | 345895 | 1.1040951 | 2.7554926 | 9.63E-04 | 7.17E-03 |
| CTSO | 1519 | 1.1033354 | 3.028561 | 4.12E-02 | 1.29E-01 |
| CMTM1 | 113540 | 1.1032527 | 0.3760744 | 6.42E-02 | 1.75E-01 |
| CMAS | 55907 | 1.1029705 | 7.960655 | 1.61E-05 | 2.41E-04 |
| PRKACB | 5567 | 1.1025361 | 7.245422 | 6.93E-03 | 3.44E-02 |
| EFL1 | 79631 | 1.1021631 | 3.7432488 | 1.43E-05 | 2.16E-04 |
| LITAF | 9516 | 1.1009915 | 5.0506886 | 1.26E-02 | 5.35E-02 |
| RCOR3 | 55758 | 1.1008405 | 5.1895049 | 1.75E-08 | 6.02E-07 |
| DNAH9 | 1770 | 1.0996696 | 3.2917576 | 1.10E-04 | 1.22E-03 |
| EPB41 | 2035 | 1.0994778 | 4.5036464 | 1.75E-03 | 1.16E-02 |
| EML1 | 2009 | 1.099419 | 4.9463207 | 1.28E-02 | 5.42E-02 |
| TMEM108 | 66000 | 1.0987854 | 5.1121763 | 1.50E-07 | 4.08E-06 |
| DNAH14 | 127602 | 1.0987612 | 4.6533764 | 4.93E-05 | 6.23E-04 |
| TNKS2 | 80351 | 1.0974412 | 6.0597915 | 2.35E-07 | 6.01E-06 |
| ZNF285 | 26974 | 1.0972224 | 3.6207899 | 5.35E-04 | 4.46E-03 |
| USP51 | 158880 | 1.0965997 | 1.8411896 | 1.82E-02 | 7.07E-02 |
| SLC12A1 | 6557 | 1.0962891 | 2.0795246 | 3.28E-01 | 5.29E-01 |
| RNF112 | 7732 | 1.0958918 | 1.3251351 | 1.12E-01 | 2.59E-01 |
| CHD7 | 55636 | 1.0953961 | 4.8705264 | 9.21E-06 | 1.49E-04 |
| PTGFR | 5737 | 1.0944023 | 0.1394906 | 3.97E-02 | 1.25E-01 |
| PNPLA3 | 80339 | 1.0942497 | 5.8167785 | 1.08E-05 | 1.71E-04 |
| UBE2Q2 | 92912 | 1.093693 | 5.3428886 | 4.71E-08 | 1.48E-06 |
| RAB31 | 11031 | 1.0934201 | 7.6041264 | 1.27E-05 | 1.96E-04 |
| MNS1 | 55329 | 1.0922857 | 1.2693922 | 5.96E-02 | 1.66E-01 |
| UBE2E3 | 10477 | 1.0908568 | 7.8146954 | 3.19E-04 | 2.92E-03 |
| PTPRO | 5800 | 1.0905707 | 6.6292815 | 9.58E-02 | 2.31E-01 |
| CNST | 163882 | 1.0898349 | 5.5886722 | 4.52E-04 | 3.90E-03 |
| ZNF418 | 147686 | 1.0895199 | 3.0487257 | 1.79E-04 | 1.81E-03 |
| TMPPE | 643853 | 1.0891067 | 0.0224658 | 1.23E-01 | 2.77E-01 |
| MIA3 | 375056 | 1.0880004 | 6.1335709 | 2.11E-07 | 5.45E-06 |
| NCBP3 | 55421 | 1.087837 | 5.2216021 | 4.48E-05 | 5.70E-04 |
| HSF4 | 3299 | 1.0877819 | 0.8934402 | 3.23E-01 | 5.24E-01 |
| NPAS1 | 4861 | 1.0874665 | 2.8337413 | 4.33E-04 | 3.77E-03 |
| ARCN1 | 372 | 1.087268 | 6.9559507 | 6.51E-06 | 1.10E-04 |
| ACAT2 | 39 | 1.0866995 | 9.077404 | 1.05E-08 | 3.83E-07 |
| SOGA3 | 387104 | 1.0845843 | 6.2105884 | 2.17E-02 | 8.04E-02 |
| SFMBT2 | 57713 | 1.0840024 | 3.1457226 | 4.88E-02 | 1.44E-01 |
| PMS1 | 5378 | 1.084002 | 4.5769365 | 1.55E-06 | 3.15E-05 |
| MGME1 | 92667 | 1.0830323 | 2.8009747 | 3.00E-04 | 2.77E-03 |
| TET1 | 80312 | 1.0819686 | 4.5150778 | 2.13E-03 | 1.35E-02 |
| SMARCA5 | 8467 | 1.081318 | 6.4303324 | 1.12E-05 | 1.76E-04 |
| TNFRSF21 | 27242 | 1.0811923 | 6.0380461 | 4.39E-06 | 7.78E-05 |
| PLPP2 | 8612 | 1.0807246 | 2.4148746 | 2.29E-04 | 2.21E-03 |
| CDC42SE2 | 56990 | 1.0807211 | 5.486665 | 1.22E-06 | 2.57E-05 |
| PIK3R3 | 8503 | 1.0807074 | 6.3774078 | 6.04E-03 | 3.12E-02 |
| KLHL12 | 59349 | 1.0805801 | 6.1157088 | 4.26E-08 | 1.35E-06 |
| GPR139 | 124274 | 1.0780669 | 0.2697953 | 1.44E-01 | 3.06E-01 |
| HCRT | 3060 | 1.0769529 | 1.0542554 | 8.75E-02 | 2.17E-01 |
| CCNB2 | 9133 | 1.0765439 | 2.0767041 | 3.90E-02 | 1.23E-01 |
| ZNF550 | 162972 | 1.0762471 | 3.4484481 | 1.15E-05 | 1.81E-04 |
| FAM111B | 374393 | 1.0755276 | 1.398831 | 2.91E-01 | 4.90E-01 |
| SLC8A3 | 6547 | 1.0755226 | 3.9184364 | 2.69E-01 | 4.65E-01 |
| LDLR | 3949 | 1.0754394 | 5.5090591 | 7.81E-03 | 3.77E-02 |
| JHY | 79864 | 1.0739837 | 3.8941448 | 2.04E-05 | 2.94E-04 |
| ZNF182 | 7569 | 1.0719656 | 2.9550397 | 1.00E-03 | 7.41E-03 |
| TYW1B | 441250 | 1.0713158 | 1.6752692 | 2.88E-03 | 1.72E-02 |
| LACC1 | 144811 | 1.0708739 | 2.54276 | 3.37E-03 | 1.95E-02 |
| TAF9B | 51616 | 1.0703917 | 5.7337613 | 3.76E-07 | 9.05E-06 |
| NOTCH4 | 4855 | 1.0699919 | 2.9717787 | 3.24E-04 | 2.94E-03 |
| MARVELD2 | 153562 | 1.0694202 | 0.5849688 | 2.50E-01 | 4.43E-01 |
| MEST | 4232 | 1.0690468 | 7.5579471 | 2.70E-06 | 5.05E-05 |
| POPDC2 | 64091 | 1.0687551 | 1.5904078 | 3.47E-03 | 2.00E-02 |
| DMTF1 | 9988 | 1.0685932 | 5.4762554 | 2.78E-05 | 3.81E-04 |
| GPD2 | 2820 | 1.0685303 | 3.6695044 | 5.46E-03 | 2.88E-02 |
| EIF4E1B | 253314 | 1.0684566 | 0.1212078 | 2.62E-01 | 4.57E-01 |
| KCTD7 | 154881 | 1.068454 | 5.8571353 | 1.47E-06 | 3.02E-05 |
| TRMT1L | 81627 | 1.0653799 | 4.293903 | 1.86E-07 | 4.88E-06 |
| ADGRD1 | 283383 | 1.0642595 | 0.4419393 | 8.05E-03 | 3.85E-02 |
| LMOD1 | 25802 | 1.0637203 | -0.274772 | 4.03E-01 | 6.04E-01 |
| SLC25A36 | 55186 | 1.0634302 | 7.3657451 | 7.06E-05 | 8.47E-04 |
| RAB6C | 84084 | 1.0630074 | 0.8013824 | 3.70E-02 | 1.19E-01 |
| NT5C3A | 51251 | 1.0627158 | 6.8281337 | 2.06E-03 | 1.31E-02 |
| ZRANB3 | 84083 | 1.0625616 | 2.2571961 | 1.26E-03 | 8.87E-03 |
| L3MBTL2 | 83746 | 1.060727 | 4.089312 | 8.92E-06 | 1.45E-04 |
| IMPA2 | 3613 | 1.059811 | 2.3528465 | 3.86E-01 | 5.87E-01 |
| LCOR | 84458 | 1.0592085 | 6.0450486 | 7.87E-05 | 9.24E-04 |
| DBP | 1628 | 1.0589592 | 2.295253 | 2.67E-03 | 1.62E-02 |
| ADGRL4 | 64123 | 1.0589049 | 1.6254994 | 3.46E-01 | 5.46E-01 |
| IFRD1 | 3475 | 1.0588704 | 4.6103124 | 1.39E-06 | 2.87E-05 |
| FLOT2 | 2319 | 1.0583657 | 6.3344851 | 1.02E-04 | 1.14E-03 |
| TUFT1 | 7286 | 1.0578376 | 4.7318034 | 1.38E-07 | 3.76E-06 |
| RGS10 | 6001 | 1.0577324 | 0.3528906 | 5.59E-02 | 1.59E-01 |
| ELK3 | 2004 | 1.0572026 | 2.1668495 | 2.85E-01 | 4.83E-01 |
| SMN1 | 6606 | 1.0570931 | 4.3341079 | 5.59E-06 | 9.57E-05 |
| CCDC30 | 728621 | 1.0566017 | 5.0469825 | 4.91E-05 | 6.21E-04 |
| CDC42BPB | 9578 | 1.0555654 | 4.6760718 | 8.72E-06 | 1.42E-04 |
| R3HCC1L | 27291 | 1.0552044 | 3.8023528 | 2.34E-03 | 1.45E-02 |
| TUT7 | 79670 | 1.0547459 | 4.3512851 | 2.59E-06 | 4.88E-05 |
| CLHC1 | 130162 | 1.0544786 | 3.608049 | 2.38E-05 | 3.34E-04 |
| SEZ6L | 23544 | 1.054376 | 5.4234954 | 2.59E-01 | 4.54E-01 |
| ANKRD44 | 91526 | 1.0540546 | 4.2421235 | 2.19E-03 | 1.38E-02 |
| CFC1B | 653275 | 1.0539501 | 0.0686191 | 2.32E-02 | 8.43E-02 |
| CDKN1C | 1028 | 1.0536778 | 4.1585669 | 5.83E-06 | 9.92E-05 |
| GCH1 | 2643 | 1.0526372 | 3.649582 | 2.74E-02 | 9.54E-02 |
| MTMR2 | 8898 | 1.0524536 | 6.1559138 | 2.24E-07 | 5.75E-06 |
| H2BC12 | 85236 | 1.05221 | 3.9904299 | 1.19E-02 | 5.16E-02 |
| MAP3K14 | 9020 | 1.0517141 | 0.6586898 | 1.51E-01 | 3.16E-01 |
| CNGA3 | 1261 | 1.0512248 | 1.9357769 | 2.46E-01 | 4.39E-01 |
| ZFR | 51663 | 1.049438 | 7.7866304 | 4.58E-09 | 1.79E-07 |
| ACBD7 | 414149 | 1.0489058 | 0.809822 | 1.09E-02 | 4.85E-02 |
| ROPN1L | 83853 | 1.047973 | 2.5984544 | 3.17E-04 | 2.90E-03 |
| LRRC23 | 10233 | 1.0464422 | 4.2882147 | 7.49E-08 | 2.22E-06 |
| TAC3 | 6866 | 1.046109 | 1.7257736 | 3.19E-01 | 5.19E-01 |
| DUSP6 | 1848 | 1.0452485 | 6.0638375 | 2.23E-02 | 8.21E-02 |
| ERI1 | 90459 | 1.0447 | 3.9511342 | 3.74E-03 | 2.12E-02 |
| INVS | 27130 | 1.043902 | 3.2509005 | 5.74E-04 | 4.72E-03 |
| ZFP28 | 140612 | 1.0435207 | 4.6101359 | 2.58E-04 | 2.44E-03 |
| PAICS | 10606 | 1.0426444 | 6.0751989 | 1.83E-03 | 1.19E-02 |
| BTBD10 | 84280 | 1.0420694 | 7.7676816 | 8.03E-07 | 1.78E-05 |
| SLC2A3 | 6515 | 1.0420257 | 7.6556604 | 3.51E-02 | 1.15E-01 |
| LHX5 | 64211 | 1.0401254 | 1.4473211 | 1.41E-01 | 3.03E-01 |
| PLPP5 | 84513 | 1.04009 | 5.8510895 | 5.02E-08 | 1.55E-06 |
| CYP1A1 | 1543 | 1.0390951 | 0.4800967 | 2.88E-02 | 9.87E-02 |
| PMFBP1 | 83449 | 1.037318 | 0.2811938 | 3.23E-02 | 1.08E-01 |
| N6AMT1 | 29104 | 1.0358829 | 3.4739242 | 2.70E-04 | 2.53E-03 |
| L3MBTL3 | 84456 | 1.035581 | 3.3995503 | 7.07E-04 | 5.62E-03 |
| NID2 | 22795 | 1.0355763 | 0.0670351 | 9.48E-03 | 4.34E-02 |
| TTI1 | 9675 | 1.0354485 | 4.2121173 | 2.73E-06 | 5.11E-05 |
| C1orf162 | 128346 | 1.0353686 | -0.276472 | 3.12E-01 | 5.12E-01 |
| NUDT17 | 200035 | 1.0341931 | 3.5685202 | 2.15E-03 | 1.35E-02 |
| FBXO11 | 80204 | 1.0337961 | 4.1383656 | 3.49E-05 | 4.59E-04 |
| DNAJB4 | 11080 | 1.0335022 | 5.1491239 | 3.38E-05 | 4.47E-04 |
| ARHGEF28 | 64283 | 1.0329667 | 0.2105204 | 1.68E-01 | 3.42E-01 |
| ALDH3A2 | 224 | 1.0321366 | 6.417659 | 2.68E-02 | 9.38E-02 |
| PIP5KL1 | 138429 | 1.0315479 | 3.0744687 | 1.74E-01 | 3.50E-01 |
| KHDRBS3 | 10656 | 1.0313554 | 7.2111486 | 3.30E-06 | 6.05E-05 |
| C19orf38 | 255809 | 1.0301457 | 0.9529417 | 4.99E-02 | 1.46E-01 |
| CABCOCO1 | 219621 | 1.0297697 | 0.3938283 | 1.32E-01 | 2.91E-01 |
| WDR48 | 57599 | 1.0296766 | 5.9319346 | 2.43E-08 | 8.12E-07 |
| CERS2 | 29956 | 1.0285519 | 4.6479223 | 1.84E-02 | 7.12E-02 |
| LYN | 4067 | 1.0281068 | 2.4478773 | 2.74E-01 | 4.71E-01 |
| TMEM263 | 90488 | 1.0279443 | 5.972041 | 2.25E-02 | 8.28E-02 |
| FAM126A | 84668 | 1.0276831 | 4.4676292 | 7.36E-03 | 3.60E-02 |
| RMDN2 | 151393 | 1.027661 | 2.6178621 | 1.04E-01 | 2.47E-01 |
| ST8SIA6 | 338596 | 1.0265713 | 1.7026372 | 2.49E-01 | 4.43E-01 |
| ZNF480 | 147657 | 1.0258688 | 4.709236 | 1.10E-04 | 1.22E-03 |
| DCLRE1A | 9937 | 1.0253854 | 2.4789637 | 2.69E-03 | 1.63E-02 |
| HOMEZ | 57594 | 1.0245149 | 2.5808196 | 2.41E-02 | 8.68E-02 |
| LCA5 | 167691 | 1.0243436 | 4.1758628 | 4.73E-04 | 4.04E-03 |
| NEMP1 | 23306 | 1.0239016 | 1.4831557 | 2.52E-02 | 9.00E-02 |
| QTRT2 | 79691 | 1.0238742 | 3.5131608 | 3.24E-03 | 1.88E-02 |
| CPAMD8 | 27151 | 1.0234403 | 1.1696584 | 1.94E-02 | 7.41E-02 |
| ARHGEF3 | 50650 | 1.0229369 | 4.7431124 | 2.05E-04 | 2.03E-03 |
| PHEX | 5251 | 1.0209353 | 1.214519 | 6.54E-02 | 1.77E-01 |
| FEN1 | 2237 | 1.020154 | 5.4793438 | 4.76E-08 | 1.49E-06 |
| PKNOX2 | 63876 | 1.0198853 | 2.7256604 | 4.54E-04 | 3.91E-03 |
| TGFA | 7039 | 1.0198792 | 1.2451699 | 1.42E-01 | 3.04E-01 |
| ANP32E | 81611 | 1.0192533 | 4.6111958 | 1.44E-06 | 2.96E-05 |
| TNC | 3371 | 1.0179388 | 2.0164209 | 1.44E-01 | 3.07E-01 |
| SENP1 | 29843 | 1.0175677 | 3.1728498 | 4.13E-03 | 2.28E-02 |
| FILIP1L | 11259 | 1.0171598 | 2.1292443 | 2.33E-02 | 8.48E-02 |
| CFAP44 | 55779 | 1.0171283 | 1.1062856 | 4.46E-02 | 1.36E-01 |
| PALM2AKAP2 | 445815 | 1.0167459 | 6.0316004 | 3.33E-07 | 8.12E-06 |
| CCDC117 | 150275 | 1.0166969 | 4.585757 | 3.61E-07 | 8.74E-06 |
| NEO1 | 4756 | 1.0158629 | 4.330336 | 1.35E-04 | 1.44E-03 |
| PALB2 | 79728 | 1.0156814 | 3.7124 | 3.18E-06 | 5.85E-05 |
| GOLGA6L10 | 647042 | 1.0154916 | -0.272238 | 1.46E-01 | 3.09E-01 |
| SYBU | 55638 | 1.0137921 | 6.462765 | 2.95E-05 | 4.02E-04 |
| CBWD1 | 55871 | 1.0136675 | 6.0292204 | 1.51E-05 | 2.27E-04 |
| CTR9 | 9646 | 1.0136094 | 5.9432483 | 3.91E-07 | 9.33E-06 |
| SP3 | 6670 | 1.0121611 | 5.0590842 | 1.51E-05 | 2.27E-04 |
| NUDT11 | 55190 | 1.0120921 | 6.7152293 | 1.63E-03 | 1.09E-02 |
| PCBP4 | 57060 | 1.0101956 | 6.5698238 | 1.58E-03 | 1.06E-02 |
| CENPA | 1058 | 1.010059 | 0.5970561 | 2.12E-01 | 3.99E-01 |
| DEK | 7913 | 1.0087255 | 6.1264931 | 6.23E-07 | 1.41E-05 |
| MOB4 | 25843 | 1.0084339 | 6.4987176 | 1.22E-04 | 1.32E-03 |
| TAFA2 | 338811 | 1.0083181 | 4.390308 | 6.38E-02 | 1.74E-01 |
| STK32A | 202374 | 1.0081305 | 2.8820587 | 3.76E-02 | 1.20E-01 |
| FAM214A | 56204 | 1.00735 | 3.9002733 | 1.50E-03 | 1.02E-02 |
| LRIG1 | 26018 | 1.0072715 | 2.3027217 | 1.54E-01 | 3.21E-01 |
| PPP2R1B | 5519 | 1.0049202 | 4.5790902 | 3.78E-02 | 1.21E-01 |
| HS3ST3A1 | 9955 | 1.0046689 | 1.3588375 | 4.79E-01 | 6.68E-01 |
| GABARAPL1 | 23710 | 1.0043485 | 7.3652445 | 4.30E-07 | 1.02E-05 |
| GTF2A1 | 2957 | 1.0026791 | 4.4857058 | 3.05E-06 | 5.64E-05 |
| USP1 | 7398 | 1.0023626 | 4.8907187 | 2.37E-05 | 3.33E-04 |
| DUSP22 | 56940 | 1.0022933 | 4.1252658 | 1.29E-05 | 1.98E-04 |
| GARNL3 | 84253 | 1.0021252 | 3.4811062 | 3.76E-03 | 2.12E-02 |
| DLG2 | 1740 | 1.000755 | 6.6702969 | 1.65E-01 | 3.37E-01 |
| NUB1 | 51667 | 1.0005418 | 5.3060675 | 1.83E-07 | 4.83E-06 |
| NFKB2 | 4791 | 1.0000162 | 1.4993351 | 3.44E-01 | 5.45E-01 |
| ZFP69 | 339559 | 0.9995826 | 3.7884869 | 8.17E-04 | 6.30E-03 |
| CEP85 | 64793 | 0.9995293 | 3.4730801 | 2.41E-05 | 3.37E-04 |
| ZNF621 | 285268 | 0.9994842 | 3.2967179 | 4.02E-04 | 3.55E-03 |
| RANBP9 | 10048 | 0.9993723 | 3.9948285 | 1.08E-06 | 2.32E-05 |
| FAM135A | 57579 | 0.9991523 | 4.2434508 | 1.64E-05 | 2.44E-04 |
| MTIF2 | 4528 | 0.9976672 | 4.9396474 | 1.14E-06 | 2.43E-05 |
| RRM2B | 50484 | 0.9969276 | 5.1166322 | 1.49E-01 | 3.14E-01 |
| PRDM4 | 11108 | 0.9963032 | 4.6857965 | 6.01E-07 | 1.36E-05 |
| C2CD5 | 9847 | 0.9957821 | 4.5353517 | 2.40E-06 | 4.58E-05 |
| MRC2 | 9902 | 0.9952558 | 2.4900627 | 3.09E-01 | 5.08E-01 |
| NFIX | 4784 | 0.9946088 | 3.9644547 | 1.58E-01 | 3.26E-01 |
| ZNF703 | 80139 | 0.993756 | 0.1293765 | 7.30E-02 | 1.92E-01 |
| PAQR3 | 152559 | 0.9924796 | 5.5990902 | 3.32E-08 | 1.08E-06 |
| GLUD2 | 2747 | 0.9920801 | 1.8061525 | 8.63E-03 | 4.06E-02 |
| LRRC63 | 220416 | 0.9908833 | 0.245273 | 4.28E-02 | 1.32E-01 |
| AFF2 | 2334 | 0.9905358 | 3.3235531 | 4.51E-02 | 1.37E-01 |
| WDR6 | 11180 | 0.9898635 | 6.1141975 | 2.20E-05 | 3.14E-04 |
| RCHY1 | 25898 | 0.9891162 | 6.8053467 | 3.23E-05 | 4.31E-04 |
| PROSER1 | 80209 | 0.9887768 | 3.9082206 | 5.51E-03 | 2.90E-02 |
| EFCAB2 | 84288 | 0.9885909 | 4.0175102 | 5.03E-06 | 8.71E-05 |
| STAMBPL1 | 57559 | 0.988345 | 4.3098679 | 1.16E-02 | 5.06E-02 |
| HINT3 | 135114 | 0.9874644 | 6.1868229 | 1.83E-04 | 1.84E-03 |
| KIF18A | 81930 | 0.9874113 | 0.766082 | 4.16E-02 | 1.29E-01 |
| GJD2 | 57369 | 0.9872376 | 1.3286591 | 2.70E-01 | 4.66E-01 |
| AAGAB | 79719 | 0.9870607 | 6.4437882 | 2.47E-04 | 2.35E-03 |
| DEPDC7 | 91614 | 0.987019 | 1.9582901 | 1.02E-02 | 4.57E-02 |
| TMPO | 7112 | 0.9861275 | 4.9530204 | 1.09E-04 | 1.22E-03 |
| RGS11 | 8786 | 0.9858556 | 0.099874 | 6.38E-02 | 1.74E-01 |
| RWDD2A | 112611 | 0.9852334 | 4.5681625 | 7.42E-06 | 1.23E-04 |
| STXBP3 | 6814 | 0.9851743 | 5.2388609 | 1.74E-03 | 1.15E-02 |
| C10orf67 | 256815 | 0.9839527 | 0.5078002 | 6.15E-02 | 1.70E-01 |
| SOX2 | 6657 | 0.9829179 | 3.7124414 | 1.70E-02 | 6.69E-02 |
| ID1 | 3397 | 0.9827218 | 2.1430521 | 2.32E-02 | 8.45E-02 |
| IRAK1 | 3654 | 0.9822226 | 3.9744078 | 4.13E-02 | 1.29E-01 |
| ZNF597 | 146434 | 0.9816737 | 2.1308819 | 3.70E-03 | 2.10E-02 |
| SLC35A5 | 55032 | 0.981335 | 5.8725211 | 8.59E-07 | 1.89E-05 |
| DNAAF1 | 123872 | 0.9806815 | 0.0563657 | 1.41E-01 | 3.03E-01 |
| OARD1 | 221443 | 0.9795718 | 5.9054999 | 1.05E-04 | 1.18E-03 |
| C1orf54 | 79630 | 0.9782435 | 3.4728491 | 8.66E-04 | 6.58E-03 |
| KLHL2 | 11275 | 0.9777788 | 6.0927771 | 1.04E-03 | 7.61E-03 |
| CEP170 | 9859 | 0.9776214 | 7.3869296 | 6.93E-04 | 5.53E-03 |
| FAM24B | 196792 | 0.9760918 | 1.4397721 | 3.45E-03 | 1.98E-02 |
| EPHB3 | 2049 | 0.9748143 | 2.9773128 | 4.14E-03 | 2.29E-02 |
| PLPP7 | 84814 | 0.9747867 | 2.8096129 | 1.08E-01 | 2.53E-01 |
| BRPF3 | 27154 | 0.9741215 | 3.3344975 | 3.21E-04 | 2.92E-03 |
| NUDT10 | 170685 | 0.9736715 | 5.5960006 | 7.56E-03 | 3.68E-02 |
| RNF111 | 54778 | 0.9736659 | 4.8169927 | 2.31E-06 | 4.44E-05 |
| FCGRT | 2217 | 0.9735639 | 4.1312307 | 7.54E-02 | 1.96E-01 |
| NNMT | 4837 | 0.9734986 | 5.330594 | 1.61E-01 | 3.31E-01 |
| TMX4 | 56255 | 0.9733434 | 7.1541627 | 8.01E-05 | 9.34E-04 |
| ZBTB8B | 728116 | 0.9723874 | 2.7301885 | 1.58E-01 | 3.25E-01 |
| ENTPD5 | 957 | 0.9714653 | 2.8321153 | 1.02E-03 | 7.48E-03 |
| PAFAH2 | 5051 | 0.9703347 | 2.9774297 | 1.51E-02 | 6.15E-02 |
| PDGFRB | 5159 | 0.9699 | 1.2758115 | 5.14E-01 | 6.95E-01 |
| GPNMB | 10457 | 0.9697283 | 4.0828274 | 1.30E-03 | 9.12E-03 |
| KIF13A | 63971 | 0.9686687 | 3.1341053 | 2.21E-02 | 8.15E-02 |
| CCDC62 | 84660 | 0.9684955 | 0.8376555 | 4.03E-02 | 1.26E-01 |
| MARCHF1 | 55016 | 0.9684334 | 5.9290946 | 2.06E-01 | 3.91E-01 |
| OSBPL9 | 114883 | 0.968359 | 6.2518589 | 4.56E-03 | 2.48E-02 |
| STAG1 | 10274 | 0.9674779 | 3.7877668 | 8.23E-04 | 6.33E-03 |
| NBN | 4683 | 0.9670358 | 4.9688869 | 8.33E-07 | 1.84E-05 |
| ASTE1 | 28990 | 0.9664791 | 3.6405504 | 7.29E-05 | 8.70E-04 |
| LRP2 | 4036 | 0.9664703 | 4.782361 | 2.07E-02 | 7.77E-02 |
| AKIRIN1 | 79647 | 0.9659122 | 8.1941971 | 2.69E-06 | 5.04E-05 |
| IMPACT | 55364 | 0.9655961 | 4.9552256 | 1.15E-06 | 2.45E-05 |
| NMU | 10874 | 0.9651081 | 3.7926434 | 2.28E-01 | 4.18E-01 |
| HS1BP3 | 64342 | 0.9649779 | 3.423879 | 2.59E-03 | 1.58E-02 |
| CYGB | 114757 | 0.9638353 | 2.4831561 | 2.70E-03 | 1.63E-02 |
| CABLES1 | 91768 | 0.9636132 | 0.1763633 | 2.15E-01 | 4.03E-01 |
| ZNF670 | 93474 | 0.9614444 | 3.4210846 | 3.45E-04 | 3.11E-03 |
| AIDA | 64853 | 0.9612177 | 3.0074385 | 2.18E-03 | 1.37E-02 |
| RNF19B | 127544 | 0.9606889 | 5.1418113 | 1.08E-03 | 7.85E-03 |
| ATMIN | 23300 | 0.9591276 | 6.5873526 | 1.75E-05 | 2.58E-04 |
| WDFY1 | 57590 | 0.9588898 | 4.6315756 | 6.03E-04 | 4.91E-03 |
| PHYHIPL | 84457 | 0.9587066 | 7.2340571 | 2.17E-01 | 4.06E-01 |
| SPIN2A | 54466 | 0.9584527 | 2.8913826 | 2.47E-01 | 4.40E-01 |
| MMP2 | 4313 | 0.9578344 | 4.4127244 | 1.53E-01 | 3.19E-01 |
| GPRASP2 | 100528062 | 0.9575984 | 5.798391 | 1.52E-04 | 1.58E-03 |
| KRT222 | 125113 | 0.957575 | 3.0361274 | 3.74E-01 | 5.74E-01 |
| BEST4 | 266675 | 0.9575025 | 0.4220825 | 4.32E-02 | 1.33E-01 |
| PDGFD | 80310 | 0.9573003 | 3.4360388 | 1.78E-01 | 3.55E-01 |
| PRTFDC1 | 56952 | 0.9572179 | 3.7444959 | 8.05E-02 | 2.05E-01 |
| ANP32B | 10541 | 0.956471 | 4.975937 | 2.74E-06 | 5.11E-05 |
| SMARCAL1 | 50485 | 0.9561097 | 4.0286956 | 4.30E-03 | 2.36E-02 |
| NRM | 11270 | 0.9556714 | 3.7060389 | 2.54E-04 | 2.41E-03 |
| CCAR1 | 55749 | 0.9551171 | 6.6610185 | 1.49E-06 | 3.03E-05 |
| PLPP1 | 8611 | 0.9543499 | 4.803248 | 2.96E-06 | 5.51E-05 |
| SGO2 | 151246 | 0.954261 | 2.633123 | 2.45E-02 | 8.81E-02 |
| IQCB1 | 9657 | 0.9530513 | 4.3938288 | 7.65E-06 | 1.27E-04 |
| PPIL4 | 85313 | 0.9529199 | 6.1443137 | 1.21E-06 | 2.56E-05 |
| FDFT1 | 2222 | 0.9526836 | 9.8611556 | 3.84E-07 | 9.23E-06 |
| TOPBP1 | 11073 | 0.9526762 | 4.4941922 | 1.18E-02 | 5.12E-02 |
| CIT | 11113 | 0.9525111 | 2.4367019 | 2.73E-02 | 9.52E-02 |
| TSPAN33 | 340348 | 0.951161 | 4.496456 | 3.15E-03 | 1.84E-02 |
| TUBE1 | 51175 | 0.9502539 | 4.5304669 | 9.04E-04 | 6.82E-03 |
| TIGD2 | 166815 | 0.9492732 | 2.1248102 | 1.85E-02 | 7.14E-02 |
| SIRT1 | 23411 | 0.9487878 | 4.2988449 | 9.45E-05 | 1.08E-03 |
| TBC1D23 | 55773 | 0.9481782 | 5.5763628 | 1.24E-04 | 1.34E-03 |
| DCAF17 | 80067 | 0.9481147 | 3.7164215 | 3.20E-05 | 4.29E-04 |
| DELE1 | 9812 | 0.9475224 | 3.6129579 | 5.08E-04 | 4.27E-03 |
| DDX52 | 11056 | 0.9474709 | 5.4361617 | 1.25E-03 | 8.85E-03 |
| ZNF512 | 84450 | 0.9471579 | 5.7301193 | 1.52E-05 | 2.28E-04 |
| RB1CC1 | 9821 | 0.946755 | 6.5811645 | 1.60E-06 | 3.24E-05 |
| SLC20A1 | 6574 | 0.9464509 | 5.7276666 | 2.75E-04 | 2.57E-03 |
| BPGM | 669 | 0.9464328 | 6.4160527 | 1.39E-05 | 2.11E-04 |
| PPP3CC | 5533 | 0.945369 | 5.5206079 | 5.29E-07 | 1.21E-05 |
| FMR1 | 2332 | 0.9450698 | 5.2638204 | 3.28E-07 | 8.08E-06 |
| CHD8 | 57680 | 0.9440234 | 4.7835699 | 2.14E-06 | 4.17E-05 |
| TONSL | 4796 | 0.9438975 | 0.5819135 | 1.25E-02 | 5.34E-02 |
| ABI1 | 10006 | 0.9435071 | 6.2694103 | 7.33E-07 | 1.64E-05 |
| NAGS | 162417 | 0.943174 | 1.6477866 | 1.47E-02 | 6.00E-02 |
| RAB8B | 51762 | 0.9425318 | 5.3711039 | 5.42E-06 | 9.32E-05 |
| COL11A1 | 1301 | 0.941969 | 5.1198424 | 2.46E-01 | 4.39E-01 |
| WASHC2C | 253725 | 0.9418157 | 4.5628372 | 6.61E-07 | 1.49E-05 |
| PLAG1 | 5324 | 0.9416038 | 0.5885501 | 1.40E-01 | 3.02E-01 |
| JPT2 | 90861 | 0.9415592 | 4.6586257 | 1.17E-03 | 8.39E-03 |
| SYTL3 | 94120 | 0.9409481 | 0.9424673 | 3.14E-02 | 1.05E-01 |
| SEMA4A | 64218 | 0.9405653 | 4.0510693 | 1.95E-01 | 3.77E-01 |
| NUDT12 | 83594 | 0.9401017 | 3.4778255 | 2.56E-04 | 2.43E-03 |
| MYBBP1A | 10514 | 0.9394102 | 2.6389943 | 3.43E-02 | 1.12E-01 |
| TBXAS1 | 6916 | 0.9388892 | 0.1731319 | 8.10E-02 | 2.06E-01 |
| DUSP1 | 1843 | 0.9386101 | 7.2897736 | 3.99E-02 | 1.25E-01 |
| KCNA3 | 3738 | 0.9383946 | 2.7488272 | 1.60E-01 | 3.30E-01 |
| MOB3C | 148932 | 0.9382066 | 1.561238 | 1.16E-01 | 2.66E-01 |
| RP2 | 6102 | 0.9380118 | 3.5028789 | 3.86E-02 | 1.22E-01 |
| MUC12 | 10071 | 0.9379791 | 0.5566517 | 8.40E-02 | 2.11E-01 |
| FBXL3 | 26224 | 0.937928 | 4.6698675 | 8.65E-04 | 6.57E-03 |
| NOL3 | 8996 | 0.9377653 | 4.5149147 | 2.44E-02 | 8.77E-02 |
| ABTB2 | 25841 | 0.9372053 | 1.0782155 | 1.24E-01 | 2.78E-01 |
| RFESD | 317671 | 0.9368135 | 0.4689711 | 9.95E-02 | 2.38E-01 |
| GTF3C3 | 9330 | 0.9362064 | 4.3353292 | 8.87E-05 | 1.02E-03 |
| ZSCAN16 | 80345 | 0.9360296 | 4.1742719 | 2.34E-03 | 1.45E-02 |
| PGBD1 | 84547 | 0.9350833 | 4.6906702 | 1.09E-05 | 1.73E-04 |
| C17orf100 | 388327 | 0.935019 | 4.6053587 | 1.83E-03 | 1.19E-02 |
| BRCC3 | 79184 | 0.9347072 | 5.8374192 | 1.10E-06 | 2.36E-05 |
| SRBD1 | 55133 | 0.9342795 | 3.6216354 | 1.20E-03 | 8.57E-03 |
| MPPE1 | 65258 | 0.933665 | 3.9981758 | 5.27E-05 | 6.62E-04 |
| TSKU | 25987 | 0.9336241 | 2.0436958 | 1.33E-01 | 2.92E-01 |
| PPP1R3B | 79660 | 0.9335683 | 4.4730049 | 2.36E-01 | 4.27E-01 |
| HLTF | 6596 | 0.9333886 | 6.3893574 | 1.28E-05 | 1.98E-04 |
| DLX2 | 1746 | 0.9333665 | 2.7393421 | 2.90E-01 | 4.89E-01 |
| DDX60L | 91351 | 0.9325645 | 0.6210472 | 3.33E-01 | 5.34E-01 |
| LANCL2 | 55915 | 0.932183 | 5.5576598 | 7.81E-04 | 6.07E-03 |
| LHX1 | 3975 | 0.9311917 | 3.6877927 | 2.88E-01 | 4.87E-01 |
| TYW3 | 127253 | 0.9304607 | 3.02814 | 9.82E-03 | 4.45E-02 |
| ACTA1 | 58 | 0.9298138 | 1.2225234 | 3.79E-01 | 5.78E-01 |
| FGD6 | 55785 | 0.9297035 | 2.679845 | 2.81E-03 | 1.69E-02 |
| NEK11 | 79858 | 0.929206 | 3.4296239 | 4.16E-04 | 3.66E-03 |
| FGF14 | 2259 | 0.928788 | 6.0778469 | 1.83E-01 | 3.61E-01 |
| DNAJC6 | 9829 | 0.9280042 | 6.606747 | 7.78E-04 | 6.05E-03 |
| HSD17B6 | 8630 | 0.9276505 | 1.1307861 | 1.24E-01 | 2.78E-01 |
| H2BC8 | 8339 | 0.9268012 | 0.3916798 | 5.20E-02 | 1.51E-01 |
| ZNF215 | 7762 | 0.9267397 | 1.8049078 | 1.64E-01 | 3.36E-01 |
| HLA-DPA1 | 3113 | 0.9266018 | 4.5695303 | 1.48E-01 | 3.12E-01 |
| ZNF606 | 80095 | 0.9264738 | 4.2301893 | 6.01E-05 | 7.42E-04 |
| CCZ1 | 51622 | 0.9247238 | 4.0939762 | 1.51E-05 | 2.27E-04 |
| NQO1 | 1728 | 0.9238437 | 8.2397492 | 8.70E-02 | 2.16E-01 |
| CLVS2 | 134829 | 0.923307 | 3.6491595 | 1.58E-01 | 3.25E-01 |
| FAM50B | 26240 | 0.9232587 | 3.6469393 | 4.78E-02 | 1.42E-01 |
| PTGER3 | 5733 | 0.9231198 | 3.1709419 | 1.42E-01 | 3.05E-01 |
| LEPROT | 54741 | 0.9219383 | 5.9400416 | 3.02E-02 | 1.02E-01 |
| DDX59 | 83479 | 0.9217473 | 4.2596018 | 5.46E-05 | 6.83E-04 |
| MKRN3 | 7681 | 0.9215232 | 2.338564 | 1.71E-03 | 1.14E-02 |
| SCML2 | 10389 | 0.9209505 | 2.0341596 | 9.76E-02 | 2.35E-01 |
| STOX1 | 219736 | 0.9202713 | 1.3296338 | 7.61E-02 | 1.98E-01 |
| PRKAR2B | 5577 | 0.9202323 | 7.9907904 | 1.28E-01 | 2.84E-01 |
| BRD2 | 6046 | 0.9197493 | 6.4388188 | 8.01E-05 | 9.34E-04 |
| VEGFA | 7422 | 0.9192441 | 2.8484904 | 1.75E-01 | 3.51E-01 |
| TTC23 | 64927 | 0.9182961 | 2.4429769 | 3.89E-02 | 1.23E-01 |
| HSD17B7 | 51478 | 0.9171767 | 6.568773 | 1.43E-06 | 2.94E-05 |
| QPRT | 23475 | 0.9170984 | 5.4810614 | 6.03E-03 | 3.11E-02 |
| RBM22 | 55696 | 0.9162416 | 5.9957568 | 3.86E-07 | 9.26E-06 |
| FSBP | 100861412 | 0.9153728 | 0.960773 | 9.95E-02 | 2.38E-01 |
| ZFP36 | 7538 | 0.9147475 | 1.9222982 | 3.22E-01 | 5.23E-01 |
| TYMS | 7298 | 0.9146808 | 1.4270437 | 9.63E-02 | 2.32E-01 |
| NOLC1 | 9221 | 0.9146071 | 7.0186603 | 1.22E-06 | 2.58E-05 |
| UQCC3 | 790955 | 0.9140819 | 1.2404266 | 5.01E-02 | 1.47E-01 |
| UHRF2 | 115426 | 0.9136836 | 4.0187989 | 3.07E-05 | 4.15E-04 |
| ZNF197 | 110354863 | 0.9132188 | 4.4306782 | 1.00E-03 | 7.40E-03 |
| SHISAL2B | 100132916 | 0.9130554 | 0.5662803 | 3.49E-01 | 5.49E-01 |
| RAB3GAP1 | 22930 | 0.9130137 | 5.3570128 | 6.52E-05 | 7.91E-04 |
| HSD17B14 | 51171 | 0.9129053 | 5.2369063 | 2.94E-05 | 4.00E-04 |
| ZNF391 | 346157 | 0.9124848 | 2.9560175 | 6.50E-04 | 5.22E-03 |
| ADAM21 | 8747 | 0.9122488 | 0.413027 | 4.64E-02 | 1.40E-01 |
| CABYR | 26256 | 0.9120373 | 4.3255889 | 9.22E-02 | 2.25E-01 |
| PENK | 5179 | 0.9120297 | 2.550411 | 2.81E-01 | 4.79E-01 |
| HSPA6 | 3310 | 0.9109786 | 1.8779607 | 8.56E-02 | 2.14E-01 |
| ZMAT1 | 84460 | 0.9108733 | 2.5210052 | 9.97E-02 | 2.39E-01 |
| C5 | 727 | 0.9077499 | 1.0216122 | 9.62E-02 | 2.32E-01 |
| SAMD4A | 23034 | 0.9073374 | 1.8559559 | 7.08E-02 | 1.88E-01 |
| CD9 | 928 | 0.9067215 | 4.0659499 | 1.57E-02 | 6.32E-02 |
| BAZ1B | 9031 | 0.9065156 | 5.9487405 | 1.58E-06 | 3.20E-05 |
| RAB4A | 5867 | 0.9061206 | 6.9459739 | 1.25E-06 | 2.63E-05 |
| STXBP5L | 9515 | 0.9060294 | 3.7092855 | 5.04E-03 | 2.70E-02 |
| MTURN | 222166 | 0.9059557 | 6.2300988 | 9.09E-03 | 4.21E-02 |
| ZRANB1 | 54764 | 0.9059 | 4.2096578 | 8.62E-04 | 6.56E-03 |
| LIN9 | 286826 | 0.9057065 | 1.8518268 | 1.61E-02 | 6.42E-02 |
| TMEM143 | 55260 | 0.9051832 | 3.5306915 | 4.27E-04 | 3.73E-03 |
| ZSCAN9 | 7746 | 0.9048868 | 4.9898303 | 2.24E-05 | 3.17E-04 |
| MTHFD2 | 10797 | 0.9041292 | 4.8558061 | 7.03E-03 | 3.47E-02 |
| ADD3 | 120 | 0.9038464 | 5.2755516 | 4.12E-02 | 1.29E-01 |
| SMC6 | 79677 | 0.9030312 | 4.8687353 | 1.84E-04 | 1.85E-03 |
| HPS5 | 11234 | 0.9027686 | 3.0466185 | 1.14E-01 | 2.61E-01 |
| TMEM130 | 222865 | 0.9024172 | 7.2428376 | 2.04E-02 | 7.68E-02 |
| VCL | 7414 | 0.900935 | 5.6267636 | 1.83E-01 | 3.62E-01 |
| C9orf72 | 203228 | 0.9007699 | 5.8898102 | 1.45E-02 | 5.96E-02 |
| USP6NL | 9712 | 0.900765 | 3.8661665 | 2.45E-05 | 3.41E-04 |
| SLC10A7 | 84068 | 0.900533 | 2.1542149 | 8.13E-03 | 3.88E-02 |
| ZNF385C | 201181 | 0.9004912 | 0.3542254 | 2.59E-01 | 4.54E-01 |
| SDE2 | 163859 | 0.9002507 | 4.3379115 | 2.30E-04 | 2.22E-03 |
| BIRC2 | 329 | 0.9002318 | 5.882342 | 7.83E-07 | 1.75E-05 |
| GDAP1 | 54332 | 0.8995148 | 6.7791632 | 3.05E-03 | 1.80E-02 |
| GINS2 | 51659 | 0.8993326 | 3.2152516 | 1.43E-02 | 5.88E-02 |
| TMEM169 | 92691 | 0.8975389 | 5.0378962 | 1.23E-01 | 2.76E-01 |
| EXOC5 | 10640 | 0.8972788 | 6.5667761 | 1.76E-06 | 3.51E-05 |
| BTG2 | 7832 | 0.8972101 | 4.9624976 | 7.79E-02 | 2.01E-01 |
| TOPORS | 10210 | 0.8963873 | 5.5130052 | 1.19E-05 | 1.87E-04 |
| ZNF610 | 162963 | 0.8961234 | 3.9400525 | 1.14E-02 | 4.97E-02 |
| PREPL | 9581 | 0.8954631 | 7.2477638 | 3.32E-05 | 4.42E-04 |
| GRID2 | 2895 | 0.8951843 | 2.4271478 | 2.26E-03 | 1.41E-02 |
| FARS2 | 10667 | 0.8949239 | 3.9103868 | 5.76E-05 | 7.17E-04 |
| SLC25A13 | 10165 | 0.8946429 | 4.9583698 | 7.96E-03 | 3.82E-02 |
| RNF20 | 56254 | 0.8945306 | 6.145251 | 4.21E-07 | 9.98E-06 |
| FMO5 | 2330 | 0.893497 | 0.1800086 | 3.28E-01 | 5.28E-01 |
| LRRFIP2 | 9209 | 0.8927261 | 5.3893322 | 2.52E-03 | 1.54E-02 |
| ALKBH8 | 91801 | 0.8926151 | 3.703522 | 9.75E-05 | 1.11E-03 |
| SMIM10L1 | 100129361 | 0.892279 | 5.5318858 | 1.53E-05 | 2.30E-04 |
| TP53INP1 | 94241 | 0.8922662 | 3.0738294 | 1.55E-01 | 3.22E-01 |
| SPATA6 | 54558 | 0.8917482 | 2.956928 | 9.32E-03 | 4.28E-02 |
| INTS13 | 55726 | 0.8904935 | 4.4973392 | 8.31E-04 | 6.37E-03 |
| TTLL5 | 23093 | 0.8904839 | 3.5317962 | 1.30E-03 | 9.13E-03 |
| EIF4G2 | 1982 | 0.8901223 | 9.5027074 | 1.66E-04 | 1.70E-03 |
| TAS2R20 | 259295 | 0.8899494 | 1.7460052 | 8.63E-03 | 4.06E-02 |
| CDKN2AIP | 55602 | 0.8897831 | 4.5981766 | 5.39E-04 | 4.48E-03 |
| RAB27B | 5874 | 0.8897548 | 3.2721014 | 2.51E-04 | 2.38E-03 |
| SPP1 | 6696 | 0.8892801 | 7.4380539 | 4.42E-02 | 1.35E-01 |
| PIP5K1A | 8394 | 0.8892055 | 4.1928881 | 8.65E-03 | 4.06E-02 |
| DAAM1 | 23002 | 0.8887721 | 7.8045407 | 7.46E-02 | 1.95E-01 |
| NCOA2 | 10499 | 0.8884708 | 4.2486936 | 2.21E-04 | 2.16E-03 |
| HSPA1L | 3305 | 0.8882483 | 2.2877334 | 7.42E-03 | 3.62E-02 |
| NOL4 | 8715 | 0.8880508 | 5.2352625 | 4.18E-07 | 9.94E-06 |
| SUPT6H | 6830 | 0.8862584 | 4.1956594 | 6.51E-03 | 3.28E-02 |
| CCNJ | 54619 | 0.8860716 | 3.7864437 | 1.24E-03 | 8.74E-03 |
| STAM | 8027 | 0.8860684 | 5.7938427 | 1.66E-05 | 2.46E-04 |
| COPB1 | 1315 | 0.8849975 | 6.9543576 | 2.03E-03 | 1.30E-02 |
| ZBTB46 | 140685 | 0.8849343 | 1.7705988 | 4.91E-02 | 1.45E-01 |
| WDR31 | 114987 | 0.8848137 | 2.6546432 | 8.83E-02 | 2.18E-01 |
| AURKB | 9212 | 0.8842342 | 1.3497165 | 3.69E-01 | 5.68E-01 |
| TTC17 | 55761 | 0.8826993 | 5.7595541 | 4.25E-05 | 5.44E-04 |
| RAB33B | 83452 | 0.8825176 | 3.9924549 | 7.49E-05 | 8.91E-04 |
| PJA2 | 9867 | 0.8824383 | 7.8624982 | 2.21E-06 | 4.29E-05 |
| LAMA1 | 284217 | 0.8823941 | 0.8677703 | 1.07E-01 | 2.51E-01 |
| BRINP2 | 57795 | 0.8822982 | 4.211018 | 1.94E-01 | 3.76E-01 |
| CDH12 | 1010 | 0.8816607 | 2.9991424 | 8.91E-02 | 2.20E-01 |
| MMRN1 | 22915 | 0.8815792 | 0.8447644 | 3.03E-02 | 1.03E-01 |
| ALAD | 210 | 0.8806796 | 4.590191 | 2.76E-04 | 2.58E-03 |
| CACNA2D1 | 781 | 0.8804346 | 4.9956075 | 3.96E-02 | 1.25E-01 |
| RAD51AP1 | 10635 | 0.8802774 | 0.8677205 | 8.11E-02 | 2.06E-01 |
| BSPRY | 54836 | 0.8793215 | -0.211226 | 5.03E-02 | 1.47E-01 |
| UPK2 | 7379 | 0.8785762 | 2.6599538 | 4.93E-04 | 4.18E-03 |
| GALNT12 | 79695 | 0.8775706 | 0.0524722 | 1.72E-01 | 3.46E-01 |
| MFSD6 | 54842 | 0.8772972 | 3.8751758 | 6.13E-03 | 3.15E-02 |
| ORC5 | 5001 | 0.8770428 | 4.6526529 | 1.14E-05 | 1.79E-04 |
| MTHFS | 10588 | 0.876375 | 0.9753929 | 1.12E-02 | 4.94E-02 |
| PCSK5 | 5125 | 0.8761704 | 3.4790834 | 2.17E-01 | 4.05E-01 |
| IFT80 | 57560 | 0.8754723 | 2.428734 | 7.10E-03 | 3.50E-02 |
| BARX1 | 56033 | 0.8751178 | 1.9396369 | 6.31E-02 | 1.73E-01 |
| MCMBP | 79892 | 0.8748862 | 5.7346364 | 3.67E-05 | 4.79E-04 |
| ACTL6A | 86 | 0.874867 | 5.2767222 | 6.17E-03 | 3.16E-02 |
| TSFM | 10102 | 0.8746352 | 0.3093567 | 1.02E-01 | 2.43E-01 |
| SENP7 | 57337 | 0.8745996 | 4.5932267 | 7.06E-03 | 3.49E-02 |
| OLFML2B | 25903 | 0.8743456 | 2.3781065 | 5.94E-02 | 1.66E-01 |
| SUPT16H | 11198 | 0.8740872 | 7.3773476 | 2.05E-06 | 4.03E-05 |
| ASS1 | 445 | 0.871808 | 5.8154365 | 1.17E-04 | 1.28E-03 |
| ARHGEF40 | 55701 | 0.8717304 | 3.1347377 | 6.14E-03 | 3.15E-02 |
| TUBGCP3 | 10426 | 0.8714246 | 3.6952739 | 1.23E-04 | 1.33E-03 |
| SLC25A46 | 91137 | 0.8713252 | 5.957204 | 3.44E-06 | 6.25E-05 |
| CLTRN | 57393 | 0.8711213 | 1.5360196 | 4.30E-02 | 1.32E-01 |
| KIAA1958 | 158405 | 0.8710115 | 3.5645109 | 1.05E-03 | 7.71E-03 |
| THOC3 | 84321 | 0.8707203 | 5.3754599 | 1.20E-06 | 2.55E-05 |
| PARG | 8505 | 0.8706269 | 4.4939042 | 2.86E-04 | 2.66E-03 |
| FAM184A | 79632 | 0.8705598 | 4.5464174 | 8.39E-04 | 6.42E-03 |
| MSMO1 | 6307 | 0.8705124 | 9.620513 | 6.08E-05 | 7.49E-04 |
| LCA5L | 150082 | 0.8703418 | 0.6500519 | 1.69E-01 | 3.42E-01 |
| ERCC5 | 100533467 | 0.8702526 | 1.6207736 | 5.82E-02 | 1.64E-01 |
| TEDC1 | 283643 | 0.8700528 | 2.8328793 | 1.51E-03 | 1.02E-02 |
| TRUB1 | 142940 | 0.8695841 | 5.4845049 | 9.75E-06 | 1.57E-04 |
| KCTD3 | 51133 | 0.8691567 | 5.5902958 | 6.55E-07 | 1.48E-05 |
| RGS3 | 5998 | 0.8689441 | 5.6802228 | 6.37E-05 | 7.79E-04 |
| TDRKH | 11022 | 0.8685766 | 4.3466382 | 8.75E-04 | 6.63E-03 |
| BMS1 | 9790 | 0.868061 | 5.3867679 | 5.71E-03 | 2.98E-02 |
| H4C8 | 8365 | 0.8679622 | 2.8607526 | 1.09E-01 | 2.54E-01 |
| ZMYM1 | 79830 | 0.8677811 | 3.7743615 | 2.71E-03 | 1.64E-02 |
| PCLAF | 9768 | 0.8672076 | 1.6249821 | 1.66E-01 | 3.37E-01 |
| TPD52 | 7163 | 0.8665745 | 5.2700997 | 3.64E-03 | 2.07E-02 |
| XPC | 7508 | 0.8664849 | 4.1772787 | 1.41E-02 | 5.82E-02 |
| COL7A1 | 1294 | 0.8663409 | 0.6134773 | 1.56E-01 | 3.23E-01 |
| ADSS1 | 122622 | 0.8662043 | 2.3988597 | 2.02E-02 | 7.63E-02 |
| CA2 | 760 | 0.8653339 | 5.1946284 | 3.09E-02 | 1.04E-01 |
| VRK2 | 7444 | 0.863896 | 1.2291241 | 3.04E-01 | 5.03E-01 |
| CLIC1 | 1192 | 0.8630984 | 7.6040488 | 9.32E-04 | 6.98E-03 |
| LCORL | 254251 | 0.8629292 | 4.664036 | 3.05E-03 | 1.80E-02 |
| CARF | 79800 | 0.8622525 | 3.1944765 | 1.96E-03 | 1.26E-02 |
| TBK1 | 29110 | 0.8612515 | 4.9523155 | 1.14E-04 | 1.25E-03 |
| ADI1 | 55256 | 0.8611462 | 5.5576037 | 1.90E-02 | 7.31E-02 |
| OTUD3 | 23252 | 0.8601047 | 3.1234254 | 1.17E-03 | 8.41E-03 |
| EN1 | 2019 | 0.8600131 | -0.168868 | 7.97E-02 | 2.04E-01 |
| CCDC160 | 347475 | 0.859891 | 3.0420504 | 2.77E-03 | 1.67E-02 |
| DOCK5 | 80005 | 0.8595456 | 0.8788568 | 4.61E-01 | 6.52E-01 |
| ATF7IP | 55729 | 0.8593567 | 6.0686425 | 8.02E-05 | 9.34E-04 |
| TNFRSF12A | 51330 | 0.8592188 | 6.8678627 | 2.36E-01 | 4.28E-01 |
| PPP4R4 | 57718 | 0.8592166 | 1.5871701 | 1.14E-01 | 2.61E-01 |
| PGRMC1 | 10857 | 0.8588515 | 9.1956483 | 6.11E-06 | 1.04E-04 |
| BCL3 | 602 | 0.8587001 | 2.8314543 | 5.40E-02 | 1.55E-01 |
| CTPS2 | 56474 | 0.8584972 | 4.7259536 | 2.06E-03 | 1.31E-02 |
| DONSON | 29980 | 0.8584418 | 4.5479127 | 1.13E-03 | 8.14E-03 |
| CAP2 | 10486 | 0.857198 | 6.6405711 | 1.34E-06 | 2.78E-05 |
| ATG4C | 84938 | 0.8571603 | 4.2035839 | 1.29E-04 | 1.38E-03 |
| FNTA | 2339 | 0.8571344 | 5.6130615 | 2.37E-06 | 4.53E-05 |
| PHF6 | 84295 | 0.8569684 | 5.6781588 | 2.84E-04 | 2.64E-03 |
| ARL8B | 55207 | 0.8569163 | 6.7493087 | 3.23E-04 | 2.94E-03 |
| LRRN3 | 54674 | 0.856424 | 7.1299959 | 3.36E-02 | 1.11E-01 |
| SPAG8 | 26206 | 0.8562368 | 1.592805 | 5.41E-02 | 1.55E-01 |
| RRM1 | 6240 | 0.8560973 | 6.0171009 | 2.04E-05 | 2.95E-04 |
| SLC5A2 | 6524 | 0.8560394 | 0.0904017 | 1.50E-01 | 3.15E-01 |
| ZNF407 | 55628 | 0.8559589 | 1.9155262 | 2.51E-02 | 8.98E-02 |
| CBWD6 | 644019 | 0.8559508 | 3.7507451 | 5.59E-03 | 2.93E-02 |
| REV3L | 5980 | 0.8556569 | 6.2612732 | 4.83E-03 | 2.60E-02 |
| DARS1 | 1615 | 0.8548394 | 6.5464016 | 1.46E-04 | 1.53E-03 |
| WWP2 | 11060 | 0.8547844 | 4.183116 | 1.21E-03 | 8.63E-03 |
| FZD6 | 8323 | 0.8547457 | 4.1880236 | 2.20E-01 | 4.09E-01 |
| KLF11 | 8462 | 0.8539804 | 2.9145534 | 1.88E-01 | 3.67E-01 |
| TATDN3 | 128387 | 0.8532028 | 5.4935178 | 5.69E-04 | 4.70E-03 |
| ZNF184 | 7738 | 0.852812 | 3.9861577 | 2.06E-03 | 1.31E-02 |
| SYT1 | 6857 | 0.8526542 | 9.3198764 | 2.78E-01 | 4.74E-01 |
| ZNF33B | 7582 | 0.8525352 | 4.1958582 | 8.03E-03 | 3.84E-02 |
| PRIM2 | 5558 | 0.8519282 | 2.5926079 | 2.65E-03 | 1.61E-02 |
| RAB3IL1 | 5866 | 0.8516853 | 2.260425 | 1.23E-02 | 5.26E-02 |
| RASA3 | 22821 | 0.8513774 | 4.0208576 | 2.25E-02 | 8.28E-02 |
| CCDC125 | 202243 | 0.8511615 | 2.2511604 | 2.32E-01 | 4.24E-01 |
| UAP1 | 6675 | 0.8507672 | 5.6248933 | 6.36E-03 | 3.23E-02 |
| SOX30 | 11063 | 0.8505681 | 0.1468303 | 1.30E-01 | 2.88E-01 |
| CASC3 | 22794 | 0.8494026 | 5.6469364 | 3.92E-05 | 5.06E-04 |
| LMO2 | 4005 | 0.849158 | 1.9740095 | 1.82E-01 | 3.60E-01 |
| CBFB | 865 | 0.8490639 | 4.5278643 | 1.51E-03 | 1.03E-02 |
| VAMP3 | 9341 | 0.8488509 | 5.093284 | 7.01E-02 | 1.86E-01 |
| DTX2 | 113878 | 0.8481443 | 2.1859231 | 1.17E-01 | 2.67E-01 |
| TPBGL | 100507050 | 0.8474562 | 0.4116141 | 3.34E-01 | 5.34E-01 |
| OTULINL | 54491 | 0.8473446 | 2.9806389 | 1.64E-03 | 1.10E-02 |
| GEMIN5 | 25929 | 0.8472725 | 4.46869 | 2.28E-03 | 1.42E-02 |
| SFT2D2 | 375035 | 0.8468974 | 4.39235 | 1.47E-03 | 1.01E-02 |
| HNRNPR | 10236 | 0.8468826 | 8.0088112 | 5.04E-05 | 6.35E-04 |
| NIPAL2 | 79815 | 0.8464172 | 1.5011168 | 2.42E-02 | 8.74E-02 |
| SMC2 | 10592 | 0.8461429 | 4.17667 | 2.64E-03 | 1.60E-02 |
| LIN54 | 132660 | 0.845493 | 2.9402258 | 3.59E-03 | 2.05E-02 |
| INCA1 | 388324 | 0.8454662 | 1.8810057 | 8.78E-03 | 4.11E-02 |
| FXR1 | 8087 | 0.8452851 | 5.6618673 | 2.05E-03 | 1.31E-02 |
| THEM4 | 117145 | 0.8448895 | 4.9957238 | 1.10E-03 | 7.97E-03 |
| ASTN2 | 23245 | 0.8441686 | 3.0839376 | 4.69E-02 | 1.41E-01 |
| UHRF1BP1L | 23074 | 0.8432628 | 4.8810762 | 5.19E-04 | 4.35E-03 |
| SUOX | 6821 | 0.8432089 | 4.8519601 | 1.87E-03 | 1.22E-02 |
| ACOT1 | 641371 | 0.8431138 | 3.19483 | 2.96E-02 | 1.01E-01 |
| TRAK1 | 22906 | 0.8416795 | 5.3159765 | 6.58E-03 | 3.31E-02 |
| KDM6A | 7403 | 0.8403651 | 3.2971748 | 7.81E-03 | 3.77E-02 |
| CCN2 | 1490 | 0.8402776 | 9.0061317 | 3.45E-01 | 5.46E-01 |
| RASEF | 158158 | 0.8397722 | -0.352156 | 4.08E-01 | 6.08E-01 |
| AP3M2 | 10947 | 0.8395626 | 5.8089368 | 1.63E-06 | 3.29E-05 |
| ATG10 | 83734 | 0.8395157 | 5.0067828 | 7.00E-03 | 3.46E-02 |
| DIXDC1 | 85458 | 0.8387721 | 5.3678449 | 7.38E-03 | 3.60E-02 |
| PLEKHO2 | 80301 | 0.8386082 | 0.9342652 | 3.45E-01 | 5.46E-01 |
| MSH2 | 4436 | 0.8380323 | 5.7080667 | 7.66E-05 | 9.07E-04 |
| ZNF488 | 118738 | 0.8375055 | 1.3737496 | 7.14E-02 | 1.89E-01 |
| TNPO1 | 3842 | 0.8374796 | 6.0686044 | 3.61E-04 | 3.23E-03 |
| PNMA3 | 29944 | 0.8373933 | 3.7282959 | 9.08E-02 | 2.23E-01 |
| VCPKMT | 79609 | 0.8372642 | 4.5890462 | 3.85E-05 | 4.98E-04 |
| TSPAN6 | 7105 | 0.837183 | 6.4929927 | 4.15E-03 | 2.29E-02 |
| INPP4B | 8821 | 0.8370045 | 2.1206873 | 1.63E-02 | 6.48E-02 |
| LIG4 | 3981 | 0.8368956 | 4.9038399 | 1.19E-04 | 1.29E-03 |
| ITPR1 | 3708 | 0.8365949 | 2.0377226 | 2.78E-01 | 4.75E-01 |
| AGTPBP1 | 23287 | 0.8363616 | 5.348651 | 1.31E-03 | 9.17E-03 |
| FIP1L1 | 81608 | 0.8356524 | 4.8505779 | 1.98E-05 | 2.88E-04 |
| OSGIN2 | 734 | 0.8350287 | 4.7000397 | 3.38E-02 | 1.11E-01 |
| C18orf54 | 162681 | 0.8347394 | 0.5893031 | 1.55E-01 | 3.22E-01 |
| AURKC | 6795 | 0.8333314 | 0.1477298 | 4.16E-01 | 6.15E-01 |
| ZNF143 | 7702 | 0.8332892 | 4.6684303 | 9.07E-05 | 1.04E-03 |
| LIPA | 3988 | 0.8332736 | 5.1500756 | 5.72E-02 | 1.62E-01 |
| MEF2A | 4205 | 0.8331489 | 4.3214738 | 7.72E-05 | 9.11E-04 |
| FNBP1L | 54874 | 0.83201 | 7.8034147 | 3.13E-03 | 1.83E-02 |
| PNRC2 | 55629 | 0.8312005 | 5.8434086 | 6.76E-04 | 5.40E-03 |
| TMEM116 | 89894 | 0.8301716 | 4.4720968 | 1.80E-04 | 1.82E-03 |
| ELAVL2 | 1993 | 0.8297857 | 6.7227316 | 2.15E-03 | 1.36E-02 |
| APBB3 | 10307 | 0.8296917 | 3.706758 | 1.43E-02 | 5.88E-02 |
| DACT2 | 168002 | 0.829374 | 0.2463286 | 2.30E-01 | 4.21E-01 |
| LRRC75A | 388341 | 0.8293197 | 2.7163789 | 1.41E-02 | 5.84E-02 |
| TRIM36 | 55521 | 0.8291954 | 6.3690274 | 5.56E-02 | 1.59E-01 |
| NBPF1 | 55672 | 0.8278698 | 5.8064706 | 2.90E-03 | 1.73E-02 |
| RFX2 | 5990 | 0.8276381 | 3.1171022 | 1.18E-01 | 2.68E-01 |
| ZNF624 | 57547 | 0.8276075 | 3.0479526 | 4.45E-03 | 2.43E-02 |
| FAM8A1 | 51439 | 0.8274182 | 5.3753785 | 7.71E-04 | 6.02E-03 |
| ZNF619 | 285267 | 0.8273988 | 1.5660119 | 7.91E-02 | 2.03E-01 |
| PSMG1 | 8624 | 0.8266709 | 5.8875165 | 2.05E-04 | 2.03E-03 |
| CAPN2 | 824 | 0.8266286 | 6.2046746 | 7.92E-02 | 2.03E-01 |
| CSDE1 | 7812 | 0.8261377 | 8.5828535 | 7.73E-06 | 1.28E-04 |
| CD276 | 80381 | 0.8259322 | 5.9527601 | 4.48E-02 | 1.36E-01 |
| RAB39B | 116442 | 0.8255669 | 5.6878992 | 3.97E-04 | 3.52E-03 |
| ZNF654 | 55279 | 0.8255619 | 3.787654 | 6.14E-03 | 3.15E-02 |
| CFAP43 | 80217 | 0.8253033 | 2.0527786 | 8.60E-02 | 2.15E-01 |
| MRPL42 | 28977 | 0.8251355 | 7.3724152 | 8.99E-06 | 1.46E-04 |
| FHAD1 | 114827 | 0.824792 | -0.311506 | 2.18E-01 | 4.06E-01 |
| LRRC8D | 55144 | 0.8245266 | 5.4728679 | 1.96E-03 | 1.26E-02 |
| TXNRD3 | 114112 | 0.8240724 | 2.4520928 | 5.41E-02 | 1.55E-01 |
| PIR | 8544 | 0.8240039 | 6.2942293 | 7.13E-03 | 3.51E-02 |
| WDPCP | 51057 | 0.8238576 | 3.1604481 | 4.78E-04 | 4.08E-03 |
| PCK2 | 5106 | 0.8235229 | 3.1328762 | 1.82E-01 | 3.61E-01 |
| LHFPL1 | 340596 | 0.8233467 | 0.6037123 | 1.14E-01 | 2.62E-01 |
| COMMD10 | 51397 | 0.8232404 | 5.494814 | 7.04E-05 | 8.45E-04 |
| TM4SF1 | 4071 | 0.8232115 | 2.1056184 | 3.95E-01 | 5.95E-01 |
| ZNF695 | 57116 | 0.8222346 | -0.288574 | 2.16E-01 | 4.04E-01 |
| GPCPD1 | 56261 | 0.8220735 | 3.8228413 | 1.18E-02 | 5.12E-02 |
| COG4 | 25839 | 0.8215077 | 5.5963007 | 2.27E-03 | 1.42E-02 |
| MTTP | 4547 | 0.8211291 | 2.3847204 | 4.57E-02 | 1.38E-01 |
| N4BP2 | 55728 | 0.8204868 | 5.4799234 | 8.53E-03 | 4.02E-02 |
| DOCK4 | 9732 | 0.8203029 | 3.5069332 | 1.84E-03 | 1.20E-02 |
| ZFP36L1 | 677 | 0.8199969 | 3.0020356 | 2.16E-01 | 4.04E-01 |
| TOX2 | 84969 | 0.8198644 | 3.6873105 | 1.38E-02 | 5.73E-02 |
| PNMA8A | 55228 | 0.8198447 | 6.1213826 | 2.35E-02 | 8.52E-02 |
| ITGB1 | 3688 | 0.8197437 | 6.8481881 | 1.70E-01 | 3.44E-01 |
| HSPA4 | 3308 | 0.8197368 | 6.7966138 | 1.69E-03 | 1.12E-02 |
| PACS1 | 55690 | 0.8195572 | 5.1935259 | 1.38E-03 | 9.55E-03 |
| PTPN9 | 5780 | 0.8194582 | 5.3851085 | 1.49E-04 | 1.55E-03 |
| BARHL2 | 343472 | 0.8193261 | -0.192846 | 4.09E-01 | 6.09E-01 |
| CDT1 | 81620 | 0.8189084 | 1.0194984 | 4.45E-02 | 1.35E-01 |
| PLEKHA6 | 22874 | 0.8188976 | 3.3297791 | 1.22E-03 | 8.66E-03 |
| GLT8D2 | 83468 | 0.818554 | 2.6907706 | 4.05E-02 | 1.27E-01 |
| MAP3K3 | 4215 | 0.8178785 | 2.9643636 | 2.44E-02 | 8.77E-02 |
| ACAA2 | 10449 | 0.8168992 | 4.8262648 | 4.64E-02 | 1.40E-01 |
| CAB39 | 51719 | 0.816694 | 6.0771662 | 2.00E-04 | 1.98E-03 |
| C1orf112 | 55732 | 0.8152259 | 1.4531281 | 2.90E-02 | 9.92E-02 |
| ZNF738 | 148203 | 0.8149608 | 5.5494299 | 1.45E-01 | 3.09E-01 |
| ATAD2 | 29028 | 0.8149597 | 2.1618148 | 1.84E-01 | 3.63E-01 |
| CD164 | 8763 | 0.8147903 | 6.4396611 | 8.46E-02 | 2.12E-01 |
| ACLY | 47 | 0.8146376 | 6.6121731 | 2.55E-03 | 1.56E-02 |
| CGN | 57530 | 0.8146211 | 1.4620093 | 1.06E-01 | 2.49E-01 |
| PAQR8 | 85315 | 0.8145978 | 3.0566176 | 1.77E-02 | 6.91E-02 |
| STON1 | 11037 | 0.8145412 | 1.0548095 | 2.65E-01 | 4.60E-01 |
| HTATIP2 | 10553 | 0.8130372 | 3.237034 | 6.38E-03 | 3.25E-02 |
| ZNF425 | 155054 | 0.8117337 | 3.0740323 | 3.25E-03 | 1.89E-02 |
| HMGCS1 | 3157 | 0.8115408 | 6.522386 | 1.31E-03 | 9.17E-03 |
| KRI1 | 65095 | 0.8111516 | 3.657473 | 3.00E-02 | 1.02E-01 |
| AKAIN1 | 642597 | 0.8098079 | 0.2772975 | 8.70E-02 | 2.16E-01 |
| KIAA0895 | 23366 | 0.8085782 | 5.6702007 | 7.45E-04 | 5.85E-03 |
| SRSF8 | 10929 | 0.8084174 | 6.6210982 | 3.38E-05 | 4.47E-04 |
| RASSF5 | 83593 | 0.8077378 | 1.214447 | 2.65E-01 | 4.61E-01 |
| FPGT | 8790 | 0.8076615 | 3.0623582 | 2.82E-02 | 9.73E-02 |
| WDCP | 80304 | 0.807054 | 3.7804202 | 2.38E-03 | 1.47E-02 |
| CPSF2 | 53981 | 0.806166 | 5.5464392 | 1.03E-03 | 7.56E-03 |
| SPATA6L | 55064 | 0.8053024 | 1.9803223 | 1.39E-02 | 5.77E-02 |
| CBLB | 868 | 0.8048463 | 3.7396056 | 1.31E-03 | 9.14E-03 |
| RB1 | 5925 | 0.8044423 | 4.7325216 | 1.57E-03 | 1.05E-02 |
| GAL3ST1 | 9514 | 0.8040536 | 2.7198819 | 1.35E-01 | 2.94E-01 |
| PLEKHG1 | 57480 | 0.8031554 | 3.3958252 | 3.84E-02 | 1.22E-01 |
| C5orf22 | 55322 | 0.80235 | 5.2512512 | 7.69E-05 | 9.10E-04 |
| MAD2L1 | 4085 | 0.8020706 | 3.9601455 | 1.10E-02 | 4.86E-02 |
| FUBP3 | 8939 | 0.8015311 | 5.1426515 | 5.65E-03 | 2.96E-02 |
| SLC2A11 | 66035 | 0.801218 | 3.3245432 | 1.78E-03 | 1.17E-02 |
| MCL1 | 4170 | 0.801009 | 5.7493184 | 1.20E-03 | 8.57E-03 |
| SHTN1 | 57698 | 0.801003 | 6.1963862 | 6.32E-02 | 1.73E-01 |
| IBTK | 25998 | 0.8001837 | 5.6923521 | 1.05E-04 | 1.18E-03 |
| TIPARP | 25976 | 0.7991214 | 4.4195085 | 1.36E-01 | 2.95E-01 |
| PBRM1 | 55193 | 0.798974 | 7.010568 | 1.32E-03 | 9.20E-03 |
| CYLD | 1540 | 0.7988782 | 5.281774 | 1.11E-03 | 8.01E-03 |
| ISOC1 | 51015 | 0.7978601 | 6.0570797 | 1.90E-02 | 7.30E-02 |
| OLFM1 | 10439 | 0.7978355 | 8.3220027 | 2.39E-01 | 4.30E-01 |
| ZNF638 | 27332 | 0.7976989 | 6.3634028 | 7.72E-05 | 9.11E-04 |
| SULT1C4 | 27233 | 0.797675 | 2.125305 | 4.13E-02 | 1.29E-01 |
| NAMPT | 10135 | 0.7973227 | 6.2915037 | 4.22E-02 | 1.31E-01 |
| GCK | 2645 | 0.7970559 | 3.8887865 | 6.99E-02 | 1.86E-01 |
| SNCAIP | 9627 | 0.7968542 | 3.4035071 | 2.06E-02 | 7.74E-02 |
| ZNF10 | 7556 | 0.7968526 | 4.0783753 | 2.32E-04 | 2.23E-03 |
| KCTD21 | 283219 | 0.7964312 | 2.8063467 | 6.10E-03 | 3.14E-02 |
| SLC25A43 | 203427 | 0.7964311 | 1.8903671 | 1.33E-01 | 2.91E-01 |
| THAP10 | 56906 | 0.7958808 | 3.4380731 | 7.09E-03 | 3.50E-02 |
| ZNF770 | 54989 | 0.7955606 | 7.1943208 | 2.47E-03 | 1.52E-02 |
| RILP | 83547 | 0.7954688 | 2.0495032 | 1.18E-02 | 5.12E-02 |
| IRF2BP2 | 359948 | 0.7950507 | 4.9181887 | 3.29E-03 | 1.91E-02 |
| MYOZ1 | 58529 | 0.7944665 | 0.3724988 | 2.50E-01 | 4.44E-01 |
| TTPAL | 79183 | 0.793924 | 4.5158216 | 2.58E-04 | 2.44E-03 |
| HBP1 | 26959 | 0.7938304 | 4.3147178 | 9.06E-02 | 2.23E-01 |
| SPAG9 | 9043 | 0.7934855 | 6.3377558 | 2.72E-03 | 1.64E-02 |
| WASF1 | 8936 | 0.7934281 | 7.4878038 | 8.59E-02 | 2.14E-01 |
| SS18 | 6760 | 0.7934 | 5.2333378 | 2.47E-02 | 8.85E-02 |
| PANK1 | 53354 | 0.7928716 | 4.2105339 | 3.29E-03 | 1.91E-02 |
| PCCA | 5095 | 0.792841 | 4.9551665 | 7.62E-04 | 5.97E-03 |
| NBPF26 | 101060684 | 0.7923894 | 1.2219038 | 1.43E-01 | 3.05E-01 |
| NRCAM | 4897 | 0.791897 | 6.3577974 | 3.61E-02 | 1.17E-01 |
| LAMP2 | 3920 | 0.7911821 | 7.4718694 | 1.20E-02 | 5.16E-02 |
| MAP4K4 | 9448 | 0.7908253 | 6.5511036 | 5.92E-05 | 7.31E-04 |
| INO80C | 125476 | 0.7906589 | 4.1311529 | 6.44E-03 | 3.27E-02 |
| RPS6KC1 | 26750 | 0.7904131 | 4.8657349 | 6.06E-04 | 4.93E-03 |
| ATP8A2 | 51761 | 0.7901744 | 4.8519818 | 3.40E-01 | 5.41E-01 |
| MINDY1 | 55793 | 0.7898623 | 3.4370499 | 2.55E-03 | 1.56E-02 |
| SYT7 | 9066 | 0.7896424 | 3.8074931 | 4.51E-01 | 6.44E-01 |
| PPIL3 | 53938 | 0.7896363 | 5.6443784 | 5.83E-05 | 7.23E-04 |
| MIS18BP1 | 55320 | 0.7893303 | 2.2062705 | 4.70E-02 | 1.41E-01 |
| KIAA0232 | 9778 | 0.7889487 | 6.39645 | 2.34E-04 | 2.24E-03 |
| DCTN4 | 51164 | 0.788795 | 5.7961337 | 3.64E-05 | 4.75E-04 |
| GOLGA1 | 2800 | 0.7886164 | 3.8142864 | 2.32E-04 | 2.23E-03 |
| PIP4K2C | 79837 | 0.7885364 | 5.1647744 | 3.10E-02 | 1.04E-01 |
| YWHAQ | 10971 | 0.7878992 | 10.934623 | 5.44E-04 | 4.52E-03 |
| CUL4A | 8451 | 0.7871993 | 5.0407999 | 3.54E-02 | 1.15E-01 |
| ELFN1 | 392617 | 0.7871197 | 2.2939688 | 1.10E-01 | 2.55E-01 |
| ZNF254 | 9534 | 0.7865355 | 4.2346777 | 1.32E-02 | 5.56E-02 |
| PRKAR1A | 5573 | 0.7864857 | 8.6940939 | 3.09E-05 | 4.17E-04 |
| CD58 | 965 | 0.7860218 | 4.789775 | 2.35E-02 | 8.52E-02 |
| TOP2A | 7153 | 0.7858937 | 2.5788641 | 1.89E-01 | 3.69E-01 |
| GPR83 | 10888 | 0.7853028 | 1.9631042 | 2.40E-01 | 4.31E-01 |
| CLDN6 | 9074 | 0.7839336 | 5.3861355 | 1.34E-01 | 2.93E-01 |
| TSNAX | 7257 | 0.7838282 | 5.6793259 | 1.64E-04 | 1.69E-03 |
| BMP8A | 353500 | 0.7833537 | -0.110578 | 1.30E-01 | 2.88E-01 |
| FANCL | 55120 | 0.7832757 | 4.7688663 | 2.21E-03 | 1.39E-02 |
| PREP | 5550 | 0.7832203 | 5.6631678 | 9.50E-05 | 1.08E-03 |
| TULP3 | 7289 | 0.7829806 | 4.1610647 | 6.81E-03 | 3.39E-02 |
| CCDC146 | 57639 | 0.7827754 | 1.7580502 | 1.62E-01 | 3.32E-01 |
| RAB11FIP1 | 80223 | 0.7827063 | 2.8470301 | 3.26E-02 | 1.08E-01 |
| PEPD | 5184 | 0.7822431 | 7.4694128 | 3.46E-05 | 4.56E-04 |
| ZNF83 | 55769 | 0.7820559 | 3.8476258 | 9.61E-03 | 4.38E-02 |
| GTSE1 | 51512 | 0.7819528 | 0.343082 | 3.50E-01 | 5.50E-01 |
| RNF2 | 6045 | 0.7818495 | 5.1383344 | 2.07E-05 | 2.98E-04 |
| CBX1 | 10951 | 0.781708 | 7.307394 | 1.69E-02 | 6.66E-02 |
| NUDT15 | 55270 | 0.7814624 | 5.0690083 | 2.28E-04 | 2.21E-03 |
| ZNF195 | 7748 | 0.7813327 | 6.2297695 | 7.14E-04 | 5.66E-03 |
| ERMARD | 55780 | 0.7810301 | 3.689744 | 3.57E-03 | 2.04E-02 |
| RCC2 | 55920 | 0.7808755 | 3.4122061 | 4.92E-02 | 1.45E-01 |
| MACIR | 90355 | 0.7806058 | 5.8331507 | 1.80E-04 | 1.82E-03 |
| TNPO3 | 23534 | 0.7803522 | 6.5499943 | 6.29E-05 | 7.71E-04 |
| CNOT11 | 55571 | 0.7803388 | 5.0762298 | 7.96E-03 | 3.82E-02 |
| MGAT4A | 11320 | 0.7803346 | 4.4450341 | 1.40E-03 | 9.67E-03 |
| TADA1 | 117143 | 0.7802855 | 6.6505125 | 7.69E-03 | 3.73E-02 |
| MAP4 | 4134 | 0.7800394 | 8.6412129 | 3.94E-05 | 5.08E-04 |
| PTX3 | 5806 | 0.7794262 | 5.1995656 | 3.13E-01 | 5.12E-01 |
| LIMS2 | 55679 | 0.7792196 | 0.7719581 | 2.61E-01 | 4.56E-01 |
| C14orf132 | 56967 | 0.7788688 | 5.9208863 | 1.82E-02 | 7.05E-02 |
| G3BP2 | 9908 | 0.7785125 | 8.0021234 | 4.99E-05 | 6.31E-04 |
| FDXACB1 | 91893 | 0.7784308 | 2.6718904 | 2.04E-02 | 7.68E-02 |
| SNCB | 6620 | 0.7779175 | 6.5144115 | 2.25E-01 | 4.14E-01 |
| SLC22A15 | 55356 | 0.777669 | 2.949515 | 4.85E-02 | 1.44E-01 |
| AFMID | 125061 | 0.7771428 | 2.5006087 | 1.33E-02 | 5.60E-02 |
| EIF4A2 | 1974 | 0.7771119 | 8.7613678 | 6.16E-05 | 7.58E-04 |
| ARMT1 | 79624 | 0.7769351 | 4.9832782 | 6.01E-04 | 4.90E-03 |
| CFAP77 | 389799 | 0.7768239 | 1.1578633 | 3.92E-01 | 5.92E-01 |
| KBTBD2 | 25948 | 0.776766 | 5.1602154 | 2.45E-04 | 2.34E-03 |
| ASNSD1 | 54529 | 0.7764283 | 5.1777559 | 1.83E-05 | 2.67E-04 |
| HDAC2 | 3066 | 0.7761566 | 9.0440735 | 1.74E-03 | 1.16E-02 |
| ELMO2 | 63916 | 0.7758802 | 5.5900187 | 6.77E-05 | 8.15E-04 |
| FDPS | 2224 | 0.7756768 | 8.3365821 | 3.16E-04 | 2.90E-03 |
| PPP2R2C | 5522 | 0.7756352 | 3.1415712 | 1.41E-01 | 3.02E-01 |
| CUL2 | 8453 | 0.7748658 | 5.7045226 | 5.49E-05 | 6.86E-04 |
| ZNF799 | 90576 | 0.7743265 | 2.0180014 | 1.58E-02 | 6.34E-02 |
| RFK | 55312 | 0.773923 | 7.5714054 | 2.96E-05 | 4.02E-04 |
| SMPD4 | 55627 | 0.7734299 | 4.9308766 | 1.35E-03 | 9.37E-03 |
| APEX2 | 27301 | 0.7732049 | 3.4953049 | 4.53E-02 | 1.37E-01 |
| HNRNPH3 | 3189 | 0.7726411 | 7.5250007 | 3.99E-05 | 5.14E-04 |
| PPP1R21 | 129285 | 0.7725578 | 4.7756615 | 9.45E-04 | 7.06E-03 |
| PLXNC1 | 10154 | 0.7722691 | 4.2668756 | 7.80E-02 | 2.01E-01 |
| USP14 | 9097 | 0.7722619 | 7.1662514 | 4.70E-05 | 5.96E-04 |
| ZFAND5 | 7763 | 0.7722063 | 8.1166059 | 2.90E-03 | 1.73E-02 |
| IGF2BP3 | 10643 | 0.7721295 | 3.5587416 | 3.85E-02 | 1.22E-01 |
| FARP2 | 9855 | 0.7719931 | 3.3038733 | 1.16E-01 | 2.65E-01 |
| CREBL2 | 1389 | 0.7717272 | 5.2830067 | 7.53E-04 | 5.91E-03 |
| DCBLD1 | 285761 | 0.7709856 | 2.7011905 | 5.48E-03 | 2.89E-02 |
| TARDBP | 23435 | 0.7705412 | 6.8882528 | 2.48E-05 | 3.45E-04 |
| MAP2K1 | 5604 | 0.7696584 | 4.8716429 | 3.34E-02 | 1.10E-01 |
| NRAS | 4893 | 0.7692882 | 6.0102298 | 2.14E-04 | 2.10E-03 |
| REV1 | 51455 | 0.7691562 | 5.1945213 | 2.75E-04 | 2.57E-03 |
| COL9A1 | 1297 | 0.7690084 | -0.417226 | 5.18E-01 | 6.99E-01 |
| LRP8 | 7804 | 0.7686924 | 4.9180103 | 2.93E-03 | 1.74E-02 |
| ERC2 | 26059 | 0.7684877 | 5.1291102 | 2.34E-01 | 4.26E-01 |
| FANCB | 2187 | 0.7677955 | 0.5324747 | 1.26E-01 | 2.81E-01 |
| KIAA0586 | 9786 | 0.7673285 | 4.0479239 | 4.03E-04 | 3.56E-03 |
| LMCD1 | 29995 | 0.7663739 | 2.8442032 | 1.57E-01 | 3.25E-01 |
| SLC35G1 | 159371 | 0.7663677 | 3.9579392 | 3.18E-04 | 2.91E-03 |
| KLHL24 | 54800 | 0.7663333 | 5.063538 | 2.46E-02 | 8.84E-02 |
| CHURC1 | 91612 | 0.766032 | 5.6225207 | 1.66E-04 | 1.70E-03 |
| MAB21L1 | 4081 | 0.7659457 | 4.4862538 | 3.95E-01 | 5.95E-01 |
| CTNNAL1 | 8727 | 0.7653816 | 3.7279259 | 1.85E-01 | 3.64E-01 |
| HSPA4L | 22824 | 0.7652875 | 5.8040837 | 1.09E-02 | 4.82E-02 |
| NRDC | 4898 | 0.7650208 | 7.1294343 | 3.51E-05 | 4.60E-04 |
| ECEL1 | 9427 | 0.7647482 | 6.4130225 | 5.71E-04 | 4.70E-03 |
| SLC7A14 | 57709 | 0.7645832 | 3.4921075 | 1.33E-01 | 2.92E-01 |
| ARMCX5 | 64860 | 0.76418 | 4.1835778 | 1.73E-04 | 1.76E-03 |
| ZNF808 | 388558 | 0.7640873 | 2.3442927 | 2.21E-02 | 8.17E-02 |
| SAV1 | 60485 | 0.7634148 | 4.6120365 | 4.95E-02 | 1.46E-01 |
| RNF38 | 152006 | 0.7617144 | 5.1739542 | 3.50E-03 | 2.01E-02 |
| TMEM86A | 144110 | 0.7617 | 2.1573385 | 1.71E-01 | 3.45E-01 |
| GABARAP | 11337 | 0.7616765 | 1.6213616 | 3.38E-02 | 1.11E-01 |
| PLEKHA8 | 84725 | 0.7615973 | 4.4422028 | 2.55E-04 | 2.41E-03 |
| PHTF2 | 57157 | 0.7613536 | 4.805296 | 4.00E-03 | 2.23E-02 |
| LIMCH1 | 22998 | 0.7612766 | 5.3044457 | 4.63E-03 | 2.51E-02 |
| MOXD1 | 26002 | 0.7611265 | 3.7751878 | 1.13E-01 | 2.60E-01 |
| NCOA4 | 8031 | 0.7606426 | 7.2602524 | 1.31E-04 | 1.40E-03 |
| TTC30A | 92104 | 0.7601787 | 2.8311457 | 6.06E-02 | 1.69E-01 |
| BEST1 | 7439 | 0.7600301 | 1.2450411 | 1.22E-01 | 2.75E-01 |
| SMOC1 | 64093 | 0.7599639 | 1.4345295 | 8.07E-02 | 2.06E-01 |
| SEC31A | 22872 | 0.7596239 | 6.8296032 | 3.86E-02 | 1.22E-01 |
| MCM9 | 254394 | 0.7592321 | 1.5734314 | 1.55E-01 | 3.21E-01 |
| DST | 667 | 0.7591807 | 7.0530391 | 7.43E-02 | 1.94E-01 |
| LDHA | 3939 | 0.7585169 | 8.6681505 | 1.65E-01 | 3.36E-01 |
| KRCC1 | 51315 | 0.7583232 | 4.4018698 | 1.95E-04 | 1.95E-03 |
| IARS1 | 3376 | 0.7579573 | 6.2882634 | 5.43E-02 | 1.56E-01 |
| FUCA1 | 2517 | 0.7575502 | 5.9573001 | 3.55E-05 | 4.65E-04 |
| MAPK6 | 5597 | 0.7569403 | 6.1203759 | 2.95E-05 | 4.01E-04 |
| POC1B | 282809 | 0.7567165 | 3.2334768 | 6.55E-02 | 1.77E-01 |
| UMAD1 | 729852 | 0.7565843 | 4.3064019 | 1.83E-03 | 1.19E-02 |
| NSDHL | 50814 | 0.7563062 | 7.1431882 | 7.75E-04 | 6.04E-03 |
| SCN2A | 6326 | 0.7558867 | 7.274713 | 3.96E-01 | 5.97E-01 |
| SEC24A | 10802 | 0.7555971 | 3.627509 | 6.03E-02 | 1.68E-01 |
| NAGA | 4668 | 0.7554788 | 3.7458414 | 4.30E-02 | 1.32E-01 |
| TMEM200A | 114801 | 0.7554741 | 4.3532206 | 4.85E-03 | 2.61E-02 |
| INA | 9118 | 0.7554202 | 9.8823108 | 3.02E-01 | 5.01E-01 |
| MAPK1IP1L | 93487 | 0.7552735 | 7.424022 | 1.95E-03 | 1.26E-02 |
| RBKS | 64080 | 0.7551774 | 3.8328451 | 8.53E-03 | 4.02E-02 |
| MLH1 | 4292 | 0.7546361 | 5.5839619 | 4.67E-05 | 5.93E-04 |
| GIGYF2 | 26058 | 0.7544067 | 5.5501173 | 5.20E-04 | 4.36E-03 |
| BPIFC | 254240 | 0.7542003 | 1.0004441 | 4.73E-01 | 6.62E-01 |
| TBCCD1 | 55171 | 0.7541699 | 4.2880297 | 2.08E-04 | 2.05E-03 |
| ALG8 | 79053 | 0.7531855 | 5.4158894 | 7.84E-03 | 3.78E-02 |
| RAB22A | 57403 | 0.7531425 | 6.5769678 | 1.61E-04 | 1.66E-03 |
| BRMS1L | 84312 | 0.7530242 | 5.1875933 | 8.09E-03 | 3.86E-02 |
| ADAM10 | 102 | 0.7529185 | 5.2202558 | 7.57E-03 | 3.68E-02 |
| CKAP5 | 9793 | 0.7525703 | 6.404958 | 2.09E-03 | 1.33E-02 |
| DNAJB9 | 4189 | 0.7523558 | 6.9492745 | 1.99E-04 | 1.98E-03 |
| CDK2AP1 | 8099 | 0.7523003 | 8.0817594 | 1.42E-02 | 5.85E-02 |
| RRAGB | 10325 | 0.7519464 | 6.2704116 | 1.31E-03 | 9.14E-03 |
| ARID3B | 10620 | 0.7510826 | 2.8488095 | 3.63E-02 | 1.17E-01 |
| RIN2 | 54453 | 0.7509681 | 2.1184692 | 6.59E-02 | 1.78E-01 |
| CIPC | 85457 | 0.7508965 | 4.5387409 | 2.32E-04 | 2.23E-03 |
| NSF | 4905 | 0.7502232 | 6.5909039 | 1.01E-03 | 7.46E-03 |
| RIOK3 | 8780 | 0.7501339 | 6.4480216 | 1.39E-04 | 1.47E-03 |
| TRIL | 9865 | 0.7498672 | 1.9767461 | 1.07E-01 | 2.51E-01 |
| CEP135 | 9662 | 0.7496131 | 2.6813195 | 4.46E-03 | 2.43E-02 |
| PTBP2 | 58155 | 0.7494846 | 6.5680496 | 2.02E-01 | 3.87E-01 |
| C2orf73 | 129852 | 0.7484238 | -0.052139 | 4.23E-01 | 6.22E-01 |
| WBP11 | 51729 | 0.748199 | 6.6971152 | 2.00E-05 | 2.89E-04 |
| TRIB3 | 57761 | 0.7480957 | 5.2033825 | 6.32E-03 | 3.22E-02 |
| UBE2J1 | 51465 | 0.7479821 | 6.6316608 | 2.15E-05 | 3.08E-04 |
| SMIM10L2B | 644596 | 0.7477455 | 3.6588916 | 6.83E-03 | 3.40E-02 |
| ICMT | 23463 | 0.7470521 | 5.4620796 | 1.21E-02 | 5.20E-02 |
| ASH2L | 9070 | 0.7468092 | 5.8295052 | 9.10E-05 | 1.04E-03 |
| UBE2R2 | 54926 | 0.7467862 | 6.8231657 | 6.71E-05 | 8.10E-04 |
| RAB23 | 51715 | 0.7463819 | 4.1294043 | 1.42E-03 | 9.74E-03 |
| ARFGEF1 | 10565 | 0.7463535 | 4.9477152 | 7.17E-04 | 5.68E-03 |
| ADAM29 | 11086 | 0.7461066 | 0.6389785 | 8.18E-02 | 2.08E-01 |
| C1orf50 | 79078 | 0.7460379 | 2.9771617 | 7.15E-02 | 1.89E-01 |
| WASHC2A | 387680 | 0.7450748 | 4.8104133 | 9.95E-05 | 1.12E-03 |
| TMEM135 | 65084 | 0.7449066 | 3.2765063 | 4.46E-02 | 1.36E-01 |
| RNGTT | 8732 | 0.7440154 | 4.619918 | 3.50E-04 | 3.15E-03 |
| CUL4B | 8450 | 0.7435846 | 4.9068986 | 9.57E-03 | 4.37E-02 |
| PDS5B | 23047 | 0.7434414 | 5.7467094 | 1.08E-03 | 7.89E-03 |
| PCYOX1 | 51449 | 0.7432109 | 6.8203495 | 3.51E-03 | 2.01E-02 |
| SLC2A10 | 81031 | 0.7425511 | 1.8833947 | 4.43E-01 | 6.36E-01 |
| IQCH | 64799 | 0.7415104 | 1.891277 | 2.86E-02 | 9.82E-02 |
| RIMKLB | 57494 | 0.7412211 | 5.7913702 | 2.47E-02 | 8.85E-02 |
| RNF146 | 81847 | 0.7411503 | 6.7337974 | 4.50E-04 | 3.89E-03 |
| GDA | 9615 | 0.7409283 | -0.183113 | 2.69E-01 | 4.65E-01 |
| COG6 | 57511 | 0.7407746 | 4.5344717 | 2.55E-02 | 9.07E-02 |
| ZFYVE16 | 9765 | 0.7406397 | 3.651616 | 3.60E-02 | 1.16E-01 |
| KAT7 | 11143 | 0.7405162 | 5.3086112 | 2.09E-03 | 1.33E-02 |
| ZNF711 | 7552 | 0.7396913 | 5.5424477 | 2.02E-02 | 7.62E-02 |
| DCP1B | 196513 | 0.7395653 | 4.4128361 | 2.25E-03 | 1.41E-02 |
| SEC23A | 10484 | 0.7394705 | 6.4465153 | 1.29E-02 | 5.43E-02 |
| PGM3 | 5238 | 0.7388102 | 5.2327183 | 6.41E-02 | 1.74E-01 |
| TMEM209 | 84928 | 0.7385829 | 4.3176495 | 1.60E-02 | 6.41E-02 |
| MARCHF7 | 64844 | 0.7383298 | 6.2583436 | 4.60E-05 | 5.84E-04 |
| ADGRA1 | 84435 | 0.7381904 | 2.820718 | 3.09E-01 | 5.08E-01 |
| ERBB2 | 2064 | 0.7381475 | 2.4095702 | 2.05E-01 | 3.90E-01 |
| RPA1 | 6117 | 0.7380905 | 5.32292 | 2.59E-02 | 9.15E-02 |
| H2AZ2 | 94239 | 0.7378683 | 6.4458485 | 7.64E-04 | 5.98E-03 |
| EXOC1 | 55763 | 0.7372044 | 5.7753791 | 1.43E-04 | 1.50E-03 |
| ZBTB33 | 10009 | 0.7371996 | 4.3111451 | 8.73E-04 | 6.62E-03 |
| WIPI1 | 55062 | 0.7368243 | 3.8550345 | 1.32E-01 | 2.90E-01 |
| ZSWIM9 | 374920 | 0.736698 | 2.2983655 | 1.79E-02 | 6.96E-02 |
| LNX2 | 222484 | 0.7360691 | 2.5606651 | 2.28E-02 | 8.33E-02 |
| IRAK2 | 3656 | 0.7359273 | 2.341216 | 1.51E-02 | 6.13E-02 |
| C11orf1 | 64776 | 0.7359205 | 5.5187223 | 1.46E-02 | 5.98E-02 |
| CHIC1 | 53344 | 0.7353114 | 2.002774 | 3.81E-02 | 1.21E-01 |
| HNRNPH2 | 3188 | 0.7352418 | 7.7401025 | 7.50E-05 | 8.91E-04 |
| CPXM1 | 56265 | 0.7349019 | 4.3098165 | 6.20E-04 | 5.01E-03 |
| CFAP97 | 57587 | 0.7348648 | 6.3029979 | 2.52E-04 | 2.38E-03 |
| ALPK1 | 80216 | 0.734613 | 1.1928658 | 2.80E-01 | 4.78E-01 |
| ATF2 | 1386 | 0.7345575 | 6.495271 | 8.41E-04 | 6.42E-03 |
| ZNF627 | 199692 | 0.7340924 | 5.7382911 | 4.13E-04 | 3.64E-03 |
| GLDC | 2731 | 0.7336871 | 2.9437081 | 1.41E-02 | 5.84E-02 |
| COQ10A | 93058 | 0.7334151 | 4.9207203 | 8.21E-04 | 6.32E-03 |
| JKAMP | 51528 | 0.7333712 | 7.4967236 | 3.77E-03 | 2.13E-02 |
| ARHGAP18 | 93663 | 0.7327112 | 3.4646797 | 1.53E-02 | 6.20E-02 |
| ZFP37 | 7539 | 0.7326768 | 4.4324617 | 3.82E-02 | 1.21E-01 |
| SPIRE1 | 56907 | 0.7324552 | 5.2782037 | 2.29E-02 | 8.37E-02 |
| FBXW11 | 23291 | 0.7324244 | 6.1238964 | 7.40E-05 | 8.83E-04 |
| SNRK | 54861 | 0.7321677 | 5.3670852 | 5.88E-03 | 3.05E-02 |
| TBX1 | 6899 | 0.7321642 | 3.1739803 | 2.45E-02 | 8.81E-02 |
| RADIL | 55698 | 0.7320521 | 3.091577 | 3.31E-01 | 5.32E-01 |
| RAB6A | 5870 | 0.7314266 | 8.4526312 | 4.52E-04 | 3.90E-03 |
| ZIC3 | 7547 | 0.7313698 | 5.0441538 | 3.79E-02 | 1.21E-01 |
| WDR93 | 56964 | 0.7309923 | 0.4309938 | 1.55E-01 | 3.22E-01 |
| ZFP2 | 80108 | 0.7306569 | 3.1553028 | 1.47E-02 | 6.00E-02 |
| NHSL2 | 392490 | 0.7306007 | 3.6023283 | 6.51E-03 | 3.28E-02 |
| TGIF1 | 7050 | 0.7303603 | 5.4274698 | 1.69E-01 | 3.42E-01 |
| GNA13 | 10672 | 0.730203 | 4.3896844 | 6.00E-03 | 3.10E-02 |
| CCDC159 | 126075 | 0.7301389 | 1.7864 | 2.20E-01 | 4.09E-01 |
| HDAC6 | 10013 | 0.7300081 | 4.9502008 | 4.65E-04 | 3.99E-03 |
| PSAT1 | 29968 | 0.7293045 | 6.6354641 | 8.70E-02 | 2.16E-01 |
| EPRS1 | 2058 | 0.7290615 | 7.1595215 | 6.68E-05 | 8.08E-04 |
| TMEM176A | 55365 | 0.7285625 | 0.7560652 | 3.63E-01 | 5.62E-01 |
| ANKRD7 | 56311 | 0.7285102 | 1.654584 | 2.29E-01 | 4.20E-01 |
| ZNF438 | 220929 | 0.7285093 | 2.1896146 | 4.75E-02 | 1.42E-01 |
| PFN2 | 5217 | 0.7282616 | 7.2997725 | 1.52E-03 | 1.03E-02 |
| NONO | 4841 | 0.7282347 | 7.8236655 | 4.18E-04 | 3.67E-03 |
| NGF | 4803 | 0.7278852 | 0.6891403 | 3.22E-01 | 5.22E-01 |
| GATM | 2628 | 0.7272467 | 4.2218757 | 6.09E-03 | 3.14E-02 |
| PPP4R3B | 57223 | 0.726816 | 6.1903815 | 1.97E-04 | 1.96E-03 |
| CCDC138 | 165055 | 0.7265177 | 3.2114626 | 4.35E-03 | 2.38E-02 |
| STRBP | 55342 | 0.7258976 | 7.4366107 | 1.50E-03 | 1.02E-02 |
| HP1BP3 | 50809 | 0.7257957 | 6.9733751 | 8.31E-04 | 6.37E-03 |
| AP3B1 | 8546 | 0.7252537 | 4.3252028 | 1.26E-02 | 5.35E-02 |
| HSD3B7 | 80270 | 0.7246526 | 0.9987895 | 2.27E-01 | 4.17E-01 |
| PDK1 | 5163 | 0.7242174 | 4.0661322 | 4.64E-02 | 1.40E-01 |
| WDR5B | 54554 | 0.7239075 | 2.9030775 | 4.97E-02 | 1.46E-01 |
| SLC30A9 | 10463 | 0.7236141 | 7.2076001 | 4.16E-05 | 5.33E-04 |
| ATP6V1B2 | 526 | 0.7234565 | 8.7470878 | 4.68E-02 | 1.40E-01 |
| ZNF185 | 7739 | 0.7233689 | 2.5672791 | 6.74E-02 | 1.81E-01 |
| FSTL4 | 23105 | 0.7220704 | 0.3763391 | 1.78E-01 | 3.55E-01 |
| ZNF66 | 7617 | 0.7218616 | 1.7562223 | 6.47E-02 | 1.76E-01 |
| TRAF3IP2 | 10758 | 0.7217036 | 3.2718498 | 1.55E-02 | 6.27E-02 |
| SLC20A2 | 6575 | 0.7216104 | 4.0446736 | 5.11E-02 | 1.49E-01 |
| USP30 | 84749 | 0.7214539 | 4.3685846 | 4.34E-04 | 3.77E-03 |
| PCDHAC2 | 56134 | 0.7209799 | 3.1311767 | 8.01E-03 | 3.83E-02 |
| AKAP12 | 9590 | 0.7205847 | 8.1363693 | 1.44E-04 | 1.51E-03 |
| C5orf24 | 134553 | 0.7204387 | 6.241388 | 1.10E-04 | 1.22E-03 |
| STX3 | 6809 | 0.7203976 | 4.5227147 | 5.59E-03 | 2.93E-02 |
| TRAIP | 10293 | 0.720226 | 0.981304 | 1.54E-01 | 3.20E-01 |
| EIF2A | 83939 | 0.7200838 | 5.4073249 | 3.42E-04 | 3.08E-03 |
| CDK14 | 5218 | 0.7199116 | 4.7816303 | 5.10E-02 | 1.48E-01 |
| TSN | 7247 | 0.719774 | 7.0000766 | 1.47E-04 | 1.53E-03 |
| UBTF | 7343 | 0.718955 | 5.301328 | 1.07E-03 | 7.81E-03 |
| CBLL1 | 79872 | 0.7189259 | 5.461112 | 6.67E-05 | 8.07E-04 |
| ZFP90 | 146198 | 0.7188933 | 5.944626 | 8.59E-04 | 6.53E-03 |
| AFAP1 | 60312 | 0.7186386 | 3.9536331 | 1.32E-03 | 9.20E-03 |
| COPG2 | 26958 | 0.7182741 | 4.6478535 | 1.53E-01 | 3.19E-01 |
| DCAF12 | 25853 | 0.7182694 | 5.7695177 | 1.80E-03 | 1.18E-02 |
| TAF5 | 6877 | 0.7182373 | 3.6576269 | 1.32E-02 | 5.57E-02 |
| ZBTB24 | 9841 | 0.7178187 | 3.1348215 | 9.09E-03 | 4.21E-02 |
| ZNF584 | 201514 | 0.717429 | 5.2020392 | 2.24E-04 | 2.17E-03 |
| LRCH3 | 84859 | 0.7173307 | 4.7906229 | 7.18E-03 | 3.53E-02 |
| FAM200A | 221786 | 0.7172368 | 4.75078 | 1.09E-03 | 7.91E-03 |
| NR1D2 | 9975 | 0.7169852 | 4.1891784 | 1.26E-01 | 2.81E-01 |
| MTMR8 | 55613 | 0.7169113 | 2.0324422 | 1.44E-01 | 3.06E-01 |
| SLC15A4 | 121260 | 0.7165226 | 4.8633911 | 5.17E-02 | 1.50E-01 |
| C19orf54 | 284325 | 0.7164496 | 3.0084052 | 4.96E-02 | 1.46E-01 |
| PLCE1 | 51196 | 0.7158951 | 1.6924021 | 2.23E-01 | 4.12E-01 |
| HTR1E | 3354 | 0.7147741 | 1.6282022 | 7.40E-02 | 1.94E-01 |
| PRELID2 | 153768 | 0.7142566 | 0.9229782 | 2.44E-01 | 4.37E-01 |
| ZNF787 | 126208 | 0.7140744 | 0.4375458 | 1.44E-01 | 3.06E-01 |
| RAB18 | 22931 | 0.7138881 | 8.4312385 | 5.90E-05 | 7.30E-04 |
| PANK2 | 80025 | 0.7137562 | 5.9041259 | 1.54E-04 | 1.60E-03 |
| SETD2 | 29072 | 0.713534 | 5.2285679 | 1.31E-04 | 1.40E-03 |
| BUD13 | 84811 | 0.7124878 | 3.6192229 | 9.26E-04 | 6.96E-03 |
| NCK1 | 4690 | 0.7123832 | 5.0297268 | 1.05E-02 | 4.67E-02 |
| TMED7 | 51014 | 0.7116062 | 5.34653 | 6.33E-02 | 1.74E-01 |
| FAM91A1 | 157769 | 0.7114252 | 4.7596563 | 8.71E-02 | 2.16E-01 |
| INTS6L | 203522 | 0.7112666 | 1.9810451 | 1.70E-02 | 6.70E-02 |
| KHNYN | 23351 | 0.7109023 | 1.2104492 | 1.37E-01 | 2.97E-01 |
| GIN1 | 54826 | 0.7103111 | 3.485537 | 2.11E-02 | 7.87E-02 |
| GTF2E1 | 2960 | 0.7100634 | 4.7132952 | 7.00E-04 | 5.58E-03 |
| RALGAPA1 | 253959 | 0.709588 | 5.1731327 | 1.54E-03 | 1.04E-02 |
| ANKMY1 | 51281 | 0.7090452 | 1.4627672 | 4.84E-02 | 1.44E-01 |
| ZSCAN12 | 9753 | 0.7089084 | 3.5086204 | 9.95E-02 | 2.38E-01 |
| VAT1 | 10493 | 0.7088698 | 9.2015302 | 3.51E-03 | 2.01E-02 |
| IFI6 | 2537 | 0.7088296 | 4.7455981 | 2.71E-02 | 9.44E-02 |
| SPIN3 | 169981 | 0.7086957 | 4.6191138 | 6.56E-04 | 5.27E-03 |
| MOSPD2 | 158747 | 0.7081748 | 4.0926509 | 7.34E-04 | 5.78E-03 |
| DAXX | 113523636 | 0.7079921 | 5.0446286 | 7.13E-03 | 3.51E-02 |
| UNC13C | 440279 | 0.7077872 | 1.4204502 | 2.66E-01 | 4.62E-01 |
| PACC1 | 55248 | 0.7072961 | 5.6986028 | 1.60E-04 | 1.65E-03 |
| BAG5 | 9529 | 0.7068446 | 5.6534916 | 1.24E-04 | 1.34E-03 |
| NAV3 | 89795 | 0.7067713 | 5.3571999 | 2.15E-01 | 4.03E-01 |
| RESF1 | 55196 | 0.7065499 | 4.5050831 | 5.46E-03 | 2.88E-02 |
| TRIT1 | 54802 | 0.7065111 | 4.8356695 | 2.21E-04 | 2.16E-03 |
| ARL4A | 10124 | 0.7061891 | 5.7631296 | 1.09E-03 | 7.91E-03 |
| NLGN3 | 54413 | 0.7060811 | 5.7154753 | 1.20E-02 | 5.18E-02 |
| FECH | 2235 | 0.7059483 | 4.8524624 | 2.09E-02 | 7.83E-02 |
| CDC42BPA | 8476 | 0.7056255 | 5.5172333 | 3.00E-04 | 2.77E-03 |
| FAIM | 55179 | 0.7049058 | 4.2171921 | 2.22E-03 | 1.39E-02 |
| LZTFL1 | 54585 | 0.7048772 | 3.7268686 | 3.56E-02 | 1.16E-01 |
| ZKSCAN7 | 55888 | 0.7047289 | 2.7682749 | 3.36E-02 | 1.11E-01 |
| HAPLN1 | 1404 | 0.704703 | -0.069486 | 2.48E-01 | 4.42E-01 |
| EZH2 | 2146 | 0.7046465 | 3.9988234 | 6.12E-03 | 3.15E-02 |
| TRIM27 | 5987 | 0.7045125 | 6.0791894 | 7.88E-05 | 9.24E-04 |
| NIPAL1 | 152519 | 0.7042352 | 1.419197 | 1.62E-01 | 3.31E-01 |
| ZMYND11 | 10771 | 0.7040749 | 6.4256073 | 3.63E-04 | 3.25E-03 |
| MACO1 | 55219 | 0.7039248 | 5.283329 | 1.23E-02 | 5.25E-02 |
| ZNF93 | 81931 | 0.7036067 | 3.3830242 | 7.03E-03 | 3.47E-02 |
| MATR3 | 9782 | 0.7035667 | 8.9934823 | 1.32E-03 | 9.22E-03 |
| MORC3 | 23515 | 0.703181 | 4.8058804 | 6.63E-03 | 3.33E-02 |
| INSIG2 | 51141 | 0.7028536 | 6.1053978 | 1.14E-03 | 8.20E-03 |
| CDKL1 | 8814 | 0.7016887 | 1.5850792 | 1.72E-01 | 3.46E-01 |
| FYTTD1 | 84248 | 0.7011796 | 7.0986457 | 8.69E-05 | 1.00E-03 |
| BUB3 | 9184 | 0.7008902 | 6.86264 | 8.38E-05 | 9.73E-04 |
| GOLGA5 | 9950 | 0.7004991 | 5.2067139 | 2.98E-03 | 1.76E-02 |
| DERA | 51071 | 0.6997888 | 3.3843707 | 4.36E-02 | 1.33E-01 |
| PHC1 | 1911 | 0.6997854 | 3.4083493 | 2.67E-03 | 1.62E-02 |
| ZNF280D | 54816 | 0.6997735 | 2.3166377 | 3.96E-02 | 1.25E-01 |
| TAF4B | 6875 | 0.6996965 | 0.2280312 | 4.34E-01 | 6.30E-01 |
| HYDIN | 54768 | 0.699489 | 2.3526827 | 2.87E-01 | 4.85E-01 |
| RANBP6 | 26953 | 0.6993358 | 6.0808338 | 1.35E-02 | 5.65E-02 |
| SCAPER | 49855 | 0.6988394 | 6.1269912 | 3.32E-02 | 1.10E-01 |
| ZNF155 | 7711 | 0.6987609 | 2.5280287 | 3.30E-02 | 1.09E-01 |
| TRIM37 | 4591 | 0.6983306 | 5.847276 | 3.94E-04 | 3.49E-03 |
| REEP3 | 221035 | 0.6979768 | 4.0529656 | 8.39E-02 | 2.11E-01 |
| IDH1 | 3417 | 0.6979587 | 8.4095191 | 1.48E-04 | 1.55E-03 |
| FHL1 | 2273 | 0.6976398 | 8.5036281 | 6.37E-04 | 5.14E-03 |
| RBBP9 | 10741 | 0.6972287 | 4.5871387 | 1.29E-03 | 9.04E-03 |
| PKIB | 5570 | 0.6971154 | 7.2716672 | 1.54E-02 | 6.24E-02 |
| PCGF1 | 84759 | 0.6969442 | 5.1163178 | 2.21E-03 | 1.39E-02 |
| SART3 | 9733 | 0.6969219 | 4.9786761 | 1.58E-04 | 1.64E-03 |
| BPTF | 2186 | 0.6966156 | 5.9599517 | 7.04E-04 | 5.61E-03 |
| ELMOD2 | 255520 | 0.6963775 | 4.8564686 | 9.77E-03 | 4.43E-02 |
| WDR36 | 134430 | 0.6960699 | 4.2305937 | 1.20E-03 | 8.57E-03 |
| STAMBP | 10617 | 0.6959856 | 5.9595609 | 6.18E-04 | 5.01E-03 |
| WTAP | 9589 | 0.6955778 | 6.926199 | 5.90E-04 | 4.82E-03 |
| ABCE1 | 6059 | 0.695415 | 5.6263891 | 3.53E-03 | 2.02E-02 |
| DDB2 | 1643 | 0.6952607 | 4.5493123 | 5.58E-02 | 1.59E-01 |
| ZNF354C | 30832 | 0.6950457 | 2.4172931 | 2.88E-02 | 9.86E-02 |
| TMPRSS7 | 344805 | 0.6945275 | 2.6352498 | 3.46E-01 | 5.46E-01 |
| ORC3 | 23595 | 0.69451 | 5.9721935 | 1.08E-04 | 1.20E-03 |
| CEP120 | 153241 | 0.6944217 | 3.8520857 | 3.27E-02 | 1.09E-01 |
| CTTNBP2NL | 55917 | 0.6941881 | 5.8399992 | 7.06E-04 | 5.61E-03 |
| SLIT1 | 6585 | 0.6932622 | 5.0924626 | 2.39E-01 | 4.30E-01 |
| NT5C2 | 22978 | 0.6929764 | 5.2294117 | 9.35E-04 | 7.00E-03 |
| DCUN1D4 | 23142 | 0.6928955 | 5.5032324 | 9.13E-05 | 1.05E-03 |
| BBS4 | 585 | 0.6927553 | 6.1607258 | 1.78E-03 | 1.17E-02 |
| CCDC40 | 55036 | 0.6927502 | 2.7993308 | 6.96E-02 | 1.86E-01 |
| PYROXD1 | 79912 | 0.6927475 | 3.9193534 | 2.25E-03 | 1.41E-02 |
| DHCR7 | 1717 | 0.6924281 | 7.6492012 | 3.34E-02 | 1.10E-01 |
| TIGD6 | 81789 | 0.6923062 | 2.8366124 | 6.51E-03 | 3.28E-02 |
| AK9 | 221264 | 0.6921522 | 4.6232696 | 4.00E-03 | 2.23E-02 |
| SMPD2 | 6610 | 0.6921296 | 2.333584 | 7.70E-02 | 1.99E-01 |
| ULK2 | 9706 | 0.6917059 | 5.1763108 | 2.68E-02 | 9.39E-02 |
| PDLIM5 | 10611 | 0.6912611 | 4.4240199 | 6.27E-02 | 1.72E-01 |
| NUP62CL | 54830 | 0.6910522 | 1.5453435 | 1.17E-01 | 2.68E-01 |
| SYNE1 | 23345 | 0.6907647 | 5.5622217 | 1.67E-02 | 6.62E-02 |
| MRPS35 | 60488 | 0.6906639 | 6.8241566 | 1.97E-04 | 1.96E-03 |
| INPP5F | 22876 | 0.6904305 | 6.5918256 | 6.17E-02 | 1.70E-01 |
| PAK5 | 57144 | 0.6901099 | 5.354507 | 4.24E-01 | 6.22E-01 |
| PSD2 | 84249 | 0.6896123 | 4.5154659 | 3.24E-01 | 5.25E-01 |
| UBFD1 | 56061 | 0.6894538 | 5.9147992 | 2.74E-03 | 1.65E-02 |
| DDIAS | 220042 | 0.6893474 | -0.130786 | 3.46E-01 | 5.46E-01 |
| WDR35 | 57539 | 0.6893256 | 3.1743351 | 7.47E-02 | 1.95E-01 |
| GLT1D1 | 144423 | 0.6893078 | 1.8897481 | 1.87E-01 | 3.66E-01 |
| CBX5 | 23468 | 0.6892803 | 7.6639339 | 8.33E-03 | 3.95E-02 |
| REEP1 | 65055 | 0.6884602 | 8.2626754 | 3.22E-01 | 5.23E-01 |
| FGFBP3 | 143282 | 0.6881317 | 5.3311104 | 1.94E-02 | 7.39E-02 |
| LUC7L2 | 51631 | 0.6878906 | 4.7063176 | 4.36E-04 | 3.78E-03 |
| DEPDC1B | 55789 | 0.6876387 | 0.929371 | 8.78E-02 | 2.18E-01 |
| GOLGB1 | 2804 | 0.6876179 | 6.4675325 | 1.48E-02 | 6.03E-02 |
| FAM216A | 29902 | 0.6876049 | 6.1484789 | 3.37E-02 | 1.11E-01 |
| SERAC1 | 84947 | 0.6873251 | 5.1667715 | 1.22E-03 | 8.68E-03 |
| SPAG17 | 200162 | 0.6873123 | 0.2950778 | 3.07E-01 | 5.06E-01 |
| NRG3 | 10718 | 0.6867571 | 3.8185244 | 2.65E-03 | 1.61E-02 |
| TPK1 | 27010 | 0.6862973 | 2.8828586 | 1.05E-01 | 2.48E-01 |
| TOMM70 | 9868 | 0.6860516 | 6.0207253 | 3.22E-04 | 2.93E-03 |
| SCCPDH | 51097 | 0.6860052 | 7.2397013 | 2.68E-04 | 2.52E-03 |
| POLR2B | 5431 | 0.6857196 | 6.9436853 | 1.71E-03 | 1.13E-02 |
| UTP15 | 84135 | 0.6857117 | 3.5254557 | 4.81E-02 | 1.43E-01 |
| STEAP3 | 55240 | 0.6854125 | 2.8936405 | 4.96E-01 | 6.82E-01 |
| SELENOI | 85465 | 0.6851273 | 4.2721787 | 8.94E-03 | 4.16E-02 |
| ERG28 | 11161 | 0.6847407 | 7.9353752 | 9.21E-04 | 6.92E-03 |
| FBXO5 | 26271 | 0.6845234 | 3.2164477 | 3.88E-02 | 1.23E-01 |
| FBXO42 | 54455 | 0.684062 | 5.5032832 | 1.65E-04 | 1.70E-03 |
| ATAD2B | 54454 | 0.6837726 | 3.5053252 | 6.65E-02 | 1.79E-01 |
| ZFAND1 | 79752 | 0.6836531 | 4.8060636 | 1.80E-03 | 1.18E-02 |
| MTSS1 | 9788 | 0.683519 | 6.2113963 | 3.59E-01 | 5.59E-01 |
| CEP95 | 90799 | 0.6831506 | 3.9160374 | 2.26E-02 | 8.30E-02 |
| GCLC | 2729 | 0.6827185 | 3.0327387 | 1.86E-01 | 3.66E-01 |
| DENND1B | 163486 | 0.6822489 | 4.0915648 | 4.89E-03 | 2.63E-02 |
| RAB12 | 201475 | 0.6822056 | 1.6545431 | 7.60E-02 | 1.98E-01 |
| DAPK1 | 1612 | 0.681699 | 4.945682 | 2.94E-03 | 1.74E-02 |
| CDHR3 | 222256 | 0.6808678 | 1.9186364 | 1.29E-01 | 2.86E-01 |
| PLEKHA1 | 59338 | 0.6808399 | 6.826732 | 1.43E-02 | 5.88E-02 |
| USP8 | 9101 | 0.6808233 | 6.1660073 | 1.09E-03 | 7.95E-03 |
| SMARCC1 | 6599 | 0.6805162 | 5.4863803 | 5.27E-03 | 2.80E-02 |
| TBCEL | 219899 | 0.680031 | 2.6084307 | 6.05E-02 | 1.68E-01 |
| ZNF317 | 57693 | 0.6799009 | 4.3600873 | 2.11E-02 | 7.87E-02 |
| ZNF589 | 51385 | 0.6797135 | 3.6557577 | 1.33E-02 | 5.58E-02 |
| SAMD15 | 161394 | 0.6790336 | 2.2228775 | 3.49E-02 | 1.14E-01 |
| SLC17A5 | 26503 | 0.6785366 | 5.2976013 | 3.00E-04 | 2.77E-03 |
| ANXA7 | 310 | 0.6782129 | 6.1043153 | 3.54E-02 | 1.15E-01 |
| MRFAP1L1 | 114932 | 0.67809 | 8.3328883 | 5.43E-03 | 2.87E-02 |
| TMEM164 | 84187 | 0.6778829 | 3.5055675 | 5.26E-03 | 2.80E-02 |
| CCP110 | 9738 | 0.6778719 | 5.5797404 | 4.87E-03 | 2.62E-02 |
| DDTL | 100037417 | 0.6772968 | 0.4090625 | 3.08E-01 | 5.08E-01 |
| ARHGEF10L | 55160 | 0.6771771 | 3.7788374 | 2.02E-03 | 1.29E-02 |
| DNAJA1 | 3301 | 0.676873 | 10.044679 | 1.51E-03 | 1.03E-02 |
| PPCS | 79717 | 0.676763 | 6.7524297 | 1.37E-03 | 9.51E-03 |
| ITPRIP | 85450 | 0.6765027 | 2.941776 | 4.58E-01 | 6.50E-01 |
| BRINP1 | 1620 | 0.6763376 | 6.5095162 | 2.66E-01 | 4.61E-01 |
| KLHL7 | 55975 | 0.6761555 | 7.0159727 | 2.78E-04 | 2.60E-03 |
| TRMT10A | 93587 | 0.6761272 | 3.0571511 | 9.48E-03 | 4.34E-02 |
| SHMT1 | 6470 | 0.6757659 | 3.1468508 | 1.47E-01 | 3.11E-01 |
| IFT88 | 8100 | 0.675692 | 4.717194 | 2.27E-03 | 1.42E-02 |
| IMPA1 | 3612 | 0.6756025 | 5.4975008 | 7.25E-04 | 5.73E-03 |
| EML4 | 27436 | 0.6750484 | 4.4512553 | 4.84E-03 | 2.60E-02 |
| PMAIP1 | 5366 | 0.6748454 | 2.8255311 | 4.71E-01 | 6.60E-01 |
| DPPA4 | 55211 | 0.6748343 | 2.7187307 | 4.32E-01 | 6.28E-01 |
| ZNF354A | 6940 | 0.6747644 | 4.528575 | 1.34E-03 | 9.35E-03 |
| KIF22 | 3835 | 0.6742378 | 4.1512512 | 6.21E-03 | 3.17E-02 |
| NKRF | 55922 | 0.6739798 | 4.0640099 | 4.76E-02 | 1.42E-01 |
| RAB2B | 84932 | 0.6736184 | 5.6200653 | 3.06E-04 | 2.82E-03 |
| ZNF334 | 55713 | 0.6735084 | 3.2676773 | 2.63E-02 | 9.26E-02 |
| CARM1 | 10498 | 0.6731331 | 3.2962882 | 9.13E-03 | 4.22E-02 |
| USP38 | 84640 | 0.6730272 | 3.7286335 | 3.90E-02 | 1.23E-01 |
| SNRNP48 | 154007 | 0.6728401 | 3.1452037 | 3.39E-02 | 1.12E-01 |
| MYO1B | 4430 | 0.6724185 | 4.1752847 | 1.14E-03 | 8.22E-03 |
| PPAT | 5471 | 0.6722294 | 4.7730794 | 1.19E-03 | 8.50E-03 |
| ALKBH1 | 8846 | 0.6721224 | 4.5991622 | 7.88E-04 | 6.12E-03 |
| LTA4H | 4048 | 0.6720613 | 5.6985413 | 1.20E-03 | 8.57E-03 |
| APBA1 | 320 | 0.671817 | 2.5695023 | 5.51E-02 | 1.58E-01 |
| ZW10 | 9183 | 0.6716246 | 3.8301188 | 6.17E-03 | 3.16E-02 |
| SRCAP | 10847 | 0.6715594 | -0.139263 | 2.36E-01 | 4.28E-01 |
| ZNF641 | 121274 | 0.6711672 | 4.3039353 | 5.53E-03 | 2.91E-02 |
| CSGALNACT2 | 55454 | 0.6708422 | 5.0659027 | 1.53E-03 | 1.03E-02 |
| IDI1 | 3422 | 0.6705645 | 9.6952145 | 4.83E-04 | 4.11E-03 |
| HEATR6 | 63897 | 0.6704681 | 4.2871201 | 2.05E-03 | 1.31E-02 |
| ABHD5 | 51099 | 0.6704582 | 4.9799402 | 9.28E-03 | 4.27E-02 |
| PCM1 | 5108 | 0.6699601 | 6.9596727 | 1.88E-03 | 1.22E-02 |
| CCDC181 | 57821 | 0.6696349 | 5.2154548 | 3.38E-02 | 1.11E-01 |
| CLEC18B | 497190 | 0.6695928 | 0.1439746 | 4.33E-01 | 6.28E-01 |
| MINDY3 | 80013 | 0.6695424 | 6.1313699 | 6.77E-04 | 5.40E-03 |
| RNF8 | 9025 | 0.6693629 | 5.9663007 | 7.72E-04 | 6.02E-03 |
| RNF168 | 165918 | 0.6691836 | 4.6705961 | 5.07E-04 | 4.27E-03 |
| ARHGAP42 | 143872 | 0.6690994 | 1.3915171 | 3.36E-01 | 5.37E-01 |
| ACBD5 | 91452 | 0.6690874 | 5.7790574 | 3.12E-03 | 1.83E-02 |
| SUPT20H | 55578 | 0.6685444 | 4.7988998 | 5.79E-03 | 3.02E-02 |
| SHROOM1 | 134549 | 0.668409 | 0.2659621 | 2.77E-01 | 4.74E-01 |
| GPR108 | 56927 | 0.6683787 | 4.875452 | 1.67E-02 | 6.60E-02 |
| TSPAN10 | 83882 | 0.6681871 | 0.2685299 | 2.31E-01 | 4.22E-01 |
| BCL2L12 | 83596 | 0.6677724 | 1.4011854 | 3.70E-01 | 5.69E-01 |
| FAM117B | 150864 | 0.6677433 | 4.0410115 | 1.22E-01 | 2.76E-01 |
| HSPH1 | 10808 | 0.6672926 | 7.9365187 | 1.95E-04 | 1.95E-03 |
| TMEM182 | 130827 | 0.6672714 | 3.5224906 | 3.10E-03 | 1.82E-02 |
| ATG3 | 64422 | 0.6669333 | 6.5441051 | 1.52E-04 | 1.58E-03 |
| SUSD4 | 55061 | 0.6661729 | 5.3893397 | 2.03E-01 | 3.88E-01 |
| EEFSEC | 60678 | 0.6660952 | 4.3390295 | 1.12E-03 | 8.09E-03 |
| ZNF823 | 55552 | 0.6660596 | 3.4755728 | 1.29E-02 | 5.44E-02 |
| NBR1 | 4077 | 0.6656878 | 5.4333402 | 8.15E-02 | 2.07E-01 |
| MAK16 | 84549 | 0.6656374 | 3.9998679 | 1.55E-03 | 1.04E-02 |
| DHX32 | 55760 | 0.6651177 | 4.7210671 | 7.14E-03 | 3.51E-02 |
| C6orf89 | 221477 | 0.6645872 | 6.2378888 | 6.91E-03 | 3.43E-02 |
| GFM2 | 84340 | 0.6641303 | 4.7887578 | 4.82E-02 | 1.43E-01 |
| GRB10 | 2887 | 0.6638513 | 5.294971 | 3.06E-03 | 1.80E-02 |
| MAP3K6 | 9064 | 0.6638181 | 1.3604497 | 1.55E-01 | 3.22E-01 |
| INTS8 | 55656 | 0.6635735 | 4.3715531 | 2.34E-02 | 8.51E-02 |
| TRADD | 8717 | 0.6635499 | 1.8091011 | 1.94E-01 | 3.76E-01 |
| C2orf42 | 54980 | 0.6635157 | 3.4978975 | 7.26E-02 | 1.91E-01 |
| DLG1 | 1739 | 0.6633165 | 4.3542486 | 5.67E-02 | 1.61E-01 |
| PGAP4 | 84302 | 0.6633123 | 8.0058292 | 2.91E-03 | 1.73E-02 |
| CDH3 | 1001 | 0.6630581 | 2.08143 | 2.21E-01 | 4.10E-01 |
| TGDS | 23483 | 0.6627348 | 3.5616023 | 9.32E-02 | 2.27E-01 |
| ZNF331 | 55422 | 0.6625074 | 5.0487109 | 2.03E-03 | 1.30E-02 |
| PIAS1 | 8554 | 0.662503 | 6.8966371 | 8.77E-04 | 6.64E-03 |
| LDAH | 60526 | 0.6623286 | 5.1756013 | 1.64E-03 | 1.10E-02 |
| PRKRA | 8575 | 0.6621256 | 6.8027827 | 8.17E-04 | 6.30E-03 |
| CHUK | 1147 | 0.6620833 | 4.1200684 | 8.25E-02 | 2.09E-01 |
| GNAS | 2778 | 0.6620054 | 10.544456 | 6.45E-03 | 3.27E-02 |
| H3C6 | 8353 | 0.6616746 | 1.8478698 | 3.45E-01 | 5.46E-01 |
| UBE2B | 7320 | 0.661623 | 6.5480261 | 6.23E-03 | 3.18E-02 |
| GNAI3 | 2773 | 0.6615185 | 7.6566122 | 2.16E-04 | 2.11E-03 |
| CARD8 | 22900 | 0.661045 | 3.0520045 | 8.66E-02 | 2.16E-01 |
| C17orf80 | 55028 | 0.6608169 | 3.7771908 | 8.95E-03 | 4.16E-02 |
| ST6GAL1 | 6480 | 0.6608013 | 3.5697572 | 7.56E-02 | 1.97E-01 |
| ZBTB41 | 360023 | 0.6606135 | 4.6789032 | 2.20E-02 | 8.12E-02 |
| GCC1 | 79571 | 0.6601795 | 3.6473883 | 1.28E-01 | 2.84E-01 |
| MAPRE2 | 10982 | 0.6600529 | 7.7410026 | 4.58E-02 | 1.38E-01 |
| TRIQK | 286144 | 0.6598696 | 4.5046664 | 1.75E-03 | 1.16E-02 |
| WIPI2 | 26100 | 0.6594996 | 6.2837505 | 3.14E-02 | 1.05E-01 |
| TMEM63C | 57156 | 0.6594137 | 1.4914979 | 1.52E-01 | 3.17E-01 |
| CD83 | 9308 | 0.659385 | 4.658494 | 1.63E-03 | 1.09E-02 |
| PIK3CA | 5290 | 0.6593761 | 4.5791139 | 2.59E-03 | 1.58E-02 |
| RPS20 | 6224 | 0.6592166 | 7.7098266 | 2.86E-03 | 1.71E-02 |
| UBAP2L | 9898 | 0.6589128 | 6.4027738 | 1.47E-02 | 6.00E-02 |
| ZNF43 | 7594 | 0.6589048 | 4.5623975 | 9.83E-03 | 4.45E-02 |
| GK | 2710 | 0.6587452 | 3.1686869 | 9.25E-03 | 4.26E-02 |
| HSD17B11 | 51170 | 0.6584923 | 3.3725431 | 8.06E-03 | 3.85E-02 |
| PPP4R3A | 55671 | 0.6582776 | 5.4779783 | 1.41E-02 | 5.84E-02 |
| MED20 | 9477 | 0.6579689 | 3.6854249 | 7.21E-02 | 1.91E-01 |
| RLF | 6018 | 0.6578122 | 5.6358817 | 4.28E-04 | 3.73E-03 |
| PRKAR2A | 5576 | 0.657543 | 6.1013436 | 4.14E-04 | 3.65E-03 |
| ALG14 | 199857 | 0.6574048 | 4.5841211 | 4.42E-04 | 3.83E-03 |
| APBB2 | 323 | 0.6572253 | 5.3569749 | 1.89E-03 | 1.23E-02 |
| RETSAT | 54884 | 0.6570153 | 3.9501803 | 1.40E-01 | 3.02E-01 |
| PCMTD2 | 55251 | 0.6567534 | 4.5731913 | 1.68E-03 | 1.12E-02 |
| AP1G2 | 8906 | 0.6565745 | 0.9364825 | 3.60E-01 | 5.60E-01 |
| RPIA | 22934 | 0.6564013 | 3.90626 | 3.14E-02 | 1.05E-01 |
| EIF3L | 51386 | 0.6559885 | 6.7412216 | 2.14E-04 | 2.10E-03 |
| CEP41 | 95681 | 0.6559235 | 6.0968116 | 1.97E-03 | 1.27E-02 |
| LEPR | 3953 | 0.6558861 | 1.7462307 | 1.10E-01 | 2.56E-01 |
| ASXL2 | 55252 | 0.6555922 | 2.7538445 | 3.16E-02 | 1.06E-01 |
| TOM1L1 | 10040 | 0.6553315 | 3.7930369 | 4.42E-02 | 1.35E-01 |
| TRIM26 | 7726 | 0.6553297 | 4.3026782 | 1.20E-02 | 5.16E-02 |
| MIEF1 | 54471 | 0.6543889 | 5.2813045 | 4.65E-03 | 2.52E-02 |
| NAPB | 63908 | 0.6529455 | 7.2097972 | 3.05E-01 | 5.04E-01 |
| CSRNP2 | 81566 | 0.6529197 | 5.1061904 | 4.63E-02 | 1.39E-01 |
| SGSM1 | 129049 | 0.6528505 | 3.6014498 | 4.06E-01 | 6.07E-01 |
| C1GALT1C1 | 29071 | 0.6527416 | 5.4327149 | 8.15E-04 | 6.29E-03 |
| SPECC1 | 92521 | 0.6523439 | 4.5491368 | 4.68E-03 | 2.53E-02 |
| ZSCAN23 | 222696 | 0.6518244 | 0.4033775 | 1.80E-01 | 3.58E-01 |
| CLNS1A | 1207 | 0.6514424 | 6.3767019 | 2.34E-03 | 1.45E-02 |
| SMG5 | 23381 | 0.6512443 | 4.6657009 | 4.92E-02 | 1.45E-01 |
| DKK4 | 27121 | 0.6512026 | -0.324088 | 3.32E-01 | 5.33E-01 |
| STX7 | 8417 | 0.6511029 | 7.4013583 | 3.52E-02 | 1.15E-01 |
| RHOT1 | 55288 | 0.6505868 | 6.4899016 | 4.79E-04 | 4.09E-03 |
| GFRA1 | 2674 | 0.6504541 | 3.5736067 | 1.26E-01 | 2.81E-01 |
| EZH1 | 2145 | 0.6504075 | 4.4247957 | 8.80E-04 | 6.66E-03 |
| DDX19B | 11269 | 0.650298 | 4.4087529 | 6.59E-03 | 3.31E-02 |
| NCAM2 | 4685 | 0.6501134 | 4.5211277 | 1.54E-01 | 3.20E-01 |
| PCDHB15 | 56121 | 0.6500342 | 3.7922342 | 2.20E-02 | 8.14E-02 |
| PDCD2L | 84306 | 0.6497223 | 4.0655816 | 2.27E-02 | 8.31E-02 |
| PSMD14 | 10213 | 0.6494679 | 8.2362832 | 4.60E-04 | 3.95E-03 |
| RAI2 | 10742 | 0.6492247 | 3.9915306 | 5.93E-02 | 1.66E-01 |
| SMG6 | 23293 | 0.6492091 | 3.5320183 | 3.68E-03 | 2.09E-02 |
| DTX3 | 196403 | 0.6489461 | 5.1200967 | 3.08E-02 | 1.04E-01 |
| IFT74 | 80173 | 0.6488614 | 4.7318096 | 2.73E-03 | 1.65E-02 |
| PIK3R4 | 30849 | 0.6485398 | 3.3215082 | 2.01E-02 | 7.61E-02 |
| SMC3 | 9126 | 0.6476786 | 7.5797286 | 1.44E-02 | 5.91E-02 |
| AIFM2 | 84883 | 0.6473915 | 2.9463711 | 1.10E-01 | 2.55E-01 |
| BCL2L11 | 10018 | 0.6472553 | 2.047649 | 1.46E-01 | 3.10E-01 |
| PNPLA8 | 50640 | 0.6471067 | 6.9855427 | 2.47E-04 | 2.35E-03 |
| FAM161A | 84140 | 0.6466686 | 5.4564924 | 1.29E-01 | 2.86E-01 |
| ARL5B | 221079 | 0.6462527 | 4.5136068 | 1.34E-03 | 9.33E-03 |
| SPG11 | 80208 | 0.6462412 | 4.0812732 | 3.87E-02 | 1.22E-01 |
| YTHDF1 | 54915 | 0.6459002 | 5.7028757 | 1.79E-03 | 1.17E-02 |
| LRRC28 | 123355 | 0.645676 | 5.0201322 | 3.28E-03 | 1.90E-02 |
| REXO5 | 81691 | 0.6452691 | 2.6154591 | 2.05E-01 | 3.90E-01 |
| ZBED8 | 63920 | 0.6451229 | 4.3821675 | 2.54E-03 | 1.56E-02 |
| ABLIM1 | 3983 | 0.6449561 | 6.6577341 | 5.80E-02 | 1.63E-01 |
| SNX13 | 23161 | 0.6449295 | 4.4565719 | 6.61E-03 | 3.32E-02 |
| PTK2 | 5747 | 0.6448516 | 5.4941075 | 5.07E-02 | 1.48E-01 |
| ZNF569 | 148266 | 0.6448178 | 4.2642464 | 1.71E-02 | 6.73E-02 |
| TSG101 | 7251 | 0.6448156 | 8.1122538 | 5.83E-04 | 4.78E-03 |
| SLMAP | 7871 | 0.6443375 | 4.5182264 | 1.02E-02 | 4.59E-02 |
| SASH1 | 23328 | 0.6442243 | 2.9004713 | 1.91E-01 | 3.72E-01 |
| GNS | 2799 | 0.6440712 | 6.3866238 | 6.72E-02 | 1.81E-01 |
| GOLPH3 | 64083 | 0.644068 | 6.5430144 | 6.53E-03 | 3.29E-02 |
| AP3S2 | 10239 | 0.6440225 | 3.5849701 | 8.26E-03 | 3.92E-02 |
| SLF2 | 55719 | 0.6439269 | 5.4337419 | 2.32E-03 | 1.44E-02 |
| KLRG1 | 10219 | 0.6430711 | 3.3095604 | 4.79E-02 | 1.42E-01 |
| ZNF768 | 79724 | 0.6428492 | 1.8768536 | 1.17E-01 | 2.67E-01 |
| BNIP3 | 664 | 0.6426069 | 7.3916592 | 4.13E-02 | 1.29E-01 |
| PPFIBP2 | 8495 | 0.6425234 | 0.5798904 | 2.34E-01 | 4.26E-01 |
| SNX10 | 29887 | 0.6424718 | 6.952194 | 2.65E-01 | 4.61E-01 |
| PLCB4 | 5332 | 0.6409764 | 4.7114066 | 1.25E-02 | 5.33E-02 |
| MCFD2 | 90411 | 0.6408832 | 5.9317338 | 4.11E-02 | 1.28E-01 |
| OPTN | 10133 | 0.6400906 | 7.1605996 | 4.80E-04 | 4.09E-03 |
| TPP1 | 1200 | 0.640089 | 5.2071744 | 6.33E-02 | 1.74E-01 |
| GFM1 | 85476 | 0.6400205 | 5.5129276 | 3.06E-04 | 2.82E-03 |
| PRKX | 5613 | 0.6399927 | 5.4168337 | 1.01E-02 | 4.54E-02 |
| FAM222A | 84915 | 0.6392442 | 4.0903328 | 2.40E-01 | 4.31E-01 |
| TAS2R14 | 106707243 | 0.6388647 | 2.0164715 | 6.39E-02 | 1.74E-01 |
| CEP126 | 57562 | 0.6388197 | 3.9539227 | 2.52E-02 | 9.00E-02 |
| MBD2 | 8932 | 0.6378268 | 3.8292509 | 1.37E-01 | 2.97E-01 |
| MAP2K5 | 5607 | 0.636945 | 4.2234722 | 3.05E-03 | 1.80E-02 |
| PARS2 | 25973 | 0.6369089 | 2.7063454 | 1.12E-01 | 2.59E-01 |
| TMEM128 | 85013 | 0.6364751 | 6.4055429 | 3.39E-03 | 1.95E-02 |
| CACNB1 | 782 | 0.6361193 | 5.0355239 | 1.47E-01 | 3.11E-01 |
| NDC1 | 55706 | 0.6359735 | 3.4766998 | 6.52E-02 | 1.77E-01 |
| ASCC1 | 51008 | 0.6357801 | 6.0752796 | 3.17E-04 | 2.90E-03 |
| DNAJC21 | 134218 | 0.6356417 | 5.4484937 | 1.55E-03 | 1.05E-02 |
| DDX1 | 1653 | 0.6354868 | 7.5532553 | 9.16E-04 | 6.89E-03 |
| TCF7L1 | 83439 | 0.6350322 | 1.0767363 | 4.76E-01 | 6.65E-01 |
| ALDOC | 230 | 0.6350061 | 4.7787858 | 3.07E-03 | 1.81E-02 |
| CCDC150 | 284992 | 0.6348759 | 0.1087459 | 3.23E-01 | 5.24E-01 |
| RAB20 | 55647 | 0.6341561 | 1.0930804 | 1.73E-01 | 3.48E-01 |
| ZBTB26 | 57684 | 0.6340096 | 2.3367276 | 1.66E-02 | 6.58E-02 |
| MORC2 | 22880 | 0.6339626 | 4.1856789 | 6.27E-03 | 3.20E-02 |
| CASD1 | 64921 | 0.6338909 | 5.3394784 | 2.25E-03 | 1.41E-02 |
| MPZL1 | 9019 | 0.633514 | 7.5368756 | 5.86E-02 | 1.65E-01 |
| EPM2AIP1 | 9852 | 0.6331345 | 5.8847371 | 9.31E-04 | 6.98E-03 |
| LMO1 | 4004 | 0.6331294 | 3.5929953 | 3.92E-01 | 5.93E-01 |
| MTHFD1 | 4522 | 0.6330908 | 4.9351302 | 6.66E-02 | 1.80E-01 |
| CFAP54 | 144535 | 0.6328142 | 1.5701947 | 2.58E-01 | 4.53E-01 |
| BNIP2 | 663 | 0.63193 | 4.9494023 | 1.30E-02 | 5.48E-02 |
| REPS1 | 85021 | 0.6317639 | 5.7137507 | 3.67E-04 | 3.28E-03 |
| TNKS | 8658 | 0.6317607 | 5.1176165 | 2.90E-03 | 1.73E-02 |
| FAM149A | 25854 | 0.6314085 | 3.7123962 | 1.62E-01 | 3.31E-01 |
| SRC | 6714 | 0.6311853 | 4.2694243 | 3.32E-02 | 1.10E-01 |
| KCNC2 | 3747 | 0.6309934 | 3.1128571 | 4.81E-01 | 6.68E-01 |
| GLUD1 | 2746 | 0.6308721 | 6.4597886 | 1.51E-02 | 6.13E-02 |
| RAB9B | 51209 | 0.6304065 | 6.1599491 | 3.13E-01 | 5.13E-01 |
| SORD | 6652 | 0.6302319 | 3.7919752 | 8.50E-03 | 4.02E-02 |
| PTPN1 | 5770 | 0.6295926 | 5.403218 | 5.54E-04 | 4.58E-03 |
| SMAD4 | 4089 | 0.6294466 | 4.9278001 | 3.65E-02 | 1.18E-01 |
| CBX4 | 8535 | 0.6293368 | 4.0913148 | 1.18E-01 | 2.68E-01 |
| VPS35 | 55737 | 0.6292106 | 8.1294395 | 5.70E-04 | 4.70E-03 |
| GPR135 | 64582 | 0.6291081 | 2.5814395 | 2.81E-02 | 9.70E-02 |
| ARRB1 | 408 | 0.6289709 | 3.0273093 | 1.45E-01 | 3.08E-01 |
| PLAA | 9373 | 0.6287744 | 5.6107309 | 1.91E-03 | 1.24E-02 |
| PSEN1 | 5663 | 0.6286212 | 5.8081203 | 1.97E-02 | 7.48E-02 |
| RPAP1 | 26015 | 0.6284278 | 4.0205366 | 1.16E-02 | 5.06E-02 |
| MPP2 | 4355 | 0.6283254 | 5.4450878 | 6.65E-03 | 3.33E-02 |
| SLC16A2 | 6567 | 0.6282678 | 5.3466848 | 1.94E-02 | 7.40E-02 |
| DYNC1LI1 | 51143 | 0.6281699 | 7.3728592 | 4.67E-02 | 1.40E-01 |
| SH3GLB1 | 51100 | 0.6280003 | 6.7098877 | 6.14E-04 | 4.98E-03 |
| PFKFB4 | 5210 | 0.6279984 | 3.8315941 | 7.36E-02 | 1.93E-01 |
| TMEM196 | 256130 | 0.6277812 | 6.0911109 | 5.30E-01 | 7.09E-01 |
| FTSJ3 | 117246 | 0.6277742 | 4.4490865 | 1.33E-02 | 5.58E-02 |
| RASSF7 | 8045 | 0.6277128 | 3.7909453 | 7.00E-02 | 1.86E-01 |
| TMEM30A | 55754 | 0.6275202 | 6.8741866 | 7.84E-02 | 2.02E-01 |
| GFOD1 | 54438 | 0.6274871 | 4.9813872 | 2.09E-01 | 3.95E-01 |
| PEX3 | 8504 | 0.6270303 | 4.6434575 | 2.12E-02 | 7.88E-02 |
| CIZ1 | 25792 | 0.6267745 | 4.6496748 | 2.21E-02 | 8.15E-02 |
| LIN52 | 91750 | 0.6267518 | 5.0532322 | 6.18E-03 | 3.16E-02 |
| CREB3L4 | 148327 | 0.6266664 | 1.6547775 | 1.40E-01 | 3.01E-01 |
| GCA | 25801 | 0.6266317 | 4.9655121 | 5.45E-03 | 2.88E-02 |
| WARS1 | 7453 | 0.6265499 | 7.1918395 | 2.17E-01 | 4.05E-01 |
| USO1 | 8615 | 0.6264735 | 5.7983663 | 3.21E-02 | 1.07E-01 |
| BAIAP3 | 8938 | 0.6263993 | 4.8880977 | 2.43E-02 | 8.75E-02 |
| TAMM41 | 132001 | 0.626395 | 4.5023829 | 9.63E-03 | 4.38E-02 |
| ACBD3 | 64746 | 0.6263387 | 5.8303304 | 8.10E-04 | 6.26E-03 |
| PLPP6 | 403313 | 0.6259074 | 4.1250148 | 4.19E-02 | 1.30E-01 |
| AASDHPPT | 60496 | 0.6255728 | 7.8814036 | 1.85E-02 | 7.15E-02 |
| SLC1A5 | 6510 | 0.6252861 | 1.8234232 | 3.48E-01 | 5.49E-01 |
| UAP1L1 | 91373 | 0.625094 | 0.7396173 | 2.64E-01 | 4.59E-01 |
| EEA1 | 8411 | 0.6249009 | 4.9749771 | 1.14E-03 | 8.22E-03 |
| NASP | 4678 | 0.624757 | 6.417036 | 2.39E-02 | 8.63E-02 |
| PPP2CA | 5515 | 0.6246964 | 9.0058955 | 3.48E-03 | 2.00E-02 |
| KCTD20 | 222658 | 0.6246587 | 5.5151613 | 4.02E-03 | 2.24E-02 |
| WDR19 | 57728 | 0.6245841 | 3.6517656 | 7.90E-02 | 2.03E-01 |
| THOC5 | 8563 | 0.6237378 | 4.6445739 | 1.76E-03 | 1.16E-02 |
| SAFB | 6294 | 0.6233208 | 6.5277715 | 5.03E-04 | 4.25E-03 |
| ATXN2 | 6311 | 0.6230775 | 5.3886984 | 1.02E-02 | 4.57E-02 |
| SUMF1 | 285362 | 0.622346 | 4.6446304 | 1.94E-01 | 3.76E-01 |
| SUDS3 | 64426 | 0.6222153 | 5.5719746 | 2.09E-02 | 7.83E-02 |
| B4GALT7 | 11285 | 0.6221818 | 4.802636 | 1.21E-01 | 2.73E-01 |
| CMTR1 | 23070 | 0.6217361 | 3.3274116 | 3.56E-02 | 1.16E-01 |
| LRIF1 | 55791 | 0.6217165 | 5.3063149 | 8.11E-03 | 3.87E-02 |
| FBXL22 | 283807 | 0.6216966 | 0.0904257 | 2.65E-01 | 4.60E-01 |
| PLCL2 | 23228 | 0.6216536 | 4.353601 | 8.68E-02 | 2.16E-01 |
| PCDHB5 | 26167 | 0.6216395 | 4.0924735 | 1.50E-02 | 6.10E-02 |
| RTCA | 8634 | 0.6215242 | 6.5851683 | 6.56E-03 | 3.30E-02 |
| WNT3 | 7473 | 0.6214595 | 3.0461563 | 1.94E-02 | 7.40E-02 |
| MDM1 | 56890 | 0.6212295 | 3.6211167 | 2.77E-02 | 9.61E-02 |
| ATG13 | 9776 | 0.6207642 | 5.1844661 | 5.50E-02 | 1.57E-01 |
| LYPLA1 | 10434 | 0.6203241 | 7.0920748 | 1.39E-03 | 9.59E-03 |
| HAUS2 | 55142 | 0.6202738 | 4.9272579 | 5.83E-02 | 1.64E-01 |
| KEAP1 | 9817 | 0.6201729 | 5.8985785 | 8.78E-03 | 4.11E-02 |
| ARHGAP29 | 9411 | 0.619601 | 5.8289757 | 3.24E-01 | 5.25E-01 |
| SEC23IP | 11196 | 0.6193569 | 4.8174009 | 2.31E-02 | 8.41E-02 |
| ZNF681 | 148213 | 0.619281 | 3.1502936 | 4.96E-02 | 1.46E-01 |
| RPL12 | 6136 | 0.6189327 | 6.831935 | 2.14E-03 | 1.35E-02 |
| EDC4 | 23644 | 0.6186862 | 3.5922415 | 2.79E-02 | 9.66E-02 |
| ZFP64 | 55734 | 0.6183977 | 4.4425649 | 1.58E-03 | 1.06E-02 |
| IKZF4 | 64375 | 0.6182967 | 3.349457 | 8.84E-02 | 2.19E-01 |
| MEGF11 | 84465 | 0.6182015 | 3.3386775 | 2.63E-01 | 4.58E-01 |
| ZXDB | 158586 | 0.6181111 | 2.9549225 | 5.80E-02 | 1.63E-01 |
| NBPF8 | 728841 | 0.6180075 | 2.0369659 | 9.74E-02 | 2.35E-01 |
| MAPK1 | 5594 | 0.6178533 | 6.7119503 | 5.36E-03 | 2.84E-02 |
| RABGAP1 | 23637 | 0.6177318 | 5.7166539 | 8.25E-04 | 6.34E-03 |
| CCNB1 | 891 | 0.6175731 | 4.2650782 | 6.57E-03 | 3.30E-02 |
| ZBTB10 | 65986 | 0.6175495 | 3.3486047 | 7.13E-03 | 3.51E-02 |
| TDP2 | 51567 | 0.6169544 | 5.2825701 | 1.01E-03 | 7.43E-03 |
| NFRKB | 4798 | 0.6168186 | 4.2793996 | 1.80E-02 | 6.99E-02 |
| STT3A | 3703 | 0.6167227 | 6.942672 | 6.05E-02 | 1.68E-01 |
| DPF2 | 5977 | 0.6165826 | 6.3527331 | 2.13E-02 | 7.91E-02 |
| KLHL26 | 55295 | 0.6157577 | 3.100914 | 6.00E-02 | 1.67E-01 |
| ZNF747 | 65988 | 0.6156088 | 2.5494354 | 2.35E-02 | 8.52E-02 |
| WAPL | 23063 | 0.6155726 | 5.5093648 | 7.38E-03 | 3.60E-02 |
| FAM174A | 345757 | 0.6154003 | 5.6755616 | 3.40E-03 | 1.96E-02 |
| RNF10 | 9921 | 0.6153651 | 7.0003813 | 1.27E-02 | 5.39E-02 |
| POLR3C | 10623 | 0.6153031 | 5.4519915 | 3.83E-03 | 2.16E-02 |
| HENMT1 | 113802 | 0.6152589 | 5.4063267 | 2.92E-01 | 4.90E-01 |
| UPF3B | 65109 | 0.6152149 | 6.1449504 | 7.46E-02 | 1.95E-01 |
| KLHL8 | 57563 | 0.6146047 | 4.9161165 | 4.26E-02 | 1.31E-01 |
| TRIM24 | 8805 | 0.6145786 | 5.8465912 | 1.74E-02 | 6.83E-02 |
| TXLNG | 55787 | 0.6145492 | 5.209836 | 1.38E-03 | 9.56E-03 |
| H3-5 | 440093 | 0.6141376 | 0.7616037 | 3.05E-01 | 5.04E-01 |
| C2orf15 | 150590 | 0.6140921 | 3.9009524 | 2.58E-01 | 4.53E-01 |
| SENP2 | 59343 | 0.6138726 | 5.0884397 | 1.47E-02 | 6.00E-02 |
| MSH6 | 2956 | 0.6134613 | 5.2385848 | 5.64E-03 | 2.96E-02 |
| CLDN12 | 102723899 | 0.6133054 | 4.9622126 | 7.62E-03 | 3.70E-02 |
| ZNF530 | 348327 | 0.6132733 | 2.6548059 | 2.30E-02 | 8.39E-02 |
| H2AC8 | 3012 | 0.6128597 | 1.6461872 | 1.37E-01 | 2.97E-01 |
| HAP1 | 9001 | 0.6127048 | 3.4766791 | 1.94E-01 | 3.76E-01 |
| GAREM1 | 64762 | 0.6122714 | 3.5035055 | 2.85E-02 | 9.78E-02 |
| POLG2 | 11232 | 0.6122072 | 3.3518057 | 2.51E-02 | 8.98E-02 |
| PLD5 | 200150 | 0.61202 | 2.7278602 | 2.65E-01 | 4.61E-01 |
| FXR2 | 9513 | 0.6117698 | 5.6538162 | 3.24E-02 | 1.08E-01 |
| SYNPR | 132204 | 0.6117404 | 5.2274613 | 5.30E-01 | 7.08E-01 |
| ARFGEF3 | 57221 | 0.611707 | 5.8342343 | 1.52E-01 | 3.18E-01 |
| NBPF3 | 84224 | 0.6115942 | 2.0896174 | 8.93E-02 | 2.20E-01 |
| APOOL | 139322 | 0.6114593 | 4.1498744 | 1.27E-02 | 5.38E-02 |
| PSMD12 | 5718 | 0.6114117 | 7.0099371 | 2.24E-03 | 1.40E-02 |
| FBXO3 | 26273 | 0.6111848 | 6.7328626 | 1.65E-03 | 1.10E-02 |
| AIG1 | 51390 | 0.6104345 | 6.0665605 | 6.20E-04 | 5.01E-03 |
| SRPK1 | 6732 | 0.6100743 | 6.0804977 | 1.83E-02 | 7.09E-02 |
| RAB28 | 9364 | 0.6099965 | 4.7874415 | 2.47E-03 | 1.52E-02 |
| PDIA5 | 10954 | 0.6099092 | 2.7638103 | 8.82E-02 | 2.18E-01 |
| LONRF1 | 91694 | 0.6098868 | 4.9402524 | 3.15E-02 | 1.05E-01 |
| TMEM225B | 100289187 | 0.6094705 | 0.3718956 | 2.37E-01 | 4.29E-01 |
| PSMD1 | 5707 | 0.609206 | 7.9737261 | 3.99E-03 | 2.23E-02 |
| POLK | 51426 | 0.6091035 | 4.5007707 | 7.07E-03 | 3.49E-02 |
| NCAPH | 23397 | 0.6088018 | 1.5879259 | 1.84E-01 | 3.63E-01 |
| CLK4 | 57396 | 0.6086611 | 4.7765892 | 1.23E-02 | 5.28E-02 |
| FUT11 | 170384 | 0.608532 | 4.0918832 | 6.82E-02 | 1.83E-01 |
| SNRPA1 | 6627 | 0.6077634 | 7.0223936 | 2.03E-03 | 1.30E-02 |
| REEP5 | 7905 | 0.6077476 | 8.645163 | 9.16E-04 | 6.89E-03 |
| PLRG1 | 5356 | 0.6076403 | 6.2396509 | 1.02E-02 | 4.59E-02 |
| ARMC1 | 55156 | 0.6076383 | 6.569047 | 2.04E-03 | 1.30E-02 |
| SCFD1 | 23256 | 0.6073892 | 6.8507773 | 1.47E-03 | 1.00E-02 |
| MSH3 | 4437 | 0.6071352 | 3.8125757 | 1.24E-02 | 5.28E-02 |
| TCOF1 | 6949 | 0.6066138 | 4.0953499 | 3.51E-02 | 1.15E-01 |
| AMZ2 | 51321 | 0.6065212 | 7.4189472 | 1.06E-03 | 7.79E-03 |
| PARN | 5073 | 0.6062965 | 4.8588753 | 4.67E-02 | 1.40E-01 |
| PYGO2 | 90780 | 0.6060538 | 4.2528886 | 1.46E-02 | 5.99E-02 |
| MANEA | 79694 | 0.6057203 | 3.4465021 | 1.20E-02 | 5.19E-02 |
| SMARCB1 | 6598 | 0.6055874 | 5.0415992 | 3.06E-02 | 1.03E-01 |
| CENPVL2 | 441495 | 0.6055067 | 0.3149533 | 2.53E-01 | 4.47E-01 |
| ZNF420 | 147923 | 0.6052077 | 4.5739385 | 3.63E-02 | 1.17E-01 |
| ABCD3 | 5825 | 0.60516 | 5.8146978 | 4.35E-03 | 2.38E-02 |
| EIF4ENIF1 | 56478 | 0.6049297 | 4.4350363 | 4.24E-03 | 2.34E-02 |
| GTPBP4 | 23560 | 0.6045043 | 6.6043249 | 4.92E-03 | 2.64E-02 |
| GPAM | 57678 | 0.6041208 | 3.2538381 | 1.81E-01 | 3.59E-01 |
| FBXO28 | 23219 | 0.6038127 | 4.2221193 | 7.78E-02 | 2.01E-01 |
| G6PC3 | 92579 | 0.6033028 | 7.1454909 | 3.53E-02 | 1.15E-01 |
| COP1 | 64326 | 0.6030972 | 6.077734 | 1.91E-03 | 1.24E-02 |
| TAPT1 | 202018 | 0.6030568 | 4.7214741 | 6.81E-03 | 3.39E-02 |
| CCT8 | 10694 | 0.6025257 | 8.2875723 | 9.92E-04 | 7.34E-03 |
| THBS4 | 7060 | 0.6024881 | 1.0040549 | 8.22E-02 | 2.08E-01 |
| TINF2 | 26277 | 0.6021068 | 6.0735118 | 9.28E-04 | 6.96E-03 |
| SYN2 | 6854 | 0.6019326 | 5.05079 | 4.62E-01 | 6.53E-01 |
| EML6 | 400954 | 0.6018403 | 2.4567505 | 1.24E-01 | 2.78E-01 |
| TBC1D14 | 57533 | 0.6017836 | 4.0193779 | 1.92E-02 | 7.34E-02 |
| NCKAP5 | 344148 | 0.6009581 | 2.181427 | 2.06E-01 | 3.92E-01 |
| RASSF8 | 11228 | 0.6006729 | 4.1884736 | 2.95E-01 | 4.93E-01 |
| ZNF318 | 24149 | 0.5996728 | 3.6040592 | 3.81E-03 | 2.15E-02 |
| ACTN1 | 87 | 0.5995882 | 6.2134412 | 3.22E-01 | 5.22E-01 |
| ZNF37A | 7587 | 0.5993168 | 4.692252 | 6.36E-02 | 1.74E-01 |
| C19orf12 | 83636 | 0.5992355 | 5.8620338 | 5.73E-04 | 4.71E-03 |
| TRO | 7216 | 0.5990726 | 6.7769391 | 1.11E-03 | 8.07E-03 |
| STAG2 | 10735 | 0.598988 | 4.9912453 | 1.78E-03 | 1.17E-02 |
| UBE2E1 | 7324 | 0.5987317 | 2.1189823 | 1.43E-01 | 3.06E-01 |
| HIVEP1 | 3096 | 0.5986807 | 3.6626527 | 4.42E-02 | 1.35E-01 |
| VPS26A | 9559 | 0.5985215 | 6.8585837 | 1.09E-03 | 7.92E-03 |
| AJAP1 | 55966 | 0.5982973 | 6.0429221 | 1.76E-01 | 3.52E-01 |
| CPNE4 | 131034 | 0.5982676 | 5.0039039 | 4.60E-02 | 1.39E-01 |
| MTMR6 | 9107 | 0.5981189 | 5.8560595 | 4.55E-04 | 3.91E-03 |
| HNRNPU | 3192 | 0.5980934 | 8.2216738 | 9.92E-04 | 7.34E-03 |
| PCBD1 | 5092 | 0.5978643 | 6.3853539 | 1.31E-02 | 5.53E-02 |
| PEX12 | 5193 | 0.5977211 | 3.2866869 | 1.19E-01 | 2.70E-01 |
| CCNO | 10309 | 0.5976958 | -0.196651 | 4.44E-01 | 6.38E-01 |
| IQCG | 84223 | 0.5974887 | 5.0618194 | 7.81E-03 | 3.77E-02 |
| ZNF134 | 7693 | 0.5973719 | 4.858047 | 4.83E-02 | 1.43E-01 |
| PUDP | 8226 | 0.5970732 | 6.2014375 | 2.02E-03 | 1.29E-02 |
| FTO | 79068 | 0.5970152 | 6.3749181 | 3.21E-03 | 1.87E-02 |
| CRBN | 51185 | 0.5967624 | 6.6738714 | 5.12E-03 | 2.74E-02 |
| RWDD4 | 201965 | 0.596716 | 5.397461 | 3.16E-03 | 1.84E-02 |
| NGB | 58157 | 0.5963677 | 1.6668495 | 5.21E-01 | 7.02E-01 |
| GFRA2 | 2675 | 0.5958705 | 4.4894249 | 3.25E-01 | 5.25E-01 |
| DDX10 | 1662 | 0.5957105 | 4.9837515 | 9.97E-03 | 4.50E-02 |
| FOXJ2 | 55810 | 0.5954345 | 3.8118038 | 1.23E-01 | 2.76E-01 |
| PITPNC1 | 26207 | 0.5954177 | 5.3512019 | 2.05E-03 | 1.31E-02 |
| NAP1L2 | 4674 | 0.5947062 | 5.2998117 | 1.57E-01 | 3.25E-01 |
| ZBED5 | 58486 | 0.5943559 | 6.0755801 | 5.83E-03 | 3.03E-02 |
| ZCWPW1 | 55063 | 0.5940404 | 1.6136049 | 1.12E-01 | 2.59E-01 |
| CHMP7 | 91782 | 0.5937618 | 5.4827206 | 7.04E-03 | 3.48E-02 |
| POLR2M | 81488 | 0.5937018 | 4.4948814 | 3.09E-03 | 1.81E-02 |
| TAF12 | 6883 | 0.593644 | 6.1903934 | 5.33E-03 | 2.83E-02 |
| GJA1 | 2697 | 0.5934183 | 5.7605451 | 4.00E-01 | 6.01E-01 |
| RUFY1 | 80230 | 0.592949 | 4.9361254 | 1.17E-02 | 5.10E-02 |
| CISD2 | 493856 | 0.5927595 | 7.64276 | 1.85E-02 | 7.14E-02 |
| ACP1 | 52 | 0.5926153 | 8.0071946 | 4.58E-03 | 2.48E-02 |
| PRUNE1 | 58497 | 0.5922323 | 5.9881079 | 5.11E-03 | 2.73E-02 |
| FHOD3 | 80206 | 0.592188 | 3.4243703 | 5.05E-02 | 1.48E-01 |
| ZNF620 | 253639 | 0.5921465 | 4.4567301 | 6.51E-02 | 1.76E-01 |
| HLA-DMB | 3109 | 0.5919332 | 1.7007358 | 3.86E-01 | 5.87E-01 |
| RHOD | 29984 | 0.5909411 | 0.5744227 | 4.46E-01 | 6.39E-01 |
| LBR | 3930 | 0.590921 | 5.4367117 | 7.27E-04 | 5.74E-03 |
| FUBP1 | 8880 | 0.5906244 | 5.7593883 | 3.78E-03 | 2.13E-02 |
| SMAD5 | 4090 | 0.5904488 | 4.9285289 | 5.63E-02 | 1.60E-01 |
| AGPAT5 | 55326 | 0.5903765 | 6.1327338 | 6.22E-02 | 1.71E-01 |
| KLF7 | 8609 | 0.5903111 | 6.5206197 | 1.74E-01 | 3.49E-01 |
| AHNAK2 | 113146 | 0.5898753 | 3.0333976 | 4.47E-01 | 6.40E-01 |
| NME9 | 347736 | 0.5898488 | 1.7454937 | 1.94E-01 | 3.76E-01 |
| SMCR8 | 140775 | 0.5897356 | 1.0401072 | 8.63E-02 | 2.15E-01 |
| BBS2 | 583 | 0.589603 | 4.0439569 | 1.04E-02 | 4.64E-02 |
| BCL2L13 | 23786 | 0.5894686 | 5.8235688 | 6.13E-02 | 1.70E-01 |
| GAPVD1 | 26130 | 0.5894175 | 5.8316713 | 7.88E-03 | 3.80E-02 |
| JAKMIP1 | 152789 | 0.589411 | 2.0967682 | 4.86E-01 | 6.73E-01 |
| COLEC12 | 81035 | 0.5892424 | 3.1937116 | 3.53E-01 | 5.52E-01 |
| CTNNA1 | 1495 | 0.5891128 | 7.3203335 | 2.01E-01 | 3.85E-01 |
| KDM1A | 23028 | 0.5891055 | 7.3492468 | 1.46E-03 | 1.00E-02 |
| CDK5R2 | 8941 | 0.5887871 | 5.1216925 | 3.98E-01 | 5.99E-01 |
| PTPRA | 5786 | 0.5881715 | 6.6910363 | 5.52E-03 | 2.90E-02 |
| CDO1 | 1036 | 0.5880711 | 7.4604845 | 2.59E-03 | 1.58E-02 |
| BTRC | 8945 | 0.5871045 | 5.2225733 | 9.16E-03 | 4.23E-02 |
| EHBP1 | 23301 | 0.5866433 | 4.8840027 | 1.57E-02 | 6.32E-02 |
| DCAF16 | 54876 | 0.5865251 | 4.853942 | 1.34E-02 | 5.62E-02 |
| TTLL1 | 25809 | 0.5863602 | 5.5959953 | 9.29E-03 | 4.27E-02 |
| DHX38 | 9785 | 0.5863423 | 3.9623173 | 4.55E-02 | 1.38E-01 |
| DPH5 | 51611 | 0.5863393 | 4.9690693 | 3.72E-03 | 2.11E-02 |
| KIF13B | 23303 | 0.5862769 | 1.9702152 | 1.64E-01 | 3.36E-01 |
| RBM8A | 9939 | 0.5862546 | 5.4084047 | 2.82E-03 | 1.69E-02 |
| IPP | 3652 | 0.5861969 | 4.0665909 | 2.75E-02 | 9.55E-02 |
| TFG | 10342 | 0.5858806 | 7.8025922 | 3.49E-02 | 1.14E-01 |
| ERMAP | 114625 | 0.5856882 | 0.5676106 | 3.55E-01 | 5.54E-01 |
| RNF139 | 11236 | 0.5853556 | 5.6945124 | 5.44E-03 | 2.88E-02 |
| LRRTM4 | 80059 | 0.5851781 | 3.5853269 | 2.75E-01 | 4.71E-01 |
| ARL1 | 400 | 0.5850598 | 6.1613244 | 4.82E-02 | 1.43E-01 |
| DNAJB12 | 54788 | 0.5844453 | 4.9584758 | 3.07E-02 | 1.04E-01 |
| METTL5 | 29081 | 0.5842624 | 7.4671202 | 4.21E-02 | 1.31E-01 |
| VPS8 | 23355 | 0.5840034 | 4.8138644 | 4.93E-03 | 2.65E-02 |
| PDSS2 | 57107 | 0.5833924 | 4.0063844 | 5.11E-02 | 1.49E-01 |
| ZNF713 | 349075 | 0.583316 | 2.5459387 | 9.16E-02 | 2.24E-01 |
| ERLIN1 | 10613 | 0.583099 | 3.8056769 | 2.06E-01 | 3.92E-01 |
| SURF1 | 6834 | 0.5823454 | 5.8126321 | 1.14E-03 | 8.23E-03 |
| CDS2 | 8760 | 0.5820096 | 6.444485 | 1.34E-03 | 9.32E-03 |
| CYP2C8 | 1558 | 0.5817537 | 0.9833707 | 4.25E-01 | 6.23E-01 |
| ST8SIA2 | 8128 | 0.5814232 | 6.7849474 | 1.51E-01 | 3.16E-01 |
| CLDND1 | 56650 | 0.5813809 | 6.8671851 | 3.75E-03 | 2.12E-02 |
| RBM18 | 92400 | 0.5813379 | 6.1743828 | 1.27E-03 | 8.95E-03 |
| MFSD14A | 64645 | 0.5811948 | 2.5677581 | 2.29E-02 | 8.37E-02 |
| NBPF9 | 400818 | 0.5806303 | 3.2248239 | 1.95E-02 | 7.42E-02 |
| THAP6 | 152815 | 0.5805898 | 3.9259202 | 1.40E-02 | 5.80E-02 |
| MPZL3 | 196264 | 0.5804097 | 1.9019161 | 1.58E-01 | 3.25E-01 |
| DLAT | 1737 | 0.5803542 | 5.7124348 | 1.95E-03 | 1.26E-02 |
| ATP6V1C1 | 528 | 0.5799232 | 6.8903354 | 6.94E-03 | 3.44E-02 |
| ERRFI1 | 54206 | 0.5797263 | 5.2440795 | 7.34E-03 | 3.60E-02 |
| NOL11 | 25926 | 0.5795523 | 5.8785071 | 9.92E-04 | 7.34E-03 |
| ZNF675 | 171392 | 0.5793232 | 4.7648827 | 1.74E-01 | 3.50E-01 |
| GPBP1L1 | 60313 | 0.5793001 | 4.2902712 | 6.47E-02 | 1.76E-01 |
| FGFR1 | 2260 | 0.5790328 | 5.889825 | 3.06E-01 | 5.05E-01 |
| ZNF763 | 284390 | 0.5789978 | 0.3112829 | 3.17E-01 | 5.17E-01 |
| TTC4 | 7268 | 0.5782496 | 0.6922009 | 1.82E-01 | 3.60E-01 |
| ATAD1 | 84896 | 0.5780569 | 7.6025013 | 2.38E-03 | 1.47E-02 |
| SPPL3 | 121665 | 0.5778688 | 6.3747552 | 4.27E-03 | 2.35E-02 |
| SHOC2 | 8036 | 0.5777176 | 6.3706102 | 1.32E-03 | 9.20E-03 |
| UBAP1 | 51271 | 0.5776278 | 5.1717061 | 2.02E-02 | 7.62E-02 |
| RPL5 | 6125 | 0.5770994 | 9.0807137 | 3.24E-03 | 1.88E-02 |
| FBXO33 | 254170 | 0.5763243 | 4.4413685 | 7.42E-03 | 3.62E-02 |
| NSUN2 | 54888 | 0.5760832 | 4.9863072 | 1.36E-01 | 2.95E-01 |
| PRPSAP2 | 5636 | 0.5760238 | 5.4342523 | 3.11E-03 | 1.82E-02 |
| SAMD5 | 389432 | 0.5759536 | 2.6791286 | 1.05E-01 | 2.48E-01 |
| ZFP36L2 | 678 | 0.5755605 | 2.4191213 | 4.84E-01 | 6.72E-01 |
| NOL10 | 79954 | 0.5755499 | 4.4879328 | 7.76E-02 | 2.00E-01 |
| LRRC40 | 55631 | 0.5752858 | 6.3731036 | 6.89E-02 | 1.84E-01 |
| ZNF585A | 199704 | 0.575054 | 4.1435961 | 6.95E-03 | 3.45E-02 |
| TPMT | 7172 | 0.5748839 | 4.8753387 | 4.41E-02 | 1.35E-01 |
| RNF152 | 220441 | 0.5746012 | 3.2182375 | 3.93E-01 | 5.93E-01 |
| TTC38 | 55020 | 0.5745006 | 2.915898 | 1.19E-01 | 2.70E-01 |
| CTH | 1491 | 0.5742823 | 3.4793369 | 1.87E-01 | 3.67E-01 |
| CAPZA1 | 829 | 0.573907 | 7.489672 | 3.70E-03 | 2.10E-02 |
| ZNF540 | 163255 | 0.5738212 | 4.5828583 | 4.92E-01 | 6.79E-01 |
| LRRC42 | 115353 | 0.5737996 | 5.1741775 | 3.63E-02 | 1.17E-01 |
| DSN1 | 79980 | 0.5737986 | 2.5328605 | 1.66E-01 | 3.39E-01 |
| ZC3H7A | 29066 | 0.5736698 | 5.5594963 | 4.05E-02 | 1.27E-01 |
| RAB32 | 10981 | 0.5734687 | 5.2650019 | 7.74E-02 | 2.00E-01 |
| ATP11A | 23250 | 0.572783 | 3.2405726 | 1.54E-02 | 6.25E-02 |
| ANKLE2 | 23141 | 0.5727224 | 5.213408 | 9.07E-03 | 4.20E-02 |
| UBE2C | 11065 | 0.5717244 | 3.4171358 | 3.36E-01 | 5.37E-01 |
| POLR3F | 10621 | 0.5713607 | 4.7281474 | 4.82E-03 | 2.60E-02 |
| AP3S1 | 1176 | 0.5709377 | 6.7672398 | 2.67E-03 | 1.62E-02 |
| CWC27 | 10283 | 0.5702677 | 6.3695443 | 7.58E-03 | 3.68E-02 |
| GSPT2 | 23708 | 0.5702582 | 5.9679867 | 2.38E-03 | 1.47E-02 |
| ALKBH6 | 84964 | 0.5701038 | 0.4273582 | 2.46E-01 | 4.39E-01 |
| SEC23B | 10483 | 0.5698759 | 6.5682796 | 1.75E-02 | 6.85E-02 |
| EIF4A3 | 9775 | 0.5697197 | 7.5217865 | 1.23E-03 | 8.74E-03 |
| EIF4G3 | 8672 | 0.569568 | 5.8483782 | 1.92E-03 | 1.24E-02 |
| SESTD1 | 91404 | 0.5694732 | 6.5168513 | 1.65E-01 | 3.36E-01 |
| LGALSL | 29094 | 0.5692442 | 5.5412123 | 8.48E-02 | 2.13E-01 |
| ANKRD50 | 57182 | 0.5691356 | 4.8933466 | 1.52E-01 | 3.17E-01 |
| SMIM13 | 221710 | 0.5691087 | 4.4801118 | 3.91E-02 | 1.24E-01 |
| RPF2 | 84154 | 0.5687712 | 5.5584937 | 5.32E-03 | 2.82E-02 |
| ZNF572 | 137209 | 0.5683399 | 0.5686516 | 2.40E-01 | 4.32E-01 |
| STRAP | 11171 | 0.5682519 | 8.1108428 | 8.80E-03 | 4.11E-02 |
| OXSR1 | 9943 | 0.5682445 | 5.3388047 | 9.19E-03 | 4.23E-02 |
| AP3M1 | 26985 | 0.5678271 | 5.3479813 | 2.02E-02 | 7.62E-02 |
| ANAPC7 | 51434 | 0.567558 | 5.3691015 | 3.09E-03 | 1.82E-02 |
| SLC39A9 | 55334 | 0.5672582 | 5.0772807 | 4.25E-02 | 1.31E-01 |
| ZIK1 | 284307 | 0.5672467 | 3.8908411 | 2.35E-02 | 8.53E-02 |
| ZNF30 | 90075 | 0.5671002 | 4.1493155 | 7.78E-02 | 2.01E-01 |
| RABEP1 | 9135 | 0.5669692 | 6.906799 | 1.92E-02 | 7.34E-02 |
| ANXA2 | 302 | 0.5667519 | 8.8434993 | 2.97E-01 | 4.96E-01 |
| MVD | 4597 | 0.5667507 | 7.9505941 | 2.26E-03 | 1.41E-02 |
| AKAP6 | 9472 | 0.5666952 | 5.3143297 | 5.00E-02 | 1.46E-01 |
| CPT2 | 1376 | 0.5665951 | 3.069101 | 6.39E-02 | 1.74E-01 |
| SPIDR | 23514 | 0.5662813 | 4.4576214 | 1.99E-02 | 7.56E-02 |
| RARS1 | 5917 | 0.5660286 | 6.9583912 | 7.70E-03 | 3.73E-02 |
| C17orf75 | 64149 | 0.5658295 | 6.115294 | 2.94E-02 | 1.00E-01 |
| ARMCX4 | 100131755 | 0.5656904 | 3.4907084 | 1.19E-01 | 2.71E-01 |
| FRMD6 | 122786 | 0.5656084 | 1.4226945 | 5.25E-01 | 7.05E-01 |
| CCDC171 | 203238 | 0.5653454 | 3.810733 | 3.24E-01 | 5.25E-01 |
| DYRK1B | 9149 | 0.5651612 | 2.9078123 | 8.19E-02 | 2.08E-01 |
| RNF144A | 9781 | 0.565121 | 4.0955134 | 3.49E-01 | 5.50E-01 |
| TLCD3A | 79850 | 0.5650863 | 3.4564169 | 1.78E-01 | 3.55E-01 |
| PPP1R2 | 5504 | 0.5649065 | 6.9369442 | 6.33E-03 | 3.22E-02 |
| MTHFSD | 64779 | 0.5648813 | 3.2127085 | 4.20E-02 | 1.30E-01 |
| LRRC37B | 114659 | 0.5648452 | 3.2370804 | 1.14E-01 | 2.63E-01 |
| PTPN11 | 5781 | 0.564811 | 7.553082 | 1.21E-02 | 5.21E-02 |
| PPFIA1 | 8500 | 0.5645966 | 4.7379599 | 1.50E-02 | 6.10E-02 |
| MAP2K6 | 5608 | 0.5645817 | 4.4004956 | 1.47E-02 | 6.00E-02 |
| CKS2 | 1164 | 0.5645592 | 5.2809705 | 2.01E-02 | 7.61E-02 |
| CAPRIN1 | 4076 | 0.5637276 | 7.4317232 | 5.85E-03 | 3.04E-02 |
| UACA | 55075 | 0.5634516 | 3.4137998 | 3.33E-01 | 5.34E-01 |
| COL9A2 | 1298 | 0.5634188 | 2.121695 | 2.18E-01 | 4.07E-01 |
| TAF1A | 9015 | 0.5633996 | 4.0652454 | 7.59E-02 | 1.97E-01 |
| TNFAIP8 | 25816 | 0.5631974 | 1.0504006 | 4.36E-01 | 6.32E-01 |
| ZNF585B | 92285 | 0.5628855 | 2.9081851 | 7.32E-02 | 1.92E-01 |
| SLC17A7 | 57030 | 0.5624612 | 0.930486 | 4.28E-01 | 6.26E-01 |
| GNA12 | 2768 | 0.5615314 | 5.7531837 | 1.50E-01 | 3.15E-01 |
| SRP9 | 6726 | 0.561449 | 9.8816658 | 4.83E-03 | 2.60E-02 |
| AKAP14 | 158798 | 0.5612776 | 2.2464045 | 1.02E-01 | 2.42E-01 |
| CUL3 | 8452 | 0.5611698 | 5.8558763 | 2.75E-02 | 9.55E-02 |
| SEPTIN7 | 989 | 0.5607905 | 9.2018031 | 2.36E-02 | 8.54E-02 |
| PDP2 | 57546 | 0.5606717 | 3.6911087 | 1.94E-02 | 7.40E-02 |
| SNX11 | 29916 | 0.5604632 | 4.8411306 | 1.86E-02 | 7.16E-02 |
| TYW5 | 129450 | 0.5603924 | 3.8052838 | 1.89E-02 | 7.28E-02 |
| OSTM1 | 28962 | 0.5602055 | 6.1497346 | 7.40E-02 | 1.94E-01 |
| C1GALT1 | 56913 | 0.5600699 | 4.957909 | 2.75E-02 | 9.55E-02 |
| FYN | 2534 | 0.5600158 | 8.0788032 | 9.83E-03 | 4.45E-02 |
| C12orf60 | 144608 | 0.5599886 | 1.9340615 | 1.86E-01 | 3.65E-01 |
| ZDHHC22 | 283576 | 0.5599576 | 3.5568463 | 4.81E-02 | 1.43E-01 |
| COX16 | 51241 | 0.559918 | 4.2756001 | 1.38E-02 | 5.74E-02 |
| ZNF616 | 90317 | 0.5598591 | 3.4450811 | 3.67E-02 | 1.18E-01 |
| SPDL1 | 54908 | 0.5598491 | 4.0528813 | 5.51E-02 | 1.58E-01 |
| COG5 | 10466 | 0.5595631 | 4.3077776 | 7.46E-02 | 1.95E-01 |
| ESYT1 | 23344 | 0.5594855 | 4.3910665 | 1.97E-01 | 3.80E-01 |
| CYB5R4 | 51167 | 0.559417 | 4.2740535 | 8.68E-03 | 4.07E-02 |
| DSCC1 | 79075 | 0.5593107 | 1.0878604 | 3.00E-01 | 4.99E-01 |
| ZNF598 | 90850 | 0.5591796 | 0.9036414 | 1.75E-01 | 3.50E-01 |
| MCM5 | 4174 | 0.5591514 | 2.0591601 | 2.57E-01 | 4.52E-01 |
| MCM7 | 4176 | 0.5591071 | 4.6483654 | 1.41E-02 | 5.84E-02 |
| CYP4A11 | 1579 | 0.5584757 | 0.7490445 | 2.84E-01 | 4.83E-01 |
| MORN4 | 118812 | 0.557798 | 6.8242293 | 1.21E-02 | 5.20E-02 |
| KSR2 | 283455 | 0.5575678 | 5.0138995 | 4.73E-01 | 6.62E-01 |
| NCOA7 | 135112 | 0.5574181 | 5.4907323 | 2.26E-01 | 4.16E-01 |
| GPRASP1 | 9737 | 0.557347 | 4.8673166 | 3.31E-01 | 5.32E-01 |
| UBR1 | 197131 | 0.5568311 | 5.3971785 | 1.61E-02 | 6.42E-02 |
| APAF1 | 317 | 0.5567524 | 3.9862775 | 8.53E-02 | 2.14E-01 |
| HARS2 | 23438 | 0.5563461 | 5.1100693 | 7.94E-03 | 3.81E-02 |
| SPIN4 | 139886 | 0.5561401 | 3.3608958 | 1.81E-01 | 3.59E-01 |
| CITED4 | 163732 | 0.5556898 | 4.3993166 | 2.15E-02 | 7.99E-02 |
| ALG11 | 440138 | 0.5543929 | 4.1218189 | 4.98E-02 | 1.46E-01 |
| PPP1R11 | 6992 | 0.5540043 | 6.8330735 | 2.90E-03 | 1.72E-02 |
| SMARCA1 | 6594 | 0.5538763 | 7.0000538 | 9.17E-03 | 4.23E-02 |
| AKAP8L | 26993 | 0.5536856 | 6.4824087 | 7.48E-03 | 3.64E-02 |
| SEL1L3 | 23231 | 0.5536675 | 4.3727448 | 1.47E-01 | 3.11E-01 |
| DKC1 | 1736 | 0.5527852 | 5.6312276 | 3.39E-03 | 1.95E-02 |
| GPR137C | 283554 | 0.5527383 | 3.7596475 | 4.22E-01 | 6.20E-01 |
| BEND3 | 57673 | 0.5521675 | 1.0158498 | 1.43E-01 | 3.05E-01 |
| TCF12 | 6938 | 0.5520553 | 5.3965915 | 3.00E-02 | 1.02E-01 |
| WDR11 | 55717 | 0.5519913 | 4.3022767 | 8.34E-02 | 2.10E-01 |
| ZER1 | 10444 | 0.5517084 | 4.9454509 | 5.62E-02 | 1.60E-01 |
| RAB40A | 142684 | 0.5516589 | 0.8960275 | 2.47E-01 | 4.40E-01 |
| AHCYL2 | 23382 | 0.551627 | 4.0448315 | 2.02E-02 | 7.62E-02 |
| PIGK | 10026 | 0.551326 | 5.12544 | 3.20E-02 | 1.07E-01 |
| NEXMIF | 340533 | 0.5511886 | 4.6787469 | 2.53E-01 | 4.47E-01 |
| SALL2 | 6297 | 0.551175 | 3.7172714 | 3.77E-02 | 1.20E-01 |
| TFCP2 | 7024 | 0.5508348 | 4.8388144 | 3.57E-03 | 2.04E-02 |
| STK39 | 27347 | 0.5504501 | 3.8250409 | 1.85E-02 | 7.15E-02 |
| VAPB | 9217 | 0.5502744 | 5.3308149 | 4.75E-02 | 1.42E-01 |
| PEX19 | 5824 | 0.5499939 | 5.1259047 | 4.27E-02 | 1.32E-01 |
| GDI2 | 2665 | 0.5499938 | 8.3665855 | 3.15E-03 | 1.84E-02 |
| GLO1 | 2739 | 0.5497268 | 8.3073951 | 2.11E-02 | 7.87E-02 |
| BLOC1S2 | 282991 | 0.5496546 | 7.9683785 | 6.42E-03 | 3.25E-02 |
| SUPT5H | 6829 | 0.5490069 | 5.94023 | 1.85E-02 | 7.14E-02 |
| KCNK2 | 3776 | 0.5487325 | 1.8171695 | 3.15E-01 | 5.15E-01 |
| EGR1 | 1958 | 0.5486893 | 1.884821 | 4.06E-01 | 6.07E-01 |
| HADHA | 3030 | 0.5485913 | 7.0642288 | 3.88E-02 | 1.23E-01 |
| ENPP4 | 22875 | 0.5481229 | 4.2536145 | 9.05E-02 | 2.22E-01 |
| THRAP3 | 9967 | 0.5478359 | 7.914221 | 4.39E-03 | 2.40E-02 |
| CABLES2 | 81928 | 0.5477823 | 3.8112205 | 7.96E-02 | 2.04E-01 |
| SEPTIN11 | 55752 | 0.5475971 | 7.7278734 | 1.67E-02 | 6.62E-02 |
| NPHP1 | 4867 | 0.5470323 | 3.5972463 | 7.89E-02 | 2.03E-01 |
| PPT1 | 5538 | 0.546535 | 7.5723213 | 3.41E-02 | 1.12E-01 |
| TSTD3 | 100130890 | 0.5464223 | 2.4645131 | 8.81E-02 | 2.18E-01 |
| VPS35L | 57020 | 0.5463549 | 5.2875345 | 5.21E-03 | 2.77E-02 |
| CDC73 | 79577 | 0.5460371 | 5.355854 | 2.80E-02 | 9.68E-02 |
| NAA50 | 80218 | 0.5459783 | 6.3435193 | 3.00E-02 | 1.02E-01 |
| ERBIN | 55914 | 0.5455702 | 3.7784726 | 2.95E-01 | 4.93E-01 |
| CDKN1A | 1026 | 0.545478 | 8.6183001 | 4.48E-01 | 6.41E-01 |
| RTCB | 51493 | 0.5454266 | 7.2878507 | 2.17E-02 | 8.05E-02 |
| ZNF419 | 79744 | 0.5450286 | 3.5964319 | 1.29E-02 | 5.45E-02 |
| CAPS2 | 84698 | 0.5448946 | 3.1493632 | 9.32E-02 | 2.27E-01 |
| SF3B1 | 23451 | 0.5447535 | 7.6619362 | 4.33E-03 | 2.38E-02 |
| DNAJC7 | 7266 | 0.5444786 | 6.5365598 | 2.08E-02 | 7.79E-02 |
| ACSL3 | 2181 | 0.5442058 | 7.6196353 | 8.00E-03 | 3.83E-02 |
| LYSMD2 | 256586 | 0.5441528 | 4.9826586 | 1.11E-02 | 4.90E-02 |
| SRP72 | 6731 | 0.5433569 | 7.8846902 | 2.88E-03 | 1.72E-02 |
| CARD10 | 29775 | 0.5431069 | 2.3719176 | 4.99E-01 | 6.84E-01 |
| FAM114A2 | 10827 | 0.5429811 | 4.5435982 | 5.76E-02 | 1.63E-01 |
| CREM | 1390 | 0.5428837 | 6.3159186 | 3.34E-02 | 1.10E-01 |
| CFAP65 | 255101 | 0.5428415 | 2.198035 | 1.42E-01 | 3.05E-01 |
| CAP1 | 10487 | 0.5425928 | 7.3829813 | 6.61E-02 | 1.79E-01 |
| MTO1 | 25821 | 0.5424905 | 4.560072 | 5.17E-02 | 1.50E-01 |
| HRK | 8739 | 0.5422654 | 2.5826912 | 7.81E-02 | 2.01E-01 |
| PRR14L | 253143 | 0.5418376 | 5.1848059 | 7.80E-03 | 3.77E-02 |
| FAM53C | 51307 | 0.541745 | 5.1627109 | 2.16E-02 | 8.01E-02 |
| NANP | 140838 | 0.5414109 | 3.0934492 | 7.71E-02 | 1.99E-01 |
| YJU2 | 55702 | 0.541312 | 4.7853332 | 6.78E-03 | 3.39E-02 |
| WASHC5 | 9897 | 0.5412555 | 4.5467075 | 1.95E-02 | 7.42E-02 |
| UFM1 | 51569 | 0.5408489 | 7.0905381 | 5.02E-03 | 2.69E-02 |
| CLK1 | 1195 | 0.5408 | 6.1574336 | 1.22E-02 | 5.23E-02 |
| ZNF501 | 115560 | 0.5405321 | 3.6962771 | 2.98E-02 | 1.01E-01 |
| FXYD6 | 53826 | 0.5404704 | 8.0279754 | 2.99E-01 | 4.98E-01 |
| WDR45B | 56270 | 0.5404198 | 6.8751073 | 2.01E-02 | 7.61E-02 |
| ZNF7 | 7553 | 0.5403565 | 4.0878622 | 1.66E-02 | 6.58E-02 |
| VEPH1 | 79674 | 0.5403559 | 2.9549431 | 1.05E-01 | 2.48E-01 |
| DICER1 | 23405 | 0.5402857 | 5.9110251 | 1.08E-02 | 4.79E-02 |
| ZC3H14 | 79882 | 0.5402259 | 5.6841097 | 2.91E-02 | 9.95E-02 |
| C10orf88 | 80007 | 0.5398963 | 4.8080469 | 1.09E-02 | 4.82E-02 |
| PTTG1IP | 754 | 0.5395095 | 7.4504129 | 1.54E-01 | 3.21E-01 |
| CD47 | 961 | 0.5394789 | 7.4981853 | 1.29E-02 | 5.46E-02 |
| ARMH3 | 79591 | 0.5392305 | 4.4478577 | 2.72E-02 | 9.49E-02 |
| HLA-DPB1 | 3115 | 0.5390799 | 4.1062989 | 2.68E-01 | 4.64E-01 |
| APBB1 | 322 | 0.538887 | 8.4531351 | 5.34E-02 | 1.54E-01 |
| HTATSF1 | 27336 | 0.5386877 | 6.7012732 | 4.48E-02 | 1.36E-01 |
| FCHSD2 | 9873 | 0.5386487 | 4.9110525 | 1.61E-01 | 3.31E-01 |
| SERINC2 | 347735 | 0.538475 | 3.5820635 | 8.70E-02 | 2.16E-01 |
| PPP1CB | 5500 | 0.5380815 | 7.26107 | 1.46E-01 | 3.10E-01 |
| BROX | 148362 | 0.5380768 | 4.0780715 | 3.01E-02 | 1.02E-01 |
| HHLA3 | 11147 | 0.5378153 | 4.8533721 | 1.46E-02 | 5.98E-02 |
| NEFL | 4747 | 0.5371684 | 11.358137 | 3.01E-01 | 4.99E-01 |
| SGTB | 54557 | 0.5371175 | 4.7245544 | 6.35E-02 | 1.74E-01 |
| FBXO36 | 130888 | 0.5365572 | 4.0438867 | 1.05E-01 | 2.48E-01 |
| UBAP2 | 55833 | 0.5365342 | 4.9593108 | 2.78E-02 | 9.62E-02 |
| GPBP1 | 65056 | 0.5363276 | 7.0882131 | 1.34E-02 | 5.61E-02 |
| HINFP | 25988 | 0.5361968 | 4.7235094 | 4.22E-03 | 2.33E-02 |
| ZNF496 | 84838 | 0.5361887 | 5.3480946 | 7.22E-02 | 1.91E-01 |
| SYT11 | 23208 | 0.5355758 | 8.0892574 | 2.04E-02 | 7.67E-02 |
| EFHB | 151651 | 0.5355163 | 0.6424809 | 4.97E-01 | 6.83E-01 |
| ADIPOR1 | 51094 | 0.5354812 | 7.4720459 | 2.35E-02 | 8.52E-02 |
| UVRAG | 7405 | 0.5354286 | 4.6478807 | 6.77E-03 | 3.38E-02 |
| YME1L1 | 10730 | 0.5353669 | 6.9174161 | 2.22E-02 | 8.17E-02 |
| PIGF | 5281 | 0.5353104 | 5.3776685 | 4.28E-03 | 2.35E-02 |
| CNRIP1 | 25927 | 0.5347985 | 7.6810192 | 2.91E-01 | 4.89E-01 |
| TOR1AIP1 | 26092 | 0.534696 | 4.7370585 | 1.04E-01 | 2.47E-01 |
| MED24 | 9862 | 0.5346714 | 5.6141285 | 2.36E-02 | 8.56E-02 |
| SH3GL2 | 6456 | 0.5344011 | 6.4102603 | 3.89E-01 | 5.89E-01 |
| UPRT | 139596 | 0.5323677 | 5.6375768 | 9.00E-02 | 2.22E-01 |
| PXYLP1 | 92370 | 0.532319 | 6.0153632 | 3.33E-02 | 1.10E-01 |
| SEPTIN2 | 4735 | 0.5320062 | 7.3196916 | 1.56E-01 | 3.24E-01 |
| C4orf46 | 201725 | 0.5318513 | 3.1240064 | 5.23E-02 | 1.51E-01 |
| ZBTB38 | 253461 | 0.5316972 | 5.5598033 | 9.43E-03 | 4.32E-02 |
| LSM14A | 26065 | 0.5316187 | 6.4912361 | 9.57E-03 | 4.37E-02 |
| FRMD4A | 55691 | 0.5315168 | 4.4352525 | 3.95E-02 | 1.25E-01 |
| CCDC153 | 283152 | 0.5314611 | 0.3688052 | 5.26E-01 | 7.06E-01 |
| GGPS1 | 9453 | 0.5313755 | 5.1420235 | 1.37E-02 | 5.72E-02 |
| NFX1 | 4799 | 0.5312999 | 4.8479392 | 1.76E-02 | 6.86E-02 |
| ATP6V1A | 523 | 0.531045 | 8.6225129 | 8.26E-03 | 3.92E-02 |
| UBQLN2 | 29978 | 0.5302409 | 7.2133668 | 1.24E-02 | 5.28E-02 |
| IFIT5 | 24138 | 0.529492 | 5.9253479 | 4.78E-03 | 2.58E-02 |
| BIK | 638 | 0.529385 | 1.2708283 | 2.72E-01 | 4.68E-01 |
| CRTC2 | 200186 | 0.5293388 | 2.7777562 | 2.12E-01 | 3.99E-01 |
| GABPB1 | 2553 | 0.5292973 | 4.8811949 | 2.72E-02 | 9.49E-02 |
| FSD1L | 83856 | 0.5290215 | 5.1064913 | 4.05E-01 | 6.06E-01 |
| ZNF18 | 7566 | 0.5289138 | 3.4298074 | 3.64E-02 | 1.17E-01 |
| PFKFB3 | 5209 | 0.5287156 | 3.2563389 | 4.07E-01 | 6.07E-01 |
| AK3 | 50808 | 0.5284976 | 5.9966724 | 6.36E-02 | 1.74E-01 |
| CDK17 | 5128 | 0.5284933 | 5.5678167 | 5.29E-03 | 2.81E-02 |
| SLC25A53 | 401612 | 0.5284211 | 3.0411394 | 5.18E-02 | 1.50E-01 |
| TIGD4 | 201798 | 0.5282315 | 0.8628673 | 3.49E-01 | 5.50E-01 |
| SPSB2 | 84727 | 0.5281736 | 2.7392118 | 7.69E-02 | 1.99E-01 |
| TOM1 | 10043 | 0.5281017 | 5.1277746 | 8.26E-02 | 2.09E-01 |
| ZNF263 | 10127 | 0.5280582 | 4.8413948 | 8.10E-02 | 2.06E-01 |
| IFT46 | 56912 | 0.5278243 | 5.1057862 | 6.07E-03 | 3.13E-02 |
| ZNF416 | 55659 | 0.5277618 | 3.5945091 | 2.35E-02 | 8.52E-02 |
| SPAG5 | 10615 | 0.5274928 | 0.5249346 | 4.69E-01 | 6.59E-01 |
| PRKAB2 | 5565 | 0.5272915 | 4.5470167 | 1.77E-02 | 6.91E-02 |
| UNKL | 64718 | 0.5268032 | 1.1884839 | 3.39E-01 | 5.40E-01 |
| ACADM | 34 | 0.5267607 | 5.8433784 | 5.25E-03 | 2.80E-02 |
| LGI1 | 9211 | 0.5266254 | 5.9864377 | 6.27E-02 | 1.72E-01 |
| MMD | 23531 | 0.52654 | 6.5089997 | 1.81E-01 | 3.59E-01 |
| PRPF39 | 55015 | 0.5265078 | 4.5781388 | 6.07E-02 | 1.69E-01 |
| INTS14 | 81556 | 0.5261087 | 4.9255959 | 2.79E-02 | 9.67E-02 |
| PCNA | 5111 | 0.5260306 | 5.9789089 | 2.68E-02 | 9.38E-02 |
| NRIP3 | 56675 | 0.5255617 | 6.087526 | 1.73E-01 | 3.47E-01 |
| ZNF45 | 7596 | 0.5255284 | 3.2564529 | 8.23E-02 | 2.09E-01 |
| MMUT | 4594 | 0.5253874 | 5.7820883 | 1.12E-02 | 4.94E-02 |
| KIFBP | 26128 | 0.5248782 | 6.6461115 | 2.55E-03 | 1.56E-02 |
| ALOX12B | 242 | 0.524856 | 0.8583286 | 2.85E-01 | 4.83E-01 |
| CYB561 | 1534 | 0.5247816 | 6.3500053 | 9.78E-03 | 4.43E-02 |
| ACTR3 | 10096 | 0.5245884 | 7.8360107 | 3.82E-02 | 1.22E-01 |
| JUP | 3728 | 0.5243091 | 6.2958878 | 1.17E-01 | 2.68E-01 |
| DCAF12L2 | 340578 | 0.5243043 | 2.727954 | 1.76E-01 | 3.52E-01 |
| GIT2 | 9815 | 0.5241096 | 4.5626332 | 1.74E-02 | 6.83E-02 |
| CDC23 | 8697 | 0.524104 | 5.3696471 | 2.69E-02 | 9.42E-02 |
| DDX5 | 1655 | 0.5237743 | 9.9018087 | 6.45E-03 | 3.27E-02 |
| POMT2 | 29954 | 0.5237383 | 3.3620202 | 2.15E-01 | 4.02E-01 |
| PHF21A | 51317 | 0.523488 | 4.7568729 | 4.26E-02 | 1.31E-01 |
| AHCTF1 | 25909 | 0.523427 | 3.9881705 | 7.54E-02 | 1.96E-01 |
| SBNO1 | 55206 | 0.5232601 | 6.9399208 | 8.54E-03 | 4.03E-02 |
| RPE | 6120 | 0.5231879 | 4.8964272 | 5.79E-02 | 1.63E-01 |
| GFPT1 | 2673 | 0.5231173 | 6.6214594 | 1.69E-02 | 6.67E-02 |
| PPP1R3E | 90673 | 0.5230127 | 2.8782087 | 1.36E-01 | 2.95E-01 |
| UBE2D1 | 7321 | 0.5227039 | 7.2476796 | 2.73E-02 | 9.50E-02 |
| NCL | 4691 | 0.5225941 | 9.0170157 | 8.80E-03 | 4.11E-02 |
| SRRT | 51593 | 0.5225639 | 5.2732121 | 5.12E-02 | 1.49E-01 |
| PARP6 | 56965 | 0.5223477 | 7.1329109 | 2.49E-01 | 4.43E-01 |
| DENND2A | 27147 | 0.5223461 | 3.9465936 | 2.20E-01 | 4.08E-01 |
| SPATA20 | 64847 | 0.522237 | 5.0882045 | 1.35E-01 | 2.95E-01 |
| AMMECR1L | 83607 | 0.5220479 | 3.5173915 | 1.74E-01 | 3.49E-01 |
| FAM169A | 26049 | 0.5218844 | 4.8691111 | 1.44E-01 | 3.07E-01 |
| CHRNA6 | 8973 | 0.5215636 | 2.7093658 | 3.40E-01 | 5.41E-01 |
| KMT5B | 51111 | 0.5210892 | 6.2285844 | 1.12E-02 | 4.94E-02 |
| GNPNAT1 | 64841 | 0.5209347 | 3.6183 | 7.95E-02 | 2.04E-01 |
| EI24 | 9538 | 0.5208836 | 8.2834323 | 5.08E-03 | 2.72E-02 |
| HSPA13 | 6782 | 0.5206917 | 6.8930548 | 4.01E-03 | 2.23E-02 |
| VAMP4 | 8674 | 0.5206313 | 4.8328972 | 2.57E-02 | 9.11E-02 |
| WDSUB1 | 151525 | 0.5204276 | 4.7367832 | 5.94E-03 | 3.08E-02 |
| NIPSNAP2 | 2631 | 0.5201264 | 7.036413 | 5.32E-03 | 2.82E-02 |
| VPS39 | 23339 | 0.5201108 | 4.9821834 | 2.85E-02 | 9.78E-02 |
| SPARC | 6678 | 0.5200464 | 9.5056513 | 4.18E-01 | 6.17E-01 |
| KLF3 | 51274 | 0.519719 | 4.8045761 | 4.20E-02 | 1.30E-01 |
| NTNG2 | 84628 | 0.5196832 | 0.7295775 | 2.07E-01 | 3.93E-01 |
| IP6K1 | 9807 | 0.5195838 | 4.9195552 | 5.58E-02 | 1.59E-01 |
| GPN1 | 11321 | 0.5192003 | 5.6386923 | 5.65E-02 | 1.60E-01 |
| MAPK8IP1 | 9479 | 0.5188196 | 4.2045795 | 2.08E-01 | 3.94E-01 |
| WDR47 | 22911 | 0.5188023 | 6.9156002 | 1.41E-01 | 3.03E-01 |
| STOM | 2040 | 0.5186491 | 3.1178194 | 5.70E-02 | 1.61E-01 |
| HMX1 | 3166 | 0.5184964 | 1.8948368 | 1.01E-01 | 2.40E-01 |
| DYNLRB2 | 83657 | 0.5184357 | 1.6570467 | 2.14E-01 | 4.02E-01 |
| CEBPG | 1054 | 0.5181649 | 5.9737267 | 4.29E-03 | 2.36E-02 |
| DNAJA2 | 10294 | 0.5181134 | 7.4301037 | 3.85E-03 | 2.17E-02 |
| TIPRL | 261726 | 0.5180356 | 7.7658511 | 7.24E-02 | 1.91E-01 |
| UPF3A | 65110 | 0.5177992 | 5.7782653 | 1.06E-02 | 4.73E-02 |
| RAI14 | 26064 | 0.5173139 | 4.5784754 | 2.88E-01 | 4.87E-01 |
| ANKRA2 | 57763 | 0.516665 | 5.0950967 | 1.16E-01 | 2.66E-01 |
| SMIM15 | 643155 | 0.5165767 | 6.1622307 | 1.78E-02 | 6.92E-02 |
| HDGFL3 | 50810 | 0.5163909 | 7.5421876 | 1.99E-01 | 3.83E-01 |
| DR1 | 1810 | 0.5162535 | 6.0926897 | 6.26E-03 | 3.20E-02 |
| PICALM | 8301 | 0.5162261 | 5.089327 | 5.37E-03 | 2.85E-02 |
| WDR3 | 10885 | 0.5160002 | 5.0362591 | 7.18E-03 | 3.53E-02 |
| NET1 | 10276 | 0.5159904 | 2.9617586 | 8.39E-02 | 2.11E-01 |
| AP1G1 | 164 | 0.5158082 | 5.1046126 | 2.47E-02 | 8.85E-02 |
| GNB5 | 10681 | 0.5157964 | 6.4586103 | 8.72E-03 | 4.09E-02 |
| VTA1 | 51534 | 0.5157569 | 7.8746255 | 4.10E-03 | 2.27E-02 |
| KLHDC8A | 55220 | 0.5156573 | 4.9366154 | 3.27E-01 | 5.28E-01 |
| ABCC4 | 10257 | 0.5151779 | 1.7322434 | 4.27E-01 | 6.25E-01 |
| TSTD2 | 158427 | 0.5150426 | 4.2789052 | 1.04E-01 | 2.45E-01 |
| TRPC4AP | 26133 | 0.5150161 | 6.4579126 | 4.26E-02 | 1.31E-01 |
| SERPINH1 | 871 | 0.5148861 | 5.3149802 | 3.38E-01 | 5.38E-01 |
| WDR75 | 84128 | 0.5148819 | 5.3107726 | 6.56E-03 | 3.30E-02 |
| SCG5 | 6447 | 0.5147011 | 0.4921961 | 2.55E-01 | 4.49E-01 |
| CEP152 | 22995 | 0.5146966 | 1.4219793 | 3.82E-01 | 5.82E-01 |
| MBTD1 | 54799 | 0.5144546 | 3.2927992 | 1.22E-01 | 2.75E-01 |
| SCOC | 60592 | 0.514447 | 9.0764291 | 7.86E-03 | 3.79E-02 |
| ZDHHC15 | 158866 | 0.5141467 | 4.5790954 | 1.95E-02 | 7.42E-02 |
| CCDC90B | 60492 | 0.5140456 | 8.0378742 | 4.22E-02 | 1.31E-01 |
| COPS4 | 51138 | 0.5138922 | 7.6748759 | 1.61E-02 | 6.42E-02 |
| TYW1 | 55253 | 0.5135729 | 3.5311495 | 8.24E-02 | 2.09E-01 |
| TTC37 | 9652 | 0.5135233 | 5.7434682 | 2.57E-02 | 9.12E-02 |
| USP33 | 23032 | 0.5133954 | 6.7057917 | 4.39E-03 | 2.40E-02 |
| PLPBP | 11212 | 0.5126805 | 6.0757423 | 2.55E-02 | 9.06E-02 |
| OXA1L | 5018 | 0.5126609 | 6.2548988 | 4.19E-03 | 2.31E-02 |
| MTAP | 4507 | 0.5124576 | 3.5798789 | 1.31E-01 | 2.88E-01 |
| ZNF76 | 7629 | 0.5123339 | 4.0698574 | 2.84E-02 | 9.77E-02 |
| GSE1 | 23199 | 0.5122582 | 4.2232018 | 3.70E-02 | 1.19E-01 |
| USPL1 | 10208 | 0.51219 | 5.2628351 | 1.14E-02 | 4.97E-02 |
| ZNF714 | 148206 | 0.5121228 | 5.033208 | 1.51E-01 | 3.17E-01 |
| DCUN1D2 | 55208 | 0.5118172 | 4.2678402 | 2.57E-02 | 9.12E-02 |
| INPP1 | 3628 | 0.5117367 | 5.8162719 | 6.19E-02 | 1.71E-01 |
| CDC25B | 994 | 0.5116869 | 3.8794463 | 7.14E-02 | 1.89E-01 |
| CREG2 | 200407 | 0.5114106 | 5.017915 | 5.25E-01 | 7.06E-01 |
| PPP2R2B | 5521 | 0.5111386 | 8.2114381 | 3.79E-01 | 5.78E-01 |
| GLUL | 2752 | 0.5110823 | 7.782897 | 6.39E-02 | 1.74E-01 |
| CMTM3 | 123920 | 0.5110216 | 3.5332829 | 3.09E-01 | 5.08E-01 |
| SRSF5 | 6430 | 0.5110196 | 7.574109 | 2.29E-02 | 8.36E-02 |
| VPS4B | 9525 | 0.5108731 | 5.5241244 | 6.13E-03 | 3.15E-02 |
| RIMS3 | 9783 | 0.5105884 | 5.7814434 | 3.19E-01 | 5.19E-01 |
| DHRS7 | 51635 | 0.5105624 | 7.3792128 | 6.86E-03 | 3.41E-02 |
| EEF1E1 | 9521 | 0.5105251 | 5.9267895 | 2.29E-02 | 8.37E-02 |
| CEP192 | 55125 | 0.5105118 | 2.2308056 | 1.42E-01 | 3.04E-01 |
| FEM1C | 56929 | 0.5103645 | 4.3277644 | 1.94E-01 | 3.76E-01 |
| CHAMP1 | 283489 | 0.510228 | 3.779744 | 6.75E-02 | 1.81E-01 |
| RAB39A | 54734 | 0.5101792 | 0.9418579 | 4.13E-01 | 6.12E-01 |
| CFAP126 | 257177 | 0.5100723 | 1.3025737 | 2.77E-01 | 4.74E-01 |
| HECA | 51696 | 0.5100327 | 4.2697515 | 1.12E-01 | 2.59E-01 |
| CD200 | 4345 | 0.5099491 | 8.6280469 | 2.81E-01 | 4.78E-01 |
| ABCB7 | 22 | 0.509802 | 3.4960409 | 3.69E-02 | 1.19E-01 |
| SPA17 | 53340 | 0.5091708 | 3.3428417 | 5.92E-02 | 1.66E-01 |
| NUDCD1 | 84955 | 0.509109 | 5.3413394 | 1.15E-02 | 5.00E-02 |
| GGCT | 79017 | 0.5084583 | 5.6271725 | 2.07E-02 | 7.77E-02 |
| RAB11FIP2 | 22841 | 0.5082914 | 5.5263266 | 5.73E-02 | 1.62E-01 |
| ZNF582 | 147948 | 0.508034 | 2.3994247 | 1.01E-01 | 2.41E-01 |
| PDCD2 | 5134 | 0.5080053 | 6.5067137 | 4.30E-03 | 2.36E-02 |
| ACADS | 35 | 0.5077077 | 1.3519374 | 1.31E-01 | 2.88E-01 |
| SDK2 | 54549 | 0.5072746 | 2.3523357 | 3.42E-01 | 5.43E-01 |
| RAB3GAP2 | 25782 | 0.5070608 | 5.517895 | 1.14E-02 | 4.98E-02 |
| EIF4E | 1977 | 0.5069725 | 8.1696618 | 1.18E-02 | 5.12E-02 |
| XPNPEP3 | 63929 | 0.5069212 | 3.2942141 | 1.36E-01 | 2.95E-01 |
| UFC1 | 51506 | 0.5069132 | 6.761341 | 2.54E-02 | 9.04E-02 |
| CBWD5 | 220869 | 0.506805 | 4.7836336 | 2.82E-02 | 9.73E-02 |
| ZNF280C | 55609 | 0.5067638 | 2.5785521 | 1.76E-01 | 3.51E-01 |
| METTL15 | 196074 | 0.5062449 | 3.8271811 | 2.63E-02 | 9.25E-02 |
| ZNF337 | 26152 | 0.5059373 | 0.8920273 | 3.65E-01 | 5.64E-01 |
| RBL2 | 5934 | 0.5058364 | 4.1985269 | 1.91E-02 | 7.33E-02 |
| ZNF92 | 168374 | 0.5049747 | 4.139264 | 4.63E-02 | 1.39E-01 |
| AP2B1 | 163 | 0.5048967 | 6.4468579 | 2.87E-02 | 9.86E-02 |
| ABHD14B | 84836 | 0.5047493 | 2.3019777 | 4.74E-01 | 6.63E-01 |
| DCLRE1B | 64858 | 0.5047112 | 3.925699 | 2.36E-02 | 8.56E-02 |
| ARMC12 | 221481 | 0.5044476 | 0.5712374 | 4.96E-01 | 6.82E-01 |
| LAMTOR3 | 8649 | 0.504443 | 7.2792871 | 5.59E-03 | 2.93E-02 |
| PIGW | 284098 | 0.503933 | 3.3013631 | 3.55E-02 | 1.15E-01 |
| ARMH1 | 339541 | 0.5039144 | 2.2224356 | 2.54E-01 | 4.48E-01 |
| GTF2H2C | 728340 | 0.5038222 | 4.0714701 | 1.64E-02 | 6.50E-02 |
| PPP1R15A | 23645 | 0.5037996 | 6.9088585 | 1.33E-01 | 2.91E-01 |
| TMED8 | 283578 | 0.5036272 | 4.4083946 | 8.97E-03 | 4.17E-02 |
| BABAM2 | 9577 | 0.5035634 | 5.8020129 | 2.06E-02 | 7.75E-02 |
| ZNF708 | 7562 | 0.5034613 | 4.0443108 | 9.58E-02 | 2.31E-01 |
| COG3 | 83548 | 0.5034243 | 4.091619 | 6.95E-02 | 1.86E-01 |
| UBXN8 | 7993 | 0.5033533 | 3.161311 | 6.07E-02 | 1.69E-01 |
| CENPJ | 55835 | 0.5032208 | 3.3076949 | 1.76E-01 | 3.52E-01 |
| SCYL2 | 55681 | 0.5031983 | 5.0650951 | 1.00E-02 | 4.52E-02 |
| ZNF207 | 7756 | 0.5025476 | 6.6231579 | 3.52E-02 | 1.15E-01 |
| RSPH9 | 221421 | 0.5023086 | 4.0092862 | 9.22E-02 | 2.25E-01 |
| NVL | 4931 | 0.5020465 | 4.3633213 | 1.39E-02 | 5.78E-02 |
| RCAN1 | 1827 | 0.5015659 | 8.7196269 | 1.32E-01 | 2.90E-01 |
| JAM3 | 83700 | 0.5012199 | 4.3935958 | 2.43E-01 | 4.35E-01 |
| SUV39H1 | 6839 | 0.5012036 | 1.8269042 | 1.11E-01 | 2.58E-01 |
| MIS12 | 79003 | 0.5011273 | 4.8537684 | 2.88E-02 | 9.86E-02 |
| C5orf51 | 285636 | 0.5010275 | 5.1407267 | 3.70E-02 | 1.19E-01 |
| SQOR | 58472 | 0.5010195 | 0.5497702 | 5.40E-01 | 7.16E-01 |
| ANKRD17 | 26057 | 0.5008317 | 6.1167964 | 1.12E-02 | 4.93E-02 |
| UBE2Z | 65264 | 0.5006449 | 6.9189978 | 1.10E-02 | 4.86E-02 |
| ANKRD10 | 55608 | 0.5005937 | 5.2874086 | 3.03E-02 | 1.03E-01 |
| SLIT3 | 6586 | 0.5005695 | 3.4007733 | 2.31E-01 | 4.23E-01 |
| FGFR1OP2 | 26127 | 0.5005378 | 5.3639605 | 2.82E-02 | 9.73E-02 |
| E2F6 | 1876 | 0.5004532 | 4.395253 | 1.43E-01 | 3.05E-01 |
| KCTD10 | 83892 | 0.5001978 | 5.6482376 | 7.02E-03 | 3.47E-02 |
| NIPAL3 | 57185 | 0.500191 | 4.5549547 | 1.49E-02 | 6.09E-02 |
| POLR3A | 11128 | 0.5000097 | 5.09464 | 9.36E-03 | 4.29E-02 |
| SMYD4 | 114826 | 0.5000093 | 3.1466537 | 1.12E-01 | 2.58E-01 |
| SLC25A27 | 9481 | 0.5000016 | 4.0601986 | 4.38E-01 | 6.32E-01 |
| ERI2 | 112479 | 0.4999414 | 1.8623153 | 1.11E-01 | 2.58E-01 |
| FNDC3B | 64778 | 0.4998504 | 5.1964857 | 3.42E-01 | 5.43E-01 |
| CDK5RAP2 | 55755 | 0.4998074 | 3.9588491 | 1.89E-01 | 3.70E-01 |
| HHAT | 55733 | 0.4995881 | 1.4529289 | 2.60E-01 | 4.56E-01 |
| COQ8B | 79934 | 0.4993586 | 2.8825938 | 7.21E-02 | 1.91E-01 |
| NEDD1 | 121441 | 0.4990765 | 3.8490746 | 2.60E-01 | 4.55E-01 |
| LRRC58 | 116064 | 0.4982791 | 4.5716748 | 1.35E-01 | 2.95E-01 |
| CHRAC1 | 54108 | 0.4980283 | 3.5916468 | 8.95E-02 | 2.21E-01 |
| CLP1 | 10978 | 0.4979254 | 4.5938274 | 1.01E-02 | 4.54E-02 |
| ROCK2 | 9475 | 0.4979037 | 5.3109055 | 5.34E-02 | 1.54E-01 |
| PIP4P1 | 90809 | 0.4978518 | 5.9218125 | 1.18E-02 | 5.12E-02 |
| STX2 | 2054 | 0.4977444 | 4.178207 | 8.29E-02 | 2.09E-01 |
| SEPTIN6 | 23157 | 0.497688 | 7.3774763 | 1.98E-01 | 3.82E-01 |
| NUFIP2 | 57532 | 0.4975599 | 5.2165452 | 6.67E-03 | 3.34E-02 |
| RHOQ | 23433 | 0.4974129 | 4.5341437 | 2.81E-02 | 9.71E-02 |
| RAB10 | 10890 | 0.4973008 | 7.8362118 | 9.58E-03 | 4.37E-02 |
| CYTH2 | 9266 | 0.4972489 | 6.1725397 | 1.81E-02 | 7.03E-02 |
| HELLS | 3070 | 0.497178 | 2.6720314 | 1.24E-01 | 2.78E-01 |
| SRP54 | 6729 | 0.4967647 | 7.0466591 | 1.09E-02 | 4.83E-02 |
| BBS7 | 55212 | 0.4964367 | 5.7521391 | 1.39E-02 | 5.78E-02 |
| CLEC2D | 29121 | 0.4963665 | 0.4241782 | 4.90E-01 | 6.77E-01 |
| UBE2H | 7328 | 0.4962978 | 7.667128 | 6.94E-02 | 1.85E-01 |
| MED21 | 9412 | 0.4955039 | 6.568476 | 1.10E-02 | 4.88E-02 |
| RO60 | 6738 | 0.4953843 | 7.5630645 | 9.58E-03 | 4.37E-02 |
| LAS1L | 81887 | 0.4950473 | 4.2780133 | 2.12E-01 | 3.98E-01 |
| DENR | 8562 | 0.4946592 | 7.4214523 | 4.02E-03 | 2.24E-02 |
| DNAJC28 | 54943 | 0.4944167 | 3.2199319 | 1.61E-01 | 3.30E-01 |
| ME2 | 4200 | 0.4943126 | 6.2631333 | 5.45E-03 | 2.88E-02 |
| CPD | 1362 | 0.4942641 | 4.4194515 | 3.04E-01 | 5.03E-01 |
| LONRF2 | 164832 | 0.4942498 | 5.5664347 | 1.41E-02 | 5.82E-02 |
| ZNHIT6 | 54680 | 0.4941663 | 4.7351213 | 4.22E-02 | 1.31E-01 |
| LRBA | 987 | 0.4940743 | 2.6862663 | 3.95E-01 | 5.95E-01 |
| CDC27 | 996 | 0.4938002 | 6.3990183 | 9.00E-03 | 4.18E-02 |
| BLOC1S6 | 26258 | 0.4936583 | 6.7324253 | 2.27E-02 | 8.31E-02 |
| DECR1 | 1666 | 0.4936207 | 6.2964228 | 2.26E-02 | 8.30E-02 |
| CHTOP | 26097 | 0.4935818 | 6.8826139 | 1.34E-02 | 5.61E-02 |
| MKRN1 | 23608 | 0.4933635 | 7.8454584 | 1.52E-02 | 6.16E-02 |
| PDSS1 | 23590 | 0.4933242 | 3.5934305 | 5.12E-02 | 1.49E-01 |
| MYSM1 | 114803 | 0.4932369 | 3.4345838 | 1.02E-01 | 2.42E-01 |
| TRIM39 | 56658 | 0.49315 | 2.597249 | 1.17E-01 | 2.67E-01 |
| COX15 | 1355 | 0.4926358 | 3.7027776 | 8.43E-02 | 2.12E-01 |
| TCEA1 | 6917 | 0.4918449 | 6.9144951 | 1.64E-02 | 6.51E-02 |
| ZNF274 | 10782 | 0.4915855 | 5.5952964 | 1.61E-02 | 6.42E-02 |
| PET117 | 100303755 | 0.491583 | -0.015322 | 3.38E-01 | 5.39E-01 |
| MAP3K8 | 1326 | 0.4915565 | 1.973798 | 4.18E-01 | 6.17E-01 |
| DNAAF2 | 55172 | 0.4913533 | 6.0650295 | 3.83E-02 | 1.22E-01 |
| TMTC4 | 84899 | 0.491242 | 5.6608101 | 1.12E-01 | 2.59E-01 |
| HSDL1 | 83693 | 0.4911118 | 7.2613057 | 1.34E-01 | 2.93E-01 |
| VPS52 | 6293 | 0.4908013 | 4.1796926 | 7.87E-02 | 2.02E-01 |
| TRMT11 | 60487 | 0.4907866 | 5.0744886 | 1.83E-02 | 7.08E-02 |
| HSD17B4 | 3295 | 0.4904172 | 6.2480082 | 2.57E-02 | 9.12E-02 |
| TBL2 | 26608 | 0.4904134 | 5.4437704 | 9.81E-02 | 2.36E-01 |
| CNOT4 | 4850 | 0.4902928 | 5.1957488 | 9.46E-03 | 4.33E-02 |
| TSPAN18 | 90139 | 0.4900223 | 3.1034048 | 3.56E-01 | 5.56E-01 |
| ARMCX1 | 51309 | 0.4897486 | 7.3583034 | 1.87E-01 | 3.67E-01 |
| API5 | 8539 | 0.4892749 | 6.1080936 | 8.97E-03 | 4.17E-02 |
| RNF170 | 81790 | 0.4892161 | 5.9166301 | 5.14E-03 | 2.74E-02 |
| VPS13C | 54832 | 0.4891478 | 4.6486966 | 5.89E-02 | 1.65E-01 |
| SPEF2 | 79925 | 0.488961 | 3.0041306 | 1.43E-01 | 3.05E-01 |
| PMP22 | 5376 | 0.488897 | 5.8666189 | 4.29E-01 | 6.26E-01 |
| UFD1 | 7353 | 0.4882877 | 6.3574811 | 1.43E-02 | 5.88E-02 |
| RERG | 85004 | 0.4878539 | 1.866157 | 1.36E-01 | 2.95E-01 |
| SYPL1 | 6856 | 0.487848 | 5.225597 | 3.13E-01 | 5.13E-01 |
| RELCH | 57614 | 0.4876861 | 4.6317684 | 4.31E-02 | 1.32E-01 |
| LTN1 | 26046 | 0.4875469 | 4.9011342 | 1.76E-02 | 6.88E-02 |
| PBXIP1 | 57326 | 0.4874529 | 5.6924327 | 4.40E-01 | 6.34E-01 |
| C1orf198 | 84886 | 0.486721 | 5.1156219 | 1.81E-01 | 3.59E-01 |
| TMEM184C | 55751 | 0.486675 | 5.9306596 | 9.00E-02 | 2.22E-01 |
| EIF2AK1 | 27102 | 0.4864139 | 6.7342411 | 7.00E-02 | 1.86E-01 |
| NEIL2 | 252969 | 0.4863889 | 4.6711441 | 3.94E-02 | 1.24E-01 |
| TSACC | 128229 | 0.4861923 | 0.4826228 | 4.37E-01 | 6.32E-01 |
| NUDT18 | 79873 | 0.4858536 | 2.7687709 | 1.21E-01 | 2.73E-01 |
| LIFR | 3977 | 0.4857568 | 5.3187771 | 2.07E-01 | 3.93E-01 |
| LIPH | 200879 | 0.4857425 | 0.673778 | 3.08E-01 | 5.07E-01 |
| CACHD1 | 57685 | 0.4856012 | 4.3525145 | 1.72E-01 | 3.46E-01 |
| PSMC6 | 5706 | 0.4855332 | 7.5521926 | 1.12E-02 | 4.93E-02 |
| ERCC6 | 2074 | 0.4854134 | 3.459461 | 3.57E-02 | 1.16E-01 |
| NOL8 | 55035 | 0.4853679 | 4.8995736 | 3.51E-02 | 1.15E-01 |
| CENPC | 1060 | 0.4853128 | 3.6680325 | 1.49E-01 | 3.14E-01 |
| IFNGR1 | 3459 | 0.4851579 | 6.3344547 | 8.87E-02 | 2.19E-01 |
| VIPAS39 | 63894 | 0.4845481 | 4.2962033 | 1.09E-01 | 2.54E-01 |
| NSL1 | 25936 | 0.4844773 | 6.5505815 | 1.34E-02 | 5.61E-02 |
| CFAP298 | 56683 | 0.4835904 | 3.570485 | 1.43E-01 | 3.06E-01 |
| CCDC91 | 55297 | 0.483525 | 5.6334527 | 1.38E-02 | 5.75E-02 |
| RASGRP3 | 25780 | 0.4834786 | 3.9494082 | 3.46E-01 | 5.46E-01 |
| LSG1 | 55341 | 0.4834553 | 5.6807903 | 2.19E-02 | 8.10E-02 |
| OBI1 | 79596 | 0.4834302 | 5.2674209 | 1.76E-01 | 3.52E-01 |
| ZNF678 | 339500 | 0.4832053 | 2.625397 | 3.09E-01 | 5.08E-01 |
| PRKCE | 5581 | 0.4830971 | 3.7615729 | 3.23E-01 | 5.24E-01 |
| ZBED9 | 114821 | 0.4828744 | 2.9831478 | 1.73E-01 | 3.47E-01 |
| RHOA | 387 | 0.4827455 | 8.5591912 | 2.70E-02 | 9.44E-02 |
| PUS7 | 54517 | 0.4826747 | 2.6663347 | 2.52E-01 | 4.46E-01 |
| CAMK2D | 817 | 0.4822455 | 5.3477036 | 1.85E-01 | 3.64E-01 |
| OPA1 | 4976 | 0.4821384 | 5.5410753 | 1.21E-02 | 5.20E-02 |
| PPP1CC | 5501 | 0.4821158 | 6.8803764 | 6.45E-03 | 3.27E-02 |
| FKBP15 | 23307 | 0.4819248 | 3.7259441 | 7.29E-02 | 1.92E-01 |
| CHPT1 | 56994 | 0.4818784 | 6.4026382 | 7.63E-03 | 3.70E-02 |
| SIMC1 | 375484 | 0.4815253 | 4.385601 | 6.72E-02 | 1.81E-01 |
| CCSER2 | 54462 | 0.4813924 | 7.0841035 | 1.07E-02 | 4.78E-02 |
| SMAD2 | 4087 | 0.4813465 | 6.7694139 | 9.17E-02 | 2.24E-01 |
| TSPAN19 | 144448 | 0.4810688 | 2.093391 | 2.63E-01 | 4.58E-01 |
| NSD1 | 64324 | 0.4807733 | 5.7268304 | 7.42E-02 | 1.94E-01 |
| LAMA2 | 3908 | 0.480566 | 1.699394 | 4.33E-01 | 6.28E-01 |
| ACTR8 | 93973 | 0.4799131 | 5.2152993 | 2.54E-02 | 9.04E-02 |
| RGS19 | 10287 | 0.4798217 | 3.6481532 | 8.55E-02 | 2.14E-01 |
| PAK2 | 5062 | 0.4797128 | 5.4613718 | 1.68E-02 | 6.65E-02 |
| DENND2B | 6764 | 0.4795178 | 3.6366386 | 3.44E-01 | 5.45E-01 |
| FBXL5 | 26234 | 0.4794968 | 6.827909 | 4.88E-02 | 1.44E-01 |
| STYX | 6815 | 0.4793816 | 3.7534218 | 1.94E-01 | 3.76E-01 |
| TBC1D9B | 23061 | 0.4793535 | 4.7489405 | 1.36E-01 | 2.95E-01 |
| PIH1D2 | 120379 | 0.4792041 | 2.1975278 | 1.27E-01 | 2.81E-01 |
| TRABD2A | 129293 | 0.4790877 | 0.2389967 | 3.67E-01 | 5.66E-01 |
| INAFM2 | 100505573 | 0.4790756 | 2.6529938 | 1.21E-01 | 2.73E-01 |
| QRSL1 | 55278 | 0.4785634 | 5.0538854 | 3.44E-02 | 1.13E-01 |
| LARP4B | 23185 | 0.4785477 | 5.0049283 | 2.94E-02 | 1.00E-01 |
| CORO1C | 23603 | 0.4784953 | 8.1705369 | 8.83E-03 | 4.12E-02 |
| COPA | 1314 | 0.4784683 | 6.7077802 | 7.65E-02 | 1.98E-01 |
| CASP2 | 835 | 0.4783898 | 3.1861336 | 1.44E-01 | 3.07E-01 |
| SENP6 | 26054 | 0.4783643 | 5.7480495 | 6.51E-03 | 3.28E-02 |
| ZNF157 | 7712 | 0.4783256 | 1.2444245 | 3.32E-01 | 5.33E-01 |
| NEK8 | 284086 | 0.4781473 | 1.3314958 | 2.81E-01 | 4.78E-01 |
| MAP2 | 4133 | 0.4780916 | 9.0266979 | 4.52E-02 | 1.37E-01 |
| RNPEP | 6051 | 0.4780489 | 4.0199146 | 2.16E-01 | 4.04E-01 |
| USP27X | 389856 | 0.4779162 | 1.6604438 | 1.46E-01 | 3.10E-01 |
| CDC16 | 8881 | 0.4779152 | 5.4245271 | 5.76E-02 | 1.63E-01 |
| ACTR10 | 55860 | 0.4777209 | 8.354566 | 2.94E-02 | 1.00E-01 |
| MARK3 | 4140 | 0.477691 | 6.6800244 | 1.10E-02 | 4.86E-02 |
| SEPTIN9 | 10801 | 0.4773472 | 4.356215 | 4.75E-02 | 1.42E-01 |
| SSR3 | 6747 | 0.4773074 | 6.9189934 | 1.36E-01 | 2.95E-01 |
| PPM1B | 5495 | 0.4771055 | 5.0789601 | 1.10E-02 | 4.85E-02 |
| ELP1 | 8518 | 0.4769348 | 4.8268351 | 3.63E-02 | 1.17E-01 |
| GATC | 283459 | 0.4767134 | 4.9627041 | 1.03E-02 | 4.63E-02 |
| PDLIM7 | 9260 | 0.4764602 | 5.996479 | 1.74E-02 | 6.82E-02 |
| SUCO | 51430 | 0.4760788 | 5.6615618 | 2.62E-02 | 9.23E-02 |
| WAC | 51322 | 0.4758016 | 6.5904859 | 2.26E-02 | 8.30E-02 |
| ZNF596 | 169270 | 0.4757066 | 3.1089498 | 4.38E-02 | 1.34E-01 |
| WDR7 | 23335 | 0.4756818 | 4.3836716 | 3.59E-02 | 1.16E-01 |
| RAPGEF2 | 9693 | 0.4756207 | 4.6573227 | 2.29E-02 | 8.37E-02 |
| CSKMT | 751071 | 0.4753198 | 1.5857518 | 1.76E-01 | 3.52E-01 |
| CHN1 | 1123 | 0.4747905 | 7.1347556 | 1.17E-01 | 2.67E-01 |
| YPEL1 | 29799 | 0.4747823 | 5.7080966 | 3.59E-01 | 5.58E-01 |
| EFHD1 | 80303 | 0.4745796 | 2.2412608 | 3.69E-01 | 5.68E-01 |
| ZNF766 | 90321 | 0.474109 | 4.8615999 | 4.25E-02 | 1.31E-01 |
| RABGGTB | 5876 | 0.473643 | 5.2254971 | 1.20E-02 | 5.18E-02 |
| GNB1 | 2782 | 0.4733947 | 8.4285101 | 2.74E-02 | 9.54E-02 |
| THAP12 | 5612 | 0.4733508 | 4.5392424 | 5.73E-02 | 1.62E-01 |
| ZNF329 | 79673 | 0.4733482 | 3.768339 | 6.99E-02 | 1.86E-01 |
| STX12 | 23673 | 0.4733279 | 6.9900021 | 6.66E-02 | 1.80E-01 |
| DMAC2L | 27109 | 0.4732264 | 2.2287005 | 1.59E-01 | 3.27E-01 |
| SEC22C | 9117 | 0.4732128 | 5.8268029 | 4.66E-02 | 1.40E-01 |
| SMURF1 | 57154 | 0.4731804 | 3.3102165 | 9.34E-02 | 2.27E-01 |
| RBM4B | 83759 | 0.4729705 | 6.3427647 | 1.54E-02 | 6.24E-02 |
| PITPNB | 23760 | 0.4728577 | 6.6105687 | 8.63E-03 | 4.06E-02 |
| DNM2 | 1785 | 0.4728375 | 4.8787952 | 2.25E-01 | 4.14E-01 |
| ADSL | 158 | 0.4726725 | 2.8172209 | 1.02E-01 | 2.42E-01 |
| RIOK1 | 83732 | 0.4724804 | 5.6416173 | 9.76E-02 | 2.35E-01 |
| CLGN | 1047 | 0.4719231 | 6.1797647 | 1.59E-02 | 6.37E-02 |
| RBM6 | 10180 | 0.4718369 | 5.5501315 | 1.46E-02 | 5.98E-02 |
| CENPE | 1062 | 0.4716839 | 1.8512823 | 3.36E-01 | 5.37E-01 |
| FLVCR1 | 28982 | 0.4715671 | 4.66335 | 1.23E-01 | 2.76E-01 |
| MYO3A | 53904 | 0.471456 | 1.235899 | 4.12E-01 | 6.11E-01 |
| COPG1 | 22820 | 0.4712287 | 7.009079 | 1.58E-01 | 3.25E-01 |
| DAP | 1611 | 0.471066 | 6.2372428 | 3.36E-01 | 5.37E-01 |
| RNF185 | 91445 | 0.4709968 | 5.324896 | 4.01E-02 | 1.26E-01 |
| SACM1L | 22908 | 0.4707667 | 4.8807145 | 4.11E-02 | 1.28E-01 |
| ZNF404 | 342908 | 0.4703477 | 1.4535614 | 2.00E-01 | 3.84E-01 |
| ATXN7L3B | 552889 | 0.4701866 | 7.5010659 | 8.69E-02 | 2.16E-01 |
| ATG12 | 9140 | 0.4701468 | 7.0425421 | 7.25E-02 | 1.91E-01 |
| NAE1 | 8883 | 0.4701374 | 7.308631 | 2.74E-02 | 9.54E-02 |
| BAG4 | 9530 | 0.4699935 | 5.4109624 | 9.54E-03 | 4.36E-02 |
| MPHOSPH6 | 10200 | 0.4698886 | 5.9851176 | 2.69E-02 | 9.42E-02 |
| FBLN7 | 129804 | 0.4698025 | 0.6165407 | 4.53E-01 | 6.45E-01 |
| HACD3 | 51495 | 0.4697289 | 9.189667 | 1.20E-02 | 5.19E-02 |
| LACTB2 | 51110 | 0.468934 | 2.840153 | 2.55E-01 | 4.50E-01 |
| LSS | 4047 | 0.4687374 | 5.4134963 | 1.28E-01 | 2.83E-01 |
| ANKFY1 | 51479 | 0.4685701 | 4.5942192 | 2.69E-02 | 9.42E-02 |
| TIRAP | 114609 | 0.4685503 | 2.2017294 | 1.01E-01 | 2.41E-01 |
| AGPS | 8540 | 0.4685371 | 4.9792721 | 1.53E-02 | 6.20E-02 |
| ARIH2 | 10425 | 0.4682934 | 6.3838079 | 8.68E-03 | 4.07E-02 |
| DNTTIP2 | 30836 | 0.4682871 | 6.953526 | 1.76E-02 | 6.89E-02 |
| RPS27L | 51065 | 0.4681991 | 7.3626162 | 6.80E-02 | 1.82E-01 |
| ZNF644 | 84146 | 0.4681501 | 6.7550889 | 1.15E-02 | 5.01E-02 |
| SMG8 | 55181 | 0.4680873 | 4.5520829 | 4.52E-02 | 1.37E-01 |
| METTL18 | 92342 | 0.4677533 | 3.9579937 | 9.11E-02 | 2.23E-01 |
| ZFAND6 | 54469 | 0.4677296 | 7.918427 | 2.80E-02 | 9.68E-02 |
| TGFBRAP1 | 9392 | 0.4676953 | 4.5321721 | 5.32E-02 | 1.53E-01 |
| EXOC4 | 60412 | 0.4675524 | 5.3847065 | 2.58E-02 | 9.14E-02 |
| ABL1 | 25 | 0.4674313 | 4.3903197 | 2.35E-01 | 4.27E-01 |
| ATG9A | 79065 | 0.4673391 | 4.5452972 | 1.97E-01 | 3.80E-01 |
| ZNF880 | 400713 | 0.4672253 | 3.9888807 | 4.80E-02 | 1.43E-01 |
| TXLNA | 200081 | 0.4671979 | 4.3762812 | 1.32E-01 | 2.90E-01 |
| RRN3 | 54700 | 0.4669864 | 5.3907263 | 1.04E-02 | 4.63E-02 |
| BCORL1 | 63035 | 0.4668726 | 3.148294 | 1.21E-01 | 2.74E-01 |
| PLCD1 | 5333 | 0.4667315 | 2.6725743 | 3.19E-01 | 5.19E-01 |
| TMC6 | 11322 | 0.4666778 | 0.8557716 | 3.96E-01 | 5.96E-01 |
| CREBRF | 153222 | 0.4666744 | 4.0305703 | 8.03E-02 | 2.05E-01 |
| OGA | 10724 | 0.4665813 | 6.5741497 | 1.16E-02 | 5.06E-02 |
| CXXC1 | 30827 | 0.4665089 | 5.35691 | 2.64E-02 | 9.28E-02 |
| MAST3 | 23031 | 0.4664526 | 2.488053 | 2.79E-01 | 4.77E-01 |
| DCPS | 28960 | 0.4662385 | 4.289498 | 6.44E-02 | 1.75E-01 |
| ARPP19 | 10776 | 0.4657763 | 8.4229986 | 2.87E-02 | 9.86E-02 |
| ZNF384 | 171017 | 0.4657492 | 2.6763034 | 1.61E-01 | 3.31E-01 |
| PARP8 | 79668 | 0.465666 | 4.1731909 | 2.30E-01 | 4.22E-01 |
| RBM7 | 10179 | 0.4653694 | 5.6554774 | 1.23E-01 | 2.76E-01 |
| FNDC3A | 22862 | 0.4650833 | 6.198165 | 1.98E-02 | 7.50E-02 |
| GRB2 | 2885 | 0.4648796 | 7.6465666 | 2.08E-02 | 7.81E-02 |
| SLC1A4 | 6509 | 0.4643499 | 5.9610344 | 3.96E-02 | 1.25E-01 |
| OMA1 | 115209 | 0.4642888 | 2.3224544 | 2.64E-01 | 4.59E-01 |
| ZBTB1 | 22890 | 0.4640089 | 4.4288111 | 2.66E-01 | 4.62E-01 |
| ZBTB6 | 10773 | 0.4639397 | 3.8632992 | 1.36E-01 | 2.95E-01 |
| MYOM2 | 9172 | 0.4638357 | 3.5785268 | 3.79E-01 | 5.78E-01 |
| MRAP2 | 112609 | 0.4638242 | 3.298875 | 9.50E-02 | 2.30E-01 |
| ECI1 | 1632 | 0.4635508 | 4.7060676 | 7.27E-02 | 1.91E-01 |
| SBDS | 51119 | 0.4634232 | 7.265137 | 1.00E-02 | 4.51E-02 |
| RNASEH1 | 246243 | 0.4630239 | 4.6878932 | 2.02E-02 | 7.62E-02 |
| NYNRIN | 57523 | 0.4628463 | 2.4277388 | 2.39E-01 | 4.30E-01 |
| PRELID3B | 51012 | 0.4627179 | 5.5744053 | 2.23E-02 | 8.22E-02 |
| RFWD3 | 55159 | 0.4626015 | 2.7264109 | 1.82E-01 | 3.60E-01 |
| RAP1B | 5908 | 0.4618889 | 7.5559921 | 1.11E-02 | 4.90E-02 |
| GTF2H1 | 2965 | 0.4617549 | 5.9128161 | 4.43E-02 | 1.35E-01 |
| STK11IP | 114790 | 0.4614758 | 3.212902 | 1.41E-01 | 3.02E-01 |
| NAA16 | 79612 | 0.4613359 | 3.8226501 | 6.24E-02 | 1.72E-01 |
| RIT1 | 6016 | 0.4612891 | 5.8437694 | 6.38E-02 | 1.74E-01 |
| LNPK | 80856 | 0.4611689 | 5.7823067 | 3.10E-02 | 1.04E-01 |
| UEVLD | 55293 | 0.4610103 | 4.5123805 | 1.14E-01 | 2.63E-01 |
| NCAPG2 | 54892 | 0.4609807 | 2.137514 | 2.52E-01 | 4.46E-01 |
| AMIGO1 | 57463 | 0.4606972 | 3.9118609 | 1.16E-01 | 2.65E-01 |
| CCDC184 | 387856 | 0.4606481 | 6.4956484 | 3.81E-02 | 1.21E-01 |
| PCNX4 | 64430 | 0.4605725 | 6.3625468 | 1.42E-02 | 5.85E-02 |
| MRPL21 | 219927 | 0.4601065 | 6.8544366 | 1.39E-01 | 3.01E-01 |
| PSMA1 | 5682 | 0.4600787 | 5.4951224 | 3.09E-02 | 1.04E-01 |
| TCAIM | 285343 | 0.4600347 | 5.3808754 | 2.62E-02 | 9.23E-02 |
| SAP130 | 79595 | 0.4599413 | 4.5428908 | 3.55E-02 | 1.15E-01 |
| FKRP | 79147 | 0.4598771 | 3.84954 | 4.82E-02 | 1.43E-01 |
| SLC44A2 | 57153 | 0.4593875 | 5.6517252 | 1.84E-01 | 3.63E-01 |
| ATP8A1 | 10396 | 0.4591424 | 3.6064673 | 4.54E-01 | 6.46E-01 |
| PSMC1 | 5700 | 0.459132 | 6.6859245 | 1.43E-02 | 5.88E-02 |
| SNRNP40 | 9410 | 0.4590591 | 6.6109863 | 1.07E-02 | 4.77E-02 |
| HIRA | 7290 | 0.4589844 | 2.2062611 | 2.50E-01 | 4.43E-01 |
| TRIM52 | 84851 | 0.4587474 | 3.2673563 | 1.08E-01 | 2.52E-01 |
| ZBTB40 | 9923 | 0.4585563 | 2.4236102 | 3.04E-01 | 5.03E-01 |
| IFT81 | 28981 | 0.4579562 | 6.0594607 | 2.56E-02 | 9.09E-02 |
| RAB30 | 27314 | 0.4577644 | 4.6465718 | 2.53E-01 | 4.47E-01 |
| AFF4 | 27125 | 0.4576637 | 6.5461646 | 5.94E-02 | 1.66E-01 |
| SMU1 | 55234 | 0.4575358 | 6.1449598 | 2.38E-02 | 8.61E-02 |
| CHKA | 1119 | 0.4573193 | 5.5759215 | 1.10E-01 | 2.56E-01 |
| ZNF74 | 7625 | 0.4570361 | 4.6878386 | 7.97E-02 | 2.04E-01 |
| RALGPS2 | 55103 | 0.4567776 | 4.430237 | 6.71E-02 | 1.81E-01 |
| RBM10 | 8241 | 0.456646 | 4.4331693 | 6.26E-02 | 1.72E-01 |
| EFNA1 | 1942 | 0.4565095 | 4.040474 | 2.99E-01 | 4.97E-01 |
| POT1 | 25913 | 0.4559037 | 4.0437045 | 6.72E-02 | 1.81E-01 |
| BMP1 | 649 | 0.4557478 | 1.7570613 | 5.07E-01 | 6.91E-01 |
| SPAG16 | 79582 | 0.4557351 | 6.416962 | 4.64E-02 | 1.40E-01 |
| TRPM8 | 79054 | 0.4556782 | 0.8960362 | 4.61E-01 | 6.52E-01 |
| TAX1BP1 | 8887 | 0.4555759 | 7.949329 | 2.49E-02 | 8.91E-02 |
| DAB2 | 1601 | 0.4554776 | 3.2104828 | 5.34E-01 | 7.11E-01 |
| KARS1 | 3735 | 0.4554762 | 7.5182716 | 1.57E-02 | 6.32E-02 |
| GRK3 | 157 | 0.4554639 | 3.6739414 | 4.94E-01 | 6.80E-01 |
| PKIA | 5569 | 0.4553089 | 9.6275403 | 5.27E-01 | 7.06E-01 |
| SUPT7L | 9913 | 0.4552854 | 5.7982482 | 1.89E-02 | 7.26E-02 |
| UTP4 | 84916 | 0.455174 | 6.3808468 | 1.90E-02 | 7.31E-02 |
| PER3 | 8863 | 0.4549465 | 1.6345052 | 3.47E-01 | 5.48E-01 |
| PDLIM1 | 9124 | 0.4537073 | 4.8868098 | 4.71E-01 | 6.60E-01 |
| RNF227 | 284023 | 0.4534478 | 3.2508658 | 7.51E-02 | 1.96E-01 |
| SGCB | 6443 | 0.4533304 | 6.9313729 | 6.46E-02 | 1.76E-01 |
| VGLL4 | 9686 | 0.4532815 | 6.0340631 | 5.91E-02 | 1.66E-01 |
| PDIK1L | 149420 | 0.4531814 | 5.1390881 | 2.32E-01 | 4.23E-01 |
| ASCC2 | 84164 | 0.4530549 | 4.9685511 | 1.42E-01 | 3.04E-01 |
| VAC14 | 55697 | 0.4529407 | 5.5618822 | 1.61E-02 | 6.42E-02 |
| EIF2D | 1939 | 0.4526464 | 3.462301 | 3.02E-01 | 5.00E-01 |
| DNAJC13 | 23317 | 0.4525526 | 3.7791088 | 1.44E-01 | 3.06E-01 |
| PNN | 5411 | 0.4523812 | 6.5065153 | 1.94E-02 | 7.40E-02 |
| PI4K2A | 55361 | 0.4523741 | 3.8907438 | 2.25E-01 | 4.14E-01 |
| PIFO | 128344 | 0.4522867 | 4.6259292 | 1.18E-01 | 2.69E-01 |
| CCDC82 | 79780 | 0.4522055 | 6.3356354 | 3.09E-02 | 1.04E-01 |
| CKAP2 | 26586 | 0.4520228 | 5.0568565 | 3.31E-02 | 1.10E-01 |
| ZNF443 | 10224 | 0.4513672 | 2.9270817 | 2.24E-01 | 4.14E-01 |
| ZCCHC7 | 84186 | 0.4510105 | 4.8243831 | 1.96E-02 | 7.46E-02 |
| UBE2K | 3093 | 0.4508869 | 7.0898741 | 1.93E-02 | 7.38E-02 |
| NDRG4 | 65009 | 0.4508809 | 8.8664327 | 3.12E-01 | 5.12E-01 |
| DUSP10 | 11221 | 0.450547 | 3.9254966 | 3.78E-01 | 5.77E-01 |
| ZNF689 | 115509 | 0.4503211 | 4.5828515 | 5.63E-02 | 1.60E-01 |
| DNAJC18 | 202052 | 0.4502999 | 5.8461524 | 1.59E-02 | 6.38E-02 |
| MAPK12 | 6300 | 0.4499259 | 4.1793151 | 1.98E-02 | 7.50E-02 |
| METTL14 | 57721 | 0.4497829 | 5.4754093 | 1.09E-01 | 2.54E-01 |
| MAPK14 | 1432 | 0.4495219 | 4.6800473 | 1.21E-01 | 2.73E-01 |
| PAPPA | 5069 | 0.4494998 | 2.8266941 | 5.28E-01 | 7.07E-01 |
| ARMCX2 | 9823 | 0.4494367 | 4.99935 | 2.59E-02 | 9.15E-02 |
| MCU | 90550 | 0.4489961 | 4.791705 | 2.11E-02 | 7.87E-02 |
| BAK1 | 578 | 0.4486784 | 4.1283628 | 2.76E-01 | 4.72E-01 |
| REPIN1 | 29803 | 0.4486265 | 4.7660538 | 1.32E-01 | 2.91E-01 |
| SPECC1L | 23384 | 0.4483933 | 1.6940323 | 2.97E-01 | 4.95E-01 |
| TRA2A | 29896 | 0.4481491 | 5.1547655 | 2.82E-02 | 9.73E-02 |
| NPTN | 27020 | 0.4478567 | 6.5560371 | 6.38E-02 | 1.74E-01 |
| IRF5 | 3663 | 0.4476975 | 1.8177602 | 5.11E-01 | 6.94E-01 |
| HSPA14 | 51182 | 0.4474853 | 4.6040899 | 5.25E-02 | 1.52E-01 |
| G3BP1 | 10146 | 0.44745 | 6.8025953 | 1.46E-01 | 3.09E-01 |
| ANXA4 | 307 | 0.4472206 | 3.6511253 | 4.56E-01 | 6.48E-01 |
| PSMD2 | 5708 | 0.447183 | 8.4307947 | 6.79E-02 | 1.82E-01 |
| KANSL3 | 55683 | 0.4466759 | 3.6220526 | 6.44E-02 | 1.75E-01 |
| ZNF41 | 7592 | 0.4464842 | 2.9581833 | 1.05E-01 | 2.48E-01 |
| CHST1 | 8534 | 0.4458183 | 2.7706672 | 3.51E-01 | 5.51E-01 |
| COQ5 | 84274 | 0.445593 | 6.4583662 | 1.13E-02 | 4.96E-02 |
| UBA5 | 79876 | 0.4453875 | 5.789097 | 2.15E-02 | 8.00E-02 |
| COG1 | 9382 | 0.4453691 | 4.4801777 | 6.53E-02 | 1.77E-01 |
| GYG2 | 8908 | 0.4453625 | 5.6849542 | 1.84E-01 | 3.63E-01 |
| TRIB1 | 10221 | 0.4452858 | 3.7847871 | 2.33E-01 | 4.24E-01 |
| COQ10B | 80219 | 0.4449486 | 6.2271411 | 1.49E-01 | 3.13E-01 |
| HNRNPLL | 92906 | 0.444916 | 5.9038483 | 2.62E-02 | 9.23E-02 |
| SULF2 | 55959 | 0.4443077 | 5.4502149 | 2.79E-01 | 4.77E-01 |
| PI4KB | 5298 | 0.4439802 | 5.7534215 | 1.06E-01 | 2.49E-01 |
| MX1 | 4599 | 0.4438054 | 0.9892864 | 3.26E-01 | 5.26E-01 |
| ABRAXAS2 | 23172 | 0.4437332 | 5.0858415 | 2.05E-02 | 7.70E-02 |
| CRACD | 57482 | 0.4433627 | 4.6754377 | 1.40E-01 | 3.01E-01 |
| SPATA17 | 128153 | 0.4432609 | 3.0188329 | 1.05E-01 | 2.48E-01 |
| YWHAH | 7533 | 0.4431572 | 10.903616 | 4.18E-01 | 6.18E-01 |
| OSBPL8 | 114882 | 0.4431357 | 6.9745282 | 7.63E-02 | 1.98E-01 |
| SCRN1 | 9805 | 0.4429892 | 7.2509254 | 2.52E-02 | 8.99E-02 |
| RRP1B | 23076 | 0.4425611 | 5.4775036 | 1.44E-02 | 5.91E-02 |
| NUP62 | 23636 | 0.4424046 | 5.0014563 | 3.18E-02 | 1.06E-01 |
| PIN4 | 5303 | 0.4423723 | 6.4909086 | 7.23E-02 | 1.91E-01 |
| KIF21A | 55605 | 0.4423582 | 7.8800226 | 9.28E-02 | 2.26E-01 |
| BRAP | 8315 | 0.4422434 | 4.9146192 | 6.16E-02 | 1.70E-01 |
| ARHGAP22 | 58504 | 0.4420594 | 2.1694675 | 2.42E-01 | 4.34E-01 |
| ZNF71 | 58491 | 0.4417132 | 4.7194543 | 4.64E-02 | 1.40E-01 |
| RUFY3 | 22902 | 0.4417128 | 9.1262468 | 2.04E-01 | 3.89E-01 |
| DUSP12 | 11266 | 0.4414738 | 6.5989968 | 1.62E-01 | 3.32E-01 |
| DNAJC10 | 54431 | 0.4412547 | 5.7157539 | 1.28E-01 | 2.84E-01 |
| CCDC186 | 55088 | 0.4411453 | 5.8041836 | 9.36E-02 | 2.28E-01 |
| BLZF1 | 8548 | 0.4409414 | 4.7127667 | 7.48E-02 | 1.95E-01 |
| ARFGEF2 | 10564 | 0.4408874 | 4.1472543 | 4.99E-02 | 1.46E-01 |
| KIF5B | 3799 | 0.440056 | 8.394425 | 2.72E-02 | 9.49E-02 |
| UBXN7 | 26043 | 0.4399684 | 5.4727477 | 4.39E-02 | 1.34E-01 |
| HSF2 | 3298 | 0.4398825 | 6.4635314 | 6.51E-02 | 1.76E-01 |
| FANCM | 57697 | 0.4398505 | 2.0545007 | 3.43E-01 | 5.44E-01 |
| ARF6 | 382 | 0.4398253 | 5.8671975 | 2.77E-02 | 9.61E-02 |
| WHRN | 25861 | 0.4397289 | 2.8117732 | 9.42E-02 | 2.29E-01 |
| SMNDC1 | 10285 | 0.4396868 | 4.1777156 | 8.80E-02 | 2.18E-01 |
| SLC9A7 | 84679 | 0.4396264 | 4.4993391 | 2.83E-02 | 9.75E-02 |
| HIF1AN | 55662 | 0.4395704 | 5.3362108 | 1.02E-01 | 2.42E-01 |
| AKIRIN2 | 55122 | 0.439022 | 6.8203827 | 3.01E-02 | 1.02E-01 |
| GDAP2 | 54834 | 0.4389622 | 4.4482596 | 3.55E-02 | 1.15E-01 |
| RAB14 | 51552 | 0.4389553 | 7.0978126 | 1.96E-02 | 7.45E-02 |
| METTL2B | 55798 | 0.4389468 | 4.8386953 | 2.78E-02 | 9.63E-02 |
| TTC33 | 23548 | 0.438921 | 5.5686967 | 1.65E-01 | 3.36E-01 |
| ME1 | 4199 | 0.4389034 | 5.3818078 | 3.71E-01 | 5.70E-01 |
| HS3ST5 | 222537 | 0.4388143 | 2.5176136 | 3.06E-01 | 5.06E-01 |
| NAA30 | 122830 | 0.4387232 | 4.5955689 | 6.16E-02 | 1.70E-01 |
| NKAPD1 | 55216 | 0.4386701 | 5.4213068 | 5.91E-02 | 1.65E-01 |
| BRD4 | 23476 | 0.4383707 | 5.9484962 | 4.89E-02 | 1.44E-01 |
| C20orf96 | 140680 | 0.4382342 | 1.9285475 | 1.50E-01 | 3.15E-01 |
| CARS1 | 833 | 0.4381705 | 6.3924659 | 5.30E-02 | 1.53E-01 |
| ZBTB11 | 27107 | 0.4381497 | 4.1036396 | 2.90E-02 | 9.92E-02 |
| MRPL45 | 84311 | 0.4377322 | 5.8691927 | 1.45E-02 | 5.96E-02 |
| RBM19 | 9904 | 0.4376084 | 3.5732242 | 7.87E-02 | 2.02E-01 |
| KIF3A | 11127 | 0.4373989 | 8.7433589 | 3.04E-01 | 5.03E-01 |
| ATP9B | 374868 | 0.4372785 | 3.3711335 | 2.13E-01 | 4.00E-01 |
| RPAP3 | 79657 | 0.4368547 | 5.6440766 | 1.12E-02 | 4.94E-02 |
| XRN1 | 54464 | 0.436606 | 4.2602789 | 6.06E-02 | 1.69E-01 |
| PDCD4 | 27250 | 0.4365448 | 5.03343 | 2.72E-01 | 4.68E-01 |
| ZCCHC10 | 54819 | 0.4360201 | 5.5948998 | 4.09E-02 | 1.28E-01 |
| MYH10 | 4628 | 0.4359532 | 7.4089909 | 3.82E-02 | 1.21E-01 |
| PTPN2 | 5771 | 0.4358494 | 4.8774699 | 3.49E-02 | 1.14E-01 |
| LZTS2 | 84445 | 0.4356887 | 4.3317655 | 1.76E-01 | 3.51E-01 |
| AK7 | 122481 | 0.4355503 | 2.3635988 | 3.59E-01 | 5.59E-01 |
| DLGAP3 | 58512 | 0.4353919 | 1.5141707 | 4.56E-01 | 6.47E-01 |
| MTRR | 4552 | 0.434919 | 3.6590146 | 2.06E-01 | 3.91E-01 |
| CFL2 | 1073 | 0.434788 | 6.5860578 | 4.98E-02 | 1.46E-01 |
| RMND5A | 64795 | 0.4343537 | 4.8234303 | 6.89E-02 | 1.84E-01 |
| CUL5 | 8065 | 0.4339692 | 5.8650921 | 1.46E-02 | 5.98E-02 |
| ATE1 | 11101 | 0.4338747 | 5.1297464 | 2.69E-02 | 9.42E-02 |
| EPHA8 | 2046 | 0.4337596 | 2.0621052 | 3.07E-01 | 5.06E-01 |
| MED1 | 5469 | 0.4334087 | 4.4475976 | 4.56E-02 | 1.38E-01 |
| FANCC | 2176 | 0.4333493 | 2.7297024 | 1.20E-01 | 2.72E-01 |
| ZNF607 | 84775 | 0.4329789 | 3.0952526 | 1.12E-01 | 2.59E-01 |
| NRBF2 | 29982 | 0.4329602 | 5.7257654 | 2.77E-02 | 9.60E-02 |
| CARNMT1 | 138199 | 0.4328065 | 2.6223567 | 1.20E-01 | 2.71E-01 |
| FAF2 | 23197 | 0.4327041 | 6.3945113 | 4.94E-02 | 1.45E-01 |
| EBAG9 | 9166 | 0.4323155 | 5.1012097 | 4.30E-02 | 1.32E-01 |
| NUDT16 | 131870 | 0.4323027 | 4.876519 | 6.79E-02 | 1.82E-01 |
| PHC2 | 1912 | 0.4321886 | 5.6156092 | 1.03E-01 | 2.44E-01 |
| ZNF221 | 7638 | 0.4321166 | 1.5384194 | 3.68E-01 | 5.68E-01 |
| ZNF775 | 285971 | 0.4320659 | 3.7503294 | 6.95E-02 | 1.86E-01 |
| TBC1D13 | 54662 | 0.4316262 | 4.92191 | 4.06E-02 | 1.27E-01 |
| KPNA1 | 3836 | 0.4314339 | 5.6140219 | 8.34E-02 | 2.10E-01 |
| TSGA10 | 80705 | 0.4314292 | 4.1870439 | 8.11E-02 | 2.06E-01 |
| PPM1D | 8493 | 0.4312801 | 4.5940324 | 1.85E-01 | 3.64E-01 |
| ZHX1 | 11244 | 0.4311503 | 5.3543956 | 1.69E-01 | 3.43E-01 |
| CRNKL1 | 51340 | 0.4306625 | 4.5725253 | 1.50E-01 | 3.15E-01 |
| ANKRD46 | 157567 | 0.4305507 | 7.4784399 | 2.94E-01 | 4.93E-01 |
| TARBP2 | 6895 | 0.4305381 | 5.1662193 | 3.28E-02 | 1.09E-01 |
| MTRF1L | 54516 | 0.4303278 | 4.0474988 | 3.49E-02 | 1.14E-01 |
| TMEM17 | 200728 | 0.4302982 | 4.7201191 | 3.72E-02 | 1.19E-01 |
| DBT | 1629 | 0.4302923 | 4.3727585 | 9.62E-02 | 2.32E-01 |
| NGLY1 | 55768 | 0.430091 | 5.1697494 | 7.11E-02 | 1.89E-01 |
| DNAJC14 | 85406 | 0.4299314 | 2.9700742 | 1.89E-01 | 3.70E-01 |
| INO80 | 54617 | 0.4298664 | 2.9510903 | 1.25E-01 | 2.79E-01 |
| MED16 | 10025 | 0.4298005 | 4.9832271 | 1.32E-01 | 2.90E-01 |
| MSANTD2 | 79684 | 0.4297351 | 3.8045848 | 3.77E-02 | 1.21E-01 |
| DCAF13 | 25879 | 0.4296002 | 6.5892952 | 3.61E-02 | 1.17E-01 |
| WDR82 | 80335 | 0.4295333 | 6.7232695 | 7.95E-02 | 2.04E-01 |
| RNF13 | 11342 | 0.4294927 | 6.850815 | 1.25E-01 | 2.79E-01 |
| RPL4 | 6124 | 0.429344 | 8.5719674 | 4.32E-02 | 1.33E-01 |
| TACC2 | 10579 | 0.4293342 | 5.4797063 | 4.54E-01 | 6.46E-01 |
| ZNF484 | 83744 | 0.4292155 | 3.6522511 | 1.10E-01 | 2.56E-01 |
| ATF6 | 22926 | 0.4291425 | 5.5048881 | 7.53E-02 | 1.96E-01 |
| PPP1R12A | 4659 | 0.4290715 | 6.512203 | 6.54E-02 | 1.77E-01 |
| DUS4L | 11062 | 0.4287843 | 1.6128003 | 2.98E-01 | 4.96E-01 |
| BICD1 | 636 | 0.4287374 | 5.4286313 | 3.86E-02 | 1.22E-01 |
| NUP43 | 348995 | 0.428716 | 3.3641697 | 7.73E-02 | 2.00E-01 |
| NUP155 | 9631 | 0.4285436 | 3.1899913 | 1.40E-01 | 3.02E-01 |
| PRCC | 5546 | 0.4281943 | 5.9252947 | 3.49E-02 | 1.14E-01 |
| DERL1 | 79139 | 0.4280513 | 5.9991585 | 9.77E-02 | 2.35E-01 |
| USF2 | 7392 | 0.4278143 | 4.567424 | 3.24E-02 | 1.08E-01 |
| RNF14 | 9604 | 0.4276905 | 6.6477242 | 2.75E-02 | 9.55E-02 |
| SAMD9 | 54809 | 0.427688 | 1.1026283 | 4.43E-01 | 6.37E-01 |
| PIGU | 128869 | 0.4275951 | 6.2580759 | 2.63E-02 | 9.25E-02 |
| TMEM267 | 64417 | 0.4274772 | 3.9954858 | 1.63E-01 | 3.34E-01 |
| ADAT2 | 134637 | 0.4274631 | 2.1702187 | 1.94E-01 | 3.76E-01 |
| VWA5A | 4013 | 0.427073 | 6.3041099 | 1.09E-01 | 2.54E-01 |
| PTPN18 | 26469 | 0.4268111 | 4.4761895 | 6.13E-02 | 1.70E-01 |
| HAS3 | 3038 | 0.4267681 | 3.4662945 | 2.78E-01 | 4.75E-01 |
| ZNF429 | 353088 | 0.4267234 | 5.4806488 | 3.03E-01 | 5.02E-01 |
| SRSF6 | 6431 | 0.4264354 | 4.5473292 | 5.26E-02 | 1.52E-01 |
| OSBPL7 | 114881 | 0.4261612 | 1.4174387 | 3.55E-01 | 5.55E-01 |
| RMDN1 | 51115 | 0.4258648 | 5.2971576 | 5.64E-02 | 1.60E-01 |
| C3orf38 | 285237 | 0.4253975 | 5.2639543 | 7.03E-02 | 1.87E-01 |
| SUMO2 | 6613 | 0.4253381 | 9.3120289 | 1.73E-01 | 3.48E-01 |
| UIMC1 | 51720 | 0.4251146 | 4.4362727 | 4.34E-02 | 1.33E-01 |
| CD46 | 4179 | 0.4246963 | 6.626944 | 1.71E-01 | 3.44E-01 |
| VTI1A | 143187 | 0.4244727 | 3.5656587 | 1.08E-01 | 2.53E-01 |
| RBM12 | 10137 | 0.4242007 | 4.9513298 | 8.53E-02 | 2.14E-01 |
| TXNDC11 | 51061 | 0.4241701 | 4.2662832 | 1.56E-01 | 3.24E-01 |
| MICU2 | 221154 | 0.4241322 | 6.5609403 | 3.86E-02 | 1.22E-01 |
| PPP2R5C | 5527 | 0.4241186 | 6.3490002 | 1.56E-02 | 6.29E-02 |
| DENND1A | 57706 | 0.4238724 | 3.5004699 | 1.81E-01 | 3.59E-01 |
| LSM11 | 134353 | 0.4237228 | 5.0286707 | 1.65E-01 | 3.36E-01 |
| ZNF253 | 56242 | 0.4236028 | 4.1676813 | 1.05E-01 | 2.48E-01 |
| ARFIP1 | 27236 | 0.423514 | 4.5614757 | 7.90E-02 | 2.03E-01 |
| TPGS2 | 25941 | 0.4235122 | 8.2681711 | 3.11E-02 | 1.05E-01 |
| EFCAB5 | 374786 | 0.423327 | 2.0150402 | 4.57E-01 | 6.49E-01 |
| USP46 | 64854 | 0.4231174 | 5.168334 | 1.95E-02 | 7.42E-02 |
| GANC | 2595 | 0.4230767 | 2.8960661 | 1.95E-01 | 3.77E-01 |
| IFT122 | 55764 | 0.4230387 | 4.4816636 | 2.76E-01 | 4.72E-01 |
| WDR33 | 55339 | 0.4228876 | 6.4180334 | 8.48E-02 | 2.13E-01 |
| SPG21 | 51324 | 0.4225518 | 7.3987271 | 1.68E-02 | 6.62E-02 |
| INIP | 58493 | 0.4225373 | 6.7100518 | 1.02E-01 | 2.42E-01 |
| TMTC3 | 160418 | 0.4224856 | 3.9253734 | 3.11E-01 | 5.10E-01 |
| FAM76B | 143684 | 0.4222491 | 4.6391478 | 1.35E-01 | 2.94E-01 |
| KANSL2 | 54934 | 0.4221281 | 6.2711307 | 2.17E-02 | 8.03E-02 |
| DIPK2A | 205428 | 0.4220402 | 4.2780519 | 9.95E-02 | 2.38E-01 |
| S100A13 | 6284 | 0.4219393 | 2.0747248 | 1.87E-01 | 3.67E-01 |
| NCAPD2 | 9918 | 0.4217755 | 2.3228013 | 1.99E-01 | 3.83E-01 |
| NDRG3 | 57446 | 0.4217317 | 7.4600158 | 1.34E-01 | 2.94E-01 |
| ZNF613 | 79898 | 0.4217037 | 3.8659328 | 9.57E-02 | 2.31E-01 |
| CMBL | 134147 | 0.4217036 | 5.2590558 | 1.06E-01 | 2.49E-01 |
| SPTSSA | 171546 | 0.4215367 | 7.2563923 | 1.13E-01 | 2.60E-01 |
| IQCC | 55721 | 0.4215347 | 2.2682176 | 1.94E-01 | 3.76E-01 |
| CYFIP2 | 26999 | 0.4213699 | 7.3399082 | 3.13E-02 | 1.05E-01 |
| PCBD2 | 84105 | 0.4211749 | 3.7222524 | 6.95E-02 | 1.86E-01 |
| TF | 7018 | 0.4211678 | 0.6155422 | 5.01E-01 | 6.86E-01 |
| SMURF2 | 64750 | 0.4203256 | 4.0885477 | 1.28E-01 | 2.83E-01 |
| SLC24A1 | 9187 | 0.4200372 | 0.0200655 | 3.92E-01 | 5.92E-01 |
| TBC1D12 | 23232 | 0.4199743 | 3.88272 | 1.18E-01 | 2.68E-01 |
| NMD3 | 51068 | 0.4199474 | 5.3106378 | 8.79E-02 | 2.18E-01 |
| PCNP | 57092 | 0.4199295 | 7.5661247 | 3.44E-02 | 1.13E-01 |
| ELK1 | 2002 | 0.4196083 | 1.884575 | 3.76E-01 | 5.75E-01 |
| FLRT3 | 23767 | 0.4195047 | 4.5401376 | 2.17E-01 | 4.05E-01 |
| APPBP2 | 10513 | 0.4194783 | 5.7784792 | 7.49E-02 | 1.96E-01 |
| FAM104B | 90736 | 0.4194268 | 5.1534631 | 1.35E-01 | 2.94E-01 |
| POLR2C | 5432 | 0.4194097 | 5.2230806 | 2.12E-02 | 7.88E-02 |
| RNFT1 | 51136 | 0.419252 | 2.6727927 | 2.61E-01 | 4.56E-01 |
| UFSP1 | 402682 | 0.4190307 | 0.9145578 | 2.82E-01 | 4.80E-01 |
| TTC13 | 79573 | 0.4188269 | 5.8957592 | 6.79E-02 | 1.82E-01 |
| ANKRD28 | 23243 | 0.4187693 | 4.1944629 | 1.48E-01 | 3.13E-01 |
| SLC16A1 | 6566 | 0.4185438 | 5.3125408 | 3.63E-01 | 5.62E-01 |
| BZW1 | 9689 | 0.4183933 | 8.0317419 | 5.00E-02 | 1.46E-01 |
| RBBP5 | 5929 | 0.4183439 | 4.5364439 | 3.78E-02 | 1.21E-01 |
| HSPA9 | 3313 | 0.4183132 | 8.2412668 | 1.46E-01 | 3.10E-01 |
| PKD2 | 5311 | 0.4182474 | 3.5150696 | 4.41E-01 | 6.35E-01 |
| CNEP1R1 | 255919 | 0.4180827 | 5.3302079 | 4.98E-02 | 1.46E-01 |
| TBC1D22A | 25771 | 0.4176987 | 3.5540734 | 1.95E-01 | 3.78E-01 |
| RBM4 | 5936 | 0.4172585 | 5.165629 | 4.97E-02 | 1.46E-01 |
| RABL3 | 285282 | 0.4169391 | 5.3037191 | 2.20E-02 | 8.14E-02 |
| CTDSPL2 | 51496 | 0.4169379 | 5.1079073 | 2.64E-02 | 9.28E-02 |
| TMEM39A | 55254 | 0.4167256 | 2.9823983 | 4.70E-01 | 6.59E-01 |
| KRBOX4 | 55634 | 0.4165954 | 5.219474 | 8.68E-02 | 2.16E-01 |
| POLB | 5423 | 0.4163218 | 7.0368671 | 3.02E-01 | 5.01E-01 |
| MAX | 4149 | 0.4163089 | 5.4644391 | 3.21E-02 | 1.07E-01 |
| DPYSL5 | 56896 | 0.4162895 | 8.0118493 | 4.38E-01 | 6.33E-01 |
| LPAR4 | 2846 | 0.416222 | 2.0851083 | 3.45E-01 | 5.45E-01 |
| SENP8 | 123228 | 0.4161689 | 3.0324336 | 2.37E-01 | 4.28E-01 |
| ALKBH5 | 54890 | 0.41607 | 5.4824768 | 2.83E-01 | 4.81E-01 |
| MAGEE1 | 57692 | 0.4155908 | 4.954923 | 2.72E-01 | 4.68E-01 |
| TLK2 | 11011 | 0.4154621 | 4.4082155 | 5.60E-02 | 1.59E-01 |
| PTS | 5805 | 0.4153889 | 7.8787213 | 1.30E-01 | 2.86E-01 |
| CPNE2 | 221184 | 0.4152225 | 6.6080836 | 3.63E-02 | 1.17E-01 |
| RTN3 | 10313 | 0.4152208 | 9.3677641 | 5.26E-02 | 1.52E-01 |
| RWDD3 | 25950 | 0.4150654 | 1.6828198 | 1.65E-01 | 3.37E-01 |
| C9orf85 | 138241 | 0.4150449 | 3.9521577 | 6.43E-02 | 1.75E-01 |
| ZNF468 | 90333 | 0.4149375 | 3.7255731 | 1.18E-01 | 2.68E-01 |
| ALDH1B1 | 219 | 0.4144626 | 4.5006785 | 2.22E-01 | 4.11E-01 |
| MFN2 | 9927 | 0.4142766 | 5.9528622 | 5.28E-02 | 1.53E-01 |
| PIGV | 55650 | 0.414257 | 3.4219311 | 3.13E-01 | 5.12E-01 |
| VAPA | 9218 | 0.4141576 | 9.1220108 | 3.93E-02 | 1.24E-01 |
| TBCK | 93627 | 0.4138356 | 4.4123103 | 3.61E-02 | 1.17E-01 |
| GSPT1 | 2935 | 0.4138001 | 6.6650998 | 4.97E-02 | 1.46E-01 |
| CSE1L | 1434 | 0.4135787 | 6.6302909 | 3.01E-02 | 1.02E-01 |
| HDLBP | 3069 | 0.4135445 | 6.9325106 | 2.21E-01 | 4.10E-01 |
| DCTD | 1635 | 0.4128834 | 5.4171595 | 2.56E-01 | 4.51E-01 |
| ZRSR2 | 8233 | 0.4128719 | 5.3045055 | 4.96E-02 | 1.46E-01 |
| RBM3 | 5935 | 0.4125853 | 6.2609871 | 8.03E-02 | 2.05E-01 |
| NCOA6 | 23054 | 0.4122021 | 4.9328055 | 1.93E-01 | 3.75E-01 |
| KLHDC2 | 23588 | 0.4121933 | 6.8388902 | 5.21E-02 | 1.51E-01 |
| ZNF510 | 22869 | 0.4120696 | 3.3585161 | 5.80E-02 | 1.63E-01 |
| ZBTB2 | 57621 | 0.4119222 | 3.4793687 | 1.36E-01 | 2.96E-01 |
| ZC4H2 | 55906 | 0.4118662 | 6.1133377 | 1.47E-01 | 3.11E-01 |
| LRRC41 | 10489 | 0.4116819 | 6.3313774 | 8.67E-02 | 2.16E-01 |
| ATRX | 546 | 0.4115189 | 8.0136972 | 1.01E-01 | 2.41E-01 |
| PHF13 | 148479 | 0.4111404 | 3.91622 | 1.23E-01 | 2.76E-01 |
| NFIA | 4774 | 0.4110244 | 4.4820796 | 4.16E-01 | 6.15E-01 |
| OTUD6B | 51633 | 0.4109616 | 4.1527429 | 1.26E-01 | 2.81E-01 |
| CC2D2A | 57545 | 0.4108353 | 4.0145545 | 1.05E-01 | 2.48E-01 |
| NPM1 | 4869 | 0.4107092 | 8.5513196 | 5.43E-02 | 1.56E-01 |
| CNKSR2 | 22866 | 0.4105891 | 4.4094644 | 2.34E-01 | 4.26E-01 |
| ATXN10 | 25814 | 0.4104478 | 8.5764299 | 2.25E-02 | 8.28E-02 |
| CREBZF | 58487 | 0.4100267 | 4.8735122 | 5.56E-02 | 1.59E-01 |
| CLSPN | 63967 | 0.4099661 | 3.5380993 | 4.03E-01 | 6.04E-01 |
| PHIP | 55023 | 0.409802 | 5.9895724 | 3.79E-02 | 1.21E-01 |
| ARHGAP17 | 55114 | 0.409566 | 3.1417488 | 2.98E-01 | 4.96E-01 |
| PSMC2 | 5701 | 0.4092171 | 7.9396891 | 2.10E-02 | 7.85E-02 |
| COX18 | 285521 | 0.4092084 | 3.9992662 | 7.48E-02 | 1.95E-01 |
| UBA2 | 10054 | 0.4090089 | 5.6600762 | 5.10E-02 | 1.49E-01 |
| SEMA4F | 10505 | 0.4090081 | 4.5838775 | 1.43E-01 | 3.06E-01 |
| F8 | 2157 | 0.408982 | 2.8167624 | 1.85E-01 | 3.64E-01 |
| USP34 | 9736 | 0.4077784 | 5.9544928 | 1.26E-01 | 2.81E-01 |
| USP16 | 10600 | 0.4077719 | 5.9075126 | 5.82E-02 | 1.64E-01 |
| TADA2A | 6871 | 0.4074332 | 4.7730117 | 7.50E-02 | 1.96E-01 |
| NOA1 | 84273 | 0.4070727 | 4.1436713 | 6.24E-02 | 1.72E-01 |
| C1orf52 | 148423 | 0.4069943 | 6.8596527 | 1.54E-01 | 3.20E-01 |
| CCDC77 | 84318 | 0.4069296 | 2.8190913 | 1.35E-01 | 2.95E-01 |
| DDX50 | 79009 | 0.4067838 | 5.6200787 | 2.80E-02 | 9.67E-02 |
| TEX2 | 55852 | 0.4067831 | 4.6157464 | 3.26E-01 | 5.26E-01 |
| HLCS | 3141 | 0.4066695 | 3.5453053 | 2.67E-01 | 4.62E-01 |
| RPGR | 6103 | 0.406479 | 2.9763521 | 1.65E-01 | 3.36E-01 |
| ZNF28 | 7576 | 0.4061102 | 3.6278448 | 1.39E-01 | 3.01E-01 |
| MAGI1 | 9223 | 0.4059297 | 5.494929 | 3.12E-01 | 5.12E-01 |
| USP15 | 9958 | 0.405839 | 6.0687246 | 3.08E-02 | 1.04E-01 |
| ARHGAP5 | 394 | 0.405497 | 5.4608301 | 1.24E-01 | 2.77E-01 |
| DNAJC25 | 548645 | 0.405198 | 4.0217388 | 1.99E-01 | 3.83E-01 |
| LEO1 | 123169 | 0.4050074 | 6.2772933 | 5.93E-02 | 1.66E-01 |
| TTC21B | 79809 | 0.4049885 | 3.3525394 | 1.13E-01 | 2.61E-01 |
| TRAPPC8 | 22878 | 0.4047783 | 3.5633597 | 6.60E-02 | 1.78E-01 |
| HIPK3 | 10114 | 0.4046358 | 4.2186273 | 2.03E-01 | 3.87E-01 |
| ZNF268 | 10795 | 0.4044558 | 4.0407229 | 1.84E-01 | 3.63E-01 |
| ZRANB2 | 9406 | 0.4043158 | 6.5093519 | 8.64E-02 | 2.15E-01 |
| LRCH1 | 23143 | 0.4041921 | 2.5127712 | 2.04E-01 | 3.89E-01 |
| FXN | 2395 | 0.4040745 | 3.5650474 | 1.56E-01 | 3.22E-01 |
| DHX16 | 8449 | 0.4040026 | 4.787687 | 1.17E-01 | 2.67E-01 |
| HERPUD2 | 64224 | 0.4040016 | 4.2790772 | 5.78E-02 | 1.63E-01 |
| FAM167A | 83648 | 0.4035972 | 3.8320569 | 1.77E-01 | 3.54E-01 |
| UNC80 | 285175 | 0.4035187 | 2.7797811 | 2.82E-01 | 4.80E-01 |
| NUP205 | 23165 | 0.4034237 | 3.2097624 | 1.79E-01 | 3.56E-01 |
| RBM23 | 55147 | 0.4033648 | 6.8806376 | 3.16E-02 | 1.06E-01 |
| PRDM10 | 56980 | 0.403336 | 2.33601 | 1.51E-01 | 3.17E-01 |
| LRRFIP1 | 9208 | 0.4029455 | 6.5271673 | 1.37E-01 | 2.97E-01 |
| U2SURP | 23350 | 0.4029092 | 6.9347984 | 1.49E-01 | 3.14E-01 |
| WDR25 | 79446 | 0.4027766 | 5.1521609 | 5.51E-02 | 1.58E-01 |
| ZC2HC1A | 51101 | 0.4026427 | 7.4601959 | 4.04E-01 | 6.05E-01 |
| NATD1 | 256302 | 0.4025207 | 1.9389053 | 2.36E-01 | 4.27E-01 |
| PANK3 | 79646 | 0.4024593 | 6.1020092 | 5.07E-02 | 1.48E-01 |
| SDC1 | 6382 | 0.4024432 | 4.332231 | 2.09E-01 | 3.96E-01 |
| PPP6R3 | 55291 | 0.4023834 | 4.7367265 | 1.15E-01 | 2.63E-01 |
| SLC30A1 | 7779 | 0.4023778 | 4.1916219 | 4.29E-01 | 6.26E-01 |
| RNF26 | 79102 | 0.4023526 | 4.1143483 | 3.04E-01 | 5.03E-01 |
| UBE2D2 | 7322 | 0.4022535 | 6.9331958 | 5.70E-02 | 1.61E-01 |
| COPS2 | 9318 | 0.4022224 | 5.7824186 | 6.32E-02 | 1.73E-01 |
| MRPL48 | 51642 | 0.4018844 | 6.7239375 | 1.28E-01 | 2.85E-01 |
| PSIP1 | 11168 | 0.4009984 | 7.7966637 | 1.33E-01 | 2.92E-01 |
| RABIF | 5877 | 0.4009511 | 6.2430178 | 1.22E-01 | 2.75E-01 |
| GLS | 2744 | 0.400775 | 5.3031069 | 4.70E-02 | 1.41E-01 |
| APEX1 | 328 | 0.4007528 | 7.7931364 | 3.56E-02 | 1.16E-01 |
| MGST2 | 4258 | 0.4007399 | 4.0266842 | 6.46E-02 | 1.76E-01 |
| KIAA0319L | 79932 | 0.4006763 | 5.676979 | 1.51E-01 | 3.17E-01 |
| CEP350 | 9857 | 0.4001715 | 5.3985626 | 7.46E-02 | 1.95E-01 |
| FARSB | 10056 | 0.4000458 | 7.5399791 | 1.15E-01 | 2.64E-01 |
| ZNF718 | 255403 | 0.3996313 | 3.0446957 | 1.45E-01 | 3.09E-01 |
| SF3A3 | 10946 | 0.3994847 | 6.3090331 | 3.13E-02 | 1.05E-01 |
| ZNF14 | 7561 | 0.3994456 | 4.4241729 | 7.39E-02 | 1.94E-01 |
| SEH1L | 81929 | 0.3994086 | 5.1921494 | 7.43E-02 | 1.94E-01 |
| EXOC6 | 54536 | 0.3993956 | 4.6513141 | 1.24E-01 | 2.78E-01 |
| DCAF11 | 80344 | 0.3991679 | 5.0268795 | 7.93E-02 | 2.03E-01 |
| GRAMD2A | 196996 | 0.3990376 | 0.603309 | 4.71E-01 | 6.60E-01 |
| SLC41A2 | 84102 | 0.3985098 | 3.5972675 | 9.60E-02 | 2.32E-01 |
| SEC22A | 26984 | 0.3981714 | 4.2825298 | 5.89E-02 | 1.65E-01 |
| TAF9 | 6880 | 0.3976117 | 7.958388 | 1.25E-01 | 2.80E-01 |
| OSBPL11 | 114885 | 0.3976041 | 4.0916017 | 3.54E-01 | 5.54E-01 |
| PPEF1 | 5475 | 0.3975604 | 1.6018747 | 5.23E-01 | 7.04E-01 |
| CBR1 | 873 | 0.3975206 | 6.6774418 | 1.88E-01 | 3.68E-01 |
| ORC6 | 23594 | 0.3974242 | 3.2319705 | 3.01E-01 | 5.00E-01 |
| TRAM2 | 9697 | 0.3973786 | 2.8915014 | 5.42E-01 | 7.17E-01 |
| RAB2A | 5862 | 0.3971908 | 9.2891079 | 9.99E-02 | 2.39E-01 |
| ADNP | 23394 | 0.3970616 | 5.5352743 | 1.42E-01 | 3.04E-01 |
| DPYSL2 | 1808 | 0.3970249 | 8.6036766 | 9.35E-02 | 2.28E-01 |
| TRMO | 51531 | 0.3969268 | 3.2121822 | 2.10E-01 | 3.96E-01 |
| YIPF6 | 286451 | 0.3967881 | 7.0325701 | 3.86E-02 | 1.22E-01 |
| SYCE3 | 644186 | 0.3967196 | 1.5620354 | 3.56E-01 | 5.55E-01 |
| SLC35A1 | 10559 | 0.3965913 | 4.0901706 | 2.09E-01 | 3.95E-01 |
| ZYG11B | 79699 | 0.3965586 | 6.0495429 | 2.54E-01 | 4.49E-01 |
| PRDX1 | 5052 | 0.3964855 | 8.5475346 | 7.86E-02 | 2.02E-01 |
| SLC39A14 | 23516 | 0.3963491 | 3.7365649 | 4.12E-01 | 6.11E-01 |
| ALG10 | 84920 | 0.3961773 | 1.4719299 | 3.00E-01 | 4.99E-01 |
| GABPA | 2551 | 0.3959091 | 3.5081949 | 1.64E-01 | 3.36E-01 |
| CRLF3 | 51379 | 0.3958941 | 3.3120712 | 2.58E-01 | 4.53E-01 |
| AP1AR | 55435 | 0.3955439 | 5.8865227 | 7.20E-02 | 1.91E-01 |
| THOC1 | 9984 | 0.3955368 | 4.6570522 | 5.99E-02 | 1.67E-01 |
| USP28 | 57646 | 0.3953679 | 3.1871519 | 3.96E-01 | 5.97E-01 |
| IPO13 | 9670 | 0.3952609 | 4.2697832 | 2.14E-01 | 4.02E-01 |
| YEATS4 | 8089 | 0.3951959 | 5.2144502 | 2.57E-01 | 4.51E-01 |
| TATDN1 | 83940 | 0.3951742 | 5.6411757 | 8.13E-02 | 2.07E-01 |
| MED8 | 112950 | 0.3949561 | 5.7867169 | 4.41E-02 | 1.35E-01 |
| NUDT7 | 283927 | 0.3946406 | 1.5309361 | 2.92E-01 | 4.91E-01 |
| ZNF251 | 90987 | 0.3945824 | 3.2826594 | 1.30E-01 | 2.87E-01 |
| SIKE1 | 80143 | 0.3945785 | 6.5219553 | 4.22E-02 | 1.31E-01 |
| CNIH4 | 29097 | 0.3945415 | 6.6850619 | 2.66E-02 | 9.34E-02 |
| UBTD2 | 92181 | 0.394445 | 5.2798884 | 4.25E-02 | 1.31E-01 |
| SHISA4 | 149345 | 0.3941577 | 6.6773161 | 3.64E-02 | 1.17E-01 |
| PPWD1 | 23398 | 0.3937903 | 4.1597282 | 1.86E-01 | 3.66E-01 |
| TMEM62 | 80021 | 0.3937479 | 4.0431513 | 4.43E-02 | 1.35E-01 |
| NCOA5 | 57727 | 0.3936762 | 4.8874638 | 4.81E-02 | 1.43E-01 |
| RPRD1A | 55197 | 0.3936318 | 5.7397899 | 3.29E-02 | 1.09E-01 |
| FBXO21 | 23014 | 0.3933326 | 7.2728635 | 2.67E-02 | 9.37E-02 |
| ZBBX | 79740 | 0.3932717 | 1.4374867 | 2.96E-01 | 4.95E-01 |
| TXNRD2 | 10587 | 0.3932362 | 2.2992676 | 4.63E-01 | 6.53E-01 |
| CA5B | 11238 | 0.3930055 | 2.5231538 | 1.58E-01 | 3.26E-01 |
| TDP1 | 55775 | 0.3929716 | 4.1706277 | 7.33E-02 | 1.93E-01 |
| VBP1 | 7411 | 0.3928598 | 8.2104386 | 1.54E-01 | 3.21E-01 |
| ANKS1A | 23294 | 0.3927825 | 5.1027996 | 1.56E-01 | 3.23E-01 |
| TARS1 | 6897 | 0.3927732 | 7.1040313 | 3.68E-02 | 1.18E-01 |
| NUP98 | 4928 | 0.3925987 | 4.0937642 | 2.11E-01 | 3.98E-01 |
| MAGOHB | 55110 | 0.3925556 | 5.103462 | 2.01E-01 | 3.86E-01 |
| CSNK1D | 1453 | 0.3924087 | 6.6883231 | 3.63E-02 | 1.17E-01 |
| HAUS1 | 115106 | 0.3923859 | 6.1339983 | 2.13E-01 | 4.01E-01 |
| ELAC1 | 55520 | 0.3923663 | 4.1778434 | 9.93E-02 | 2.38E-01 |
| SRRM1 | 10250 | 0.3921248 | 6.8086565 | 7.30E-02 | 1.92E-01 |
| FRS3 | 10817 | 0.392121 | 4.0808905 | 4.01E-01 | 6.02E-01 |
| CWC22 | 57703 | 0.3921166 | 5.179178 | 8.23E-02 | 2.09E-01 |
| TMF1 | 7110 | 0.3920825 | 5.4582342 | 4.15E-02 | 1.29E-01 |
| XRN2 | 22803 | 0.3917953 | 6.7202524 | 4.86E-02 | 1.44E-01 |
| MSANTD3 | 91283 | 0.3916526 | 6.3288464 | 3.26E-02 | 1.08E-01 |
| RABL2B | 11158 | 0.3913051 | 5.2822593 | 3.35E-02 | 1.11E-01 |
| ZFP1 | 162239 | 0.3910515 | 4.9470362 | 9.92E-02 | 2.38E-01 |
| PRPSAP1 | 5635 | 0.3908351 | 6.1543782 | 3.97E-02 | 1.25E-01 |
| CORO7 | 79585 | 0.390809 | 2.9337778 | 3.01E-01 | 4.99E-01 |
| XBP1 | 7494 | 0.3907641 | 7.4413534 | 1.53E-01 | 3.19E-01 |
| C5orf15 | 56951 | 0.3906123 | 6.5392588 | 1.21E-01 | 2.73E-01 |
| PML | 5371 | 0.3903914 | 2.7830815 | 2.02E-01 | 3.86E-01 |
| MIGA1 | 374986 | 0.3901893 | 4.1662458 | 7.21E-02 | 1.91E-01 |
| LIN7C | 55327 | 0.3898581 | 6.8007454 | 8.97E-02 | 2.21E-01 |
| NAP1L1 | 4673 | 0.3898191 | 7.381975 | 5.06E-02 | 1.48E-01 |
| KLHDC9 | 126823 | 0.3892847 | 5.172884 | 2.31E-01 | 4.22E-01 |
| COA7 | 65260 | 0.3891665 | 5.8478372 | 3.69E-01 | 5.68E-01 |
| ANAPC4 | 29945 | 0.3888368 | 4.8513053 | 4.74E-02 | 1.42E-01 |
| CBLN2 | 147381 | 0.3887362 | 4.5910414 | 4.98E-01 | 6.84E-01 |
| ANAPC13 | 25847 | 0.3887202 | 8.1086311 | 1.08E-01 | 2.52E-01 |
| ANKRD24 | 170961 | 0.3883376 | 0.5380932 | 5.06E-01 | 6.90E-01 |
| TEX10 | 54881 | 0.3882714 | 5.0247246 | 5.74E-02 | 1.62E-01 |
| EAPP | 55837 | 0.38823 | 6.7153032 | 3.10E-02 | 1.04E-01 |
| GART | 2618 | 0.3881116 | 5.9758989 | 1.09E-01 | 2.54E-01 |
| HDAC8 | 55869 | 0.3880867 | 3.6282462 | 1.67E-01 | 3.39E-01 |
| ZSCAN20 | 7579 | 0.3880574 | 1.7206632 | 5.08E-01 | 6.91E-01 |
| ZNF623 | 9831 | 0.3879997 | 4.1242448 | 8.82E-02 | 2.18E-01 |
| PRUNE2 | 158471 | 0.3879049 | 4.2347918 | 1.38E-01 | 2.99E-01 |
| MTM1 | 4534 | 0.3873067 | 3.4445108 | 2.30E-01 | 4.21E-01 |
| ERCC3 | 2071 | 0.3872275 | 5.4117575 | 1.28E-01 | 2.85E-01 |
| RNF214 | 257160 | 0.3870197 | 3.6648304 | 6.81E-02 | 1.82E-01 |
| SLU7 | 10569 | 0.3869247 | 6.6025776 | 3.13E-02 | 1.05E-01 |
| CTBP1 | 1487 | 0.3864873 | 5.5009811 | 9.99E-02 | 2.39E-01 |
| CLCN3 | 1182 | 0.3863429 | 6.0306066 | 3.54E-02 | 1.15E-01 |
| SMC1A | 8243 | 0.3860696 | 5.4085839 | 1.26E-01 | 2.80E-01 |
| NCBP2 | 22916 | 0.3860643 | 7.4301702 | 3.98E-02 | 1.25E-01 |
| NIPSNAP1 | 8508 | 0.3860386 | 7.9085745 | 7.81E-02 | 2.01E-01 |
| RSRC2 | 65117 | 0.3860014 | 6.73657 | 1.26E-01 | 2.81E-01 |
| CSNK2A1 | 1457 | 0.3859328 | 6.988161 | 3.41E-02 | 1.12E-01 |
| ZSCAN26 | 7741 | 0.3858599 | 5.0939611 | 7.41E-02 | 1.94E-01 |
| STPG1 | 90529 | 0.385806 | 4.7107868 | 8.83E-02 | 2.19E-01 |
| DDX20 | 11218 | 0.3856151 | 4.841373 | 4.91E-02 | 1.45E-01 |
| LETM2 | 137994 | 0.3855156 | 4.2102932 | 3.24E-01 | 5.25E-01 |
| KIAA1191 | 57179 | 0.3854574 | 7.4506841 | 3.14E-02 | 1.05E-01 |
| CWF19L1 | 55280 | 0.385303 | 4.0756465 | 1.06E-01 | 2.49E-01 |
| STRADB | 55437 | 0.385061 | 6.6980518 | 3.38E-02 | 1.11E-01 |
| PSMF1 | 9491 | 0.3847435 | 7.1851474 | 4.27E-02 | 1.32E-01 |
| RECQL5 | 9400 | 0.3845143 | 2.2227057 | 4.07E-01 | 6.07E-01 |
| CCT5 | 22948 | 0.3844216 | 8.4952473 | 3.46E-02 | 1.13E-01 |
| UTP14A | 10813 | 0.3842739 | 3.3277393 | 1.05E-01 | 2.47E-01 |
| EP300 | 2033 | 0.383925 | 4.505973 | 1.46E-01 | 3.10E-01 |
| NRBP1 | 29959 | 0.3838061 | 7.3873875 | 1.04E-01 | 2.45E-01 |
| RACK1 | 10399 | 0.3837302 | 8.8129211 | 7.62E-02 | 1.98E-01 |
| XPOT | 11260 | 0.3837007 | 5.8579693 | 2.31E-01 | 4.22E-01 |
| PMF1 | 11243 | 0.3836919 | 4.8368011 | 4.40E-02 | 1.35E-01 |
| RPS6KA2 | 6196 | 0.3836794 | 6.0948907 | 7.32E-02 | 1.92E-01 |
| TUT4 | 23318 | 0.383605 | 5.5984732 | 8.88E-02 | 2.19E-01 |
| IL20RB | 53833 | 0.3835952 | 0.3205086 | 4.28E-01 | 6.25E-01 |
| PCF11 | 51585 | 0.3835791 | 4.8518393 | 8.97E-02 | 2.21E-01 |
| ZNF202 | 7753 | 0.3834912 | 3.1840732 | 1.43E-01 | 3.05E-01 |
| TSPAN11 | 441631 | 0.3834635 | 3.1480747 | 4.16E-01 | 6.15E-01 |
| SNX7 | 51375 | 0.3832818 | 4.9623573 | 1.02E-01 | 2.42E-01 |
| CAMTA1 | 23261 | 0.3832535 | 7.3207275 | 3.00E-02 | 1.02E-01 |
| ISCU | 23479 | 0.3831421 | 7.805006 | 7.53E-02 | 1.96E-01 |
| B3GNT5 | 84002 | 0.3829865 | 2.4472182 | 4.86E-01 | 6.73E-01 |
| TRIM65 | 201292 | 0.382719 | 1.7413357 | 3.76E-01 | 5.75E-01 |
| CTDSP2 | 10106 | 0.3826909 | 2.7630502 | 5.27E-01 | 7.06E-01 |
| RNF114 | 55905 | 0.3825003 | 6.5231418 | 2.42E-01 | 4.34E-01 |
| EIF4B | 1975 | 0.3824189 | 5.7848595 | 1.17E-01 | 2.67E-01 |
| MEIG1 | 644890 | 0.3822376 | 1.4576925 | 4.38E-01 | 6.32E-01 |
| FNIP2 | 57600 | 0.3821906 | 5.6696304 | 2.36E-01 | 4.27E-01 |
| MAP4K5 | 11183 | 0.3821741 | 5.8074282 | 1.11E-01 | 2.58E-01 |
| PM20D2 | 135293 | 0.382072 | 3.3254435 | 1.67E-01 | 3.39E-01 |
| MAP1LC3B | 81631 | 0.3817609 | 9.2835406 | 3.87E-02 | 1.22E-01 |
| PLA2G4C | 8605 | 0.3817244 | 3.5730732 | 9.55E-02 | 2.31E-01 |
| CLYBL | 171425 | 0.3815013 | 2.4357783 | 2.29E-01 | 4.20E-01 |
| RAB7A | 7879 | 0.3814596 | 8.9896259 | 3.70E-02 | 1.19E-01 |
| VEZF1 | 7716 | 0.3807345 | 5.5794626 | 5.34E-02 | 1.54E-01 |
| CEP70 | 80321 | 0.3805069 | 4.9510154 | 8.77E-02 | 2.17E-01 |
| PWWP2A | 114825 | 0.3803715 | 4.6959931 | 1.39E-01 | 3.00E-01 |
| STK36 | 27148 | 0.3800969 | 2.5338971 | 4.37E-01 | 6.32E-01 |
| BRD8 | 10902 | 0.38003 | 5.6552192 | 4.55E-02 | 1.38E-01 |
| ERCC8 | 1161 | 0.3799385 | 4.5417584 | 4.81E-02 | 1.43E-01 |
| PANX1 | 24145 | 0.3798868 | 4.7905407 | 8.04E-02 | 2.05E-01 |
| MIOS | 54468 | 0.379781 | 4.1295092 | 1.44E-01 | 3.06E-01 |
| VPS33A | 65082 | 0.379668 | 3.7225408 | 9.81E-02 | 2.36E-01 |
| PALLD | 23022 | 0.3794458 | 6.4269705 | 4.56E-01 | 6.48E-01 |
| ZBTB49 | 166793 | 0.3791084 | 2.9545941 | 1.99E-01 | 3.83E-01 |
| SUCLA2 | 8803 | 0.3787874 | 7.8216741 | 6.83E-02 | 1.83E-01 |
| ZNF300 | 91975 | 0.3786062 | 5.2069104 | 1.70E-01 | 3.43E-01 |
| EIF4E3 | 317649 | 0.3783687 | 5.1821677 | 1.40E-01 | 3.02E-01 |
| PECR | 55825 | 0.3782859 | 3.2580949 | 1.95E-01 | 3.78E-01 |
| TMX1 | 81542 | 0.3782473 | 5.5140585 | 2.83E-01 | 4.80E-01 |
| PIDD1 | 55367 | 0.3778536 | 2.1261077 | 4.49E-01 | 6.42E-01 |
| PRMT3 | 10196 | 0.3778511 | 3.8544567 | 3.53E-01 | 5.52E-01 |
| C9orf116 | 138162 | 0.3776866 | 4.6525585 | 3.87E-02 | 1.23E-01 |
| GSTM4 | 2948 | 0.3774895 | 5.2214796 | 1.18E-01 | 2.69E-01 |
| TMOD3 | 29766 | 0.3773644 | 5.4598901 | 8.45E-02 | 2.12E-01 |
| MKNK1 | 8569 | 0.3770051 | 3.1383756 | 3.00E-01 | 4.99E-01 |
| SPIN1 | 10927 | 0.3769178 | 6.7864805 | 3.42E-01 | 5.43E-01 |
| CDH24 | 64403 | 0.3768058 | 2.1618418 | 2.69E-01 | 4.65E-01 |
| ZWILCH | 55055 | 0.3767125 | 4.7155284 | 1.57E-01 | 3.24E-01 |
| CCT6A | 908 | 0.3766196 | 8.3754056 | 4.35E-02 | 1.33E-01 |
| UBC | 7316 | 0.3765089 | 11.858572 | 9.90E-02 | 2.37E-01 |
| PSPC1 | 55269 | 0.3763497 | 5.5356577 | 4.43E-02 | 1.35E-01 |
| MAN2B2 | 23324 | 0.3758996 | 3.497638 | 3.02E-01 | 5.01E-01 |
| C11orf58 | 10944 | 0.3756196 | 8.8201909 | 5.92E-02 | 1.66E-01 |
| ASXL3 | 80816 | 0.375385 | 4.2983571 | 4.77E-01 | 6.66E-01 |
| PKM | 5315 | 0.3753586 | 8.8010396 | 4.37E-01 | 6.32E-01 |
| DNM1L | 10059 | 0.3750941 | 6.7741685 | 6.38E-02 | 1.74E-01 |
| PIKFYVE | 200576 | 0.3750919 | 3.6578062 | 9.41E-02 | 2.29E-01 |
| ZKSCAN3 | 80317 | 0.3750727 | 3.0512482 | 1.51E-01 | 3.17E-01 |
| UGCG | 7357 | 0.3750192 | 5.5719989 | 3.69E-01 | 5.68E-01 |
| MRPL22 | 29093 | 0.3749282 | 7.299577 | 9.08E-02 | 2.23E-01 |
| STAT1 | 6772 | 0.3748346 | 4.4574948 | 3.04E-01 | 5.03E-01 |
| SCYL3 | 57147 | 0.3746843 | 2.7807116 | 2.14E-01 | 4.02E-01 |
| NEK4 | 6787 | 0.3746808 | 4.1199196 | 1.78E-01 | 3.55E-01 |
| ZNF436 | 80818 | 0.3746791 | 4.7769759 | 1.23E-01 | 2.76E-01 |
| STAT6 | 6778 | 0.3746658 | 0.7793676 | 4.88E-01 | 6.75E-01 |
| ARMC8 | 25852 | 0.374658 | 6.603976 | 5.39E-02 | 1.55E-01 |
| TANK | 10010 | 0.3742828 | 6.2686297 | 4.64E-02 | 1.40E-01 |
| FNBP1 | 23048 | 0.374261 | 5.2366738 | 6.16E-02 | 1.70E-01 |
| SHLD3 | 112441434 | 0.3741528 | 3.6872436 | 1.41E-01 | 3.02E-01 |
| FBXW2 | 26190 | 0.3738928 | 5.0754787 | 1.23E-01 | 2.76E-01 |
| NSMCE2 | 286053 | 0.3738724 | 5.6318437 | 9.65E-02 | 2.33E-01 |
| NBPF15 | 284565 | 0.3738229 | 4.0051711 | 1.85E-01 | 3.64E-01 |
| TTC39C | 125488 | 0.3734869 | 2.9677086 | 2.05E-01 | 3.90E-01 |
| TERF2 | 7014 | 0.3734532 | 5.0697806 | 1.02E-01 | 2.42E-01 |
| MPDZ | 8777 | 0.3733245 | 4.3589192 | 1.27E-01 | 2.82E-01 |
| POLR2K | 5440 | 0.3732484 | 7.9298609 | 1.45E-01 | 3.09E-01 |
| COPRS | 55352 | 0.3730197 | 7.6989598 | 7.11E-02 | 1.89E-01 |
| MXD1 | 4084 | 0.3726074 | 3.4791461 | 1.84E-01 | 3.63E-01 |
| ADPGK | 83440 | 0.3723272 | 5.009047 | 2.71E-01 | 4.67E-01 |
| SPATA5L1 | 79029 | 0.3722358 | 3.0463989 | 1.48E-01 | 3.13E-01 |
| NXF1 | 10482 | 0.3721489 | 5.801086 | 1.08E-01 | 2.53E-01 |
| PPAN | 56342 | 0.3720927 | 0.5290881 | 3.55E-01 | 5.54E-01 |
| MARCHF8 | 220972 | 0.3718834 | 3.0422787 | 2.03E-01 | 3.87E-01 |
| MINDY2 | 54629 | 0.3718314 | 6.3307265 | 7.23E-02 | 1.91E-01 |
| PDXDC1 | 23042 | 0.3717254 | 5.799962 | 8.57E-02 | 2.14E-01 |
| SLTM | 79811 | 0.3710875 | 6.7701372 | 1.36E-01 | 2.95E-01 |
| C2orf69 | 205327 | 0.3707172 | 5.6913744 | 2.36E-01 | 4.28E-01 |
| EXOC2 | 55770 | 0.3707134 | 5.0520569 | 1.18E-01 | 2.68E-01 |
| SPTLC2 | 9517 | 0.3705794 | 4.2418536 | 1.11E-01 | 2.57E-01 |
| SMARCD1 | 6602 | 0.3704294 | 3.9835084 | 1.34E-01 | 2.92E-01 |
| BTBD1 | 53339 | 0.3703545 | 6.639867 | 6.30E-02 | 1.73E-01 |
| COTL1 | 23406 | 0.3701105 | 9.1441532 | 2.83E-01 | 4.80E-01 |
| STARD3NL | 83930 | 0.3700691 | 8.4738976 | 1.71E-01 | 3.45E-01 |
| MAN1A2 | 10905 | 0.3699847 | 6.2585834 | 2.42E-01 | 4.34E-01 |
| SUSD6 | 9766 | 0.3698629 | 2.3732116 | 3.75E-01 | 5.74E-01 |
| RPS6KA6 | 27330 | 0.3696233 | 3.0192964 | 2.34E-01 | 4.26E-01 |
| ZNF655 | 79027 | 0.3695661 | 5.0645198 | 1.33E-01 | 2.92E-01 |
| EPB41L3 | 23136 | 0.3693752 | 5.7323516 | 5.88E-02 | 1.65E-01 |
| ZNF223 | 7766 | 0.3693523 | 1.5966646 | 3.02E-01 | 5.01E-01 |
| REXO4 | 57109 | 0.369303 | 4.8096259 | 6.66E-02 | 1.80E-01 |
| AADAT | 51166 | 0.3692147 | 4.9820161 | 3.07E-01 | 5.07E-01 |
| BCAR1 | 9564 | 0.3688071 | 5.3030832 | 3.82E-01 | 5.82E-01 |
| PEA15 | 8682 | 0.3687372 | 6.1395137 | 1.24E-01 | 2.78E-01 |
| EPHX2 | 2053 | 0.3686174 | 2.7615163 | 3.11E-01 | 5.11E-01 |
| ABITRAM | 54942 | 0.3684947 | 4.7900359 | 2.42E-01 | 4.33E-01 |
| PPP2R3A | 5523 | 0.3684818 | 5.3838354 | 2.19E-01 | 4.07E-01 |
| SAE1 | 10055 | 0.3683919 | 7.3559293 | 4.60E-02 | 1.39E-01 |
| SIGMAR1 | 10280 | 0.3682866 | 7.1507276 | 1.25E-01 | 2.79E-01 |
| ZNF35 | 7584 | 0.3680675 | 3.7163097 | 1.22E-01 | 2.74E-01 |
| XRRA1 | 143570 | 0.367996 | 2.8036736 | 2.61E-01 | 4.57E-01 |
| IFT57 | 55081 | 0.3679357 | 7.3353342 | 9.05E-02 | 2.22E-01 |
| JAZF1 | 221895 | 0.3678796 | 4.643328 | 5.86E-02 | 1.65E-01 |
| OGFOD1 | 55239 | 0.3678539 | 5.2580845 | 1.51E-01 | 3.16E-01 |
| ANO4 | 121601 | 0.3677219 | 3.1753637 | 5.10E-01 | 6.93E-01 |
| ZNF574 | 64763 | 0.3674202 | 4.8522147 | 8.09E-02 | 2.06E-01 |
| DDHD1 | 80821 | 0.3672443 | 4.5003446 | 5.42E-02 | 1.56E-01 |
| ZBTB22 | 9278 | 0.367155 | 3.9143575 | 4.53E-01 | 6.45E-01 |
| POGLUT1 | 56983 | 0.3669926 | 4.4787328 | 1.50E-01 | 3.15E-01 |
| USP12 | 219333 | 0.3660599 | 4.4294928 | 1.55E-01 | 3.22E-01 |
| ADAL | 161823 | 0.3657169 | 4.9190245 | 1.18E-01 | 2.68E-01 |
| MOAP1 | 64112 | 0.365694 | 7.7972715 | 7.11E-02 | 1.89E-01 |
| ARMC9 | 80210 | 0.3656853 | 4.3140842 | 3.11E-01 | 5.11E-01 |
| DIS3 | 22894 | 0.3654892 | 5.6069839 | 5.22E-02 | 1.51E-01 |
| CBWD3 | 445571 | 0.3654645 | 3.0465292 | 1.87E-01 | 3.66E-01 |
| MAP3K4 | 4216 | 0.3653982 | 2.6740845 | 3.55E-01 | 5.54E-01 |
| H2AC20 | 8338 | 0.3653589 | 0.6360369 | 5.13E-01 | 6.95E-01 |
| PCDHB4 | 56131 | 0.365227 | 0.1459902 | 5.00E-01 | 6.86E-01 |
| MANBA | 4126 | 0.3652103 | 3.9771938 | 3.95E-01 | 5.95E-01 |
| SEC24B | 10427 | 0.3651587 | 4.9410171 | 1.14E-01 | 2.62E-01 |
| SRP68 | 6730 | 0.365134 | 5.9792235 | 2.25E-01 | 4.14E-01 |
| ALS2 | 57679 | 0.3648684 | 4.2832093 | 1.58E-01 | 3.26E-01 |
| MRPL32 | 64983 | 0.3647012 | 7.2912667 | 6.48E-02 | 1.76E-01 |
| GARS1 | 2617 | 0.3646258 | 7.9210367 | 4.84E-02 | 1.43E-01 |
| ARHGEF9 | 23229 | 0.3645963 | 5.0602137 | 9.55E-02 | 2.31E-01 |
| CANX | 821 | 0.3645772 | 8.718434 | 2.87E-01 | 4.85E-01 |
| RPS6 | 6194 | 0.3643439 | 8.152718 | 1.20E-01 | 2.71E-01 |
| SREBF2 | 6721 | 0.3643289 | 5.9470908 | 2.25E-01 | 4.14E-01 |
| ZNF669 | 79862 | 0.3641071 | 4.7055784 | 2.28E-01 | 4.19E-01 |
| SOCS5 | 9655 | 0.3640553 | 4.1677155 | 1.95E-01 | 3.78E-01 |
| YIPF1 | 54432 | 0.363935 | 5.5037546 | 7.82E-02 | 2.01E-01 |
| MFAP2 | 4237 | 0.363742 | 5.3598655 | 8.76E-02 | 2.17E-01 |
| CCL3 | 6348 | 0.3632708 | 0.6069082 | 4.29E-01 | 6.26E-01 |
| USP3 | 9960 | 0.3632217 | 4.9655117 | 2.16E-01 | 4.04E-01 |
| RFLNB | 359845 | 0.363161 | 2.2676107 | 4.68E-01 | 6.58E-01 |
| NUDT21 | 11051 | 0.3631004 | 4.8558917 | 2.04E-01 | 3.89E-01 |
| CAVIN4 | 347273 | 0.3630419 | 2.0639973 | 5.29E-01 | 7.08E-01 |
| KCMF1 | 56888 | 0.3628535 | 6.0831261 | 3.68E-02 | 1.18E-01 |
| MTMR14 | 64419 | 0.3626107 | 4.6122612 | 1.51E-01 | 3.17E-01 |
| GMFB | 2764 | 0.3625184 | 7.9593051 | 1.76E-01 | 3.52E-01 |
| MAP1A | 4130 | 0.362183 | 8.9043192 | 1.53E-01 | 3.19E-01 |
| TBC1D22B | 55633 | 0.3621194 | 3.5718829 | 1.91E-01 | 3.72E-01 |
| SNX2 | 6643 | 0.3620464 | 6.9682382 | 5.88E-02 | 1.65E-01 |
| CD59 | 966 | 0.3613326 | 7.2004885 | 2.94E-01 | 4.92E-01 |
| TNFSF9 | 8744 | 0.3610675 | 2.6156028 | 2.65E-01 | 4.61E-01 |
| FZD1 | 8321 | 0.3605883 | 2.3034213 | 4.03E-01 | 6.03E-01 |
| TBC1D9 | 23158 | 0.3605606 | 5.1460043 | 2.13E-01 | 4.00E-01 |
| PLB1 | 151056 | 0.3603969 | 0.6455089 | 5.02E-01 | 6.87E-01 |
| SLC25A18 | 83733 | 0.360226 | 1.356757 | 5.08E-01 | 6.91E-01 |
| NFS1 | 9054 | 0.3601094 | 4.609219 | 1.18E-01 | 2.69E-01 |
| ZNF543 | 125919 | 0.3600253 | 2.5538682 | 2.19E-01 | 4.07E-01 |
| ACADSB | 36 | 0.3599624 | 3.8422701 | 1.80E-01 | 3.58E-01 |
| TMEM214 | 54867 | 0.3599376 | 3.8828855 | 4.37E-01 | 6.32E-01 |
| SF3B4 | 10262 | 0.3597391 | 6.6796298 | 6.17E-02 | 1.71E-01 |
| CGRRF1 | 10668 | 0.3596338 | 5.3387425 | 5.33E-02 | 1.54E-01 |
| ASIC3 | 9311 | 0.3595722 | 0.8343684 | 5.20E-01 | 7.01E-01 |
| C5orf34 | 375444 | 0.3593619 | 0.7849044 | 4.85E-01 | 6.72E-01 |
| ABRAXAS1 | 84142 | 0.3589478 | 1.242904 | 4.20E-01 | 6.19E-01 |
| FAM151B | 167555 | 0.358658 | 1.86746 | 3.72E-01 | 5.71E-01 |
| SNX3 | 8724 | 0.3586429 | 8.5105433 | 4.89E-02 | 1.44E-01 |
| BTBD3 | 22903 | 0.3584759 | 5.8936686 | 2.83E-01 | 4.81E-01 |
| GPX3 | 2878 | 0.3584192 | 6.7217879 | 2.09E-01 | 3.95E-01 |
| DIO3 | 1735 | 0.3583989 | 5.9265503 | 5.36E-01 | 7.12E-01 |
| ZNF460 | 10794 | 0.3582641 | 2.7648389 | 2.98E-01 | 4.97E-01 |
| ZCCHC3 | 85364 | 0.3582013 | 3.0812073 | 2.07E-01 | 3.92E-01 |
| POU2F1 | 5451 | 0.3580255 | 4.823267 | 1.36E-01 | 2.95E-01 |
| FAXC | 84553 | 0.3578606 | 6.0036253 | 4.37E-01 | 6.32E-01 |
| SLBP | 7884 | 0.3575521 | 6.7280255 | 6.49E-02 | 1.76E-01 |
| ENDOD1 | 23052 | 0.3572372 | 4.7639918 | 2.23E-01 | 4.12E-01 |
| FAAH2 | 158584 | 0.3572316 | 2.4262756 | 5.35E-01 | 7.12E-01 |
| CYRIB | 51571 | 0.3571401 | 7.6299318 | 1.17E-01 | 2.67E-01 |
| CNOT1 | 23019 | 0.3570224 | 3.3766321 | 3.42E-01 | 5.43E-01 |
| BLOC1S5 | 63915 | 0.3569908 | 3.6433323 | 1.54E-01 | 3.20E-01 |
| DHDH | 27294 | 0.3567088 | 1.8267558 | 4.66E-01 | 6.56E-01 |
| DROSHA | 29102 | 0.3567072 | 3.5971167 | 1.96E-01 | 3.78E-01 |
| COPS8 | 10920 | 0.3563667 | 8.5256809 | 8.09E-02 | 2.06E-01 |
| ARRDC3 | 57561 | 0.3562194 | 5.1645115 | 4.25E-01 | 6.23E-01 |
| ZNF135 | 7694 | 0.3561604 | 3.0589424 | 3.24E-01 | 5.24E-01 |
| FBH1 | 84893 | 0.3561102 | 4.6217495 | 1.91E-01 | 3.72E-01 |
| NUP37 | 79023 | 0.3559409 | 3.4053902 | 2.29E-01 | 4.20E-01 |
| SCML4 | 256380 | 0.3558263 | 2.9023448 | 5.01E-01 | 6.87E-01 |
| VPS37A | 137492 | 0.3555994 | 5.8486355 | 5.42E-02 | 1.56E-01 |
| AKAP8 | 10270 | 0.3554301 | 3.8917473 | 1.30E-01 | 2.88E-01 |
| SRSF12 | 135295 | 0.3553818 | 5.3385634 | 2.87E-01 | 4.85E-01 |
| TMEM126B | 55863 | 0.3553663 | 6.7899808 | 1.17E-01 | 2.67E-01 |
| FNBP4 | 23360 | 0.3552332 | 4.9691399 | 1.65E-01 | 3.36E-01 |
| UBE2D3 | 7323 | 0.3552235 | 8.8012958 | 4.90E-02 | 1.45E-01 |
| KLC2 | 64837 | 0.3550805 | 4.2892274 | 1.47E-01 | 3.11E-01 |
| SEPTIN8 | 23176 | 0.3550162 | 2.3582021 | 2.54E-01 | 4.48E-01 |
| RBM45 | 129831 | 0.3547444 | 3.9047238 | 1.39E-01 | 2.99E-01 |
| PKNOX1 | 5316 | 0.3546077 | 4.5248686 | 1.88E-01 | 3.68E-01 |
| CHCHD4 | 131474 | 0.3545965 | 5.5383001 | 1.83E-01 | 3.61E-01 |
| PITHD1 | 57095 | 0.3545945 | 7.3160389 | 1.26E-01 | 2.80E-01 |
| ARHGEF12 | 23365 | 0.3545488 | 6.5066655 | 5.07E-02 | 1.48E-01 |
| DOCK7 | 85440 | 0.3545466 | 5.4641543 | 7.97E-02 | 2.04E-01 |
| RNF216 | 54476 | 0.3543386 | 5.8177122 | 1.35E-01 | 2.95E-01 |
| SLC30A8 | 169026 | 0.3541414 | 3.4338328 | 1.51E-01 | 3.16E-01 |
| ADD1 | 118 | 0.3540756 | 6.5884656 | 8.19E-02 | 2.08E-01 |
| DHX9 | 1660 | 0.3540334 | 6.4441181 | 1.39E-01 | 3.00E-01 |
| GOLPH3L | 55204 | 0.3533836 | 3.92497 | 2.54E-01 | 4.48E-01 |
| GULP1 | 51454 | 0.3529134 | 3.3057911 | 3.18E-01 | 5.18E-01 |
| SUPV3L1 | 6832 | 0.3526705 | 5.3188597 | 5.01E-02 | 1.47E-01 |
| C1orf56 | 54964 | 0.3526559 | 3.3208936 | 2.55E-01 | 4.49E-01 |
| SGCE | 8910 | 0.3524312 | 6.5322935 | 1.15E-01 | 2.64E-01 |
| IST1 | 9798 | 0.3524102 | 7.0900566 | 1.01E-01 | 2.40E-01 |
| SLC4A8 | 9498 | 0.3522972 | 5.0921786 | 1.54E-01 | 3.21E-01 |
| CFLAR | 8837 | 0.3522481 | 3.991841 | 3.67E-01 | 5.66E-01 |
| SLC25A40 | 55972 | 0.3519732 | 3.4989806 | 1.32E-01 | 2.90E-01 |
| OLA1 | 29789 | 0.3518334 | 7.903328 | 1.47E-01 | 3.10E-01 |
| PHF3 | 23469 | 0.3517234 | 5.7889251 | 7.38E-02 | 1.94E-01 |
| GTF2B | 2959 | 0.3511098 | 6.125476 | 1.22E-01 | 2.75E-01 |
| GRPEL2 | 134266 | 0.3505108 | 4.781472 | 7.60E-02 | 1.98E-01 |
| LIG1 | 3978 | 0.3501392 | 3.2789442 | 1.76E-01 | 3.51E-01 |
| IWS1 | 55677 | 0.3500731 | 5.9124748 | 4.76E-02 | 1.42E-01 |
| CEBPZ | 10153 | 0.3499495 | 5.5944225 | 5.71E-02 | 1.61E-01 |
| TFE3 | 7030 | 0.3497955 | 3.7449251 | 2.91E-01 | 4.90E-01 |
| ASB12 | 142689 | 0.3497079 | 1.1173083 | 4.10E-01 | 6.10E-01 |
| PTGES3 | 10728 | 0.3494292 | 9.0191675 | 6.97E-02 | 1.86E-01 |
| CTBP2 | 1488 | 0.3492975 | 5.4187223 | 9.10E-02 | 2.23E-01 |
| LMNA | 4000 | 0.3490286 | 6.712719 | 4.33E-01 | 6.29E-01 |
| ALG1L | 200810 | 0.3490269 | 0.1285413 | 5.37E-01 | 7.14E-01 |
| CREB5 | 9586 | 0.3490191 | 3.2737361 | 3.25E-01 | 5.26E-01 |
| ZNF326 | 284695 | 0.3488878 | 5.9940994 | 5.07E-02 | 1.48E-01 |
| GSTO2 | 119391 | 0.3485049 | 3.303314 | 3.71E-01 | 5.70E-01 |
| CPOX | 1371 | 0.3484732 | 3.2575879 | 1.85E-01 | 3.64E-01 |
| NKIRAS2 | 28511 | 0.3483574 | 7.5165831 | 5.69E-02 | 1.61E-01 |
| CEP78 | 84131 | 0.3482138 | 4.6763217 | 1.25E-01 | 2.79E-01 |
| GPR89A | 653519 | 0.3480874 | 4.1948017 | 1.09E-01 | 2.54E-01 |
| FER | 2241 | 0.3479685 | 5.127207 | 1.29E-01 | 2.85E-01 |
| TAF2 | 6873 | 0.3478548 | 5.6745671 | 7.51E-02 | 1.96E-01 |
| BRF2 | 55290 | 0.3476327 | 2.9371983 | 2.75E-01 | 4.72E-01 |
| RLIM | 51132 | 0.3476119 | 4.9355496 | 1.84E-01 | 3.63E-01 |
| GET1 | 7485 | 0.3475842 | 8.4448633 | 3.02E-01 | 5.01E-01 |
| SETDB2 | 83852 | 0.3475169 | 3.3521561 | 2.18E-01 | 4.07E-01 |
| R3HDM1 | 23518 | 0.3475166 | 5.7949828 | 3.94E-01 | 5.94E-01 |
| SND1 | 27044 | 0.3471538 | 5.943641 | 3.27E-01 | 5.28E-01 |
| CRMP1 | 1400 | 0.3470754 | 10.316033 | 4.76E-01 | 6.65E-01 |
| PAAF1 | 80227 | 0.3469552 | 5.594501 | 7.76E-02 | 2.00E-01 |
| CHIC2 | 26511 | 0.3467253 | 5.4255419 | 1.19E-01 | 2.70E-01 |
| GORASP2 | 26003 | 0.3465863 | 7.3317745 | 1.14E-01 | 2.62E-01 |
| CNOT7 | 29883 | 0.3465849 | 7.6322154 | 7.67E-02 | 1.99E-01 |
| PLAAT1 | 57110 | 0.3465253 | 2.5013726 | 2.38E-01 | 4.30E-01 |
| HARBI1 | 283254 | 0.3461226 | 3.6248181 | 1.09E-01 | 2.55E-01 |
| SCLY | 51540 | 0.3460303 | 1.0101571 | 3.64E-01 | 5.63E-01 |
| LRRIQ1 | 84125 | 0.3459897 | 3.3457369 | 4.06E-01 | 6.07E-01 |
| UNG | 7374 | 0.3458367 | 5.2126113 | 8.67E-02 | 2.16E-01 |
| PDE12 | 201626 | 0.345625 | 4.4504227 | 1.36E-01 | 2.96E-01 |
| PCLO | 27445 | 0.3455108 | 6.2397421 | 5.40E-01 | 7.16E-01 |
| DNAJB14 | 79982 | 0.3455041 | 5.4371735 | 1.35E-01 | 2.94E-01 |
| MRPS6 | 64968 | 0.345471 | 7.5537834 | 6.13E-02 | 1.70E-01 |
| PARP2 | 10038 | 0.3453481 | 6.5353275 | 1.98E-01 | 3.82E-01 |
| HMG20A | 10363 | 0.3453102 | 5.1788571 | 7.74E-02 | 2.00E-01 |
| GGNBP2 | 79893 | 0.345107 | 7.3395972 | 6.19E-02 | 1.71E-01 |
| PELO | 53918 | 0.3447798 | 5.4646796 | 1.77E-01 | 3.53E-01 |
| TRAFD1 | 10906 | 0.3447 | 5.9830143 | 6.34E-02 | 1.74E-01 |
| EPHB2 | 2048 | 0.344676 | 5.6199187 | 2.62E-01 | 4.57E-01 |
| AMY2B | 280 | 0.3446394 | 0.2819765 | 5.40E-01 | 7.16E-01 |
| RNLS | 55328 | 0.3446232 | 3.7677374 | 1.79E-01 | 3.57E-01 |
| THUMPD1 | 55623 | 0.3442688 | 5.7449526 | 1.00E-01 | 2.39E-01 |
| MMAB | 326625 | 0.3442223 | 5.7509675 | 9.20E-02 | 2.25E-01 |
| ZNF485 | 220992 | 0.3440267 | 2.742323 | 3.24E-01 | 5.25E-01 |
| PIGS | 94005 | 0.3438688 | 5.7859588 | 2.60E-01 | 4.55E-01 |
| VPS45 | 11311 | 0.343717 | 6.2278154 | 9.47E-02 | 2.29E-01 |
| KBTBD6 | 89890 | 0.3437016 | 6.4396927 | 2.56E-01 | 4.50E-01 |
| TMEM216 | 51259 | 0.3436 | 4.3150749 | 9.56E-02 | 2.31E-01 |
| WDFY2 | 115825 | 0.3432564 | 3.5896496 | 3.44E-01 | 5.44E-01 |
| ETFBKMT | 254013 | 0.3429871 | 2.9552436 | 2.98E-01 | 4.97E-01 |
| THNSL1 | 79896 | 0.3429652 | 3.938028 | 1.51E-01 | 3.16E-01 |
| CLTA | 1211 | 0.3427932 | 8.5280663 | 6.04E-02 | 1.68E-01 |
| EHD3 | 30845 | 0.3426987 | 4.3315975 | 2.32E-01 | 4.23E-01 |
| KLF8 | 11279 | 0.3426424 | 2.9987394 | 2.48E-01 | 4.41E-01 |
| EXOSC8 | 11340 | 0.3424648 | 5.9396155 | 1.84E-01 | 3.63E-01 |
| ATXN3 | 4287 | 0.3424397 | 4.5216738 | 1.39E-01 | 3.00E-01 |
| TRIP6 | 7205 | 0.3423118 | 4.1462185 | 5.26E-01 | 7.06E-01 |
| TVP23C | 201158 | 0.3418679 | 1.2399961 | 3.64E-01 | 5.63E-01 |
| SCAF1 | 58506 | 0.3416414 | 2.1271838 | 3.40E-01 | 5.41E-01 |
| NUP188 | 23511 | 0.3415098 | 3.26823 | 3.73E-01 | 5.72E-01 |
| NAA60 | 79903 | 0.3415037 | 4.9546352 | 2.19E-01 | 4.08E-01 |
| YAE1 | 57002 | 0.3414948 | 5.9067775 | 9.80E-02 | 2.36E-01 |
| SLX4IP | 128710 | 0.3414789 | 4.4218648 | 2.04E-01 | 3.89E-01 |
| ZFX | 7543 | 0.3412436 | 4.9100916 | 1.66E-01 | 3.38E-01 |
| CACFD1 | 11094 | 0.3411651 | 4.9925626 | 1.90E-01 | 3.71E-01 |
| NCDN | 23154 | 0.3410247 | 5.7045187 | 3.30E-01 | 5.31E-01 |
| IDNK | 414328 | 0.3407936 | 3.6047195 | 2.09E-01 | 3.96E-01 |
| NT5DC1 | 221294 | 0.3405778 | 3.010563 | 4.40E-01 | 6.34E-01 |
| ZFP82 | 284406 | 0.3404086 | 5.7869251 | 1.34E-01 | 2.93E-01 |
| TUT1 | 64852 | 0.3403845 | 2.649167 | 4.49E-01 | 6.43E-01 |
| RNF138 | 51444 | 0.3403542 | 4.9401465 | 2.00E-01 | 3.84E-01 |
| FBXW8 | 26259 | 0.3403202 | 3.0192375 | 2.86E-01 | 4.85E-01 |
| POM121 | 9883 | 0.3402584 | 4.3112248 | 1.44E-01 | 3.07E-01 |
| LATS1 | 9113 | 0.3401497 | 3.9587291 | 1.52E-01 | 3.17E-01 |
| BNIP3L | 665 | 0.3401345 | 7.8503458 | 1.90E-01 | 3.71E-01 |
| ZNF595 | 152687 | 0.3400349 | 3.5168492 | 2.19E-01 | 4.07E-01 |
| PGBD2 | 267002 | 0.3398852 | 2.5224628 | 1.97E-01 | 3.80E-01 |
| NBPF12 | 149013 | 0.33986 | 2.5095519 | 2.21E-01 | 4.10E-01 |
| UFSP2 | 55325 | 0.3393404 | 5.5979133 | 6.68E-02 | 1.80E-01 |
| SPEN | 23013 | 0.3392499 | 5.9991602 | 1.10E-01 | 2.56E-01 |
| CCDC74B | 91409 | 0.3390377 | 4.1115352 | 1.36E-01 | 2.95E-01 |
| ZNF287 | 57336 | 0.3388928 | 4.2501403 | 1.65E-01 | 3.36E-01 |
| AAR2 | 25980 | 0.3384085 | 5.5023763 | 1.49E-01 | 3.14E-01 |
| CETN3 | 1070 | 0.3383631 | 5.3595234 | 1.83E-01 | 3.61E-01 |
| RBPMS2 | 348093 | 0.3382161 | 0.2583716 | 5.01E-01 | 6.87E-01 |
| SNX30 | 401548 | 0.3380509 | 3.5534962 | 2.56E-01 | 4.50E-01 |
| STRN4 | 29888 | 0.3379728 | 6.370381 | 1.07E-01 | 2.51E-01 |
| NOL9 | 79707 | 0.3379564 | 4.5770143 | 1.24E-01 | 2.78E-01 |
| PBX4 | 80714 | 0.3379561 | 2.3536952 | 2.87E-01 | 4.85E-01 |
| DPH6 | 89978 | 0.33726 | 3.5107924 | 1.85E-01 | 3.65E-01 |
| ZNF441 | 126068 | 0.3370548 | 3.4419626 | 1.70E-01 | 3.43E-01 |
| SLC11A2 | 4891 | 0.3370005 | 5.4064025 | 2.21E-01 | 4.10E-01 |
| RINT1 | 60561 | 0.3369386 | 4.2612563 | 1.38E-01 | 2.99E-01 |
| SOS1 | 6654 | 0.3367579 | 4.6339352 | 1.12E-01 | 2.58E-01 |
| FAM214B | 80256 | 0.3367564 | 4.2823797 | 2.53E-01 | 4.47E-01 |
| SNX5 | 27131 | 0.3363936 | 5.2061911 | 1.86E-01 | 3.65E-01 |
| NFE2L1 | 4779 | 0.3363633 | 4.708072 | 2.47E-01 | 4.40E-01 |
| EXO5 | 64789 | 0.3363305 | 2.8980836 | 2.91E-01 | 4.90E-01 |
| ADD2 | 119 | 0.336309 | 6.3534411 | 5.24E-01 | 7.04E-01 |
| SCAF11 | 9169 | 0.3362314 | 5.612886 | 1.68E-01 | 3.41E-01 |
| SETD6 | 79918 | 0.3360767 | 3.7444399 | 2.52E-01 | 4.46E-01 |
| RIOK2 | 55781 | 0.3360545 | 4.7574471 | 7.91E-02 | 2.03E-01 |
| RAB3IP | 117177 | 0.3357538 | 5.2485809 | 1.37E-01 | 2.97E-01 |
| TMEM41B | 440026 | 0.3355781 | 4.7740676 | 1.72E-01 | 3.46E-01 |
| VASH2 | 79805 | 0.3354899 | 4.3133733 | 4.37E-01 | 6.32E-01 |
| ITSN2 | 50618 | 0.3354316 | 4.4968462 | 2.68E-01 | 4.64E-01 |
| KDM4A | 9682 | 0.335349 | 2.9268613 | 2.63E-01 | 4.58E-01 |
| PCDHA11 | 56138 | 0.3353136 | 3.0218176 | 2.80E-01 | 4.77E-01 |
| THAP8 | 199745 | 0.3352939 | 5.6394731 | 1.11E-01 | 2.58E-01 |
| NXT2 | 55916 | 0.3352649 | 2.8644558 | 3.35E-01 | 5.36E-01 |
| OAZ2 | 4947 | 0.3352509 | 9.0860177 | 5.82E-02 | 1.64E-01 |
| LAMA4 | 3910 | 0.3351803 | 2.3118813 | 4.51E-01 | 6.44E-01 |
| DERL2 | 51009 | 0.3350246 | 5.7792856 | 1.72E-01 | 3.46E-01 |
| CASP9 | 842 | 0.3350189 | 2.8932427 | 3.72E-01 | 5.71E-01 |
| GBF1 | 8729 | 0.3348667 | 5.3195903 | 1.49E-01 | 3.14E-01 |
| INTS5 | 80789 | 0.334733 | 3.8189934 | 2.43E-01 | 4.36E-01 |
| SEMA6A | 57556 | 0.3347258 | 4.6937735 | 1.48E-01 | 3.12E-01 |
| ZNF614 | 80110 | 0.3345887 | 3.6182065 | 1.80E-01 | 3.58E-01 |
| KLHL25 | 64410 | 0.3344585 | 3.9363853 | 1.90E-01 | 3.71E-01 |
| NSUN3 | 63899 | 0.3343701 | 4.0117494 | 2.20E-01 | 4.08E-01 |
| EIF4G1 | 1981 | 0.3343208 | 5.7044054 | 3.44E-01 | 5.45E-01 |
| KIF1C | 10749 | 0.3343046 | 3.1753424 | 5.28E-01 | 7.07E-01 |
| RPAP2 | 79871 | 0.334191 | 5.7102842 | 8.53E-02 | 2.14E-01 |
| YOD1 | 55432 | 0.3341788 | 2.9760464 | 1.73E-01 | 3.47E-01 |
| SHMT2 | 6472 | 0.3341563 | 5.6576557 | 3.48E-01 | 5.48E-01 |
| ZNF454 | 285676 | 0.3339278 | 3.3802075 | 2.97E-01 | 4.96E-01 |
| WDR20 | 91833 | 0.3339203 | 5.0563548 | 1.20E-01 | 2.71E-01 |
| ZNF2 | 7549 | 0.3337911 | 3.1129224 | 1.99E-01 | 3.83E-01 |
| CCDC43 | 124808 | 0.3337253 | 5.0037019 | 7.91E-02 | 2.03E-01 |
| MARK2 | 2011 | 0.333719 | 4.9382579 | 1.57E-01 | 3.25E-01 |
| METAP1 | 23173 | 0.3337141 | 5.3957805 | 8.11E-02 | 2.06E-01 |
| TTC19 | 54902 | 0.333435 | 6.7791876 | 6.20E-02 | 1.71E-01 |
| BRD1 | 23774 | 0.3333359 | 4.0211892 | 1.50E-01 | 3.15E-01 |
| ELP3 | 55140 | 0.333289 | 6.0874747 | 5.96E-02 | 1.66E-01 |
| NRG1 | 3084 | 0.3332148 | 4.2185851 | 2.72E-01 | 4.68E-01 |
| RBBP7 | 5931 | 0.3331859 | 8.081861 | 1.39E-01 | 3.00E-01 |
| SLC25A22 | 79751 | 0.3325386 | 4.7522338 | 3.53E-01 | 5.53E-01 |
| PAXIP1 | 22976 | 0.3325194 | 1.5752177 | 4.59E-01 | 6.50E-01 |
| SRD5A1 | 6715 | 0.3322456 | 5.6775206 | 1.68E-01 | 3.42E-01 |
| BMT2 | 154743 | 0.3320174 | 4.3658392 | 2.76E-01 | 4.73E-01 |
| EARS2 | 124454 | 0.3319537 | 3.885278 | 2.60E-01 | 4.55E-01 |
| STX16 | 8675 | 0.3317187 | 4.1864143 | 1.42E-01 | 3.04E-01 |
| SETMAR | 6419 | 0.3315277 | 4.6960088 | 2.64E-01 | 4.59E-01 |
| ACAD8 | 27034 | 0.3314932 | 5.1566099 | 2.52E-01 | 4.46E-01 |
| KIF26B | 55083 | 0.3314176 | 3.691399 | 4.43E-01 | 6.37E-01 |
| HS2ST1 | 9653 | 0.3313685 | 4.0454855 | 2.55E-01 | 4.50E-01 |
| BTF3L4 | 91408 | 0.3312553 | 7.946213 | 1.89E-01 | 3.70E-01 |
| LSM6 | 11157 | 0.3309298 | 5.2502891 | 2.02E-01 | 3.87E-01 |
| B4GALT4 | 8702 | 0.3308681 | 5.0759101 | 2.75E-01 | 4.71E-01 |
| WWC2 | 80014 | 0.3307063 | 2.9415728 | 4.52E-01 | 6.45E-01 |
| ANKS1B | 56899 | 0.3306751 | 6.0024153 | 3.58E-01 | 5.57E-01 |
| TMTC2 | 160335 | 0.3304136 | 1.2814134 | 4.43E-01 | 6.37E-01 |
| SCD5 | 79966 | 0.3303583 | 6.7074142 | 1.80E-01 | 3.58E-01 |
| ASAP1 | 50807 | 0.3303136 | 4.8671984 | 3.76E-01 | 5.76E-01 |
| SNX1 | 6642 | 0.3303125 | 5.5035662 | 2.95E-01 | 4.93E-01 |
| MEAK7 | 57707 | 0.3301501 | 4.7124641 | 1.29E-01 | 2.86E-01 |
| DCTN1 | 1639 | 0.3300907 | 6.4793004 | 1.64E-01 | 3.36E-01 |
| ASAH2 | 56624 | 0.3300268 | 1.5902752 | 3.44E-01 | 5.45E-01 |
| SLX4 | 84464 | 0.3297704 | 1.5882877 | 4.03E-01 | 6.03E-01 |
| FAR1 | 84188 | 0.3297129 | 5.0301544 | 2.28E-01 | 4.18E-01 |
| KLHL22 | 84861 | 0.3289878 | 4.3188866 | 1.21E-01 | 2.73E-01 |
| AMDHD2 | 51005 | 0.3289373 | 2.621822 | 2.09E-01 | 3.95E-01 |
| KIAA1143 | 57456 | 0.3283094 | 5.0367414 | 1.19E-01 | 2.71E-01 |
| MLLT1 | 4298 | 0.3282194 | 3.7388688 | 2.60E-01 | 4.55E-01 |
| ZPR1 | 8882 | 0.3278713 | 5.3944863 | 2.02E-01 | 3.87E-01 |
| ABHD6 | 57406 | 0.3277047 | 5.8299903 | 1.14E-01 | 2.63E-01 |
| GPATCH1 | 55094 | 0.3272305 | 4.1218095 | 1.27E-01 | 2.82E-01 |
| ADPRHL1 | 113622 | 0.3270759 | 0.6289345 | 4.82E-01 | 6.70E-01 |
| MFAP1 | 4236 | 0.3269787 | 6.3950779 | 9.11E-02 | 2.23E-01 |
| WDHD1 | 11169 | 0.3269021 | 2.3894042 | 3.99E-01 | 5.99E-01 |
| CAMKMT | 79823 | 0.3268792 | 4.3467488 | 2.92E-01 | 4.90E-01 |
| ZNF599 | 148103 | 0.3266891 | 3.834403 | 3.06E-01 | 5.06E-01 |
| MTIF3 | 219402 | 0.3266647 | 6.2323789 | 2.07E-01 | 3.93E-01 |
| SSRP1 | 6749 | 0.3263549 | 5.6567732 | 9.14E-02 | 2.24E-01 |
| GON7 | 84520 | 0.3262784 | 4.6746922 | 1.26E-01 | 2.81E-01 |
| STXBP4 | 252983 | 0.3262749 | 2.731123 | 5.16E-01 | 6.97E-01 |
| TPR | 7175 | 0.326243 | 6.5676154 | 7.74E-02 | 2.00E-01 |
| TBC1D2B | 23102 | 0.326233 | 2.3639538 | 3.82E-01 | 5.82E-01 |
| KCNIP1 | 30820 | 0.3262245 | 5.8478063 | 1.66E-01 | 3.37E-01 |
| TIA1 | 7072 | 0.3258631 | 5.2343298 | 1.24E-01 | 2.77E-01 |
| UMPS | 7372 | 0.3257564 | 4.9588732 | 2.86E-01 | 4.84E-01 |
| IPO11 | 51194 | 0.32574 | 3.2591787 | 3.20E-01 | 5.20E-01 |
| DDX6 | 1656 | 0.3257307 | 5.4881843 | 1.09E-01 | 2.55E-01 |
| ZNF235 | 9310 | 0.3257129 | 3.0047077 | 4.22E-01 | 6.20E-01 |
| TMED5 | 50999 | 0.325434 | 4.7180783 | 3.34E-01 | 5.34E-01 |
| TUBGCP5 | 114791 | 0.3252294 | 3.8807388 | 1.35E-01 | 2.95E-01 |
| ZNF688 | 146542 | 0.3251612 | 4.3800322 | 1.12E-01 | 2.59E-01 |
| IMMT | 10989 | 0.3250342 | 7.0012764 | 6.72E-02 | 1.81E-01 |
| ICE2 | 79664 | 0.3249509 | 4.0752958 | 2.49E-01 | 4.43E-01 |
| POC5 | 134359 | 0.3247459 | 4.437076 | 2.09E-01 | 3.95E-01 |
| ROBO3 | 64221 | 0.3246363 | 3.0207087 | 3.57E-01 | 5.56E-01 |
| CCNG2 | 901 | 0.3245194 | 5.3642307 | 2.20E-01 | 4.08E-01 |
| USP22 | 23326 | 0.3245029 | 6.5515723 | 2.99E-01 | 4.98E-01 |
| CYB5RL | 606495 | 0.3244015 | 1.3417157 | 3.87E-01 | 5.87E-01 |
| ZNF671 | 79891 | 0.3240246 | 4.5263566 | 1.26E-01 | 2.80E-01 |
| BAALC | 79870 | 0.3237554 | 7.2238479 | 1.04E-01 | 2.47E-01 |
| RETREG1 | 54463 | 0.3237315 | 6.3349327 | 7.25E-02 | 1.91E-01 |
| FAM200B | 285550 | 0.323508 | 5.4638373 | 9.12E-02 | 2.23E-01 |
| IFT52 | 51098 | 0.3234802 | 6.3486567 | 1.52E-01 | 3.17E-01 |
| ITGB3BP | 23421 | 0.3232934 | 3.2258483 | 3.45E-01 | 5.46E-01 |
| ZNF548 | 147694 | 0.3231652 | 4.4678808 | 2.10E-01 | 3.97E-01 |
| VPS51 | 738 | 0.3227378 | 4.681835 | 2.04E-01 | 3.88E-01 |
| IK | 3550 | 0.3227072 | 7.2894879 | 1.04E-01 | 2.46E-01 |
| P2RX4 | 5025 | 0.3223919 | 4.0919083 | 4.15E-01 | 6.15E-01 |
| DHX8 | 1659 | 0.3218879 | 4.3417431 | 1.27E-01 | 2.83E-01 |
| CCDC126 | 90693 | 0.321619 | 4.8293357 | 1.49E-01 | 3.14E-01 |
| CCDC163 | 126661 | 0.3214444 | 2.0127607 | 5.35E-01 | 7.12E-01 |
| ITPKC | 80271 | 0.3213588 | 3.1460652 | 1.72E-01 | 3.46E-01 |
| ZNF547 | 284306 | 0.3211928 | 2.8955261 | 2.64E-01 | 4.60E-01 |
| HEBP2 | 23593 | 0.3211654 | 5.4988288 | 8.36E-02 | 2.11E-01 |
| HDGF | 3068 | 0.3209528 | 5.4701753 | 2.18E-01 | 4.07E-01 |
| PRKD2 | 25865 | 0.3209193 | 1.6887315 | 4.74E-01 | 6.63E-01 |
| UBR7 | 55148 | 0.3208902 | 4.5850017 | 9.78E-02 | 2.35E-01 |
| CMPK1 | 51727 | 0.3208659 | 7.2068345 | 8.35E-02 | 2.10E-01 |
| MFSD9 | 84804 | 0.3207641 | 3.8070942 | 1.82E-01 | 3.60E-01 |
| HSPA12A | 259217 | 0.3203646 | 5.3466067 | 2.28E-01 | 4.19E-01 |
| NCALD | 83988 | 0.3198726 | 7.4732291 | 1.05E-01 | 2.48E-01 |
| ZNF706 | 51123 | 0.3191529 | 7.5446994 | 1.58E-01 | 3.26E-01 |
| EEF1AKMT1 | 221143 | 0.3189178 | 4.7334855 | 1.72E-01 | 3.46E-01 |
| DHTKD1 | 55526 | 0.3185878 | 4.5744674 | 2.02E-01 | 3.87E-01 |
| PRPF38B | 55119 | 0.3185596 | 5.2601384 | 1.00E-01 | 2.39E-01 |
| SOD2 | 6648 | 0.3184617 | 6.5437924 | 4.93E-01 | 6.79E-01 |
| WDR53 | 348793 | 0.3182366 | 3.6201909 | 1.72E-01 | 3.46E-01 |
| RECQL4 | 9401 | 0.3181514 | 0.4707186 | 4.73E-01 | 6.63E-01 |
| MCUR1 | 63933 | 0.3181101 | 5.9951559 | 1.15E-01 | 2.63E-01 |
| TMEM9B | 56674 | 0.3179718 | 7.2677566 | 9.12E-02 | 2.23E-01 |
| MRPL3 | 11222 | 0.3177949 | 7.2205872 | 8.29E-02 | 2.09E-01 |
| STXBP1 | 6812 | 0.3176581 | 8.5102445 | 4.37E-01 | 6.32E-01 |
| RPS23 | 6228 | 0.3175587 | 6.2433647 | 1.48E-01 | 3.12E-01 |
| NUDT8 | 254552 | 0.3173383 | 2.4496539 | 2.95E-01 | 4.93E-01 |
| ZNF789 | 285989 | 0.3173103 | 3.054702 | 3.92E-01 | 5.92E-01 |
| SYNE2 | 23224 | 0.317193 | 4.0830908 | 1.21E-01 | 2.74E-01 |
| ST7 | 7982 | 0.3171408 | 6.2637287 | 1.04E-01 | 2.46E-01 |
| CDC5L | 988 | 0.3169691 | 6.6973112 | 2.05E-01 | 3.90E-01 |
| STK38 | 11329 | 0.3169629 | 3.8722537 | 3.80E-01 | 5.80E-01 |
| DHX15 | 1665 | 0.3169331 | 6.8790791 | 1.77E-01 | 3.53E-01 |
| PTPRN | 5798 | 0.3169111 | 8.1080454 | 1.92E-01 | 3.74E-01 |
| PRR19 | 284338 | 0.3168805 | 3.1065589 | 2.32E-01 | 4.24E-01 |
| EIF4H | 7458 | 0.3168555 | 7.8065903 | 1.55E-01 | 3.21E-01 |
| POLR1D | 51082 | 0.3166242 | 6.4727178 | 1.17E-01 | 2.67E-01 |
| ENOPH1 | 58478 | 0.3165978 | 7.6999902 | 2.13E-01 | 4.00E-01 |
| CLU | 1191 | 0.3164873 | 10.094606 | 2.78E-01 | 4.75E-01 |
| UBE2V2 | 7336 | 0.3164498 | 8.7035187 | 2.37E-01 | 4.28E-01 |
| PPP6C | 5537 | 0.3161371 | 5.6096507 | 1.67E-01 | 3.40E-01 |
| WASHC1 | 100287171 | 0.3161002 | 0.6103853 | 4.50E-01 | 6.43E-01 |
| STK32C | 282974 | 0.3158889 | 5.3716475 | 2.88E-01 | 4.86E-01 |
| XPO7 | 23039 | 0.3158097 | 5.791491 | 8.45E-02 | 2.12E-01 |
| AGO4 | 192670 | 0.3155709 | 3.9443065 | 3.95E-01 | 5.95E-01 |
| GHITM | 27069 | 0.3155126 | 9.4474061 | 8.18E-02 | 2.08E-01 |
| IKZF5 | 64376 | 0.3153796 | 3.7367403 | 1.89E-01 | 3.70E-01 |
| AKAP1 | 8165 | 0.3153027 | 5.2890236 | 8.66E-02 | 2.16E-01 |
| SCAMP4 | 113178 | 0.3152699 | 4.9285756 | 2.51E-01 | 4.44E-01 |
| KLHL18 | 23276 | 0.3152155 | 5.2288891 | 7.27E-02 | 1.91E-01 |
| RPL7 | 6129 | 0.3151783 | 8.6453231 | 1.29E-01 | 2.85E-01 |
| RNF220 | 55182 | 0.3151516 | 5.5855592 | 1.57E-01 | 3.24E-01 |
| AAMDC | 28971 | 0.3149742 | 2.4207033 | 4.87E-01 | 6.74E-01 |
| MAPRE1 | 22919 | 0.3148788 | 7.2041998 | 9.41E-02 | 2.29E-01 |
| GLYR1 | 84656 | 0.3148779 | 4.6876739 | 1.09E-01 | 2.54E-01 |
| TAOK2 | 9344 | 0.3146372 | 4.0092842 | 2.54E-01 | 4.48E-01 |
| MIB1 | 57534 | 0.3144748 | 4.4192449 | 1.34E-01 | 2.93E-01 |
| B3GALT4 | 8705 | 0.3144133 | 3.3501614 | 2.33E-01 | 4.24E-01 |
| PAPSS1 | 9061 | 0.3143556 | 7.4106112 | 9.31E-02 | 2.27E-01 |
| MYRIP | 25924 | 0.3140131 | 1.710095 | 3.62E-01 | 5.62E-01 |
| NFE2L3 | 9603 | 0.3138129 | 0.9741739 | 4.52E-01 | 6.45E-01 |
| ZNF341 | 84905 | 0.3137848 | 1.294285 | 4.47E-01 | 6.41E-01 |
| RGL2 | 5863 | 0.313758 | 4.0008379 | 3.60E-01 | 5.59E-01 |
| SH3BP5 | 9467 | 0.3137361 | 4.8814428 | 3.66E-01 | 5.65E-01 |
| MAP6 | 4135 | 0.3136824 | 7.8130355 | 9.07E-02 | 2.23E-01 |
| ZMYM4 | 9202 | 0.3135978 | 6.483047 | 1.05E-01 | 2.48E-01 |
| AMOTL2 | 51421 | 0.3135104 | 5.1570706 | 4.26E-01 | 6.24E-01 |
| PITPNA | 5306 | 0.3134714 | 7.284944 | 1.26E-01 | 2.81E-01 |
| LYRM4 | 57128 | 0.313288 | 5.3466285 | 9.94E-02 | 2.38E-01 |
| PA2G4 | 5036 | 0.3131038 | 6.1295393 | 8.73E-02 | 2.17E-01 |
| NR2C2 | 7182 | 0.3130976 | 1.3644299 | 4.63E-01 | 6.54E-01 |
| ASAH1 | 427 | 0.3129403 | 8.728657 | 3.37E-01 | 5.37E-01 |
| ELOA | 6924 | 0.3128357 | 6.3686719 | 9.41E-02 | 2.28E-01 |
| HLA-DRB1 | 3123 | 0.3127342 | 5.2602959 | 5.05E-01 | 6.90E-01 |
| EFR3A | 23167 | 0.3125895 | 3.887922 | 4.12E-01 | 6.12E-01 |
| NCBP1 | 4686 | 0.3123766 | 5.4967835 | 1.64E-01 | 3.36E-01 |
| SSBP3 | 23648 | 0.3118126 | 4.7860407 | 1.76E-01 | 3.51E-01 |
| SNRPD1 | 6632 | 0.3116113 | 7.8711315 | 3.59E-01 | 5.58E-01 |
| RANBP2 | 5903 | 0.3114463 | 4.8416911 | 1.68E-01 | 3.41E-01 |
| STMP1 | 647087 | 0.3113848 | 8.1376238 | 1.00E-01 | 2.39E-01 |
| AFG1L | 246269 | 0.3111248 | 2.2033077 | 4.68E-01 | 6.58E-01 |
| ITM2C | 81618 | 0.3109897 | 7.5055957 | 1.73E-01 | 3.47E-01 |
| VPS26C | 10311 | 0.3108327 | 5.4614779 | 1.50E-01 | 3.14E-01 |
| NEU3 | 10825 | 0.3107931 | 2.1063841 | 3.57E-01 | 5.57E-01 |
| DDX23 | 9416 | 0.3107425 | 5.3765831 | 1.70E-01 | 3.43E-01 |
| THAP2 | 83591 | 0.3107408 | 4.2370661 | 1.89E-01 | 3.70E-01 |
| ST13 | 6767 | 0.3105778 | 8.1645047 | 1.46E-01 | 3.10E-01 |
| TCEAL7 | 56849 | 0.3105263 | 8.4396982 | 4.28E-01 | 6.26E-01 |
| ZSCAN32 | 54925 | 0.3104722 | 3.8721571 | 2.29E-01 | 4.20E-01 |
| WBP2 | 23558 | 0.3103919 | 6.9776133 | 2.32E-01 | 4.24E-01 |
| ACOT13 | 55856 | 0.3103485 | 5.4353585 | 1.24E-01 | 2.79E-01 |
| TOP2B | 7155 | 0.3103177 | 7.0591199 | 1.41E-01 | 3.02E-01 |
| ZNF773 | 374928 | 0.3100663 | 3.4657975 | 1.91E-01 | 3.72E-01 |
| RBM14 | 10432 | 0.3099506 | 5.00824 | 2.80E-01 | 4.78E-01 |
| NUDT4 | 11163 | 0.30994 | 4.3173494 | 1.53E-01 | 3.19E-01 |
| LONP2 | 83752 | 0.3098411 | 5.5103887 | 1.09E-01 | 2.55E-01 |
| LRRC20 | 55222 | 0.309782 | 3.6828913 | 3.09E-01 | 5.08E-01 |
| SNRPE | 6635 | 0.3097564 | 7.7887558 | 3.09E-01 | 5.08E-01 |
| UBA3 | 9039 | 0.3094892 | 6.812193 | 1.07E-01 | 2.50E-01 |
| PUS3 | 83480 | 0.3092159 | 4.6981211 | 9.33E-02 | 2.27E-01 |
| HNRNPC | 3183 | 0.3087997 | 9.4296966 | 1.04E-01 | 2.47E-01 |
| LEMD2 | 221496 | 0.3086865 | 5.0371773 | 4.54E-01 | 6.46E-01 |
| NUDCD3 | 23386 | 0.3084146 | 6.3215769 | 1.21E-01 | 2.73E-01 |
| XRCC4 | 7518 | 0.3083385 | 4.1009325 | 2.09E-01 | 3.96E-01 |
| BCAS3 | 54828 | 0.3082351 | 3.5530638 | 2.34E-01 | 4.26E-01 |
| MAPK9 | 5601 | 0.3080512 | 5.8226999 | 1.04E-01 | 2.47E-01 |
| MLLT11 | 10962 | 0.3079682 | 11.690548 | 5.39E-01 | 7.15E-01 |
| SFXN1 | 94081 | 0.3076593 | 6.6538654 | 1.38E-01 | 2.99E-01 |
| TAF1B | 9014 | 0.3074593 | 4.5913311 | 2.12E-01 | 3.99E-01 |
| C12orf4 | 57102 | 0.3073455 | 4.4573995 | 1.42E-01 | 3.04E-01 |
| GPATCH8 | 23131 | 0.3072882 | 5.8518396 | 1.18E-01 | 2.69E-01 |
| CAT | 847 | 0.3071974 | 5.0627733 | 3.72E-01 | 5.71E-01 |
| PTP4A2 | 8073 | 0.3071863 | 8.4303534 | 8.29E-02 | 2.09E-01 |
| SETD5 | 55209 | 0.3067884 | 5.5516443 | 9.12E-02 | 2.23E-01 |
| SERINC3 | 10955 | 0.3064553 | 8.0817548 | 1.33E-01 | 2.91E-01 |
| ZNF518A | 9849 | 0.3063933 | 4.4848171 | 2.00E-01 | 3.84E-01 |
| CGGBP1 | 8545 | 0.3063503 | 6.3580963 | 8.25E-02 | 2.09E-01 |
| ABHD17B | 51104 | 0.3063217 | 4.8122303 | 1.26E-01 | 2.81E-01 |
| YKT6 | 10652 | 0.3063034 | 5.7116764 | 2.42E-01 | 4.34E-01 |
| BCKDHB | 594 | 0.3062894 | 5.6371856 | 1.65E-01 | 3.37E-01 |
| USP7 | 7874 | 0.306245 | 5.8643327 | 1.16E-01 | 2.65E-01 |
| NAA35 | 60560 | 0.3061316 | 5.7262139 | 1.96E-01 | 3.79E-01 |
| GALNT7 | 51809 | 0.3058503 | 3.1811195 | 5.19E-01 | 7.00E-01 |
| ADPRM | 56985 | 0.3055968 | 3.582053 | 2.35E-01 | 4.27E-01 |
| SMIM14 | 201895 | 0.3055659 | 6.5910306 | 9.99E-02 | 2.39E-01 |
| ESD | 2098 | 0.3052085 | 7.5004423 | 1.56E-01 | 3.24E-01 |
| NLN | 57486 | 0.305183 | 5.7720975 | 2.44E-01 | 4.37E-01 |
| MCCC2 | 64087 | 0.3051285 | 4.6528652 | 4.31E-01 | 6.28E-01 |
| RMND1 | 55005 | 0.3049432 | 5.5718885 | 8.99E-02 | 2.21E-01 |
| LMBRD1 | 55788 | 0.3049324 | 6.5724777 | 8.53E-02 | 2.14E-01 |
| VIRMA | 25962 | 0.3048593 | 3.4607575 | 2.68E-01 | 4.64E-01 |
| BAG2 | 9532 | 0.3045841 | 3.9762717 | 3.50E-01 | 5.51E-01 |
| GTPBP1 | 9567 | 0.3041412 | 5.2077644 | 2.75E-01 | 4.72E-01 |
| DPY19L4 | 286148 | 0.3039086 | 2.8252235 | 4.14E-01 | 6.13E-01 |
| CDR2 | 1039 | 0.3038603 | 3.9672766 | 3.81E-01 | 5.81E-01 |
| LRRC49 | 54839 | 0.3032079 | 5.7857187 | 1.59E-01 | 3.28E-01 |
| C1orf174 | 339448 | 0.3030799 | 5.3346849 | 8.24E-02 | 2.09E-01 |
| SNIP1 | 79753 | 0.3028189 | 3.4786313 | 2.20E-01 | 4.09E-01 |
| AP5M1 | 55745 | 0.3027971 | 6.1766386 | 8.93E-02 | 2.20E-01 |
| SLC9A3R2 | 9351 | 0.302496 | 2.8013584 | 3.47E-01 | 5.47E-01 |
| USP19 | 10869 | 0.3023252 | 4.2375484 | 1.88E-01 | 3.67E-01 |
| TXNDC9 | 10190 | 0.3021223 | 6.4861887 | 1.30E-01 | 2.88E-01 |
| CDKAL1 | 54901 | 0.3020993 | 3.5038828 | 2.55E-01 | 4.50E-01 |
| DBN1 | 1627 | 0.3020161 | 7.9594936 | 1.17E-01 | 2.67E-01 |
| TMEM242 | 729515 | 0.3020053 | 5.8549437 | 1.43E-01 | 3.05E-01 |
| LTV1 | 84946 | 0.3015683 | 4.2059345 | 2.15E-01 | 4.03E-01 |
| VPS54 | 51542 | 0.3013533 | 3.5177252 | 3.49E-01 | 5.50E-01 |
| CLINT1 | 9685 | 0.3011305 | 5.9255327 | 2.04E-01 | 3.89E-01 |
| FRMD3 | 257019 | 0.3011103 | 4.8205665 | 2.53E-01 | 4.47E-01 |
| USP39 | 10713 | 0.3010298 | 6.0684115 | 2.43E-01 | 4.35E-01 |
| RAB5B | 5869 | 0.3009867 | 5.1676472 | 2.94E-01 | 4.92E-01 |
| MBTPS1 | 8720 | 0.3009721 | 5.1435001 | 2.75E-01 | 4.72E-01 |
| ZSCAN22 | 342945 | 0.3009369 | 2.0487356 | 3.05E-01 | 5.04E-01 |
| TASOR2 | 54906 | 0.300922 | 3.7187279 | 3.07E-01 | 5.06E-01 |
| NAIF1 | 203245 | 0.3009198 | 3.1961551 | 3.24E-01 | 5.25E-01 |
| RALGDS | 5900 | 0.3007862 | 3.2056714 | 2.64E-01 | 4.60E-01 |
| HSBP1 | 3281 | 0.3006777 | 9.5124521 | 2.77E-01 | 4.74E-01 |
| ADK | 132 | 0.3005045 | 6.0794959 | 1.23E-01 | 2.76E-01 |
| PER2 | 8864 | 0.3004558 | 2.3769626 | 3.20E-01 | 5.21E-01 |
| TSR1 | 55720 | 0.3004357 | 4.7025286 | 3.50E-01 | 5.51E-01 |
| COMMD7 | 149951 | 0.3004221 | 6.7770166 | 2.38E-01 | 4.30E-01 |
| POLE3 | 54107 | 0.3003423 | 6.297362 | 9.54E-02 | 2.31E-01 |
| WRNIP1 | 56897 | 0.3001997 | 6.1055826 | 1.11E-01 | 2.58E-01 |
| LEPROTL1 | 23484 | 0.3001923 | 6.9052422 | 8.38E-02 | 2.11E-01 |
| RBM28 | 55131 | 0.3001137 | 4.3179416 | 1.68E-01 | 3.41E-01 |
| NKAP | 79576 | 0.2997168 | 3.6091205 | 2.02E-01 | 3.86E-01 |
| PHTF1 | 10745 | 0.2994168 | 5.0914198 | 3.38E-01 | 5.39E-01 |
| MAP1LC3B2 | 643246 | 0.2992626 | 4.2670807 | 1.37E-01 | 2.96E-01 |
| ANKRD11 | 29123 | 0.2991802 | 7.155778 | 9.63E-02 | 2.32E-01 |
| TP53RK | 112858 | 0.2990468 | 5.8786323 | 1.60E-01 | 3.29E-01 |
| HSPA1B | 3304 | 0.2986254 | 4.4016573 | 3.01E-01 | 5.00E-01 |
| MRS2 | 57380 | 0.2983517 | 5.241926 | 3.01E-01 | 5.00E-01 |
| MAPK7 | 5598 | 0.2983393 | 3.4438753 | 2.39E-01 | 4.30E-01 |
| CEP97 | 79598 | 0.2982413 | 4.4679037 | 1.30E-01 | 2.88E-01 |
| ACSS2 | 55902 | 0.2981553 | 4.747992 | 4.20E-01 | 6.19E-01 |
| KDELR2 | 11014 | 0.2980701 | 7.8120039 | 3.54E-01 | 5.54E-01 |
| CEP57 | 9702 | 0.2980364 | 6.3577668 | 4.35E-01 | 6.30E-01 |
| CAPN7 | 23473 | 0.2979396 | 4.8859488 | 2.00E-01 | 3.84E-01 |
| PRPF4 | 9128 | 0.2978792 | 5.2686007 | 1.69E-01 | 3.43E-01 |
| BCAS2 | 10286 | 0.2977375 | 7.8861924 | 2.00E-01 | 3.84E-01 |
| SNX6 | 58533 | 0.2976913 | 6.7074346 | 8.57E-02 | 2.14E-01 |
| JAM2 | 58494 | 0.2972698 | 3.8812804 | 4.30E-01 | 6.27E-01 |
| CUL1 | 8454 | 0.297167 | 6.2633608 | 8.23E-02 | 2.09E-01 |
| MTMR9 | 66036 | 0.2967568 | 5.9178635 | 3.83E-01 | 5.83E-01 |
| DLG3 | 1741 | 0.2965146 | 4.3475824 | 1.56E-01 | 3.23E-01 |
| KANK1 | 23189 | 0.296017 | 2.7629028 | 5.17E-01 | 6.98E-01 |
| RBSN | 64145 | 0.2958878 | 5.4082643 | 1.49E-01 | 3.14E-01 |
| SNAPC3 | 6619 | 0.2958625 | 6.0751939 | 1.64E-01 | 3.35E-01 |
| SNF8 | 11267 | 0.2957869 | 5.5903259 | 8.04E-02 | 2.05E-01 |
| ZUP1 | 221302 | 0.2956439 | 5.2562073 | 2.63E-01 | 4.58E-01 |
| CAB39L | 81617 | 0.2955719 | 5.3360363 | 1.87E-01 | 3.67E-01 |
| LYSMD3 | 116068 | 0.2955434 | 4.1776785 | 3.48E-01 | 5.48E-01 |
| BPHL | 670 | 0.2951424 | 5.6522823 | 1.96E-01 | 3.79E-01 |
| PIM2 | 11040 | 0.295004 | 4.1179965 | 3.54E-01 | 5.53E-01 |
| TMEM260 | 54916 | 0.2949365 | 3.4209785 | 2.25E-01 | 4.15E-01 |
| PPT2 | 9374 | 0.2948919 | 4.013778 | 1.43E-01 | 3.05E-01 |
| REEP2 | 51308 | 0.2948702 | 6.081173 | 2.41E-01 | 4.33E-01 |
| PINLYP | 390940 | 0.2948251 | 2.3394101 | 4.08E-01 | 6.08E-01 |
| CSNK1G1 | 53944 | 0.2943881 | 3.6354075 | 2.59E-01 | 4.55E-01 |
| NARS1 | 4677 | 0.2940359 | 7.6049426 | 2.04E-01 | 3.88E-01 |
| RAD1 | 5810 | 0.2939051 | 6.1544345 | 2.18E-01 | 4.06E-01 |
| KMT2E | 55904 | 0.2937684 | 7.9141313 | 2.50E-01 | 4.43E-01 |
| MON1A | 84315 | 0.2937654 | 4.9925969 | 2.83E-01 | 4.81E-01 |
| GNPAT | 8443 | 0.2935858 | 6.1781701 | 1.05E-01 | 2.48E-01 |
| NUAK1 | 9891 | 0.2935035 | 4.0193675 | 2.20E-01 | 4.08E-01 |
| COPZ1 | 22818 | 0.2932895 | 7.6996389 | 1.03E-01 | 2.45E-01 |
| CACNB3 | 784 | 0.2931205 | 5.5888165 | 4.57E-01 | 6.49E-01 |
| SLC1A2 | 6506 | 0.2930627 | 5.4945417 | 4.36E-01 | 6.31E-01 |
| PITRM1 | 10531 | 0.2928276 | 5.3827619 | 2.17E-01 | 4.05E-01 |
| C2orf49 | 79074 | 0.2927411 | 4.725747 | 1.09E-01 | 2.54E-01 |
| ZNF34 | 80778 | 0.2926721 | 3.7556771 | 2.83E-01 | 4.81E-01 |
| SLC25A24 | 29957 | 0.2925591 | 3.8781118 | 1.83E-01 | 3.62E-01 |
| SETX | 23064 | 0.2923633 | 5.2365201 | 2.99E-01 | 4.98E-01 |
| GADD45A | 1647 | 0.2923284 | 5.9351719 | 4.93E-01 | 6.80E-01 |
| HYAL3 | 8372 | 0.2919028 | 3.7557182 | 3.11E-01 | 5.10E-01 |
| CDR2L | 30850 | 0.2918168 | 3.7457146 | 2.49E-01 | 4.43E-01 |
| SNAP91 | 9892 | 0.2915676 | 6.7693498 | 5.35E-01 | 7.12E-01 |
| RCCD1 | 91433 | 0.2914972 | 3.4234601 | 4.51E-01 | 6.44E-01 |
| GALNT17 | 64409 | 0.2913149 | 4.8996298 | 4.35E-01 | 6.30E-01 |
| MRPS27 | 23107 | 0.2908215 | 5.9289343 | 1.79E-01 | 3.55E-01 |
| ZNF397 | 84307 | 0.2907844 | 5.0636971 | 1.91E-01 | 3.72E-01 |
| PIK3CB | 5291 | 0.290742 | 3.8242511 | 2.66E-01 | 4.61E-01 |
| SECISBP2L | 9728 | 0.2903198 | 5.9048593 | 1.90E-01 | 3.71E-01 |
| TCEANC2 | 127428 | 0.2900732 | 3.3951734 | 3.32E-01 | 5.33E-01 |
| M6PR | 4074 | 0.289865 | 6.0123203 | 3.71E-01 | 5.70E-01 |
| MARK1 | 4139 | 0.2897355 | 5.9993446 | 3.89E-01 | 5.90E-01 |
| ZNF225 | 7768 | 0.2892411 | 2.875041 | 2.91E-01 | 4.90E-01 |
| ARSK | 153642 | 0.289211 | 4.3708962 | 1.86E-01 | 3.65E-01 |
| PAIP2 | 51247 | 0.289172 | 7.784441 | 1.57E-01 | 3.25E-01 |
| ZDHHC6 | 64429 | 0.2891589 | 5.7842797 | 1.35E-01 | 2.95E-01 |
| ABCC5 | 10057 | 0.2888846 | 4.5563585 | 4.06E-01 | 6.06E-01 |
| TIFA | 92610 | 0.2888597 | 2.9708311 | 4.34E-01 | 6.29E-01 |
| ZNF677 | 342926 | 0.2888451 | 5.6213119 | 2.50E-01 | 4.43E-01 |
| AREL1 | 9870 | 0.2886707 | 4.7999814 | 2.36E-01 | 4.28E-01 |
| BTAF1 | 9044 | 0.2886327 | 4.4017559 | 2.95E-01 | 4.93E-01 |
| USP24 | 23358 | 0.2879331 | 4.0520207 | 2.57E-01 | 4.52E-01 |
| DNAJB1 | 3337 | 0.2876565 | 6.4236283 | 3.52E-01 | 5.51E-01 |
| UBE2N | 7334 | 0.287547 | 8.7517403 | 2.95E-01 | 4.93E-01 |
| SUGT1 | 10910 | 0.2874337 | 6.8361882 | 1.40E-01 | 3.01E-01 |
| ZKSCAN1 | 7586 | 0.2870757 | 6.4213355 | 1.62E-01 | 3.31E-01 |
| ARL17A | 51326 | 0.2869128 | 1.689297 | 5.06E-01 | 6.90E-01 |
| ZSCAN30 | 100101467 | 0.286887 | 4.0279079 | 2.32E-01 | 4.23E-01 |
| COPB2 | 9276 | 0.2867115 | 6.9406139 | 4.12E-01 | 6.11E-01 |
| AKAP11 | 11215 | 0.2865634 | 5.393775 | 1.99E-01 | 3.83E-01 |
| DLD | 1738 | 0.2862813 | 7.260983 | 1.49E-01 | 3.14E-01 |
| ZDHHC18 | 84243 | 0.2862783 | 4.679066 | 3.33E-01 | 5.34E-01 |
| CNOT8 | 9337 | 0.2861484 | 6.7920235 | 1.57E-01 | 3.25E-01 |
| DOP1A | 23033 | 0.2861184 | 3.339854 | 2.06E-01 | 3.91E-01 |
| OGT | 8473 | 0.2861162 | 5.9691553 | 1.56E-01 | 3.23E-01 |
| MSANTD4 | 84437 | 0.2859884 | 5.8445125 | 1.01E-01 | 2.41E-01 |
| GALC | 2581 | 0.2859516 | 4.4258875 | 2.13E-01 | 4.00E-01 |
| APBA2 | 321 | 0.2855462 | 6.5122893 | 4.16E-01 | 6.16E-01 |
| ATG14 | 22863 | 0.2854467 | 3.0545958 | 2.84E-01 | 4.82E-01 |
| MACF1 | 23499 | 0.285301 | 6.332754 | 2.73E-01 | 4.69E-01 |
| TVP23B | 51030 | 0.2851601 | 6.5002106 | 3.25E-01 | 5.25E-01 |
| CYB5B | 80777 | 0.2851232 | 7.4544207 | 1.10E-01 | 2.55E-01 |
| SERPINB6 | 5269 | 0.285117 | 5.0431454 | 2.77E-01 | 4.74E-01 |
| FAM177A1 | 283635 | 0.2847518 | 7.2504551 | 2.00E-01 | 3.84E-01 |
| TIGD3 | 220359 | 0.2845985 | 2.8225239 | 2.59E-01 | 4.54E-01 |
| LRRC57 | 255252 | 0.2845932 | 3.2693076 | 3.40E-01 | 5.41E-01 |
| IPO9 | 55705 | 0.2845251 | 6.5400553 | 1.27E-01 | 2.83E-01 |
| TCP1 | 6950 | 0.2844482 | 8.4258433 | 1.35E-01 | 2.94E-01 |
| MAPK10 | 5602 | 0.2842762 | 6.9830101 | 4.69E-01 | 6.59E-01 |
| ERAL1 | 26284 | 0.2842141 | 6.3678599 | 1.70E-01 | 3.44E-01 |
| UBE2W | 55284 | 0.2839407 | 5.4586617 | 1.57E-01 | 3.25E-01 |
| RBAK | 57786 | 0.2837016 | 3.0873047 | 2.98E-01 | 4.96E-01 |
| HNRNPUL1 | 11100 | 0.2835284 | 4.5626032 | 1.72E-01 | 3.46E-01 |
| ARHGEF18 | 23370 | 0.2835249 | 3.617494 | 2.41E-01 | 4.33E-01 |
| CPEB2 | 132864 | 0.2833604 | 3.9793911 | 3.29E-01 | 5.29E-01 |
| SLC12A7 | 10723 | 0.2831469 | 1.9210002 | 4.91E-01 | 6.77E-01 |
| ZNF346 | 23567 | 0.2829793 | 5.6476175 | 1.13E-01 | 2.61E-01 |
| MCEE | 84693 | 0.2825321 | 3.5075565 | 3.83E-01 | 5.83E-01 |
| OSER1 | 51526 | 0.2824352 | 6.8218474 | 1.82E-01 | 3.60E-01 |
| PTCD2 | 79810 | 0.2823728 | 3.718007 | 2.43E-01 | 4.35E-01 |
| FEM1B | 10116 | 0.282249 | 5.4599686 | 1.42E-01 | 3.04E-01 |
| EIF2AK2 | 5610 | 0.2817459 | 5.800405 | 2.38E-01 | 4.30E-01 |
| GGH | 8836 | 0.2815488 | 8.5033497 | 1.14E-01 | 2.63E-01 |
| AK5 | 26289 | 0.2814839 | 5.1211974 | 5.16E-01 | 6.97E-01 |
| PRKAA1 | 5562 | 0.2814565 | 4.0304114 | 4.77E-01 | 6.66E-01 |
| FRS2 | 10818 | 0.2814487 | 4.3208995 | 2.35E-01 | 4.27E-01 |
| NAB1 | 4664 | 0.2810818 | 3.3110739 | 2.67E-01 | 4.62E-01 |
| ABHD13 | 84945 | 0.2809731 | 5.3694467 | 1.32E-01 | 2.90E-01 |
| MRPL10 | 124995 | 0.2809505 | 5.9438691 | 1.09E-01 | 2.54E-01 |
| RBM25 | 58517 | 0.2806916 | 7.2246325 | 2.34E-01 | 4.26E-01 |
| ACO1 | 48 | 0.2805347 | 4.9056848 | 5.06E-01 | 6.90E-01 |
| ZNF532 | 55205 | 0.2803244 | 5.3274422 | 1.49E-01 | 3.14E-01 |
| AFTPH | 54812 | 0.2801309 | 4.4937105 | 1.90E-01 | 3.71E-01 |
| CLPX | 10845 | 0.2799233 | 5.1363867 | 2.05E-01 | 3.90E-01 |
| PAIP1 | 10605 | 0.2797889 | 7.1441152 | 1.14E-01 | 2.62E-01 |
| USP4 | 107986084 | 0.2797051 | 5.0780902 | 1.45E-01 | 3.08E-01 |
| ABRACL | 58527 | 0.279647 | 7.4088925 | 4.37E-01 | 6.32E-01 |
| MDH1B | 130752 | 0.279059 | 2.6096094 | 5.20E-01 | 7.01E-01 |
| PAM | 5066 | 0.27904 | 7.9888469 | 3.24E-01 | 5.25E-01 |
| PTPN4 | 5775 | 0.2790125 | 4.0888093 | 2.87E-01 | 4.85E-01 |
| RAB5A | 5868 | 0.2789076 | 6.7989191 | 1.81E-01 | 3.59E-01 |
| MBD4 | 8930 | 0.2788758 | 5.2501176 | 1.76E-01 | 3.52E-01 |
| EIF2S1 | 1965 | 0.2787691 | 6.9681763 | 1.31E-01 | 2.89E-01 |
| GEMIN6 | 79833 | 0.2786374 | 5.0560105 | 1.32E-01 | 2.90E-01 |
| RDH13 | 112724 | 0.2786323 | 3.7877426 | 4.08E-01 | 6.08E-01 |
| YTHDF3 | 253943 | 0.2786169 | 5.3641035 | 1.42E-01 | 3.04E-01 |
| NIPA2 | 81614 | 0.2783702 | 6.29817 | 1.92E-01 | 3.74E-01 |
| CLN5 | 1203 | 0.2783095 | 2.5113914 | 5.28E-01 | 7.07E-01 |
| SHLD1 | 149840 | 0.2782803 | 4.1692057 | 2.45E-01 | 4.38E-01 |
| PNPT1 | 87178 | 0.278272 | 4.8371545 | 2.15E-01 | 4.03E-01 |
| TTLL11 | 158135 | 0.2782668 | 2.891189 | 3.73E-01 | 5.73E-01 |
| ANKRD54 | 129138 | 0.278226 | 5.987439 | 1.31E-01 | 2.88E-01 |
| ZBTB9 | 221504 | 0.2780093 | 3.7771274 | 3.03E-01 | 5.02E-01 |
| KBTBD7 | 84078 | 0.2779167 | 5.3954397 | 2.07E-01 | 3.93E-01 |
| ZNF417 | 147687 | 0.2775842 | 2.003055 | 3.87E-01 | 5.88E-01 |
| ATP2C1 | 27032 | 0.2772425 | 5.2445178 | 1.76E-01 | 3.51E-01 |
| SLAIN1 | 122060 | 0.277119 | 6.0479989 | 1.76E-01 | 3.51E-01 |
| TMEM200C | 645369 | 0.2764306 | 1.8863243 | 5.04E-01 | 6.89E-01 |
| FAM168B | 130074 | 0.2763911 | 5.8629366 | 1.17E-01 | 2.67E-01 |
| RETREG3 | 162427 | 0.2762521 | 5.2502059 | 3.37E-01 | 5.38E-01 |
| JAK1 | 3716 | 0.2760589 | 6.6339629 | 3.83E-01 | 5.83E-01 |
| GTF3C2 | 2976 | 0.2759397 | 4.1956656 | 2.70E-01 | 4.66E-01 |
| LDB1 | 8861 | 0.2757699 | 5.8997397 | 3.69E-01 | 5.68E-01 |
| HAUS3 | 79441 | 0.2757091 | 3.5029243 | 2.93E-01 | 4.91E-01 |
| ARID2 | 196528 | 0.2753692 | 3.8811061 | 2.72E-01 | 4.68E-01 |
| C22orf39 | 128977 | 0.2753039 | 5.1182711 | 1.61E-01 | 3.30E-01 |
| PTCD1 | 26024 | 0.2752836 | 0.7466296 | 5.08E-01 | 6.91E-01 |
| PGS1 | 9489 | 0.2751394 | 4.6834352 | 2.46E-01 | 4.39E-01 |
| TMEM38B | 55151 | 0.2750675 | 4.216409 | 4.90E-01 | 6.77E-01 |
| MCM3 | 4172 | 0.2748766 | 3.6867021 | 3.50E-01 | 5.50E-01 |
| ZNF660 | 285349 | 0.2748597 | 2.4136282 | 3.71E-01 | 5.70E-01 |
| MROH8 | 140699 | 0.2747875 | 2.9523342 | 3.57E-01 | 5.56E-01 |
| GMCL1 | 64395 | 0.2746853 | 4.2755017 | 4.44E-01 | 6.37E-01 |
| SMC4 | 10051 | 0.2745879 | 3.669974 | 3.61E-01 | 5.60E-01 |
| DHX40 | 79665 | 0.2739482 | 4.9217261 | 4.71E-01 | 6.60E-01 |
| CERT1 | 10087 | 0.2739381 | 5.566481 | 4.30E-01 | 6.27E-01 |
| PRDM11 | 56981 | 0.2738063 | 1.7723082 | 5.01E-01 | 6.87E-01 |
| BCL2 | 596 | 0.2736415 | 2.9048815 | 4.61E-01 | 6.52E-01 |
| CERS4 | 79603 | 0.2736233 | 3.3953046 | 3.97E-01 | 5.97E-01 |
| KPNA3 | 3839 | 0.2735634 | 6.7386532 | 1.96E-01 | 3.79E-01 |
| CTDNEP1 | 23399 | 0.2734935 | 3.3139087 | 4.01E-01 | 6.01E-01 |
| ATG7 | 10533 | 0.2730998 | 3.4194935 | 3.75E-01 | 5.75E-01 |
| TUBGCP4 | 27229 | 0.2729646 | 4.1580798 | 2.15E-01 | 4.03E-01 |
| TMEM248 | 55069 | 0.2728999 | 5.8189762 | 3.43E-01 | 5.43E-01 |
| EEF2 | 1938 | 0.2728841 | 5.7876161 | 3.62E-01 | 5.62E-01 |
| ZDHHC9 | 51114 | 0.2728598 | 6.6022887 | 1.30E-01 | 2.87E-01 |
| TEX261 | 113419 | 0.2725369 | 4.4072113 | 4.53E-01 | 6.45E-01 |
| JMJD8 | 339123 | 0.2721378 | 3.7480583 | 4.07E-01 | 6.07E-01 |
| ZNF674 | 641339 | 0.2719327 | 1.9462986 | 4.06E-01 | 6.07E-01 |
| LCMT1 | 51451 | 0.2715016 | 7.1837052 | 2.82E-01 | 4.80E-01 |
| MCM3AP | 8888 | 0.2714455 | 2.857395 | 3.19E-01 | 5.19E-01 |
| DNAJC19 | 131118 | 0.2713189 | 6.8784674 | 2.35E-01 | 4.27E-01 |
| ERO1A | 30001 | 0.2710461 | 4.7790313 | 5.29E-01 | 7.08E-01 |
| ALDH9A1 | 223 | 0.2710455 | 6.3531092 | 3.83E-01 | 5.83E-01 |
| NKIRAS1 | 28512 | 0.2708743 | 6.7540094 | 1.35E-01 | 2.94E-01 |
| GALNT1 | 2589 | 0.2703786 | 6.8170017 | 2.56E-01 | 4.51E-01 |
| HNRNPK | 3190 | 0.2701975 | 9.575538 | 1.82E-01 | 3.60E-01 |
| CBY1 | 25776 | 0.2701835 | 5.5301043 | 2.05E-01 | 3.90E-01 |
| RTN4 | 57142 | 0.2701334 | 9.0524092 | 3.65E-01 | 5.64E-01 |
| TMEM230 | 29058 | 0.2696134 | 7.2580267 | 2.70E-01 | 4.66E-01 |
| MYO5A | 4644 | 0.2696057 | 6.8324527 | 1.79E-01 | 3.55E-01 |
| OCIAD1 | 54940 | 0.2692872 | 8.42592 | 1.69E-01 | 3.43E-01 |
| GRPEL1 | 80273 | 0.2690282 | 6.1998087 | 1.69E-01 | 3.43E-01 |
| EHMT1 | 79813 | 0.2689921 | 5.1549155 | 2.33E-01 | 4.25E-01 |
| UQCC1 | 55245 | 0.268983 | 5.9714673 | 1.95E-01 | 3.78E-01 |
| MIER2 | 54531 | 0.2689732 | 1.9060701 | 4.96E-01 | 6.82E-01 |
| CSNK1A1 | 1452 | 0.2688783 | 7.3435782 | 2.06E-01 | 3.91E-01 |
| SMAP2 | 64744 | 0.2687834 | 7.2216094 | 4.61E-01 | 6.52E-01 |
| OSBP2 | 23762 | 0.2685564 | 3.6505909 | 2.54E-01 | 4.48E-01 |
| CCT4 | 10575 | 0.2684136 | 7.8631116 | 1.29E-01 | 2.85E-01 |
| RAB35 | 11021 | 0.2679879 | 3.0655311 | 4.30E-01 | 6.27E-01 |
| FAF1 | 11124 | 0.2677936 | 5.5401793 | 2.76E-01 | 4.72E-01 |
| TPD52L2 | 7165 | 0.2677742 | 6.3647573 | 3.35E-01 | 5.37E-01 |
| LARP1 | 23367 | 0.2676253 | 6.5297418 | 1.80E-01 | 3.57E-01 |
| SSR1 | 6745 | 0.2674139 | 7.0920984 | 3.79E-01 | 5.78E-01 |
| PPID | 5481 | 0.2673587 | 5.4756005 | 1.71E-01 | 3.45E-01 |
| SLC30A6 | 55676 | 0.2673293 | 3.8741197 | 4.16E-01 | 6.15E-01 |
| PRMT6 | 55170 | 0.2672196 | 5.2265647 | 1.55E-01 | 3.21E-01 |
| RPGRIP1L | 23322 | 0.2670747 | 4.2882275 | 2.29E-01 | 4.20E-01 |
| SRPRA | 6734 | 0.2670502 | 5.8215543 | 5.08E-01 | 6.91E-01 |
| ILKAP | 80895 | 0.2670318 | 5.582619 | 1.20E-01 | 2.71E-01 |
| ACSL1 | 2180 | 0.2669278 | 4.9903302 | 2.05E-01 | 3.90E-01 |
| TRIM44 | 54765 | 0.2668306 | 7.030986 | 1.82E-01 | 3.61E-01 |
| OAT | 4942 | 0.2667133 | 8.1612734 | 1.44E-01 | 3.07E-01 |
| ADGRG1 | 9289 | 0.2665795 | 5.4419943 | 3.40E-01 | 5.41E-01 |
| ZNF227 | 7770 | 0.2665437 | 3.1549061 | 2.67E-01 | 4.62E-01 |
| CAPN10 | 11132 | 0.2660782 | 3.2627353 | 3.84E-01 | 5.84E-01 |
| TMCC2 | 9911 | 0.265512 | 4.8235256 | 4.76E-01 | 6.65E-01 |
| UBE2A | 7319 | 0.2653497 | 7.3319232 | 1.47E-01 | 3.11E-01 |
| SRR | 63826 | 0.2651086 | 4.7346823 | 2.07E-01 | 3.92E-01 |
| SAMD8 | 142891 | 0.2649736 | 5.2857575 | 2.08E-01 | 3.94E-01 |
| GOLGA4 | 2803 | 0.2646613 | 5.9282501 | 3.13E-01 | 5.12E-01 |
| SRSF1 | 6426 | 0.2646 | 3.2444196 | 3.66E-01 | 5.65E-01 |
| TSC1 | 7248 | 0.2645452 | 4.883319 | 1.73E-01 | 3.47E-01 |
| ZNF181 | 339318 | 0.2644682 | 4.4027117 | 2.04E-01 | 3.88E-01 |
| HECTD1 | 25831 | 0.2642035 | 5.949296 | 2.49E-01 | 4.43E-01 |
| HELQ | 113510 | 0.2641032 | 3.3661192 | 3.18E-01 | 5.19E-01 |
| CPPED1 | 55313 | 0.2640878 | 3.9889213 | 4.67E-01 | 6.57E-01 |
| PSD3 | 23362 | 0.2640702 | 6.2974341 | 3.62E-01 | 5.62E-01 |
| RPL23 | 9349 | 0.2639201 | 7.3887135 | 2.54E-01 | 4.48E-01 |
| BRK1 | 55845 | 0.2637986 | 8.6061493 | 2.42E-01 | 4.34E-01 |
| EXOC6B | 23233 | 0.2637477 | 4.0615684 | 2.00E-01 | 3.84E-01 |
| C16orf72 | 29035 | 0.2637379 | 4.593724 | 3.56E-01 | 5.55E-01 |
| FAM171A1 | 221061 | 0.2636898 | 5.4513869 | 2.03E-01 | 3.87E-01 |
| IVD | 3712 | 0.2636219 | 5.3416944 | 4.76E-01 | 6.65E-01 |
| PDP1 | 54704 | 0.2634634 | 6.1336338 | 4.21E-01 | 6.19E-01 |
| DDB1 | 1642 | 0.2633412 | 6.6622193 | 3.45E-01 | 5.45E-01 |
| TAPBP | 6892 | 0.2633176 | 5.3178927 | 5.34E-01 | 7.11E-01 |
| NCOA3 | 8202 | 0.2631792 | 3.2648595 | 2.62E-01 | 4.57E-01 |
| HCFC2 | 29915 | 0.2630118 | 3.747744 | 2.88E-01 | 4.87E-01 |
| IGBP1 | 3476 | 0.262928 | 5.956744 | 3.07E-01 | 5.06E-01 |
| HNRNPDL | 9987 | 0.2626348 | 8.2621342 | 2.29E-01 | 4.20E-01 |
| NOC3L | 64318 | 0.2625596 | 4.2154457 | 3.55E-01 | 5.55E-01 |
| FSIP1 | 161835 | 0.2624448 | 2.3405467 | 4.56E-01 | 6.48E-01 |
| SSB | 6741 | 0.2615221 | 7.8271127 | 2.35E-01 | 4.27E-01 |
| ZNF133 | 7692 | 0.2615013 | 4.3519597 | 1.71E-01 | 3.44E-01 |
| FBXO34 | 55030 | 0.2612747 | 5.6733873 | 2.24E-01 | 4.14E-01 |
| NUP50 | 10762 | 0.2610207 | 4.8178095 | 1.54E-01 | 3.21E-01 |
| DDX27 | 55661 | 0.2609822 | 4.9327772 | 3.03E-01 | 5.02E-01 |
| UBE3B | 89910 | 0.2607394 | 4.602367 | 1.87E-01 | 3.66E-01 |
| YY1 | 7528 | 0.2606898 | 5.6793122 | 1.72E-01 | 3.46E-01 |
| GPATCH11 | 253635 | 0.2605969 | 5.0638139 | 3.62E-01 | 5.62E-01 |
| VPS25 | 84313 | 0.2604998 | 6.9509004 | 1.93E-01 | 3.75E-01 |
| NUP107 | 57122 | 0.260498 | 4.6049443 | 2.41E-01 | 4.33E-01 |
| DNAJC9 | 23234 | 0.2604972 | 4.4317919 | 2.51E-01 | 4.45E-01 |
| RPRD1B | 58490 | 0.2601495 | 3.9952349 | 3.63E-01 | 5.62E-01 |
| CUTC | 51076 | 0.2600614 | 5.6614948 | 4.35E-01 | 6.30E-01 |
| STX5 | 6811 | 0.2599555 | 2.8415866 | 4.16E-01 | 6.15E-01 |
| ZSCAN21 | 7589 | 0.2598437 | 3.6227131 | 4.24E-01 | 6.22E-01 |
| ELF2 | 1998 | 0.2596846 | 4.5976584 | 2.19E-01 | 4.08E-01 |
| ZSCAN25 | 221785 | 0.2592161 | 3.120297 | 4.70E-01 | 6.59E-01 |
| GLT8D1 | 55830 | 0.2591895 | 5.9772211 | 3.06E-01 | 5.05E-01 |
| SH3GL1 | 6455 | 0.2590823 | 4.3443692 | 3.52E-01 | 5.51E-01 |
| ZC2HC1C | 79696 | 0.258913 | 2.4697443 | 5.29E-01 | 7.08E-01 |
| SAMHD1 | 25939 | 0.2587917 | 4.4503465 | 2.33E-01 | 4.24E-01 |
| ZNF566 | 84924 | 0.2584996 | 4.7313113 | 3.98E-01 | 5.99E-01 |
| SKIL | 6498 | 0.2584151 | 4.6556767 | 2.20E-01 | 4.09E-01 |
| MAGED4B | 81557 | 0.2583019 | 3.444594 | 3.50E-01 | 5.51E-01 |
| RAB11B | 9230 | 0.2581764 | 6.560638 | 4.50E-01 | 6.44E-01 |
| GDI1 | 2664 | 0.2579124 | 8.7399172 | 3.55E-01 | 5.54E-01 |
| MEAF6 | 64769 | 0.2575486 | 8.4534059 | 4.74E-01 | 6.63E-01 |
| GRIK2 | 2898 | 0.2574994 | 3.2527475 | 4.56E-01 | 6.47E-01 |
| RAD54L2 | 23132 | 0.2571523 | 4.7125341 | 2.83E-01 | 4.81E-01 |
| CHEK2 | 11200 | 0.2571506 | 2.3134744 | 3.81E-01 | 5.81E-01 |
| POGK | 57645 | 0.2568825 | 5.398179 | 1.85E-01 | 3.64E-01 |
| PEX1 | 5189 | 0.2568605 | 4.1674632 | 3.04E-01 | 5.03E-01 |
| ATF4 | 468 | 0.2568582 | 8.9925757 | 2.19E-01 | 4.07E-01 |
| TRIM2 | 23321 | 0.256829 | 7.2664878 | 3.69E-01 | 5.68E-01 |
| CEPT1 | 10390 | 0.256805 | 4.2094073 | 4.37E-01 | 6.32E-01 |
| TP53I11 | 9537 | 0.2567659 | 6.8127085 | 4.46E-01 | 6.39E-01 |
| FKBP7 | 51661 | 0.2566609 | 5.2867636 | 2.67E-01 | 4.63E-01 |
| TBP | 6908 | 0.2566586 | 5.69271 | 1.84E-01 | 3.63E-01 |
| PIGC | 5279 | 0.2565941 | 6.6097783 | 2.37E-01 | 4.28E-01 |
| ZNF16 | 7564 | 0.2564577 | 3.5940927 | 2.64E-01 | 4.59E-01 |
| EXT2 | 2132 | 0.2563779 | 5.8171992 | 3.12E-01 | 5.11E-01 |
| UFL1 | 23376 | 0.2560276 | 4.8601386 | 2.74E-01 | 4.71E-01 |
| NECAP1 | 25977 | 0.255755 | 7.9028233 | 4.10E-01 | 6.10E-01 |
| PPME1 | 51400 | 0.2557456 | 6.9565446 | 4.26E-01 | 6.23E-01 |
| DVL2 | 1856 | 0.2556028 | 4.0430071 | 5.01E-01 | 6.86E-01 |
| EIF2B1 | 1967 | 0.2552933 | 6.2653491 | 2.88E-01 | 4.87E-01 |
| UBE2D4 | 51619 | 0.2552904 | 3.836812 | 2.28E-01 | 4.19E-01 |
| PIK3C3 | 5289 | 0.2552626 | 4.5529477 | 4.21E-01 | 6.19E-01 |
| N4BP1 | 9683 | 0.2549757 | 3.8590543 | 2.25E-01 | 4.14E-01 |
| BAZ2A | 11176 | 0.2548734 | 4.1603603 | 2.92E-01 | 4.91E-01 |
| ZNF160 | 90338 | 0.2548693 | 4.080123 | 2.78E-01 | 4.74E-01 |
| DCXR | 51181 | 0.2548424 | 6.8451547 | 1.83E-01 | 3.61E-01 |
| OTUD4 | 54726 | 0.254763 | 3.6218595 | 3.88E-01 | 5.88E-01 |
| ERGIC1 | 57222 | 0.2547482 | 5.0793246 | 5.12E-01 | 6.95E-01 |
| HPCAL1 | 3241 | 0.2547279 | 6.5614495 | 2.49E-01 | 4.43E-01 |
| CASP8AP2 | 9994 | 0.2546886 | 5.1970858 | 3.41E-01 | 5.42E-01 |
| LANCL1 | 10314 | 0.2546777 | 5.4109389 | 2.36E-01 | 4.28E-01 |
| ARL6 | 84100 | 0.2546765 | 4.6985887 | 3.01E-01 | 4.99E-01 |
| MKKS | 8195 | 0.2543262 | 6.7937279 | 1.51E-01 | 3.16E-01 |
| SPTLC1 | 10558 | 0.254241 | 5.9985082 | 2.38E-01 | 4.30E-01 |
| ICE1 | 23379 | 0.2541031 | 4.2648522 | 2.18E-01 | 4.06E-01 |
| PYCR2 | 29920 | 0.2539544 | 3.1915365 | 5.16E-01 | 6.97E-01 |
| SEC61A2 | 55176 | 0.2538632 | 6.851633 | 3.49E-01 | 5.49E-01 |
| CLOCK | 9575 | 0.2537287 | 3.8864785 | 3.01E-01 | 4.99E-01 |
| GALNT11 | 63917 | 0.2535037 | 6.5024863 | 2.24E-01 | 4.14E-01 |
| CPSF3 | 51692 | 0.2534221 | 5.8260482 | 2.14E-01 | 4.02E-01 |
| RNF6 | 6049 | 0.2532864 | 5.1571951 | 5.20E-01 | 7.01E-01 |
| HDX | 139324 | 0.25317 | 4.0448824 | 2.87E-01 | 4.85E-01 |
| ZFYVE1 | 53349 | 0.2530427 | 4.2607062 | 4.61E-01 | 6.52E-01 |
| RAD51C | 5889 | 0.2526384 | 5.2316731 | 2.51E-01 | 4.44E-01 |
| FBXO8 | 26269 | 0.2526234 | 3.9125322 | 3.74E-01 | 5.73E-01 |
| RPS6KB1 | 6198 | 0.2526081 | 4.1084801 | 5.08E-01 | 6.91E-01 |
| DDX46 | 9879 | 0.2525716 | 6.155203 | 2.48E-01 | 4.41E-01 |
| HIBCH | 26275 | 0.2525619 | 5.5906031 | 1.72E-01 | 3.46E-01 |
| PARD3 | 56288 | 0.2524983 | 4.8851706 | 5.27E-01 | 7.07E-01 |
| CSNK2A3 | 283106 | 0.252451 | 1.4703288 | 4.48E-01 | 6.41E-01 |
| ZNF277 | 11179 | 0.2520401 | 4.4106303 | 2.35E-01 | 4.27E-01 |
| NPHP4 | 261734 | 0.2520377 | 3.0576867 | 4.97E-01 | 6.83E-01 |
| LASP1 | 3927 | 0.2519247 | 5.0604631 | 4.69E-01 | 6.59E-01 |
| NFYC | 4802 | 0.2518525 | 6.1486273 | 2.61E-01 | 4.57E-01 |
| SLAIN2 | 57606 | 0.2515361 | 4.5838173 | 2.26E-01 | 4.16E-01 |
| PHAX | 51808 | 0.2514244 | 7.1918397 | 2.46E-01 | 4.39E-01 |
| AZIN1 | 51582 | 0.2514152 | 7.5184171 | 1.68E-01 | 3.41E-01 |
| ELMOD3 | 84173 | 0.2513419 | 4.166831 | 2.48E-01 | 4.42E-01 |
| STARD7 | 56910 | 0.2509546 | 7.5752102 | 2.85E-01 | 4.83E-01 |
| CPNE3 | 8895 | 0.2501727 | 5.4555501 | 2.41E-01 | 4.33E-01 |
| SEPHS2 | 22928 | 0.2501443 | 6.5391296 | 2.32E-01 | 4.23E-01 |
| LMAN1 | 3998 | 0.2500903 | 6.1524522 | 4.37E-01 | 6.32E-01 |
| ARHGEF4 | 50649 | 0.2499446 | 4.6142411 | 2.38E-01 | 4.30E-01 |
| ACER3 | 55331 | 0.2498473 | 4.2564937 | 5.02E-01 | 6.87E-01 |
| LHFPL6 | 10186 | 0.2497564 | 5.9542547 | 5.38E-01 | 7.14E-01 |
| DENND4A | 10260 | 0.2496998 | 2.8382173 | 3.43E-01 | 5.43E-01 |
| N4BP3 | 23138 | 0.2495569 | 3.1250849 | 5.42E-01 | 7.17E-01 |
| PTK2B | 2185 | 0.2491375 | 1.9447923 | 4.17E-01 | 6.16E-01 |
| STIP1 | 10963 | 0.2489244 | 8.2587529 | 1.81E-01 | 3.59E-01 |
| MICAL3 | 57553 | 0.2485471 | 3.3292598 | 5.14E-01 | 6.95E-01 |
| SLC6A15 | 55117 | 0.2484909 | 4.923847 | 2.03E-01 | 3.88E-01 |
| SLC26A11 | 284129 | 0.2484247 | 5.0437793 | 3.28E-01 | 5.29E-01 |
| CRK | 1398 | 0.248354 | 7.3202526 | 2.11E-01 | 3.98E-01 |
| MAGEF1 | 64110 | 0.24815 | 7.3609713 | 2.15E-01 | 4.02E-01 |
| CHMP3 | 51652 | 0.2480427 | 5.1874063 | 1.73E-01 | 3.47E-01 |
| ARMCX3 | 51566 | 0.2479542 | 8.1322371 | 2.04E-01 | 3.89E-01 |
| CNP | 1267 | 0.24763 | 6.4471079 | 3.77E-01 | 5.76E-01 |
| OSBPL2 | 9885 | 0.2475271 | 5.2333212 | 2.71E-01 | 4.67E-01 |
| FUS | 2521 | 0.2474007 | 6.8272529 | 2.46E-01 | 4.39E-01 |
| WDR27 | 253769 | 0.247179 | 2.6261773 | 4.28E-01 | 6.26E-01 |
| CDC37L1 | 55664 | 0.2470859 | 5.9255756 | 2.83E-01 | 4.81E-01 |
| INPP5K | 51763 | 0.246975 | 4.9814758 | 3.60E-01 | 5.59E-01 |
| ARHGAP26 | 23092 | 0.2469056 | 4.1580531 | 3.91E-01 | 5.91E-01 |
| TM9SF2 | 9375 | 0.2468267 | 7.9981846 | 2.63E-01 | 4.58E-01 |
| ARL8A | 127829 | 0.2467733 | 4.2672266 | 5.35E-01 | 7.12E-01 |
| MED28 | 80306 | 0.2466707 | 6.5226467 | 1.64E-01 | 3.35E-01 |
| S1PR2 | 9294 | 0.2464417 | 2.2739815 | 5.34E-01 | 7.11E-01 |
| EFR3B | 22979 | 0.2462695 | 5.154294 | 4.16E-01 | 6.16E-01 |
| MFSD1 | 64747 | 0.2461531 | 5.4974056 | 4.10E-01 | 6.10E-01 |
| GPR161 | 23432 | 0.2451357 | 6.4269347 | 3.80E-01 | 5.80E-01 |
| C1orf43 | 25912 | 0.2450183 | 8.2903845 | 1.67E-01 | 3.39E-01 |
| RXYLT1 | 10329 | 0.2449847 | 4.3070334 | 2.25E-01 | 4.14E-01 |
| TRAPPC6B | 122553 | 0.2449808 | 4.4900588 | 4.24E-01 | 6.23E-01 |
| YY1AP1 | 55249 | 0.2444346 | 4.4922246 | 2.70E-01 | 4.66E-01 |
| NFXL1 | 152518 | 0.2439728 | 3.1424623 | 4.29E-01 | 6.26E-01 |
| ZNF286A | 57335 | 0.2438323 | 4.152762 | 4.10E-01 | 6.10E-01 |
| C8orf48 | 157773 | 0.2438228 | 3.7558347 | 4.33E-01 | 6.28E-01 |
| ASIC1 | 41 | 0.243694 | 3.7582818 | 4.35E-01 | 6.30E-01 |
| HERPUD1 | 9709 | 0.2431419 | 7.7203039 | 3.87E-01 | 5.87E-01 |
| ZNF362 | 149076 | 0.2430845 | 5.0875647 | 3.40E-01 | 5.41E-01 |
| SNU13 | 4809 | 0.2428404 | 8.7660383 | 2.47E-01 | 4.40E-01 |
| CDV3 | 55573 | 0.2427934 | 5.4022656 | 2.97E-01 | 4.95E-01 |
| CS | 1431 | 0.242499 | 6.9875672 | 2.26E-01 | 4.16E-01 |
| ZNF415 | 55786 | 0.2422814 | 5.058309 | 3.71E-01 | 5.71E-01 |
| FOCAD | 54914 | 0.24203 | 4.7380653 | 2.73E-01 | 4.69E-01 |
| TWNK | 56652 | 0.2418985 | 2.0493902 | 4.85E-01 | 6.72E-01 |
| JMJD1C | 221037 | 0.2418269 | 6.2359677 | 3.15E-01 | 5.14E-01 |
| ZNF483 | 158399 | 0.2417768 | 3.3192986 | 4.55E-01 | 6.47E-01 |
| SARAF | 51669 | 0.2412181 | 10.700329 | 2.10E-01 | 3.96E-01 |
| PTRHD1 | 391356 | 0.2411454 | 4.4592652 | 2.76E-01 | 4.73E-01 |
| SEC61A1 | 29927 | 0.2409412 | 6.9908209 | 5.32E-01 | 7.10E-01 |
| SPEF1 | 25876 | 0.2407505 | 2.5705991 | 4.94E-01 | 6.81E-01 |
| DYNC1LI2 | 1783 | 0.2407044 | 8.2109351 | 2.51E-01 | 4.45E-01 |
| DCAF7 | 10238 | 0.2405622 | 6.3615437 | 1.91E-01 | 3.71E-01 |
| POLR1E | 64425 | 0.2405511 | 3.9997194 | 2.81E-01 | 4.79E-01 |
| TRIB2 | 28951 | 0.2404643 | 6.9769477 | 3.87E-01 | 5.87E-01 |
| ATL2 | 64225 | 0.2403292 | 5.0358689 | 3.46E-01 | 5.46E-01 |
| EFCAB7 | 84455 | 0.2402958 | 4.5646717 | 2.27E-01 | 4.17E-01 |
| MPP3 | 4356 | 0.2402736 | 5.3388687 | 2.12E-01 | 3.99E-01 |
| TMEM170B | 100113407 | 0.2402156 | 5.1542062 | 3.03E-01 | 5.02E-01 |
| PRXL2B | 127281 | 0.2397441 | 4.8952237 | 3.51E-01 | 5.51E-01 |
| CLCN7 | 1186 | 0.2397112 | 3.9437281 | 5.21E-01 | 7.02E-01 |
| ZNF12 | 7559 | 0.2396497 | 5.121838 | 2.91E-01 | 4.89E-01 |
| AHI1 | 54806 | 0.2395428 | 6.178751 | 4.12E-01 | 6.11E-01 |
| CCDC32 | 90416 | 0.2389885 | 4.3066422 | 3.93E-01 | 5.93E-01 |
| NRF1 | 4899 | 0.2389653 | 2.9222906 | 5.37E-01 | 7.14E-01 |
| TRAPPC11 | 60684 | 0.2384365 | 4.0173762 | 3.50E-01 | 5.51E-01 |
| MZT1 | 440145 | 0.238386 | 5.5869517 | 4.82E-01 | 6.70E-01 |
| SYT14 | 255928 | 0.2383401 | 4.5624588 | 2.87E-01 | 4.85E-01 |
| SMKR1 | 100287482 | 0.2381548 | 4.6474434 | 4.59E-01 | 6.51E-01 |
| MAP3K7 | 6885 | 0.2381413 | 5.4879686 | 2.92E-01 | 4.90E-01 |
| AKIP1 | 56672 | 0.2381132 | 6.4952413 | 2.15E-01 | 4.03E-01 |
| TMEM80 | 283232 | 0.2378344 | 4.1354385 | 3.05E-01 | 5.04E-01 |
| ZKSCAN5 | 23660 | 0.237556 | 3.7352 | 4.30E-01 | 6.27E-01 |
| FAM217B | 63939 | 0.2372455 | 5.3185877 | 4.62E-01 | 6.52E-01 |
| MOB3A | 126308 | 0.236742 | 5.1047207 | 3.53E-01 | 5.53E-01 |
| RFC2 | 5982 | 0.2366737 | 5.1402472 | 2.62E-01 | 4.57E-01 |
| SLC38A9 | 153129 | 0.2364923 | 4.3219157 | 2.75E-01 | 4.71E-01 |
| MFN1 | 55669 | 0.2362775 | 4.264627 | 3.56E-01 | 5.56E-01 |
| SAP18 | 10284 | 0.2362375 | 9.0387391 | 3.05E-01 | 5.04E-01 |
| TRNT1 | 51095 | 0.2356618 | 4.9689925 | 2.98E-01 | 4.97E-01 |
| NUDCD2 | 134492 | 0.2353644 | 5.8089674 | 2.70E-01 | 4.66E-01 |
| ENSA | 2029 | 0.2352703 | 7.1366108 | 3.36E-01 | 5.37E-01 |
| MTREX | 23517 | 0.235148 | 5.1408844 | 2.06E-01 | 3.91E-01 |
| PRKAB1 | 5564 | 0.2349847 | 5.4281825 | 2.61E-01 | 4.56E-01 |
| FAM219B | 57184 | 0.234968 | 4.604404 | 3.44E-01 | 5.45E-01 |
| METTL16 | 79066 | 0.234943 | 3.8538605 | 3.61E-01 | 5.61E-01 |
| RASA2 | 5922 | 0.2342244 | 3.2998823 | 5.08E-01 | 6.91E-01 |
| MACROH2A2 | 55506 | 0.2341867 | 7.4971878 | 4.20E-01 | 6.19E-01 |
| C6orf52 | 347744 | 0.2341829 | 4.339131 | 5.40E-01 | 7.16E-01 |
| PRPF3 | 9129 | 0.2337918 | 4.8713269 | 3.12E-01 | 5.11E-01 |
| DPP3 | 10072 | 0.2337397 | 4.6594684 | 3.90E-01 | 5.90E-01 |
| RSL1D1 | 26156 | 0.2336249 | 7.5134588 | 2.12E-01 | 3.98E-01 |
| MANF | 7873 | 0.233397 | 6.0014916 | 2.80E-01 | 4.78E-01 |
| CWF19L2 | 143884 | 0.2332258 | 3.65455 | 4.50E-01 | 6.43E-01 |
| FAM161B | 145483 | 0.2330665 | 4.1644283 | 4.44E-01 | 6.38E-01 |
| TOMM20 | 9804 | 0.2330463 | 6.8751099 | 2.37E-01 | 4.28E-01 |
| GPANK1 | 7918 | 0.2330085 | 4.5642411 | 3.56E-01 | 5.56E-01 |
| SRI | 6717 | 0.2328606 | 8.0663706 | 2.22E-01 | 4.12E-01 |
| ZDHHC21 | 340481 | 0.2325733 | 3.8422755 | 3.22E-01 | 5.23E-01 |
| TENM4 | 26011 | 0.2323062 | 3.9376816 | 3.97E-01 | 5.98E-01 |
| SLC30A4 | 7782 | 0.2320105 | 3.6372981 | 5.00E-01 | 6.85E-01 |
| ZNF793 | 390927 | 0.2320052 | 4.6903345 | 4.64E-01 | 6.54E-01 |
| HBS1L | 10767 | 0.2317437 | 6.3921271 | 2.08E-01 | 3.94E-01 |
| GTPBP3 | 84705 | 0.2314113 | 3.3610963 | 4.10E-01 | 6.10E-01 |
| GNL3 | 26354 | 0.2313434 | 6.7777154 | 3.17E-01 | 5.17E-01 |
| ATG5 | 9474 | 0.2312792 | 5.7303544 | 2.64E-01 | 4.59E-01 |
| PUM1 | 9698 | 0.2307459 | 7.055468 | 2.01E-01 | 3.85E-01 |
| ILF2 | 3608 | 0.2306445 | 8.9008295 | 2.27E-01 | 4.17E-01 |
| ITSN1 | 6453 | 0.2305167 | 7.6857299 | 3.94E-01 | 5.94E-01 |
| SLC36A4 | 120103 | 0.2303785 | 6.8417083 | 2.67E-01 | 4.62E-01 |
| DHRS4 | 10901 | 0.2303327 | 2.6994562 | 4.21E-01 | 6.19E-01 |
| ERCC6L2 | 375748 | 0.2301479 | 5.0790686 | 2.61E-01 | 4.57E-01 |
| NARF | 26502 | 0.2300143 | 6.7292523 | 3.35E-01 | 5.36E-01 |
| YTHDF2 | 51441 | 0.2296917 | 7.6073869 | 1.89E-01 | 3.69E-01 |
| RTL6 | 84247 | 0.2295056 | 5.5663324 | 4.43E-01 | 6.36E-01 |
| RPL6 | 6128 | 0.2293978 | 7.6994586 | 2.65E-01 | 4.61E-01 |
| LRRC27 | 80313 | 0.2293286 | 5.0807807 | 2.53E-01 | 4.48E-01 |
| CIAO1 | 9391 | 0.2291672 | 6.3885694 | 2.25E-01 | 4.15E-01 |
| FASTKD2 | 22868 | 0.2291209 | 5.7837862 | 2.94E-01 | 4.92E-01 |
| ATP6V1G1 | 9550 | 0.2287217 | 9.3639732 | 2.82E-01 | 4.80E-01 |
| DMXL2 | 23312 | 0.2286698 | 4.6330236 | 3.09E-01 | 5.08E-01 |
| FBXL4 | 26235 | 0.2282447 | 3.8212103 | 4.02E-01 | 6.02E-01 |
| USP10 | 9100 | 0.2282075 | 6.4619033 | 2.69E-01 | 4.65E-01 |
| C19orf44 | 84167 | 0.2281902 | 2.1068655 | 5.23E-01 | 7.04E-01 |
| WDR37 | 22884 | 0.2280442 | 5.2367814 | 2.87E-01 | 4.85E-01 |
| GORAB | 92344 | 0.2280121 | 3.8893506 | 3.52E-01 | 5.51E-01 |
| ACTR2 | 10097 | 0.2278554 | 7.3143474 | 2.50E-01 | 4.43E-01 |
| LYPLAL1 | 127018 | 0.2276243 | 5.9200904 | 2.49E-01 | 4.43E-01 |
| ORC4 | 5000 | 0.2275937 | 6.9902252 | 2.38E-01 | 4.30E-01 |
| SART1 | 9092 | 0.2270891 | 4.3618012 | 3.58E-01 | 5.58E-01 |
| APOE | 348 | 0.2263839 | 5.4700815 | 4.31E-01 | 6.28E-01 |
| CEP250 | 11190 | 0.2263367 | 4.6182147 | 3.27E-01 | 5.27E-01 |
| AUTS2 | 26053 | 0.2262654 | 5.8728192 | 4.09E-01 | 6.09E-01 |
| GNE | 10020 | 0.2260737 | 4.5488372 | 2.88E-01 | 4.86E-01 |
| INPP4A | 3631 | 0.2257967 | 4.8546125 | 2.84E-01 | 4.82E-01 |
| NT5C | 30833 | 0.225766 | 4.173151 | 2.80E-01 | 4.77E-01 |
| CDK13 | 8621 | 0.2256731 | 4.237536 | 4.31E-01 | 6.27E-01 |
| XRCC6 | 2547 | 0.2250632 | 8.6663941 | 2.33E-01 | 4.25E-01 |
| MEX3C | 51320 | 0.2250299 | 4.0056169 | 3.28E-01 | 5.29E-01 |
| CACYBP | 27101 | 0.2249799 | 8.6367815 | 4.46E-01 | 6.40E-01 |
| ATP6V0A1 | 535 | 0.2249409 | 7.6718797 | 2.73E-01 | 4.70E-01 |
| CREB1 | 1385 | 0.224859 | 6.1605893 | 2.28E-01 | 4.18E-01 |
| VPS26B | 112936 | 0.224833 | 6.3691461 | 3.38E-01 | 5.38E-01 |
| LCMT2 | 9836 | 0.2247529 | 4.258973 | 2.43E-01 | 4.35E-01 |
| WASHC4 | 23325 | 0.2246112 | 4.815307 | 5.09E-01 | 6.92E-01 |
| MBOAT2 | 129642 | 0.2244889 | 5.8988676 | 2.00E-01 | 3.84E-01 |
| ZXDC | 79364 | 0.224318 | 3.6854763 | 3.48E-01 | 5.48E-01 |
| CEP83 | 51134 | 0.2242372 | 3.3947394 | 4.79E-01 | 6.67E-01 |
| GNL2 | 29889 | 0.2241961 | 6.2676689 | 3.32E-01 | 5.33E-01 |
| IRS2 | 8660 | 0.2235863 | 4.8029485 | 5.09E-01 | 6.92E-01 |
| TMEM183A | 92703 | 0.2233612 | 6.9102022 | 3.61E-01 | 5.60E-01 |
| ALG6 | 29929 | 0.2233123 | 4.487408 | 3.13E-01 | 5.12E-01 |
| ATP6V0E1 | 8992 | 0.2230822 | 7.5117352 | 4.74E-01 | 6.63E-01 |
| MPV17L2 | 84769 | 0.2229725 | 4.8760997 | 4.10E-01 | 6.10E-01 |
| TTLL7 | 79739 | 0.2228186 | 5.4461659 | 3.20E-01 | 5.21E-01 |
| MRFAP1 | 93621 | 0.2226735 | 9.2799568 | 2.18E-01 | 4.06E-01 |
| GGA3 | 23163 | 0.2223739 | 3.0062135 | 4.07E-01 | 6.07E-01 |
| ATPSCKMT | 134145 | 0.2223076 | 4.0892515 | 2.72E-01 | 4.68E-01 |
| ZNF146 | 7705 | 0.2221824 | 6.0755759 | 2.56E-01 | 4.50E-01 |
| YARS1 | 8565 | 0.2216056 | 7.0922679 | 3.37E-01 | 5.38E-01 |
| NFU1 | 27247 | 0.2215564 | 7.8269851 | 4.75E-01 | 6.64E-01 |
| SPRYD7 | 57213 | 0.2215255 | 5.8947102 | 3.48E-01 | 5.48E-01 |
| BCCIP | 56647 | 0.2214922 | 6.4972782 | 3.32E-01 | 5.33E-01 |
| BTG1 | 694 | 0.2213772 | 6.1240738 | 2.75E-01 | 4.71E-01 |
| AKTIP | 64400 | 0.2213218 | 5.5832892 | 2.72E-01 | 4.68E-01 |
| TIGD7 | 91151 | 0.2212545 | 2.3194522 | 4.62E-01 | 6.52E-01 |
| NOS1AP | 9722 | 0.2210817 | 4.0205525 | 4.28E-01 | 6.25E-01 |
| RTRAF | 51637 | 0.2209462 | 7.2871864 | 2.26E-01 | 4.16E-01 |
| PATJ | 10207 | 0.2209284 | 3.8210668 | 4.19E-01 | 6.18E-01 |
| APTX | 54840 | 0.2209056 | 5.4898005 | 2.65E-01 | 4.61E-01 |
| MUL1 | 79594 | 0.2207759 | 5.7621511 | 3.51E-01 | 5.51E-01 |
| NAA20 | 51126 | 0.2206279 | 7.5372575 | 2.66E-01 | 4.62E-01 |
| EEF1B2 | 1933 | 0.2205351 | 8.3072628 | 2.63E-01 | 4.58E-01 |
| NANOS1 | 340719 | 0.2205001 | 3.4576166 | 4.06E-01 | 6.06E-01 |
| JAKMIP2 | 9832 | 0.2201616 | 6.3703965 | 5.03E-01 | 6.88E-01 |
| GABRG3 | 2567 | 0.2198734 | 5.4270676 | 3.52E-01 | 5.51E-01 |
| LRRN1 | 57633 | 0.2196175 | 5.2377702 | 5.12E-01 | 6.95E-01 |
| PPP2R3C | 55012 | 0.2195774 | 5.9242516 | 3.41E-01 | 5.42E-01 |
| TRIM11 | 81559 | 0.2195731 | 3.5304423 | 3.99E-01 | 5.99E-01 |
| FLII | 2314 | 0.2195059 | 4.9305627 | 5.19E-01 | 7.00E-01 |
| VPS50 | 55610 | 0.2194232 | 5.0886909 | 2.56E-01 | 4.51E-01 |
| COIL | 8161 | 0.21882 | 5.8020946 | 3.23E-01 | 5.24E-01 |
| THOC2 | 57187 | 0.2187413 | 6.1465811 | 2.49E-01 | 4.43E-01 |
| ZNF573 | 126231 | 0.2186992 | 4.7523381 | 3.06E-01 | 5.06E-01 |
| CEP89 | 84902 | 0.2185508 | 5.4932965 | 2.96E-01 | 4.94E-01 |
| CBWD2 | 150472 | 0.218399 | 6.4557866 | 3.42E-01 | 5.43E-01 |
| GNAQ | 2776 | 0.2183513 | 6.6400454 | 3.56E-01 | 5.55E-01 |
| HEXD | 284004 | 0.2182682 | 3.793207 | 4.38E-01 | 6.32E-01 |
| MIDN | 90007 | 0.2178754 | 4.7650559 | 4.33E-01 | 6.28E-01 |
| FAHD1 | 81889 | 0.2178455 | 6.6252877 | 2.72E-01 | 4.68E-01 |
| ZNF615 | 284370 | 0.2177486 | 3.2522256 | 4.58E-01 | 6.49E-01 |
| RSPRY1 | 89970 | 0.2175805 | 6.3094164 | 2.58E-01 | 4.53E-01 |
| PPIL1 | 51645 | 0.2174961 | 7.0357993 | 3.57E-01 | 5.56E-01 |
| ZNF365 | 22891 | 0.2173351 | 2.515087 | 4.83E-01 | 6.70E-01 |
| EXOSC1 | 51013 | 0.2172499 | 5.9234643 | 2.55E-01 | 4.49E-01 |
| BORCS5 | 118426 | 0.2170935 | 4.3767168 | 3.78E-01 | 5.78E-01 |
| RBM39 | 9584 | 0.2170447 | 7.2681331 | 2.72E-01 | 4.68E-01 |
| FITM2 | 128486 | 0.2167298 | 3.883496 | 3.64E-01 | 5.63E-01 |
| TTC1 | 7265 | 0.2163528 | 7.223502 | 2.48E-01 | 4.41E-01 |
| PPP1R18 | 107987457 | 0.2161487 | 3.406147 | 3.92E-01 | 5.93E-01 |
| DESI2 | 51029 | 0.2160816 | 5.137746 | 2.94E-01 | 4.92E-01 |
| LEMD3 | 23592 | 0.2160518 | 4.465575 | 3.46E-01 | 5.46E-01 |
| TRNAU1AP | 54952 | 0.2158119 | 5.313147 | 3.12E-01 | 5.11E-01 |
| PSMG2 | 56984 | 0.2157746 | 6.1995629 | 3.22E-01 | 5.23E-01 |
| TAF6 | 6878 | 0.2154531 | 4.6391046 | 3.90E-01 | 5.90E-01 |
| ZNF250 | 58500 | 0.2154507 | 4.752447 | 5.20E-01 | 7.01E-01 |
| TRPM7 | 54822 | 0.2151673 | 3.2032683 | 5.41E-01 | 7.16E-01 |
| TMEM87A | 25963 | 0.2151506 | 5.9433912 | 3.50E-01 | 5.50E-01 |
| ZNF567 | 163081 | 0.2145385 | 4.2862025 | 3.59E-01 | 5.58E-01 |
| ZNF473 | 25888 | 0.2143331 | 3.7338178 | 5.14E-01 | 6.96E-01 |
| TRIP4 | 9325 | 0.2143 | 4.9272775 | 2.50E-01 | 4.43E-01 |
| PDCD5 | 9141 | 0.2142326 | 7.7027275 | 4.71E-01 | 6.60E-01 |
| ASB8 | 140461 | 0.2140987 | 6.3908316 | 2.74E-01 | 4.70E-01 |
| ZDHHC12 | 84885 | 0.2140207 | 3.1293378 | 4.65E-01 | 6.55E-01 |
| ZDHHC17 | 23390 | 0.2140127 | 6.0978515 | 4.24E-01 | 6.22E-01 |
| GTF2IRD2B | 389524 | 0.2139171 | 3.5068114 | 3.63E-01 | 5.62E-01 |
| RNF7 | 9616 | 0.2134016 | 7.7630678 | 3.61E-01 | 5.60E-01 |
| SFMBT1 | 51460 | 0.2132187 | 4.6448804 | 2.64E-01 | 4.60E-01 |
| KNSTRN | 90417 | 0.2131398 | 4.8857234 | 4.13E-01 | 6.13E-01 |
| TMED10 | 10972 | 0.2129389 | 8.1917842 | 3.25E-01 | 5.25E-01 |
| MOCS3 | 27304 | 0.2129229 | 4.0360721 | 4.17E-01 | 6.16E-01 |
| TMEM258 | 746 | 0.2126809 | 7.1118417 | 4.59E-01 | 6.51E-01 |
| UNK | 85451 | 0.2126633 | 5.6581119 | 2.60E-01 | 4.56E-01 |
| DUT | 1854 | 0.2125674 | 6.3316843 | 4.64E-01 | 6.55E-01 |
| ZMYM5 | 9205 | 0.2123701 | 5.2288297 | 3.42E-01 | 5.43E-01 |
| ZNF839 | 55778 | 0.2122667 | 2.0430334 | 5.11E-01 | 6.94E-01 |
| NLE1 | 54475 | 0.212119 | 3.9342347 | 5.16E-01 | 6.97E-01 |
| BIVM | 54841 | 0.2119682 | 3.94846 | 4.30E-01 | 6.27E-01 |
| METTL17 | 64745 | 0.2119607 | 4.6762527 | 4.00E-01 | 6.00E-01 |
| TCF4 | 6925 | 0.2113827 | 5.3674251 | 5.12E-01 | 6.95E-01 |
| NCKIPSD | 51517 | 0.2113307 | 3.8458852 | 5.35E-01 | 7.12E-01 |
| ARMC10 | 83787 | 0.2112343 | 5.9133115 | 2.81E-01 | 4.79E-01 |
| METTL6 | 131965 | 0.2109666 | 5.3454177 | 2.89E-01 | 4.88E-01 |
| C20orf27 | 54976 | 0.2109259 | 6.040821 | 3.42E-01 | 5.43E-01 |
| TET2 | 54790 | 0.2108602 | 5.3342621 | 3.91E-01 | 5.92E-01 |
| FBXL14 | 144699 | 0.2108095 | 3.4232089 | 4.14E-01 | 6.13E-01 |
| ZNF32 | 7580 | 0.210778 | 6.0886435 | 4.14E-01 | 6.14E-01 |
| TRAPPC13 | 80006 | 0.2103397 | 4.7003704 | 3.23E-01 | 5.24E-01 |
| DCUN1D1 | 54165 | 0.2103014 | 5.6845313 | 2.63E-01 | 4.58E-01 |
| SNX24 | 28966 | 0.2100871 | 4.9180857 | 4.52E-01 | 6.45E-01 |
| GTF2A2 | 2958 | 0.2100062 | 8.1995603 | 3.24E-01 | 5.25E-01 |
| PHACTR4 | 65979 | 0.2091737 | 5.1904338 | 3.76E-01 | 5.75E-01 |
| TBPL1 | 9519 | 0.2088377 | 6.2383858 | 4.71E-01 | 6.60E-01 |
| CD99L2 | 83692 | 0.2084794 | 5.3174334 | 3.62E-01 | 5.62E-01 |
| ZNF502 | 91392 | 0.2082732 | 3.9097537 | 3.27E-01 | 5.27E-01 |
| PCNX1 | 22990 | 0.2082289 | 3.2684707 | 4.68E-01 | 6.58E-01 |
| ZNF639 | 51193 | 0.2082157 | 5.6605572 | 2.59E-01 | 4.54E-01 |
| MTERF2 | 80298 | 0.2081612 | 3.8746959 | 3.94E-01 | 5.94E-01 |
| PSMD3 | 5709 | 0.2080299 | 5.5440463 | 2.95E-01 | 4.93E-01 |
| PIGT | 51604 | 0.2079854 | 6.7987744 | 4.93E-01 | 6.79E-01 |
| ARNT2 | 9915 | 0.2079852 | 5.1600665 | 4.60E-01 | 6.51E-01 |
| PSMC5 | 5705 | 0.207881 | 7.4019544 | 2.98E-01 | 4.96E-01 |
| MAP3K2 | 10746 | 0.2073827 | 4.9090944 | 3.39E-01 | 5.40E-01 |
| AFF3 | 3899 | 0.2071821 | 4.9943556 | 4.87E-01 | 6.74E-01 |
| SMC5 | 23137 | 0.207081 | 5.6780667 | 4.60E-01 | 6.51E-01 |
| OTUD5 | 55593 | 0.2070677 | 5.3403133 | 3.90E-01 | 5.90E-01 |
| VOPP1 | 81552 | 0.2070172 | 5.8186432 | 3.69E-01 | 5.68E-01 |
| HMGN2 | 3151 | 0.2066123 | 8.5231131 | 5.07E-01 | 6.91E-01 |
| ASAH2B | 653308 | 0.2064943 | 3.2668942 | 4.48E-01 | 6.41E-01 |
| SLC35E2B | 728661 | 0.2063822 | 4.6305871 | 4.33E-01 | 6.28E-01 |
| SRPK2 | 6733 | 0.2058842 | 7.3863471 | 4.73E-01 | 6.62E-01 |
| TCTA | 6988 | 0.2058534 | 5.8051 | 3.26E-01 | 5.26E-01 |
| RPL15 | 6138 | 0.2057918 | 10.360448 | 3.26E-01 | 5.27E-01 |
| PDCD6IP | 10015 | 0.2056582 | 5.4700347 | 5.06E-01 | 6.90E-01 |
| CRY2 | 1408 | 0.2055993 | 4.6768642 | 4.77E-01 | 6.65E-01 |
| USP54 | 159195 | 0.2052674 | 2.4304324 | 4.59E-01 | 6.51E-01 |
| NME6 | 10201 | 0.2050183 | 5.7379734 | 2.84E-01 | 4.82E-01 |
| TM9SF3 | 56889 | 0.2049168 | 6.8090009 | 5.02E-01 | 6.87E-01 |
| PHF8 | 23133 | 0.2048336 | 3.0562923 | 5.35E-01 | 7.12E-01 |
| NIT1 | 4817 | 0.204779 | 3.943531 | 4.17E-01 | 6.16E-01 |
| SAFB2 | 9667 | 0.2046791 | 6.0819734 | 2.92E-01 | 4.90E-01 |
| PSMB2 | 5690 | 0.204475 | 8.4729733 | 3.77E-01 | 5.76E-01 |
| PRR3 | 80742 | 0.2044219 | 5.6797278 | 4.10E-01 | 6.10E-01 |
| MRPS33 | 51650 | 0.2042533 | 6.7706825 | 4.03E-01 | 6.04E-01 |
| MBD1 | 4152 | 0.2038549 | 5.1907926 | 4.19E-01 | 6.18E-01 |
| KPNA2 | 3838 | 0.2034182 | 9.1160422 | 3.91E-01 | 5.91E-01 |
| THAP11 | 57215 | 0.2033515 | 5.3841684 | 2.91E-01 | 4.90E-01 |
| HELZ | 9931 | 0.2032252 | 5.5153551 | 3.89E-01 | 5.90E-01 |
| MAEA | 10296 | 0.2029503 | 6.7810975 | 2.52E-01 | 4.46E-01 |
| RALA | 5898 | 0.2027306 | 7.0494905 | 4.41E-01 | 6.35E-01 |
| METTL9 | 51108 | 0.2023606 | 7.9644396 | 4.55E-01 | 6.47E-01 |
| MRPS10 | 55173 | 0.2021787 | 6.4316237 | 2.95E-01 | 4.94E-01 |
| KAT14 | 57325 | 0.2020051 | 3.9707976 | 3.30E-01 | 5.31E-01 |
| DCAKD | 79877 | 0.2018805 | 5.6327233 | 3.17E-01 | 5.17E-01 |
| CPSF7 | 79869 | 0.2018173 | 2.7662527 | 4.97E-01 | 6.83E-01 |
| AGBL5 | 60509 | 0.2017901 | 5.727738 | 3.57E-01 | 5.56E-01 |
| ZC3H13 | 23091 | 0.2016753 | 7.3314566 | 3.89E-01 | 5.89E-01 |
| DDX17 | 10521 | 0.2015888 | 5.9586026 | 3.52E-01 | 5.51E-01 |
| TRMT44 | 152992 | 0.2014777 | 2.1928969 | 5.26E-01 | 7.06E-01 |
| CSTF2 | 1478 | 0.2009634 | 4.2876762 | 3.15E-01 | 5.15E-01 |
| PRPF40A | 55660 | 0.2009429 | 7.6409085 | 2.84E-01 | 4.82E-01 |
| XKR6 | 286046 | 0.20042 | 4.755591 | 4.08E-01 | 6.08E-01 |
| TPPP | 11076 | 0.2003719 | 3.9074531 | 4.80E-01 | 6.68E-01 |
| WARS2 | 10352 | 0.1999841 | 3.1963434 | 4.07E-01 | 6.07E-01 |
| RAB40C | 57799 | 0.1998064 | 4.9807488 | 2.96E-01 | 4.94E-01 |
| RBMX | 27316 | 0.1997455 | 7.2139144 | 2.67E-01 | 4.62E-01 |
| SLC38A2 | 54407 | 0.1990659 | 7.0370914 | 4.56E-01 | 6.48E-01 |
| MIER3 | 166968 | 0.1990178 | 3.3683374 | 4.33E-01 | 6.28E-01 |
| TAF7 | 6879 | 0.1987813 | 8.1318253 | 2.70E-01 | 4.66E-01 |
| GTPBP10 | 85865 | 0.1984086 | 3.7070753 | 4.62E-01 | 6.53E-01 |
| PPP2R5E | 5529 | 0.1983228 | 6.015438 | 4.39E-01 | 6.33E-01 |
| ZNF148 | 7707 | 0.1982196 | 6.4494087 | 2.71E-01 | 4.67E-01 |
| CDPF1 | 150383 | 0.1981067 | 4.1385072 | 4.22E-01 | 6.20E-01 |
| RSL24D1 | 51187 | 0.1981033 | 7.8143459 | 4.37E-01 | 6.32E-01 |
| SHC3 | 53358 | 0.1979054 | 6.1155136 | 4.44E-01 | 6.38E-01 |
| ZC3H18 | 124245 | 0.1978645 | 3.1337437 | 4.80E-01 | 6.68E-01 |
| SPRED2 | 200734 | 0.1977653 | 4.3101546 | 4.79E-01 | 6.68E-01 |
| TIGD1 | 200765 | 0.1976864 | 4.3684711 | 3.87E-01 | 5.88E-01 |
| BBS9 | 27241 | 0.1975355 | 4.142626 | 4.54E-01 | 6.46E-01 |
| ISCA1 | 81689 | 0.1974266 | 7.2895486 | 2.81E-01 | 4.79E-01 |
| UBXN4 | 23190 | 0.197212 | 7.3928067 | 3.62E-01 | 5.62E-01 |
| ASB6 | 140459 | 0.1971757 | 4.6424008 | 3.74E-01 | 5.73E-01 |
| RSPH3 | 83861 | 0.1970563 | 4.4038594 | 4.26E-01 | 6.24E-01 |
| AZI2 | 64343 | 0.1970224 | 5.8415157 | 3.01E-01 | 5.00E-01 |
| OSBP | 5007 | 0.1970095 | 5.0833089 | 4.70E-01 | 6.60E-01 |
| MAIP1 | 79568 | 0.1969928 | 4.7673858 | 3.27E-01 | 5.28E-01 |
| CAMSAP2 | 23271 | 0.1965636 | 5.4138472 | 3.72E-01 | 5.71E-01 |
| UTP6 | 55813 | 0.1964569 | 5.3458332 | 4.34E-01 | 6.30E-01 |
| CCDC6 | 8030 | 0.1962918 | 4.9662994 | 5.13E-01 | 6.95E-01 |
| NPEPPS | 9520 | 0.1961544 | 6.6158305 | 3.23E-01 | 5.24E-01 |
| DUSP14 | 11072 | 0.1961099 | 6.0464492 | 4.70E-01 | 6.59E-01 |
| ENOX1 | 55068 | 0.1957628 | 4.7636208 | 3.53E-01 | 5.53E-01 |
| SMIM20 | 389203 | 0.1955621 | 5.4733868 | 3.28E-01 | 5.29E-01 |
| TIMM10B | 26515 | 0.1954496 | 5.2783604 | 5.11E-01 | 6.93E-01 |
| KDM4C | 23081 | 0.195433 | 3.4458603 | 4.48E-01 | 6.41E-01 |
| USP48 | 84196 | 0.1953836 | 5.9421329 | 3.81E-01 | 5.81E-01 |
| DECR2 | 26063 | 0.1953362 | 4.0831612 | 3.38E-01 | 5.39E-01 |
| RNF167 | 26001 | 0.1950752 | 4.880892 | 3.42E-01 | 5.43E-01 |
| FOXJ3 | 22887 | 0.1950388 | 5.3941016 | 3.97E-01 | 5.98E-01 |
| ZNF275 | 10838 | 0.195022 | 3.7496791 | 4.19E-01 | 6.18E-01 |
| ZNF554 | 115196 | 0.1949651 | 4.3000262 | 3.96E-01 | 5.96E-01 |
| WBP1L | 54838 | 0.1948234 | 4.4980055 | 4.35E-01 | 6.30E-01 |
| NUP58 | 9818 | 0.1946052 | 4.8445276 | 3.87E-01 | 5.87E-01 |
| PRPF6 | 24148 | 0.1944957 | 6.4672131 | 3.41E-01 | 5.42E-01 |
| GSTP1 | 2950 | 0.1941742 | 9.5810416 | 3.09E-01 | 5.08E-01 |
| ZNF232 | 7775 | 0.1941642 | 5.2902917 | 4.83E-01 | 6.71E-01 |
| EDRF1 | 26098 | 0.1941082 | 3.4682868 | 3.93E-01 | 5.93E-01 |
| FAM126B | 285172 | 0.1939051 | 4.992832 | 5.25E-01 | 7.05E-01 |
| GON4L | 54856 | 0.193683 | 6.0663157 | 4.27E-01 | 6.24E-01 |
| PACSIN2 | 11252 | 0.1933966 | 4.5935517 | 4.97E-01 | 6.83E-01 |
| RAP1GDS1 | 5910 | 0.1932272 | 6.4095683 | 5.08E-01 | 6.91E-01 |
| DEDD | 9191 | 0.1930701 | 5.4569767 | 3.26E-01 | 5.26E-01 |
| KIF2A | 3796 | 0.1930636 | 6.5692315 | 4.19E-01 | 6.18E-01 |
| PSMD6 | 9861 | 0.1930193 | 7.6344587 | 2.74E-01 | 4.70E-01 |
| TMCO6 | 55374 | 0.1927665 | 2.081962 | 5.13E-01 | 6.95E-01 |
| SPAG7 | 9552 | 0.1924982 | 7.7158585 | 3.50E-01 | 5.51E-01 |
| IGF2BP1 | 10642 | 0.1923351 | 2.4323897 | 5.19E-01 | 7.00E-01 |
| NUTF2 | 10204 | 0.1921595 | 8.2195085 | 2.97E-01 | 4.95E-01 |
| HECTD2 | 143279 | 0.1921178 | 4.8099568 | 4.19E-01 | 6.18E-01 |
| ANAPC10 | 10393 | 0.1921031 | 3.7904924 | 5.16E-01 | 6.98E-01 |
| MAGED4 | 728239 | 0.1915454 | 3.5998379 | 4.35E-01 | 6.30E-01 |
| ZNF629 | 23361 | 0.1915269 | 3.8481935 | 4.80E-01 | 6.68E-01 |
| RPL21 | 6144 | 0.1912611 | 9.0434814 | 4.52E-01 | 6.45E-01 |
| LYRM1 | 57149 | 0.1912528 | 6.9449127 | 3.58E-01 | 5.57E-01 |
| C17orf58 | 284018 | 0.1909924 | 4.5407162 | 5.09E-01 | 6.92E-01 |
| ZNF611 | 81856 | 0.190981 | 4.3831678 | 3.43E-01 | 5.44E-01 |
| ZFP91 | 80829 | 0.1909675 | 3.9860409 | 3.26E-01 | 5.27E-01 |
| RNF34 | 80196 | 0.1908147 | 6.0295353 | 2.78E-01 | 4.74E-01 |
| ZCCHC9 | 84240 | 0.1907953 | 3.921731 | 4.34E-01 | 6.29E-01 |
| NUP54 | 53371 | 0.1902787 | 5.3225811 | 3.22E-01 | 5.23E-01 |
| USP42 | 84132 | 0.1902473 | 4.2947416 | 3.88E-01 | 5.88E-01 |
| ADSS2 | 159 | 0.1899086 | 5.3761845 | 5.37E-01 | 7.14E-01 |
| REC8 | 9985 | 0.1893079 | 3.4958887 | 4.78E-01 | 6.67E-01 |
| SNUPN | 10073 | 0.1891954 | 4.6451456 | 3.38E-01 | 5.39E-01 |
| SOWAHC | 65124 | 0.1890913 | 3.8179399 | 4.85E-01 | 6.73E-01 |
| SLC4A1AP | 22950 | 0.1890073 | 6.2311542 | 3.17E-01 | 5.17E-01 |
| XPO5 | 57510 | 0.1888655 | 4.065834 | 4.59E-01 | 6.51E-01 |
| UQCC2 | 84300 | 0.1882092 | 6.6606445 | 4.03E-01 | 6.04E-01 |
| TRMT5 | 57570 | 0.1880827 | 4.1404226 | 4.80E-01 | 6.68E-01 |
| SPAST | 6683 | 0.187988 | 4.6854703 | 5.08E-01 | 6.91E-01 |
| HMGCL | 3155 | 0.1878392 | 5.5907271 | 4.74E-01 | 6.63E-01 |
| PAK1 | 5058 | 0.1875204 | 5.8944215 | 5.32E-01 | 7.10E-01 |
| SACS | 26278 | 0.1872166 | 4.6180855 | 5.41E-01 | 7.16E-01 |
| CHORDC1 | 26973 | 0.186981 | 6.2309158 | 3.52E-01 | 5.52E-01 |
| SMG7 | 9887 | 0.1868641 | 5.1959973 | 4.50E-01 | 6.43E-01 |
| KPNB1 | 3837 | 0.1867985 | 5.8384952 | 2.95E-01 | 4.94E-01 |
| EIF3F | 8665 | 0.1860722 | 6.7202315 | 2.88E-01 | 4.86E-01 |
| STRIP1 | 85369 | 0.1858607 | 5.9965031 | 4.11E-01 | 6.10E-01 |
| UGDH | 7358 | 0.1858298 | 5.3375623 | 5.24E-01 | 7.04E-01 |
| N4BP2L2 | 10443 | 0.1856566 | 6.6453194 | 3.76E-01 | 5.76E-01 |
| SLC23A2 | 9962 | 0.1854214 | 5.3230035 | 3.93E-01 | 5.93E-01 |
| COG8 | 84342 | 0.1851224 | 2.7087625 | 4.86E-01 | 6.73E-01 |
| ASB3 | 100302652 | 0.184142 | 5.9024333 | 3.08E-01 | 5.07E-01 |
| COA5 | 493753 | 0.183536 | 6.0936514 | 3.69E-01 | 5.68E-01 |
| TWF1 | 5756 | 0.183411 | 5.6209569 | 4.53E-01 | 6.45E-01 |
| RBM48 | 84060 | 0.1834078 | 4.8617323 | 3.13E-01 | 5.13E-01 |
| SELENOT | 51714 | 0.1829441 | 8.4007686 | 4.19E-01 | 6.18E-01 |
| TMEM170A | 124491 | 0.1824969 | 4.7267587 | 3.48E-01 | 5.48E-01 |
| MRNIP | 51149 | 0.1824437 | 4.2405486 | 4.11E-01 | 6.10E-01 |
| POLR2D | 5433 | 0.18243 | 4.9677989 | 4.42E-01 | 6.36E-01 |
| TMEM187 | 8269 | 0.1820532 | 2.8777385 | 4.91E-01 | 6.78E-01 |
| DNAJC27 | 51277 | 0.1820125 | 3.8374732 | 4.42E-01 | 6.36E-01 |
| MAGED2 | 10916 | 0.1818818 | 9.4748923 | 3.42E-01 | 5.43E-01 |
| SIAH1 | 6477 | 0.1815048 | 5.0138676 | 5.31E-01 | 7.10E-01 |
| MORF4L1 | 10933 | 0.1812938 | 10.422063 | 5.25E-01 | 7.06E-01 |
| VPS72 | 6944 | 0.1812325 | 7.1862371 | 4.30E-01 | 6.27E-01 |
| ZNF84 | 7637 | 0.1809624 | 5.458656 | 3.78E-01 | 5.78E-01 |
| PIGZ | 80235 | 0.1807063 | 3.9124989 | 3.85E-01 | 5.85E-01 |
| EDEM3 | 80267 | 0.1805233 | 4.6686042 | 4.46E-01 | 6.40E-01 |
| PEMT | 10400 | 0.1804662 | 5.4193438 | 3.72E-01 | 5.71E-01 |
| ABHD10 | 55347 | 0.1801991 | 7.1621144 | 4.10E-01 | 6.10E-01 |
| RAD21 | 5885 | 0.1797922 | 6.4459068 | 4.04E-01 | 6.05E-01 |
| NSD2 | 7468 | 0.1791586 | 5.9906481 | 4.60E-01 | 6.52E-01 |
| GTF2F2 | 2963 | 0.1789478 | 5.6700208 | 3.35E-01 | 5.36E-01 |
| EFNB3 | 1949 | 0.17832 | 4.8789148 | 4.88E-01 | 6.75E-01 |
| TRIM35 | 23087 | 0.1781979 | 3.5506465 | 4.56E-01 | 6.48E-01 |
| E4F1 | 1877 | 0.177925 | 4.2915254 | 4.33E-01 | 6.28E-01 |
| PHF23 | 79142 | 0.1778138 | 5.8182056 | 3.72E-01 | 5.71E-01 |
| RBPJ | 3516 | 0.1777976 | 7.2813798 | 3.08E-01 | 5.08E-01 |
| YIPF4 | 84272 | 0.1777965 | 6.2397702 | 3.90E-01 | 5.90E-01 |
| ARF4 | 378 | 0.1774232 | 9.4118609 | 3.63E-01 | 5.62E-01 |
| UBE3A | 7337 | 0.1773171 | 6.7178708 | 3.67E-01 | 5.66E-01 |
| ZBTB43 | 23099 | 0.1772265 | 5.4471083 | 4.08E-01 | 6.08E-01 |
| SYS1 | 90196 | 0.1771626 | 5.2476687 | 4.11E-01 | 6.10E-01 |
| RNF25 | 64320 | 0.1769245 | 5.644824 | 3.90E-01 | 5.90E-01 |
| MANSC1 | 54682 | 0.1766501 | 4.3718062 | 4.56E-01 | 6.47E-01 |
| SERINC1 | 57515 | 0.1763538 | 9.7823292 | 3.64E-01 | 5.63E-01 |
| RFX3 | 5991 | 0.1760865 | 4.8965024 | 3.76E-01 | 5.76E-01 |
| PSMD11 | 5717 | 0.1759601 | 7.8484917 | 3.30E-01 | 5.31E-01 |
| PHF20L1 | 51105 | 0.1752579 | 5.6368183 | 4.38E-01 | 6.33E-01 |
| PLXNA1 | 5361 | 0.1751678 | 6.5021794 | 4.09E-01 | 6.09E-01 |
| CYB5R3 | 1727 | 0.1750122 | 7.1169733 | 4.42E-01 | 6.36E-01 |
| UBLCP1 | 134510 | 0.17498 | 6.4973497 | 3.57E-01 | 5.56E-01 |
| COPS5 | 10987 | 0.1743397 | 7.118835 | 3.37E-01 | 5.38E-01 |
| PDCL | 5082 | 0.1742786 | 4.9766683 | 3.47E-01 | 5.48E-01 |
| POLR3GL | 84265 | 0.174206 | 4.0802011 | 4.74E-01 | 6.63E-01 |
| ZNF431 | 170959 | 0.1740266 | 3.7130943 | 5.07E-01 | 6.91E-01 |
| HSD17B12 | 51144 | 0.1738527 | 6.4558329 | 5.29E-01 | 7.08E-01 |
| SRSF10 | 10772 | 0.1734295 | 7.1958371 | 3.42E-01 | 5.43E-01 |
| DCAF10 | 79269 | 0.1729497 | 5.731813 | 3.86E-01 | 5.86E-01 |
| PHKB | 5257 | 0.1727509 | 4.866217 | 4.68E-01 | 6.58E-01 |
| NUP133 | 55746 | 0.1722873 | 5.1095395 | 4.38E-01 | 6.32E-01 |
| CNNM4 | 26504 | 0.1722843 | 3.4231435 | 5.32E-01 | 7.11E-01 |
| PRPF38A | 84950 | 0.1719548 | 5.6402859 | 3.37E-01 | 5.38E-01 |
| MRPL9 | 65005 | 0.1713773 | 5.4552746 | 3.40E-01 | 5.41E-01 |
| FRAT2 | 23401 | 0.170996 | 3.1289427 | 5.25E-01 | 7.05E-01 |
| MED29 | 55588 | 0.1703221 | 7.0583825 | 3.55E-01 | 5.54E-01 |
| ZFP62 | 643836 | 0.170112 | 3.7315206 | 5.33E-01 | 7.11E-01 |
| STK40 | 83931 | 0.1700921 | 5.0948818 | 4.13E-01 | 6.12E-01 |
| KDM5B | 10765 | 0.1699688 | 7.7077773 | 4.09E-01 | 6.08E-01 |
| PPM1G | 5496 | 0.1696852 | 7.3816039 | 3.78E-01 | 5.78E-01 |
| UBE2L6 | 9246 | 0.1694365 | 7.5466717 | 3.47E-01 | 5.48E-01 |
| STX18 | 53407 | 0.169423 | 5.3139552 | 4.55E-01 | 6.47E-01 |
| MAPKAPK5 | 8550 | 0.1693596 | 4.9883807 | 3.78E-01 | 5.78E-01 |
| TIMMDC1 | 51300 | 0.1692797 | 6.9425778 | 4.30E-01 | 6.27E-01 |
| MFF | 56947 | 0.1685114 | 7.6370211 | 3.90E-01 | 5.90E-01 |
| WDR70 | 55100 | 0.1684491 | 4.745496 | 4.10E-01 | 6.10E-01 |
| ANKRD13C | 81573 | 0.1682032 | 5.5433389 | 4.85E-01 | 6.73E-01 |
| RNF11 | 26994 | 0.1679701 | 6.3498785 | 3.93E-01 | 5.93E-01 |
| PPP1R15B | 84919 | 0.1677173 | 4.2744777 | 5.24E-01 | 7.05E-01 |
| PTBP3 | 9991 | 0.1676236 | 4.8321032 | 5.02E-01 | 6.87E-01 |
| HNRNPF | 3185 | 0.1670795 | 6.8882509 | 4.83E-01 | 6.71E-01 |
| EIF3E | 3646 | 0.1668703 | 7.0022739 | 4.30E-01 | 6.27E-01 |
| FN3KRP | 79672 | 0.16674 | 5.4439706 | 4.25E-01 | 6.23E-01 |
| EXOSC9 | 5393 | 0.16656 | 5.1488295 | 4.25E-01 | 6.23E-01 |
| ABHD12 | 26090 | 0.1665573 | 6.9311076 | 3.36E-01 | 5.37E-01 |
| WDR89 | 112840 | 0.1662354 | 3.742963 | 4.61E-01 | 6.52E-01 |
| PCDHGB6 | 56100 | 0.1661415 | 4.8547188 | 5.23E-01 | 7.03E-01 |
| SDAD1 | 55153 | 0.1660832 | 6.5316946 | 4.12E-01 | 6.12E-01 |
| QRICH1 | 54870 | 0.1660505 | 4.9402384 | 3.87E-01 | 5.88E-01 |
| SLC2A6 | 11182 | 0.1655432 | 5.7940991 | 4.10E-01 | 6.10E-01 |
| ZNF544 | 27300 | 0.1654361 | 3.8470719 | 4.30E-01 | 6.27E-01 |
| OSCP1 | 127700 | 0.1652654 | 4.5742198 | 4.91E-01 | 6.77E-01 |
| DDIT3 | 1649 | 0.1651825 | 5.9006134 | 5.10E-01 | 6.93E-01 |
| JRKL | 8690 | 0.1650551 | 3.9444053 | 5.09E-01 | 6.92E-01 |
| TSPYL2 | 64061 | 0.1647849 | 5.9691919 | 4.30E-01 | 6.27E-01 |
| MMGT1 | 93380 | 0.1643776 | 3.7090773 | 4.87E-01 | 6.74E-01 |
| SFT2D1 | 113402 | 0.1643498 | 5.9649447 | 4.98E-01 | 6.84E-01 |
| ATG16L1 | 55054 | 0.1642901 | 4.7258768 | 4.00E-01 | 6.01E-01 |
| LRSAM1 | 90678 | 0.1636318 | 5.164583 | 4.07E-01 | 6.07E-01 |
| IARS2 | 55699 | 0.163564 | 5.5813853 | 5.19E-01 | 7.00E-01 |
| CSTF2T | 23283 | 0.1629788 | 4.9481539 | 4.91E-01 | 6.78E-01 |
| TTC7B | 145567 | 0.162701 | 4.3291188 | 4.10E-01 | 6.10E-01 |
| BMPR1A | 657 | 0.1622549 | 4.4106704 | 4.85E-01 | 6.73E-01 |
| KHDRBS1 | 10657 | 0.1621801 | 7.6788795 | 4.96E-01 | 6.82E-01 |
| MSL2 | 55167 | 0.1618987 | 3.4834141 | 4.83E-01 | 6.70E-01 |
| SMARCC2 | 6601 | 0.1611807 | 6.6713287 | 5.24E-01 | 7.04E-01 |
| SET | 6418 | 0.1598693 | 7.250533 | 4.80E-01 | 6.68E-01 |
| AACS | 65985 | 0.1594812 | 4.267415 | 4.92E-01 | 6.78E-01 |
| ARPC2 | 10109 | 0.1591257 | 9.2638021 | 3.89E-01 | 5.89E-01 |
| ADAR | 103 | 0.159079 | 5.8066093 | 3.78E-01 | 5.78E-01 |
| CYP51A1 | 1595 | 0.1588072 | 4.5812792 | 4.97E-01 | 6.83E-01 |
| BRIX1 | 55299 | 0.1587947 | 6.0378444 | 4.64E-01 | 6.55E-01 |
| EMC8 | 10328 | 0.1580733 | 6.0604582 | 3.51E-01 | 5.51E-01 |
| RNF141 | 50862 | 0.1577815 | 6.1332079 | 4.41E-01 | 6.35E-01 |
| PACRGL | 133015 | 0.1569573 | 3.8789113 | 5.38E-01 | 7.14E-01 |
| USP5 | 8078 | 0.15634 | 6.0213764 | 4.95E-01 | 6.82E-01 |
| RPL10A | 4736 | 0.1561421 | 7.0655511 | 4.35E-01 | 6.30E-01 |
| IFT140 | 9742 | 0.1561248 | 3.3668398 | 5.08E-01 | 6.91E-01 |
| RPUSD4 | 84881 | 0.155642 | 4.0067348 | 5.25E-01 | 7.05E-01 |
| ATP1B3 | 483 | 0.1553947 | 9.3157225 | 4.11E-01 | 6.10E-01 |
| PEX11B | 8799 | 0.1552826 | 6.1390798 | 4.24E-01 | 6.22E-01 |
| ZNF672 | 79894 | 0.1547183 | 5.0141368 | 4.49E-01 | 6.43E-01 |
| CNDP2 | 55748 | 0.1541073 | 6.5465502 | 4.50E-01 | 6.43E-01 |
| NISCH | 11188 | 0.1539324 | 5.6898816 | 4.54E-01 | 6.46E-01 |
| MLST8 | 64223 | 0.1536698 | 5.9788327 | 4.31E-01 | 6.28E-01 |
| CNOT10 | 25904 | 0.1527903 | 4.6354761 | 4.68E-01 | 6.58E-01 |
| HAUS6 | 54801 | 0.1524738 | 4.2120143 | 5.40E-01 | 7.16E-01 |
| SMARCE1 | 6605 | 0.1523021 | 3.1950158 | 5.40E-01 | 7.16E-01 |
| DCAF1 | 9730 | 0.1519602 | 4.0141127 | 5.36E-01 | 7.12E-01 |
| NAA80 | 24142 | 0.1518603 | 3.7422269 | 5.41E-01 | 7.16E-01 |
| MTFR1L | 56181 | 0.1518446 | 6.7713095 | 3.93E-01 | 5.93E-01 |
| ZNF320 | 162967 | 0.1517 | 3.8919921 | 4.86E-01 | 6.73E-01 |
| SGK1 | 6446 | 0.151417 | 4.3980148 | 5.13E-01 | 6.95E-01 |
| NPAT | 4863 | 0.1514099 | 4.2703787 | 5.05E-01 | 6.89E-01 |
| AARS1 | 16 | 0.1512322 | 6.8494309 | 4.97E-01 | 6.83E-01 |
| RAP1A | 5906 | 0.1504501 | 4.4410372 | 4.93E-01 | 6.79E-01 |
| CHCHD3 | 54927 | 0.1503584 | 6.8524444 | 4.44E-01 | 6.38E-01 |
| GPAT4 | 137964 | 0.1502458 | 5.2212401 | 4.37E-01 | 6.32E-01 |
| AQP11 | 282679 | 0.1492418 | 4.3966555 | 5.16E-01 | 6.97E-01 |
| HNRNPA1 | 3178 | 0.1490347 | 8.5400248 | 4.32E-01 | 6.28E-01 |
| PAPOLG | 64895 | 0.1486138 | 4.2902879 | 4.75E-01 | 6.64E-01 |
| UBE4B | 10277 | 0.148308 | 4.1942745 | 4.85E-01 | 6.73E-01 |
| CAND1 | 55832 | 0.1482391 | 6.7165822 | 5.09E-01 | 6.92E-01 |
| RRP8 | 23378 | 0.1481917 | 3.9315674 | 4.69E-01 | 6.59E-01 |
| C9orf78 | 51759 | 0.1481584 | 6.9163339 | 4.43E-01 | 6.37E-01 |
| CHMP5 | 51510 | 0.147694 | 8.0844846 | 4.46E-01 | 6.39E-01 |
| PRXL2A | 84293 | 0.1476262 | 8.5489773 | 4.50E-01 | 6.44E-01 |
| COQ6 | 51004 | 0.1470025 | 4.037945 | 5.37E-01 | 7.14E-01 |
| IFNAR1 | 3454 | 0.1469646 | 5.7366762 | 4.52E-01 | 6.44E-01 |
| EWSR1 | 2130 | 0.1469116 | 8.2535758 | 4.32E-01 | 6.28E-01 |
| UBE2F | 140739 | 0.1460732 | 5.5058271 | 4.34E-01 | 6.30E-01 |
| METTL2A | 339175 | 0.1458758 | 5.722148 | 4.51E-01 | 6.44E-01 |
| DPH7 | 92715 | 0.1455462 | 4.9180969 | 4.32E-01 | 6.28E-01 |
| ELOVL6 | 79071 | 0.1451939 | 5.3504218 | 4.96E-01 | 6.82E-01 |
| DCAF5 | 8816 | 0.1447901 | 6.0040861 | 4.57E-01 | 6.48E-01 |
| NIF3L1 | 60491 | 0.1445983 | 5.5601903 | 4.82E-01 | 6.70E-01 |
| EXOSC2 | 23404 | 0.1444728 | 4.9760517 | 4.98E-01 | 6.84E-01 |
| SMYD2 | 56950 | 0.1444267 | 5.0471707 | 5.09E-01 | 6.92E-01 |
| CES2 | 8824 | 0.1441485 | 4.8284072 | 5.04E-01 | 6.89E-01 |
| ZNF398 | 57541 | 0.1441254 | 4.2740454 | 5.08E-01 | 6.91E-01 |
| PCMT1 | 5110 | 0.144067 | 8.8561459 | 5.33E-01 | 7.11E-01 |
| RNMT | 8731 | 0.1440216 | 5.9370024 | 5.32E-01 | 7.10E-01 |
| SERGEF | 26297 | 0.1433284 | 4.5868999 | 4.83E-01 | 6.71E-01 |
| PEF1 | 553115 | 0.1432507 | 7.0001579 | 4.79E-01 | 6.68E-01 |
| GPRC5B | 51704 | 0.1421688 | 4.3983852 | 5.24E-01 | 7.04E-01 |
| TUBG1 | 7283 | 0.1417263 | 6.6591825 | 4.64E-01 | 6.54E-01 |
| C14orf119 | 55017 | 0.14168 | 7.2240439 | 4.61E-01 | 6.52E-01 |
| ERGIC2 | 51290 | 0.1415823 | 6.5854483 | 4.45E-01 | 6.39E-01 |
| GOLGA7 | 51125 | 0.1409744 | 5.4360174 | 4.47E-01 | 6.41E-01 |
| STARD3 | 10948 | 0.1409534 | 5.6102172 | 4.77E-01 | 6.65E-01 |
| AATF | 26574 | 0.1409275 | 5.4969814 | 4.75E-01 | 6.64E-01 |
| GNPTAB | 79158 | 0.1406638 | 5.8822627 | 5.26E-01 | 7.06E-01 |
| TRIM13 | 10206 | 0.1404615 | 5.9225554 | 5.21E-01 | 7.02E-01 |
| COMMD2 | 51122 | 0.1403907 | 6.6825683 | 4.42E-01 | 6.36E-01 |
| PSMB1 | 5689 | 0.14025 | 8.8569581 | 4.65E-01 | 6.55E-01 |
| SQLE | 6713 | 0.140132 | 8.4589315 | 4.35E-01 | 6.30E-01 |
| CCNC | 892 | 0.1398986 | 5.9877378 | 4.83E-01 | 6.71E-01 |
| GUF1 | 60558 | 0.1398535 | 4.7626111 | 4.65E-01 | 6.55E-01 |
| DHRS13 | 147015 | 0.1398508 | 4.261394 | 4.95E-01 | 6.81E-01 |
| KPNA6 | 23633 | 0.1392503 | 5.3984912 | 4.80E-01 | 6.68E-01 |
| KLHL28 | 54813 | 0.1390863 | 5.2927689 | 4.50E-01 | 6.43E-01 |
| SREK1 | 140890 | 0.1388559 | 6.2411508 | 5.03E-01 | 6.88E-01 |
| SLC35E3 | 55508 | 0.1378453 | 5.6363453 | 4.22E-01 | 6.20E-01 |
| CAPZA2 | 830 | 0.1377319 | 8.4434157 | 4.56E-01 | 6.47E-01 |
| MCRS1 | 10445 | 0.1376328 | 6.0310114 | 4.61E-01 | 6.52E-01 |
| SLC25A44 | 9673 | 0.1375655 | 5.8566115 | 5.00E-01 | 6.86E-01 |
| SF3B2 | 10992 | 0.1375651 | 6.0271179 | 5.25E-01 | 7.05E-01 |
| ZSCAN18 | 65982 | 0.137496 | 5.7096838 | 4.34E-01 | 6.29E-01 |
| HNRNPAB | 3182 | 0.1374641 | 6.877092 | 4.80E-01 | 6.68E-01 |
| ZDHHC3 | 51304 | 0.1369062 | 5.8375846 | 4.67E-01 | 6.56E-01 |
| SRSF11 | 9295 | 0.1365802 | 7.2986595 | 4.90E-01 | 6.77E-01 |
| PCIF1 | 63935 | 0.1365276 | 5.3878822 | 4.80E-01 | 6.68E-01 |
| SENP5 | 205564 | 0.1354152 | 4.0200774 | 5.27E-01 | 7.06E-01 |
| NAA15 | 80155 | 0.1353037 | 5.6436671 | 5.02E-01 | 6.87E-01 |
| ZNF200 | 7752 | 0.1344737 | 4.1807877 | 5.31E-01 | 7.10E-01 |
| CEP68 | 23177 | 0.1344616 | 5.0465921 | 5.34E-01 | 7.12E-01 |
| PPM1A | 5494 | 0.1344199 | 4.6499674 | 5.26E-01 | 7.06E-01 |
| ZCCHC8 | 55596 | 0.1336822 | 4.0720825 | 5.13E-01 | 6.95E-01 |
| MFAP3 | 4238 | 0.133557 | 5.1635491 | 5.03E-01 | 6.88E-01 |
| DAP3 | 7818 | 0.1329343 | 6.5419062 | 4.71E-01 | 6.60E-01 |
| DEDD2 | 162989 | 0.1312095 | 5.6547655 | 4.66E-01 | 6.56E-01 |
| NFYB | 4801 | 0.1310358 | 5.5658427 | 4.56E-01 | 6.48E-01 |
| RTF2 | 51507 | 0.1309584 | 6.9216765 | 4.92E-01 | 6.78E-01 |
| LYRM2 | 57226 | 0.1297857 | 7.1309026 | 5.12E-01 | 6.95E-01 |
| RARS2 | 57038 | 0.1290419 | 6.5534994 | 4.55E-01 | 6.47E-01 |
| EIF2B4 | 8890 | 0.1272859 | 6.0262498 | 5.15E-01 | 6.97E-01 |
| PTDSS1 | 9791 | 0.1270229 | 7.0344011 | 5.03E-01 | 6.88E-01 |
| KRR1 | 11103 | 0.1269152 | 5.529118 | 5.41E-01 | 7.16E-01 |
| PGLS | 25796 | 0.1266263 | 6.2291667 | 4.90E-01 | 6.77E-01 |
| MED4 | 29079 | 0.1245221 | 6.2288463 | 5.34E-01 | 7.12E-01 |
| TMEM14C | 51522 | 0.1239112 | 7.5750442 | 5.10E-01 | 6.93E-01 |
| CPVL | 54504 | 0.1237416 | 5.2928833 | 5.38E-01 | 7.14E-01 |
| MARCHF5 | 54708 | 0.1229484 | 6.3786989 | 4.82E-01 | 6.70E-01 |
| GTF2E2 | 2961 | 0.1214357 | 5.887153 | 5.05E-01 | 6.89E-01 |
| NFKBIE | 4794 | 0.1210047 | 4.4556896 | 5.37E-01 | 7.14E-01 |
| ITFG1 | 81533 | 0.1209573 | 7.3606839 | 5.06E-01 | 6.90E-01 |
| MTERF4 | 130916 | 0.1202008 | 4.4745607 | 5.34E-01 | 7.11E-01 |
| SMS | 6611 | 0.1194731 | 8.3795431 | 5.26E-01 | 7.06E-01 |
| TRIM33 | 51592 | 0.1184384 | 5.7721279 | 5.15E-01 | 6.97E-01 |
| FAM118B | 79607 | 0.1180467 | 5.6979923 | 5.40E-01 | 7.16E-01 |
| TPT1 | 7178 | 0.11728 | 8.2762204 | 5.31E-01 | 7.10E-01 |
| CAPZB | 832 | 0.1118434 | 7.2273265 | 5.33E-01 | 7.11E-01 |
| WDR41 | 55255 | 0.1100261 | 6.7545598 | 5.26E-01 | 7.06E-01 |
| TLE1 | 7088 | 0.1089187 | 5.7042019 | 5.33E-01 | 7.11E-01 |
| ARFGAP3 | 26286 | 0.1055474 | 5.6785295 | 5.41E-01 | 7.16E-01 |
| MTLN | 205251 | -13.40062 | 5.0066409 | 9.93E-114 | 1.31E-109 |
| LAMP5 | 24141 | -9.903583 | 3.7349793 | 2.65E-08 | 8.77E-07 |
| VSTM1 | 284415 | -9.694353 | 1.0602628 | 2.36E-07 | 6.02E-06 |
| CALCR | 799 | -8.959288 | 3.7219683 | 2.82E-61 | 1.87E-57 |
| PDCD1 | 5133 | -8.876263 | 0.1848134 | 3.20E-17 | 4.87E-15 |
| CCL4L2 | 388372 | -8.853497 | 0.091946 | 3.09E-21 | 7.86E-19 |
| PPP1R3A | 5506 | -8.827275 | 0.154166 | 2.31E-07 | 5.92E-06 |
| HTR6 | 3362 | -8.742735 | 0.0448951 | 7.04E-18 | 1.18E-15 |
| MMEL1 | 79258 | -8.055584 | -0.36568 | 8.23E-08 | 2.42E-06 |
| PITX2 | 5308 | -7.946381 | 0.1387137 | 1.71E-06 | 3.44E-05 |
| HTN1 | 3346 | -7.911379 | 1.9364039 | 2.29E-05 | 3.24E-04 |
| KCNS3 | 3790 | -7.84164 | 1.8795432 | 3.28E-06 | 6.03E-05 |
| SMIM24 | 284422 | -7.825818 | 2.5970744 | 1.31E-05 | 2.02E-04 |
| DRD1 | 1812 | -7.813953 | 3.8287904 | 5.45E-08 | 1.66E-06 |
| AVP | 551 | -7.681107 | 1.5078994 | 5.47E-11 | 3.00E-09 |
| TFF3 | 7033 | -7.195228 | 2.0630116 | 1.78E-10 | 9.01E-09 |
| PDYN | 5173 | -7.188956 | 9.1102517 | 3.79E-05 | 4.93E-04 |
| FST | 10468 | -7.09729 | 2.1615324 | 1.49E-16 | 1.95E-14 |
| PRDM8 | 56978 | -7.043684 | 0.1932925 | 1.90E-03 | 1.24E-02 |
| INHBA | 3624 | -6.999281 | 3.7417533 | 2.49E-09 | 1.03E-07 |
| SLC2A5 | 6518 | -6.851484 | 2.520927 | 2.33E-43 | 3.43E-40 |
| RAMP1 | 10267 | -6.839735 | 5.8300198 | 1.03E-54 | 3.41E-51 |
| FAM181A | 90050 | -6.615714 | 2.1952005 | 6.76E-07 | 1.52E-05 |
| BCL2A1 | 597 | -6.61454 | 1.3442997 | 3.68E-08 | 1.18E-06 |
| POU5F1 | 5460 | -6.55611 | 4.8141916 | 1.25E-11 | 7.47E-10 |
| ALX1 | 8092 | -6.22386 | 0.4907646 | 3.30E-07 | 8.09E-06 |
| LYNX1-SLURP2 | 111188157 | -6.160371 | 0.0040934 | 2.02E-11 | 1.15E-09 |
| SST | 6750 | -6.154397 | 9.170788 | 7.62E-25 | 2.88E-22 |
| CHRM1 | 1128 | -6.005995 | 0.8811055 | 1.35E-03 | 9.39E-03 |
| IGF1 | 3479 | -5.976489 | 3.2081687 | 1.32E-15 | 1.48E-13 |
| NOTCH1 | 4851 | -5.97273 | 0.8701045 | 1.14E-04 | 1.26E-03 |
| SLA | 6503 | -5.909973 | 3.0985286 | 2.43E-12 | 1.73E-10 |
| NR2E1 | 7101 | -5.862284 | -0.084015 | 3.78E-05 | 4.92E-04 |
| OXT | 5020 | -5.854918 | 2.7305851 | 9.83E-14 | 8.79E-12 |
| KRT23 | 25984 | -5.846179 | -0.085538 | 6.36E-14 | 6.05E-12 |
| AMBN | 258 | -5.833966 | 3.8050844 | 1.29E-07 | 3.56E-06 |
| LYNX1 | 66004 | -5.806103 | 2.5218026 | 9.86E-17 | 1.36E-14 |
| ARHGAP36 | 158763 | -5.786709 | 7.5759239 | 1.87E-07 | 4.90E-06 |
| MBNL3 | 55796 | -5.784625 | -0.45503 | 1.74E-04 | 1.77E-03 |
| ELOVL2 | 54898 | -5.738472 | 3.9863171 | 3.25E-10 | 1.58E-08 |
| NR2F2 | 7026 | -5.710608 | 5.1692896 | 1.10E-29 | 5.41E-27 |
| CD34 | 947 | -5.560616 | 2.0343831 | 5.18E-03 | 2.76E-02 |
| NPY | 4852 | -5.554083 | 5.4479504 | 5.40E-04 | 4.49E-03 |
| RPRML | 388394 | -5.550346 | 2.8788465 | 4.04E-31 | 2.43E-28 |
| SOSTDC1 | 25928 | -5.535625 | 3.9921013 | 8.93E-09 | 3.30E-07 |
| MEIS2 | 4212 | -5.526008 | 7.1122739 | 3.72E-14 | 3.65E-12 |
| NTSR1 | 4923 | -5.524113 | 2.3936257 | 2.61E-05 | 3.61E-04 |
| OR7D2 | 162998 | -5.490295 | 0.0801238 | 6.17E-17 | 8.68E-15 |
| GSTT2B | 653689 | -5.445755 | 0.6946599 | 4.10E-04 | 3.62E-03 |
| F3 | 2152 | -5.430795 | 0.1277894 | 5.08E-04 | 4.27E-03 |
| AOAH | 313 | -5.409715 | 2.6990857 | 6.54E-16 | 7.59E-14 |
| CX3CR1 | 1524 | -5.400786 | 2.3123761 | 1.77E-24 | 6.34E-22 |
| NDP | 4693 | -5.37037 | 4.5749854 | 7.57E-10 | 3.45E-08 |
| IFIH1 | 64135 | -5.27034 | -0.187045 | 8.53E-03 | 4.02E-02 |
| POU3F4 | 5456 | -5.269407 | 2.9929069 | 9.68E-11 | 5.11E-09 |
| FGF10 | 2255 | -5.228898 | 1.2810878 | 9.20E-14 | 8.46E-12 |
| NPNT | 255743 | -5.223236 | 3.7878772 | 3.74E-16 | 4.59E-14 |
| NRGN | 4900 | -5.147143 | 1.4641243 | 2.22E-06 | 4.30E-05 |
| HLA-C | 3107 | -5.126571 | 6.4202935 | 8.98E-23 | 2.64E-20 |
| NR2F1 | 7025 | -5.123678 | 4.5964199 | 3.82E-36 | 3.37E-33 |
| TCIM | 56892 | -5.121188 | 5.3629326 | 9.87E-13 | 7.34E-11 |
| HGF | 3082 | -5.052257 | 0.5912147 | 3.52E-12 | 2.40E-10 |
| KCNJ2 | 3759 | -5.032452 | 2.3837237 | 7.27E-07 | 1.63E-05 |
| RSPO2 | 340419 | -4.981205 | 2.7486451 | 5.40E-18 | 9.28E-16 |
| CRH | 1392 | -4.946192 | 7.2366895 | 4.55E-05 | 5.78E-04 |
| PROK2 | 60675 | -4.944677 | 1.5592274 | 4.61E-04 | 3.95E-03 |
| SOX8 | 30812 | -4.934499 | 1.3313338 | 1.07E-05 | 1.70E-04 |
| GSTM1 | 2944 | -4.934222 | 0.4279086 | 2.11E-14 | 2.13E-12 |
| SH3RF2 | 153769 | -4.907943 | 1.2102572 | 3.17E-08 | 1.04E-06 |
| SIGIRR | 59307 | -4.890191 | 3.3616442 | 1.73E-05 | 2.56E-04 |
| ADRA1D | 146 | -4.875506 | -0.213854 | 9.77E-13 | 7.31E-11 |
| PRKCH | 5583 | -4.811764 | 1.9487839 | 2.27E-05 | 3.21E-04 |
| PLAAT4 | 5920 | -4.788636 | 0.4447812 | 6.30E-03 | 3.21E-02 |
| SUCNR1 | 56670 | -4.775938 | 0.6265826 | 6.21E-02 | 1.71E-01 |
| CRYGD | 1421 | -4.748279 | 2.4749978 | 1.71E-11 | 9.89E-10 |
| MAP3K7CL | 56911 | -4.671997 | 3.4085556 | 3.79E-21 | 9.29E-19 |
| SPINK5 | 11005 | -4.668648 | 3.9327106 | 1.27E-10 | 6.61E-09 |
| LRRC61 | 65999 | -4.64965 | 1.1697911 | 2.87E-08 | 9.46E-07 |
| C2orf83 | 56918 | -4.645467 | -0.299512 | 2.43E-03 | 1.50E-02 |
| ABCA10 | 10349 | -4.621758 | 0.4743478 | 3.70E-15 | 4.05E-13 |
| THEM6 | 51337 | -4.615087 | 4.2587614 | 1.52E-30 | 8.25E-28 |
| CCR7 | 1236 | -4.613622 | -0.133567 | 9.38E-08 | 2.70E-06 |
| ECM1 | 1893 | -4.602355 | 2.373471 | 8.87E-13 | 6.70E-11 |
| GASK1B | 51313 | -4.547185 | 2.2188757 | 2.00E-20 | 4.34E-18 |
| GPC3 | 2719 | -4.543458 | 2.8789097 | 7.19E-14 | 6.80E-12 |
| NETO1 | 81832 | -4.529576 | 4.9559698 | 2.52E-16 | 3.27E-14 |
| PHOX2B | 8929 | -4.529226 | -0.111526 | 8.95E-03 | 4.16E-02 |
| MEIS1 | 4211 | -4.529107 | 3.8973768 | 1.07E-27 | 4.88E-25 |
| ARHGEF26 | 26084 | -4.506154 | 2.2523865 | 5.55E-09 | 2.15E-07 |
| FGFBP2 | 83888 | -4.494473 | 2.8951799 | 2.95E-03 | 1.75E-02 |
| MPPED1 | 758 | -4.489416 | 2.6639183 | 1.17E-03 | 8.41E-03 |
| MN1 | 4330 | -4.468811 | 4.0209453 | 2.56E-28 | 1.21E-25 |
| NEXN | 91624 | -4.459826 | 2.9069402 | 1.17E-19 | 2.32E-17 |
| CXCL2 | 2920 | -4.455476 | -0.879648 | 1.22E-02 | 5.23E-02 |
| TAC1 | 6863 | -4.449488 | 9.2002863 | 9.15E-05 | 1.05E-03 |
| C15orf48 | 84419 | -4.440458 | 0.386729 | 1.17E-02 | 5.07E-02 |
| P2RY1 | 5028 | -4.379293 | 3.1607641 | 5.34E-21 | 1.24E-18 |
| BCL11B | 64919 | -4.371028 | 4.6836618 | 1.29E-04 | 1.39E-03 |
| SPRY2 | 10253 | -4.347396 | 6.402824 | 1.41E-10 | 7.28E-09 |
| GRM3 | 2913 | -4.339721 | 4.672974 | 1.31E-16 | 1.75E-14 |
| SP140L | 93349 | -4.300338 | 1.2493179 | 6.25E-05 | 7.66E-04 |
| ITGB3 | 3690 | -4.294246 | 0.4400828 | 3.19E-04 | 2.92E-03 |
| WDR49 | 151790 | -4.257771 | 0.5934333 | 2.10E-02 | 7.85E-02 |
| TNFAIP8L3 | 388121 | -4.255511 | 2.0035946 | 1.97E-19 | 3.79E-17 |
| MSTN | 2660 | -4.224158 | 1.3689611 | 3.59E-04 | 3.22E-03 |
| CRHBP | 1393 | -4.224103 | 4.6267212 | 3.82E-46 | 7.21E-43 |
| ECHDC3 | 79746 | -4.185805 | 1.02901 | 1.25E-06 | 2.63E-05 |
| UTS2B | 257313 | -4.185786 | 2.5537403 | 3.07E-07 | 7.66E-06 |
| POU3F2 | 5454 | -4.14836 | 3.5130626 | 1.02E-24 | 3.76E-22 |
| C4orf19 | 55286 | -4.090376 | 1.4939704 | 2.23E-06 | 4.31E-05 |
| ISG15 | 9636 | -4.059221 | 5.8463683 | 1.25E-48 | 2.76E-45 |
| SECTM1 | 6398 | -4.050269 | 1.7163489 | 2.09E-04 | 2.06E-03 |
| EPHA1 | 2041 | -4.047589 | -0.567633 | 2.81E-05 | 3.86E-04 |
| PAX6 | 5080 | -4.016599 | 6.9972735 | 1.76E-23 | 5.83E-21 |
| HLA-DOA | 3111 | -4.009216 | 1.2748572 | 1.10E-01 | 2.56E-01 |
| STAC2 | 342667 | -3.991286 | 1.230083 | 4.12E-05 | 5.28E-04 |
| COL6A2 | 1292 | -3.97333 | 3.2412326 | 1.59E-08 | 5.52E-07 |
| ADAMTS5 | 11096 | -3.968854 | 3.3811897 | 5.90E-34 | 4.34E-31 |
| PTTG1 | 9232 | -3.958415 | 6.8837453 | 2.73E-23 | 8.81E-21 |
| DRD5 | 1816 | -3.93914 | -0.190016 | 9.24E-06 | 1.50E-04 |
| IAPP | 3375 | -3.937207 | 1.1013816 | 5.30E-03 | 2.82E-02 |
| PLXND1 | 23129 | -3.9126 | 0.0290886 | 8.22E-05 | 9.55E-04 |
| BCHE | 590 | -3.881539 | 5.1569593 | 4.26E-53 | 1.13E-49 |
| KLF5 | 688 | -3.847285 | 4.098416 | 1.46E-45 | 2.42E-42 |
| PPP1R1B | 84152 | -3.834824 | 1.8252773 | 7.76E-08 | 2.29E-06 |
| SCARA3 | 51435 | -3.796483 | 1.9301204 | 8.64E-15 | 9.14E-13 |
| C3orf52 | 79669 | -3.789345 | 1.3257029 | 7.63E-05 | 9.04E-04 |
| RNASE1 | 6035 | -3.782159 | 0.1692016 | 3.21E-04 | 2.93E-03 |
| FOXP4 | 116113 | -3.778907 | 2.4333652 | 2.40E-09 | 1.00E-07 |
| ZNF385D | 79750 | -3.775892 | 4.9873435 | 1.96E-07 | 5.10E-06 |
| PACSIN3 | 29763 | -3.734033 | 1.7365342 | 1.39E-08 | 4.94E-07 |
| SYT10 | 341359 | -3.719363 | 3.4459748 | 5.57E-05 | 6.95E-04 |
| CRTAM | 56253 | -3.711668 | 0.9313494 | 2.57E-03 | 1.57E-02 |
| SLC40A1 | 30061 | -3.71083 | 2.7554525 | 4.63E-21 | 1.11E-18 |
| CALCRL | 10203 | -3.707781 | 2.8624863 | 3.46E-23 | 1.06E-20 |
| FOXP1 | 27086 | -3.706102 | 5.4705012 | 1.42E-31 | 8.95E-29 |
| SEMA3C | 10512 | -3.702672 | 4.8016525 | 3.59E-08 | 1.16E-06 |
| RYR2 | 6262 | -3.701269 | 2.9209225 | 6.47E-12 | 4.18E-10 |
| SPHKAP | 80309 | -3.696801 | 3.2904524 | 1.48E-06 | 3.03E-05 |
| RGS13 | 6003 | -3.696277 | 1.9285381 | 9.01E-08 | 2.60E-06 |
| LRMDA | 83938 | -3.6827 | 1.69695 | 1.12E-16 | 1.52E-14 |
| IRX5 | 10265 | -3.638195 | 1.2872055 | 1.54E-07 | 4.15E-06 |
| LY6H | 4062 | -3.608385 | 7.4129185 | 4.78E-32 | 3.16E-29 |
| TSHR | 7253 | -3.603846 | 0.7820561 | 7.13E-07 | 1.60E-05 |
| RHOJ | 57381 | -3.598481 | 2.8171749 | 6.88E-06 | 1.15E-04 |
| PRND | 23627 | -3.558564 | 1.2614175 | 4.34E-04 | 3.77E-03 |
| SAMSN1 | 388813 | -3.553307 | 1.867834 | 1.26E-04 | 1.36E-03 |
| ERMN | 57471 | -3.552861 | -0.413384 | 1.58E-01 | 3.26E-01 |
| MYH14 | 79784 | -3.550891 | 0.2943036 | 6.96E-05 | 8.36E-04 |
| COL19A1 | 1310 | -3.527601 | 2.9512592 | 4.37E-16 | 5.26E-14 |
| RASAL3 | 64926 | -3.527517 | 0.738935 | 7.48E-11 | 4.01E-09 |
| IL12RB2 | 3595 | -3.525955 | 0.2497753 | 4.08E-03 | 2.27E-02 |
| LIX1 | 167410 | -3.518934 | 3.3575266 | 1.33E-05 | 2.04E-04 |
| PRKCD | 5580 | -3.506538 | 3.5879078 | 4.72E-24 | 1.60E-21 |
| KRT15 | 3866 | -3.499964 | 2.5081145 | 4.67E-07 | 1.08E-05 |
| GBX2 | 2637 | -3.497853 | 1.5853575 | 3.46E-08 | 1.12E-06 |
| SLC19A3 | 80704 | -3.491 | 0.5526208 | 9.62E-07 | 2.09E-05 |
| CRB3 | 92359 | -3.489975 | 1.0313328 | 4.86E-04 | 4.13E-03 |
| CLEC11A | 6320 | -3.462812 | 4.9045583 | 3.97E-25 | 1.55E-22 |
| SLITRK6 | 84189 | -3.449005 | 4.7402022 | 1.25E-05 | 1.94E-04 |
| CSGALNACT1 | 55790 | -3.448078 | 3.1504176 | 1.48E-12 | 1.08E-10 |
| TICAM1 | 148022 | -3.444505 | 0.7439354 | 5.70E-11 | 3.12E-09 |
| NBL1 | 100532736 | -3.431577 | 4.682689 | 6.77E-39 | 6.89E-36 |
| F2RL1 | 2150 | -3.426705 | 2.673353 | 1.53E-05 | 2.30E-04 |
| SP110 | 3431 | -3.42148 | 0.5372575 | 1.58E-02 | 6.35E-02 |
| CTXND1 | 100996492 | -3.394967 | 0.0536389 | 4.24E-04 | 3.70E-03 |
| ALK | 238 | -3.370801 | 2.556538 | 3.20E-07 | 7.94E-06 |
| ADAMTS1 | 9510 | -3.36587 | 3.293486 | 1.44E-06 | 2.96E-05 |
| ITM2A | 9452 | -3.365165 | 3.968215 | 1.30E-11 | 7.73E-10 |
| PDE1C | 5137 | -3.358401 | 4.1724653 | 1.17E-04 | 1.28E-03 |
| SKOR1 | 390598 | -3.34702 | -0.708102 | 1.16E-03 | 8.31E-03 |
| EBF1 | 1879 | -3.346654 | 4.7906177 | 6.00E-09 | 2.29E-07 |
| STRIT1 | 100507537 | -3.340033 | 0.3024306 | 2.48E-04 | 2.35E-03 |
| FUOM | 282969 | -3.335416 | 2.8300187 | 1.77E-13 | 1.50E-11 |
| COL8A1 | 1295 | -3.331996 | 2.296951 | 2.43E-05 | 3.39E-04 |
| SOX9 | 6662 | -3.326325 | 3.5168138 | 4.87E-06 | 8.48E-05 |
| TIAM2 | 26230 | -3.325557 | 3.4094549 | 1.53E-10 | 7.83E-09 |
| HFE | 3077 | -3.293033 | 0.1175498 | 2.95E-02 | 1.01E-01 |
| OSTN | 344901 | -3.285922 | 4.8413209 | 9.71E-03 | 4.41E-02 |
| ARPP21 | 10777 | -3.285398 | 6.8237145 | 5.45E-03 | 2.88E-02 |
| GUCY1A1 | 2982 | -3.274354 | 5.9263835 | 3.58E-02 | 1.16E-01 |
| KCNG1 | 3755 | -3.263878 | 3.0188889 | 1.57E-21 | 4.06E-19 |
| CACNG3 | 10368 | -3.258043 | 2.5178869 | 7.91E-03 | 3.81E-02 |
| LY96 | 23643 | -3.239592 | 0.6084138 | 3.11E-03 | 1.82E-02 |
| PALMD | 54873 | -3.238018 | 3.8307889 | 6.56E-22 | 1.74E-19 |
| TAFA4 | 151647 | -3.23436 | 1.4068988 | 1.69E-02 | 6.68E-02 |
| ADARB2 | 105 | -3.230568 | 1.2949767 | 6.26E-03 | 3.19E-02 |
| MT-CO2 | 4513 | -3.229678 | 13.583573 | 4.33E-09 | 1.71E-07 |
| RLN2 | 6019 | -3.226248 | 0.6228064 | 6.11E-04 | 4.96E-03 |
| SHROOM4 | 57477 | -3.225148 | 0.4734975 | 1.63E-05 | 2.43E-04 |
| LMO7 | 4008 | -3.208448 | 2.6668743 | 1.28E-10 | 6.65E-09 |
| FHL2 | 2274 | -3.193634 | 4.4980364 | 1.57E-13 | 1.35E-11 |
| CPT1A | 1374 | -3.18164 | -0.485871 | 6.19E-04 | 5.01E-03 |
| L3MBTL4 | 91133 | -3.178651 | 0.5861226 | 8.93E-06 | 1.45E-04 |
| ADRA1A | 148 | -3.175669 | 1.2920572 | 1.51E-08 | 5.31E-07 |
| GRPR | 2925 | -3.16964 | 2.2909946 | 4.05E-04 | 3.58E-03 |
| DKK1 | 22943 | -3.165909 | 2.4260347 | 1.29E-04 | 1.39E-03 |
| ESRRG | 2104 | -3.155004 | 5.5511259 | 1.29E-07 | 3.56E-06 |
| RBP4 | 5950 | -3.154035 | 5.6366769 | 9.89E-20 | 2.01E-17 |
| MT-CO3 | 4514 | -3.147784 | 13.231375 | 1.15E-07 | 3.22E-06 |
| CD109 | 135228 | -3.143177 | 0.532353 | 6.94E-04 | 5.53E-03 |
| B3GNT9 | 84752 | -3.14261 | 0.6817399 | 8.50E-06 | 1.39E-04 |
| KCNA1 | 3736 | -3.134439 | 3.4983264 | 7.14E-04 | 5.66E-03 |
| IRF6 | 3664 | -3.130417 | 0.2249099 | 2.25E-03 | 1.41E-02 |
| CARTPT | 9607 | -3.129383 | 5.7378288 | 5.95E-02 | 1.66E-01 |
| SYT6 | 148281 | -3.122575 | 5.2845342 | 2.00E-34 | 1.55E-31 |
| RENBP | 5973 | -3.121737 | 0.7193596 | 3.34E-03 | 1.93E-02 |
| NPFFR2 | 10886 | -3.111827 | 2.1646775 | 4.53E-07 | 1.05E-05 |
| C7orf57 | 136288 | -3.099243 | -0.397348 | 3.11E-01 | 5.11E-01 |
| COL16A1 | 1307 | -3.090795 | 0.3651208 | 1.63E-07 | 4.36E-06 |
| CRYM | 1428 | -3.088892 | 3.8920934 | 4.64E-11 | 2.57E-09 |
| IQCA1 | 79781 | -3.082676 | 3.534319 | 3.22E-11 | 1.81E-09 |
| TLL1 | 7092 | -3.066057 | -0.081036 | 3.08E-07 | 7.66E-06 |
| HLA-A | 3105 | -3.059563 | 6.694895 | 2.52E-25 | 1.01E-22 |
| NAALAD2 | 10003 | -3.054844 | 1.6078393 | 4.78E-07 | 1.11E-05 |
| IFI16 | 3428 | -3.043676 | 2.4003564 | 9.66E-05 | 1.10E-03 |
| RELL1 | 768211 | -3.035859 | 2.5503235 | 4.92E-21 | 1.16E-18 |
| FZD7 | 8324 | -3.030829 | 1.3634506 | 2.31E-04 | 2.23E-03 |
| LY6E | 4061 | -3.028572 | 5.3812422 | 8.66E-27 | 3.82E-24 |
| TESMIN | 9633 | -3.023983 | 0.403973 | 1.79E-03 | 1.17E-02 |
| FAM71F1 | 84691 | -3.015715 | 0.957101 | 1.36E-12 | 9.91E-11 |
| CTSC | 1075 | -3.005553 | 4.6649953 | 1.72E-12 | 1.24E-10 |
| BTN3A2 | 11118 | -2.998491 | 2.2571097 | 5.78E-06 | 9.85E-05 |
| TRPC7 | 57113 | -2.996934 | -0.158322 | 1.82E-03 | 1.19E-02 |
| COBLL1 | 22837 | -2.989846 | 1.974012 | 7.72E-06 | 1.28E-04 |
| ERBB4 | 2066 | -2.98711 | 2.6441851 | 2.84E-09 | 1.15E-07 |
| RGS6 | 9628 | -2.984177 | 3.3989514 | 1.46E-22 | 4.12E-20 |
| ACTN2 | 88 | -2.96842 | 2.2577875 | 3.16E-10 | 1.54E-08 |
| ZFP41 | 286128 | -2.966999 | 2.2283316 | 1.42E-16 | 1.88E-14 |
| TSHZ1 | 10194 | -2.96086 | 3.1934325 | 7.97E-07 | 1.78E-05 |
| CFAP74 | 85452 | -2.955479 | 1.4082954 | 5.02E-04 | 4.24E-03 |
| SLC4A4 | 8671 | -2.955332 | 3.247089 | 2.06E-08 | 6.95E-07 |
| CACNA2D3 | 55799 | -2.939581 | 3.3301979 | 2.46E-04 | 2.34E-03 |
| GPR63 | 81491 | -2.936415 | 2.6080946 | 9.06E-09 | 3.33E-07 |
| TFPI2 | 7980 | -2.934572 | 9.017931 | 4.65E-06 | 8.19E-05 |
| GPR26 | 2849 | -2.93443 | 4.10032 | 7.92E-03 | 3.81E-02 |
| LMO4 | 8543 | -2.931072 | 5.6431466 | 5.71E-18 | 9.69E-16 |
| ITGB4 | 3691 | -2.918315 | 0.792882 | 9.41E-03 | 4.31E-02 |
| CCDC88C | 440193 | -2.917238 | 3.6252533 | 4.04E-17 | 6.01E-15 |
| HBQ1 | 3049 | -2.915238 | 3.9321873 | 1.53E-07 | 4.14E-06 |
| PCDH18 | 54510 | -2.909531 | 1.731134 | 3.23E-04 | 2.94E-03 |
| CPLX1 | 10815 | -2.905422 | 2.7793348 | 1.22E-03 | 8.68E-03 |
| FOXA1 | 3169 | -2.89671 | 0.4984799 | 2.50E-03 | 1.53E-02 |
| KCNJ5 | 3762 | -2.895544 | 0.8234983 | 1.67E-08 | 5.77E-07 |
| NAPRT | 93100 | -2.88393 | 2.5836347 | 3.17E-09 | 1.28E-07 |
| NKAIN3 | 286183 | -2.872171 | 5.9485175 | 3.33E-12 | 2.30E-10 |
| SNTG1 | 54212 | -2.869726 | 4.8278519 | 2.65E-03 | 1.61E-02 |
| PLPP4 | 196051 | -2.859566 | 2.0124215 | 3.87E-04 | 3.44E-03 |
| ADAMTS3 | 9508 | -2.848801 | 0.6515744 | 1.74E-04 | 1.77E-03 |
| VEGFC | 7424 | -2.843654 | 0.6138033 | 1.07E-05 | 1.70E-04 |
| GDF15 | 9518 | -2.820503 | 1.8185869 | 9.15E-05 | 1.05E-03 |
| AP1M2 | 10053 | -2.817994 | 2.0690571 | 9.63E-14 | 8.67E-12 |
| PAPPA2 | 60676 | -2.809086 | 1.2266631 | 1.77E-10 | 8.99E-09 |
| TMCC3 | 57458 | -2.80724 | 2.3540644 | 9.62E-18 | 1.57E-15 |
| RELB | 5971 | -2.803677 | 2.7828406 | 3.09E-05 | 4.17E-04 |
| ART5 | 116969 | -2.783838 | 0.3770568 | 1.70E-02 | 6.68E-02 |
| COL3A1 | 1281 | -2.782443 | 1.6565084 | 1.93E-03 | 1.25E-02 |
| MT-CO1 | 4512 | -2.779268 | 12.252623 | 4.17E-12 | 2.82E-10 |
| GABRA1 | 2554 | -2.778741 | 5.9510721 | 2.10E-03 | 1.33E-02 |
| NPAS2 | 4862 | -2.775625 | 3.1055428 | 4.53E-19 | 8.21E-17 |
| PBX3 | 5090 | -2.77209 | 7.4190044 | 2.36E-05 | 3.33E-04 |
| NPY5R | 4889 | -2.766013 | 2.1800509 | 4.60E-02 | 1.39E-01 |
| FOSL2 | 2355 | -2.758335 | 5.2030635 | 6.94E-08 | 2.08E-06 |
| SFRP2 | 6423 | -2.753728 | 0.6640689 | 2.49E-02 | 8.92E-02 |
| CHRM5 | 1133 | -2.743251 | 3.9747159 | 8.81E-03 | 4.11E-02 |
| ARAP2 | 116984 | -2.74023 | 0.5756777 | 5.46E-07 | 1.25E-05 |
| HCN1 | 348980 | -2.739892 | 2.5566903 | 1.99E-08 | 6.73E-07 |
| SH2D3A | 10045 | -2.720379 | 1.6375064 | 1.60E-10 | 8.19E-09 |
| PSMB9 | 5698 | -2.716281 | 1.4570417 | 1.27E-02 | 5.37E-02 |
| EFEMP2 | 30008 | -2.711937 | 3.829917 | 4.02E-08 | 1.29E-06 |
| ACKR3 | 57007 | -2.702482 | 4.537953 | 1.89E-15 | 2.11E-13 |
| BBX | 56987 | -2.696799 | 6.7278406 | 9.45E-14 | 8.56E-12 |
| CAPG | 822 | -2.696527 | 2.2735464 | 3.04E-05 | 4.11E-04 |
| ARHGAP20 | 57569 | -2.681608 | 3.5076765 | 4.12E-05 | 5.28E-04 |
| LRAT | 9227 | -2.673582 | 2.5550705 | 1.13E-04 | 1.24E-03 |
| CAV2 | 858 | -2.666439 | 2.5083881 | 7.10E-04 | 5.64E-03 |
| OPN3 | 23596 | -2.660631 | 3.3099035 | 2.50E-09 | 1.03E-07 |
| FRAS1 | 80144 | -2.652201 | 4.0488845 | 2.43E-08 | 8.12E-07 |
| TENM1 | 10178 | -2.651466 | 5.2063139 | 1.30E-04 | 1.39E-03 |
| ANKUB1 | 389161 | -2.647888 | 0.3126974 | 5.17E-01 | 6.99E-01 |
| ST8SIA5 | 29906 | -2.647152 | 2.3837908 | 9.04E-09 | 3.33E-07 |
| SHANK1 | 50944 | -2.645113 | 1.2060067 | 1.51E-09 | 6.57E-08 |
| PDLIM3 | 27295 | -2.630641 | 3.8704445 | 9.36E-18 | 1.55E-15 |
| DTX3L | 151636 | -2.617926 | -0.242901 | 5.22E-01 | 7.02E-01 |
| MT-ND4 | 4538 | -2.611336 | 12.560149 | 9.55E-08 | 2.72E-06 |
| TFPI | 7035 | -2.610069 | 3.2008475 | 7.44E-05 | 8.87E-04 |
| MT-ATP6 | 4508 | -2.604519 | 11.882768 | 1.78E-06 | 3.55E-05 |
| IL1RAPL1 | 11141 | -2.599431 | 3.7710762 | 7.97E-05 | 9.30E-04 |
| TSPAN9 | 10867 | -2.595557 | 2.5765456 | 6.41E-10 | 2.97E-08 |
| IRX3 | 79191 | -2.595295 | 0.6873167 | 1.92E-03 | 1.24E-02 |
| CHST3 | 9469 | -2.588706 | 2.4039566 | 1.17E-04 | 1.28E-03 |
| THEMIS2 | 9473 | -2.58761 | 1.4352064 | 5.04E-12 | 3.35E-10 |
| KCNA4 | 3739 | -2.585578 | 3.1968962 | 5.51E-03 | 2.90E-02 |
| TMEM125 | 128218 | -2.585489 | 0.0847832 | 8.27E-03 | 3.93E-02 |
| CD163L1 | 283316 | -2.58223 | 0.042862 | 3.97E-04 | 3.51E-03 |
| LINC01597 | 107985433 | -2.578808 | 0.1982536 | 2.84E-06 | 5.30E-05 |
| VCAM1 | 7412 | -2.569062 | 2.2695404 | 5.42E-02 | 1.56E-01 |
| SHISA8 | 440829 | -2.568759 | 0.4751459 | 4.30E-02 | 1.32E-01 |
| CCN1 | 3491 | -2.566567 | 6.5237137 | 8.21E-03 | 3.90E-02 |
| PCDH11X | 27328 | -2.562066 | 5.8996731 | 2.61E-04 | 2.46E-03 |
| JCHAIN | 3512 | -2.559963 | 0.9717022 | 1.29E-07 | 3.56E-06 |
| CX3CL1 | 6376 | -2.555612 | 4.4255578 | 2.41E-20 | 5.07E-18 |
| ADCY8 | 114 | -2.549144 | 2.4461956 | 2.24E-03 | 1.40E-02 |
| ARID5B | 84159 | -2.548903 | 6.7841374 | 1.82E-39 | 2.00E-36 |
| CNMD | 11061 | -2.544132 | -0.148444 | 7.93E-04 | 6.15E-03 |
| LMF1 | 64788 | -2.541424 | 4.3509996 | 2.59E-18 | 4.57E-16 |
| NABP1 | 64859 | -2.535397 | 1.4432933 | 5.08E-06 | 8.77E-05 |
| SERPINI2 | 5276 | -2.53484 | 1.2807552 | 3.25E-03 | 1.89E-02 |
| MT-CYB | 4519 | -2.525914 | 12.638101 | 2.20E-06 | 4.29E-05 |
| NEDD9 | 4739 | -2.511682 | 4.868144 | 1.10E-07 | 3.09E-06 |
| PTER | 9317 | -2.51019 | 1.1707022 | 4.86E-10 | 2.29E-08 |
| ASPM | 259266 | -2.509056 | 1.0398437 | 3.30E-03 | 1.91E-02 |
| SOWAHA | 134548 | -2.506136 | 0.8339185 | 1.37E-02 | 5.71E-02 |
| OSBPL3 | 26031 | -2.50337 | 2.8158183 | 1.23E-03 | 8.74E-03 |
| PARD3B | 117583 | -2.497711 | 2.0530946 | 9.46E-06 | 1.53E-04 |
| YPEL2 | 388403 | -2.497268 | 4.1104383 | 4.73E-22 | 1.28E-19 |
| LRRC8C | 84230 | -2.490547 | 2.1374708 | 5.28E-05 | 6.62E-04 |
| CNN2 | 1265 | -2.48594 | 6.7643052 | 5.89E-17 | 8.38E-15 |
| KCNK12 | 56660 | -2.48001 | 3.8135237 | 3.61E-05 | 4.72E-04 |
| ZNF367 | 195828 | -2.4729 | -0.012359 | 1.68E-04 | 1.72E-03 |
| ADCY7 | 113 | -2.472226 | 0.5213222 | 4.99E-06 | 8.66E-05 |
| IFITM1 | 8519 | -2.47031 | 5.8823392 | 5.03E-09 | 1.96E-07 |
| ITGAV | 3685 | -2.465821 | 3.6145072 | 9.48E-08 | 2.71E-06 |
| BMP2 | 650 | -2.463672 | 2.0643507 | 6.41E-11 | 3.48E-09 |
| GAL | 51083 | -2.461807 | 2.0537782 | 6.40E-05 | 7.80E-04 |
| CLEC2L | 154790 | -2.458202 | 2.1162422 | 8.05E-03 | 3.85E-02 |
| AKAP7 | 9465 | -2.453413 | 4.060249 | 1.04E-13 | 9.21E-12 |
| GAS1 | 2619 | -2.451926 | 1.4403257 | 8.75E-08 | 2.56E-06 |
| PCDH15 | 65217 | -2.44798 | 2.213212 | 6.84E-05 | 8.23E-04 |
| TTN | 7273 | -2.439562 | 3.9320364 | 5.89E-12 | 3.86E-10 |
| EMP1 | 2012 | -2.430624 | 2.0282863 | 5.54E-02 | 1.58E-01 |
| RBFOX1 | 54715 | -2.427452 | 5.6001588 | 3.19E-05 | 4.28E-04 |
| S100A10 | 6281 | -2.425084 | 7.4238669 | 2.45E-13 | 2.03E-11 |
| KCNF1 | 3754 | -2.424068 | 5.3857108 | 1.22E-05 | 1.90E-04 |
| GNG7 | 2788 | -2.421002 | 3.6609891 | 9.88E-09 | 3.60E-07 |
| PLXNA2 | 5362 | -2.413409 | 5.4172592 | 2.60E-13 | 2.14E-11 |
| SCRG1 | 11341 | -2.413337 | 3.3852384 | 4.66E-12 | 3.13E-10 |
| FAT4 | 79633 | -2.411734 | 2.5022994 | 1.17E-06 | 2.48E-05 |
| REPS2 | 9185 | -2.408877 | 2.1712563 | 9.88E-04 | 7.32E-03 |
| FGFR2 | 2263 | -2.408399 | 2.3031452 | 1.36E-05 | 2.08E-04 |
| HEY1 | 23462 | -2.407164 | 3.8844101 | 1.15E-11 | 6.95E-10 |
| EID3 | 493861 | -2.400198 | 1.1621072 | 2.14E-03 | 1.35E-02 |
| GPC6 | 10082 | -2.399468 | 1.8000603 | 1.61E-07 | 4.33E-06 |
| PLAGL1 | 5325 | -2.39815 | 2.883056 | 1.40E-05 | 2.12E-04 |
| KCNA5 | 3741 | -2.397946 | 3.5221128 | 1.21E-03 | 8.60E-03 |
| IL1RAP | 3556 | -2.39662 | 2.4372868 | 9.76E-05 | 1.11E-03 |
| C9orf24 | 84688 | -2.385884 | 2.6482421 | 6.88E-03 | 3.42E-02 |
| FAM241A | 132720 | -2.380952 | 3.3321675 | 5.81E-08 | 1.76E-06 |
| GPR50 | 9248 | -2.379059 | 3.6565868 | 3.20E-09 | 1.28E-07 |
| GRIK1 | 2897 | -2.377707 | 3.8685531 | 6.31E-05 | 7.73E-04 |
| C11orf96 | 387763 | -2.374872 | 0.9719511 | 1.26E-02 | 5.36E-02 |
| RASL11A | 387496 | -2.373062 | 0.0484925 | 5.23E-02 | 1.51E-01 |
| NTF3 | 4908 | -2.370519 | 1.5083828 | 3.92E-03 | 2.20E-02 |
| OLFML3 | 56944 | -2.369244 | 4.7708887 | 2.52E-02 | 8.99E-02 |
| NMI | 9111 | -2.360941 | 1.6377045 | 1.57E-02 | 6.31E-02 |
| SLITRK2 | 84631 | -2.359578 | 1.3193866 | 4.08E-02 | 1.28E-01 |
| PPP1R14A | 94274 | -2.353451 | 0.9788479 | 4.40E-03 | 2.40E-02 |
| BTN2A2 | 10385 | -2.353409 | 3.0862228 | 3.30E-07 | 8.09E-06 |
| HLA-B | 3106 | -2.35283 | 5.7852369 | 7.60E-06 | 1.26E-04 |
| ARC | 23237 | -2.340792 | 2.5222942 | 3.22E-02 | 1.07E-01 |
| MTMR10 | 54893 | -2.330942 | 0.8291671 | 2.04E-04 | 2.02E-03 |
| MLC1 | 23209 | -2.328063 | 4.3514719 | 1.15E-02 | 5.01E-02 |
| NDNF | 79625 | -2.325929 | 0.4229821 | 1.11E-02 | 4.90E-02 |
| SERPINI1 | 5274 | -2.325146 | 6.7687906 | 1.15E-09 | 5.07E-08 |
| PROC | 5624 | -2.324443 | -0.20261 | 6.98E-03 | 3.46E-02 |
| CILP2 | 148113 | -2.320969 | 0.0036794 | 1.38E-02 | 5.73E-02 |
| MAPK13 | 5603 | -2.320834 | 1.0449984 | 3.12E-02 | 1.05E-01 |
| LMO3 | 55885 | -2.314248 | 5.5576807 | 6.59E-03 | 3.31E-02 |
| FIGN | 55137 | -2.30636 | 3.4955312 | 1.35E-11 | 8.00E-10 |
| HTR2C | 3358 | -2.302911 | 2.0889801 | 5.38E-08 | 1.64E-06 |
| OCA2 | 4948 | -2.30181 | 1.5169228 | 3.26E-07 | 8.04E-06 |
| STEAP2 | 261729 | -2.300694 | 1.0968938 | 1.91E-04 | 1.91E-03 |
| IFITM2 | 10581 | -2.297941 | 3.5796844 | 1.07E-09 | 4.75E-08 |
| RFX4 | 5992 | -2.297586 | 2.7983316 | 1.54E-04 | 1.60E-03 |
| MUTYH | 4595 | -2.296096 | -0.116515 | 6.50E-04 | 5.22E-03 |
| MT-ND3 | 4537 | -2.293627 | 7.9830148 | 3.78E-06 | 6.80E-05 |
| UCN | 7349 | -2.288796 | 2.0389968 | 1.88E-04 | 1.89E-03 |
| CXCL14 | 9547 | -2.278127 | 4.278425 | 1.66E-10 | 8.43E-09 |
| N4BP2L1 | 90634 | -2.27524 | 1.8965454 | 9.01E-07 | 1.98E-05 |
| SCN5A | 6331 | -2.26893 | 0.549254 | 8.69E-03 | 4.07E-02 |
| RAB13 | 5872 | -2.267768 | 1.5708121 | 1.98E-03 | 1.27E-02 |
| HOXB2 | 3212 | -2.266236 | 0.0788972 | 2.06E-02 | 7.75E-02 |
| EPHX4 | 253152 | -2.264305 | 3.4843965 | 3.20E-13 | 2.57E-11 |
| DCAF4 | 26094 | -2.26375 | 4.0259466 | 2.72E-13 | 2.22E-11 |
| CDH20 | 28316 | -2.262261 | -0.117561 | 8.21E-03 | 3.90E-02 |
| C19orf81 | 342918 | -2.259448 | 3.1127172 | 5.37E-08 | 1.64E-06 |
| PCSK6 | 5046 | -2.256498 | 1.5913142 | 1.69E-06 | 3.39E-05 |
| RHBDL3 | 162494 | -2.252761 | 1.1896847 | 2.46E-07 | 6.23E-06 |
| SNAI2 | 6591 | -2.250328 | 2.3545133 | 1.68E-07 | 4.46E-06 |
| ZNF503 | 84858 | -2.248454 | 3.5955738 | 1.65E-13 | 1.41E-11 |
| IDUA | 3425 | -2.243482 | 1.1706674 | 1.76E-06 | 3.51E-05 |
| PTGS2 | 5743 | -2.237869 | -0.209748 | 3.23E-04 | 2.94E-03 |
| FRMD4B | 23150 | -2.236703 | 3.5382005 | 5.38E-04 | 4.48E-03 |
| CMTM8 | 152189 | -2.235098 | 1.628379 | 1.33E-07 | 3.66E-06 |
| ALKAL2 | 285016 | -2.234745 | 1.0790492 | 1.78E-03 | 1.17E-02 |
| C8orf88 | 100127983 | -2.230936 | 0.879484 | 6.49E-03 | 3.28E-02 |
| FN1 | 2335 | -2.230716 | 3.7592453 | 2.64E-05 | 3.64E-04 |
| TFEB | 7942 | -2.226644 | 1.363259 | 1.87E-03 | 1.21E-02 |
| C1QTNF2 | 114898 | -2.222461 | 0.6741366 | 4.70E-02 | 1.41E-01 |
| MAML2 | 84441 | -2.221328 | 1.5275156 | 1.50E-08 | 5.30E-07 |
| RASL11B | 65997 | -2.219296 | 0.1080057 | 1.12E-03 | 8.09E-03 |
| SMAD9 | 4093 | -2.211282 | 3.3640731 | 9.78E-15 | 1.03E-12 |
| FRMPD4 | 9758 | -2.207603 | 1.538824 | 3.40E-07 | 8.29E-06 |
| ANKRD30BL | 554226 | -2.203113 | 0.6812905 | 1.05E-04 | 1.17E-03 |
| EMP2 | 2013 | -2.202168 | 3.6272548 | 4.06E-07 | 9.68E-06 |
| DCBLD2 | 131566 | -2.197715 | 5.8347939 | 1.47E-09 | 6.41E-08 |
| BCL11A | 53335 | -2.192621 | 5.9058482 | 1.91E-03 | 1.24E-02 |
| MYL4 | 4635 | -2.190562 | 0.6124057 | 6.40E-05 | 7.80E-04 |
| CHST2 | 9435 | -2.189893 | 5.9458988 | 1.12E-19 | 2.24E-17 |
| EIF4A1 | 1973 | -2.187753 | 0.9919123 | 6.29E-06 | 1.06E-04 |
| GNG11 | 2791 | -2.187169 | 8.9297926 | 2.25E-14 | 2.26E-12 |
| SYTL5 | 94122 | -2.186358 | 0.8798563 | 2.70E-03 | 1.63E-02 |
| ABCC6 | 368 | -2.184391 | 0.2923831 | 2.29E-02 | 8.38E-02 |
| EPHB6 | 2051 | -2.176142 | 2.9958792 | 7.22E-11 | 3.88E-09 |
| SLC16A6 | 9120 | -2.169999 | 2.0554254 | 2.30E-02 | 8.39E-02 |
| EPHA6 | 285220 | -2.166827 | 1.8593869 | 8.40E-08 | 2.46E-06 |
| LYPD1 | 116372 | -2.164657 | 4.2439083 | 6.80E-15 | 7.26E-13 |
| DGAT2 | 84649 | -2.164393 | 2.6585393 | 1.29E-03 | 9.07E-03 |
| GABRA4 | 2557 | -2.161192 | 3.8857565 | 5.87E-02 | 1.65E-01 |
| OPRK1 | 4986 | -2.160377 | 1.6666958 | 1.28E-08 | 4.58E-07 |
| HTR7 | 3363 | -2.159607 | 2.0434258 | 2.28E-03 | 1.42E-02 |
| ANKRD18A | 253650 | -2.157591 | 1.1644725 | 2.24E-09 | 9.40E-08 |
| REL | 5966 | -2.15577 | 3.4973591 | 3.21E-13 | 2.57E-11 |
| ARHGAP10 | 79658 | -2.153951 | 1.1215685 | 2.39E-07 | 6.09E-06 |
| LRP5 | 4041 | -2.14667 | 1.0503854 | 7.81E-05 | 9.19E-04 |
| ADAMTS18 | 170692 | -2.146253 | 0.0374605 | 1.53E-03 | 1.03E-02 |
| FAM181B | 220382 | -2.144507 | 0.238308 | 2.57E-02 | 9.12E-02 |
| DPP7 | 29952 | -2.143336 | 4.6349984 | 2.84E-23 | 8.96E-21 |
| TRIM59 | 286827 | -2.141246 | 0.0331431 | 2.47E-02 | 8.85E-02 |
| MMP1 | 4312 | -2.136421 | 3.2199366 | 2.03E-02 | 7.66E-02 |
| RSPO3 | 84870 | -2.135548 | 1.3123991 | 1.35E-02 | 5.65E-02 |
| FBXL7 | 23194 | -2.134413 | 0.8565219 | 1.78E-02 | 6.94E-02 |
| LPIN3 | 64900 | -2.134127 | 0.0056171 | 2.89E-03 | 1.72E-02 |
| IGFBP2 | 3485 | -2.132189 | 8.49075 | 3.56E-07 | 8.66E-06 |
| CACNG5 | 27091 | -2.132066 | 1.5429437 | 8.34E-06 | 1.37E-04 |
| NR4A3 | 8013 | -2.129244 | 4.552713 | 4.46E-16 | 5.31E-14 |
| TMSB4X | 7114 | -2.128487 | 14.368035 | 4.12E-07 | 9.80E-06 |
| GUCY1A2 | 2977 | -2.127402 | 4.1907325 | 1.85E-14 | 1.89E-12 |
| GLIPR1 | 11010 | -2.125422 | 4.8817033 | 2.86E-03 | 1.71E-02 |
| OTP | 23440 | -2.123829 | 1.806659 | 5.36E-02 | 1.54E-01 |
| PLCXD3 | 345557 | -2.12179 | 4.2876608 | 5.20E-03 | 2.77E-02 |
| PILRB | 29990 | -2.117014 | 0.6019721 | 4.69E-02 | 1.41E-01 |
| CCND2 | 894 | -2.109972 | 5.4251305 | 5.48E-07 | 1.25E-05 |
| HLA-F | 3134 | -2.10911 | -0.04829 | 2.26E-03 | 1.41E-02 |
| MYO1F | 4542 | -2.108368 | 0.6751738 | 7.65E-04 | 5.98E-03 |
| DAAM2 | 23500 | -2.108334 | 0.9028531 | 6.16E-06 | 1.04E-04 |
| PLA2G4A | 5321 | -2.106782 | 0.3463658 | 5.12E-04 | 4.30E-03 |
| SERTM1 | 400120 | -2.105163 | 4.6533127 | 4.79E-06 | 8.36E-05 |
| NMBR | 4829 | -2.103987 | 1.2278965 | 9.26E-02 | 2.26E-01 |
| CSRP2 | 1466 | -2.103807 | 8.0511283 | 3.52E-23 | 1.06E-20 |
| FAT3 | 120114 | -2.09876 | 4.413388 | 3.78E-10 | 1.82E-08 |
| MSI2 | 124540 | -2.098428 | 4.2868267 | 5.27E-17 | 7.66E-15 |
| EGR4 | 1961 | -2.093578 | 1.9092392 | 4.95E-02 | 1.46E-01 |
| CNTNAP4 | 85445 | -2.089705 | 2.4494288 | 9.27E-04 | 6.96E-03 |
| RGS20 | 8601 | -2.08725 | 2.6695303 | 2.82E-09 | 1.15E-07 |
| NUDT1 | 4521 | -2.083515 | 5.4458621 | 9.91E-05 | 1.12E-03 |
| DUSP23 | 54935 | -2.078631 | 5.1762127 | 2.51E-17 | 3.91E-15 |
| ASB2 | 51676 | -2.078287 | 0.452961 | 4.12E-03 | 2.28E-02 |
| AARD | 441376 | -2.078086 | 3.4177528 | 5.57E-04 | 4.61E-03 |
| HAPLN3 | 145864 | -2.071759 | 0.5938084 | 2.36E-04 | 2.25E-03 |
| B3GLCT | 145173 | -2.068906 | 4.6037128 | 1.29E-10 | 6.65E-09 |
| PLK2 | 10769 | -2.06538 | 7.3475201 | 5.29E-07 | 1.21E-05 |
| BARD1 | 580 | -2.061573 | 2.3025763 | 1.17E-06 | 2.50E-05 |
| CACNG2 | 10369 | -2.055516 | 2.431445 | 2.91E-03 | 1.73E-02 |
| CASKIN1 | 57524 | -2.055072 | 1.1343638 | 1.83E-04 | 1.84E-03 |
| MTFP1 | 51537 | -2.053813 | 0.7169658 | 8.19E-03 | 3.90E-02 |
| RNASEH2B | 79621 | -2.053235 | 5.0063848 | 4.51E-07 | 1.05E-05 |
| HPGD | 3248 | -2.052101 | 3.4847235 | 1.27E-02 | 5.40E-02 |
| EDNRB | 1910 | -2.044959 | 2.5565948 | 1.85E-03 | 1.20E-02 |
| CPS1 | 1373 | -2.043712 | 0.2914164 | 6.50E-03 | 3.28E-02 |
| MT-ATP8 | 4509 | -2.038148 | 5.9487189 | 7.07E-05 | 8.47E-04 |
| NGEF | 25791 | -2.031724 | 4.1701883 | 1.86E-06 | 3.68E-05 |
| CES5A | 221223 | -2.031602 | 0.1646873 | 9.28E-02 | 2.26E-01 |
| MYLIP | 29116 | -2.030857 | 3.9807872 | 1.98E-12 | 1.41E-10 |
| SPOCD1 | 90853 | -2.015161 | 0.1292398 | 1.45E-01 | 3.08E-01 |
| SPDYE2 | 441273 | -2.014799 | 0.1688432 | 2.24E-02 | 8.25E-02 |
| CALN1 | 83698 | -2.010954 | 3.7514577 | 5.39E-03 | 2.85E-02 |
| PTPRD | 5789 | -2.010831 | 5.1380155 | 5.01E-14 | 4.81E-12 |
| TRHDE | 29953 | -2.010503 | 2.8316469 | 2.07E-06 | 4.06E-05 |
| VIPR1 | 7433 | -2.010138 | -0.170535 | 2.61E-03 | 1.59E-02 |
| LLGL1 | 3996 | -2.009591 | 0.529216 | 5.70E-04 | 4.70E-03 |
| MMP19 | 4327 | -2.009323 | 1.9321639 | 1.00E-01 | 2.39E-01 |
| MAP4K1 | 11184 | -2.00569 | 3.1415654 | 6.29E-02 | 1.73E-01 |
| CDCA7L | 55536 | -2.005385 | 2.1440361 | 2.69E-06 | 5.04E-05 |
| SYT3 | 84258 | -2.005012 | 4.9993308 | 5.65E-06 | 9.65E-05 |
| RELN | 5649 | -2.004725 | 6.0142485 | 1.97E-03 | 1.27E-02 |
| FBF1 | 85302 | -2.004433 | 2.1819964 | 3.47E-06 | 6.28E-05 |
| CNTNAP2 | 26047 | -2.003573 | 9.1803489 | 5.02E-13 | 3.95E-11 |
| WIPF3 | 644150 | -1.999756 | 4.0759066 | 7.99E-03 | 3.83E-02 |
| SYNDIG1 | 79953 | -1.995022 | 4.2469666 | 4.04E-06 | 7.20E-05 |
| PCDHGB4 | 8641 | -1.993401 | 2.9807865 | 1.21E-07 | 3.38E-06 |
| CORT | 1325 | -1.990763 | 0.2397905 | 3.02E-02 | 1.02E-01 |
| UNC93B1 | 81622 | -1.9881 | 3.1006539 | 2.14E-03 | 1.35E-02 |
| UST | 10090 | -1.987515 | 2.3718517 | 1.27E-06 | 2.68E-05 |
| WNT7A | 7476 | -1.986281 | 4.2917023 | 1.44E-17 | 2.30E-15 |
| TXNIP | 10628 | -1.985517 | 4.1997512 | 3.40E-12 | 2.33E-10 |
| INF2 | 64423 | -1.982087 | 2.4595295 | 4.67E-04 | 4.00E-03 |
| WSCD1 | 23302 | -1.979888 | 4.2728968 | 1.72E-06 | 3.45E-05 |
| SHCBP1 | 79801 | -1.979581 | 0.664067 | 1.59E-01 | 3.27E-01 |
| IFIT3 | 3437 | -1.976383 | 2.2401097 | 2.06E-04 | 2.03E-03 |
| TNNI3 | 7137 | -1.975061 | 2.2717584 | 9.46E-02 | 2.29E-01 |
| ELOVL7 | 79993 | -1.972043 | 1.4282152 | 5.86E-05 | 7.26E-04 |
| CHRM3 | 1131 | -1.971399 | 3.7179679 | 1.76E-12 | 1.26E-10 |
| GPR88 | 54112 | -1.970519 | 2.5234976 | 7.86E-02 | 2.02E-01 |
| AGT | 183 | -1.968157 | 1.24647 | 1.10E-03 | 7.97E-03 |
| GAD2 | 2572 | -1.968071 | 6.3785802 | 1.08E-01 | 2.53E-01 |
| NEUROD6 | 63974 | -1.964833 | 3.731035 | 1.43E-01 | 3.05E-01 |
| TMEM121 | 80757 | -1.964211 | 3.9361484 | 2.78E-10 | 1.37E-08 |
| EPCAM | 4072 | -1.964087 | 6.1592455 | 2.40E-02 | 8.66E-02 |
| NENF | 29937 | -1.962483 | 7.1310551 | 3.87E-16 | 4.69E-14 |
| SPON1 | 10418 | -1.953907 | 2.2150634 | 2.46E-06 | 4.68E-05 |
| ATP2B2 | 491 | -1.953572 | 4.1495466 | 9.82E-02 | 2.36E-01 |
| SLC22A18 | 5002 | -1.951964 | 3.5411914 | 4.69E-14 | 4.53E-12 |
| FMNL3 | 91010 | -1.951463 | 0.9199312 | 2.53E-08 | 8.43E-07 |
| S1PR1 | 1901 | -1.949327 | 1.8025664 | 5.67E-02 | 1.61E-01 |
| MCTP1 | 79772 | -1.947212 | 3.6822152 | 6.77E-05 | 8.15E-04 |
| RASGRP2 | 10235 | -1.94671 | 4.8333033 | 1.40E-01 | 3.02E-01 |
| SERTAD4 | 56256 | -1.942845 | 4.6509676 | 2.91E-03 | 1.73E-02 |
| CLDND2 | 125875 | -1.942633 | 0.3161176 | 8.81E-05 | 1.02E-03 |
| MSANTD1 | 345222 | -1.940716 | 4.5795422 | 4.21E-02 | 1.30E-01 |
| C3orf33 | 285315 | -1.940157 | 1.4499486 | 4.47E-06 | 7.91E-05 |
| SEMA3A | 10371 | -1.93914 | 5.1139896 | 6.54E-13 | 5.00E-11 |
| STAG3L3 | 378108 | -1.939132 | 1.7477892 | 2.93E-07 | 7.34E-06 |
| KCNMB2 | 10242 | -1.93557 | 1.7052603 | 8.10E-02 | 2.06E-01 |
| PRKG2 | 5593 | -1.932211 | -0.042152 | 7.80E-04 | 6.06E-03 |
| CHSY3 | 337876 | -1.930691 | 0.7100093 | 1.75E-02 | 6.85E-02 |
| EGR3 | 1960 | -1.930603 | 1.1805338 | 4.03E-03 | 2.24E-02 |
| TTYH1 | 57348 | -1.928743 | 6.9355228 | 9.57E-10 | 4.29E-08 |
| B3GNT2 | 10678 | -1.925859 | 3.8917539 | 9.18E-09 | 3.36E-07 |
| TRPC5 | 7224 | -1.922373 | 2.1614948 | 2.71E-02 | 9.44E-02 |
| SORCS1 | 114815 | -1.912548 | 4.1991673 | 1.98E-08 | 6.73E-07 |
| ZCCHC24 | 219654 | -1.911644 | 1.368853 | 1.08E-01 | 2.53E-01 |
| AARSD1 | 80755 | -1.911433 | 0.0345103 | 2.23E-02 | 8.23E-02 |
| IFITM3 | 10410 | -1.906652 | 8.4991009 | 3.41E-06 | 6.23E-05 |
| SPATS2L | 26010 | -1.904902 | 6.2050498 | 3.52E-19 | 6.47E-17 |
| SPHK1 | 8877 | -1.904894 | 4.7923753 | 6.19E-02 | 1.71E-01 |
| PCDHB12 | 56124 | -1.904122 | 1.0857156 | 4.79E-04 | 4.08E-03 |
| GRTP1 | 79774 | -1.903363 | 1.53922 | 1.42E-02 | 5.86E-02 |
| STAT4 | 6775 | -1.899095 | 1.8576815 | 8.04E-02 | 2.05E-01 |
| PDE4A | 5141 | -1.898388 | 3.4176691 | 5.63E-03 | 2.95E-02 |
| CRLF1 | 9244 | -1.898324 | 3.0634911 | 8.36E-04 | 6.41E-03 |
| PRICKLE1 | 144165 | -1.894794 | 3.6480982 | 1.10E-15 | 1.26E-13 |
| SLCO1B1 | 10599 | -1.893208 | -0.495521 | 3.80E-02 | 1.21E-01 |
| SLITRK5 | 26050 | -1.892434 | 3.7072342 | 2.01E-05 | 2.91E-04 |
| GABRB2 | 2561 | -1.890277 | 5.7346921 | 1.05E-01 | 2.48E-01 |
| PPARGC1A | 10891 | -1.890022 | 4.0171071 | 2.54E-16 | 3.27E-14 |
| IL11 | 3589 | -1.889016 | 4.1282478 | 1.06E-01 | 2.49E-01 |
| NTN1 | 9423 | -1.887715 | 2.9427913 | 6.58E-09 | 2.48E-07 |
| PCDH10 | 57575 | -1.887011 | 5.987959 | 2.48E-04 | 2.36E-03 |
| TENT5A | 55603 | -1.886968 | 4.9839281 | 3.31E-12 | 2.29E-10 |
| KANK3 | 256949 | -1.886512 | 2.4280843 | 1.67E-03 | 1.11E-02 |
| SLC26A8 | 116369 | -1.883539 | -0.161132 | 2.26E-02 | 8.30E-02 |
| C17orf50 | 146853 | -1.881331 | 1.9195771 | 8.21E-02 | 2.08E-01 |
| SLC66A1L | 152078 | -1.881248 | 0.4588579 | 2.36E-04 | 2.26E-03 |
| INSYN2A | 642938 | -1.875538 | 4.1870402 | 2.00E-06 | 3.94E-05 |
| TSHZ2 | 128553 | -1.874834 | 4.0021598 | 1.33E-01 | 2.91E-01 |
| GAS2L1 | 10634 | -1.873857 | 3.5755739 | 2.65E-03 | 1.61E-02 |
| TCEA3 | 6920 | -1.872313 | 1.4374086 | 3.44E-03 | 1.98E-02 |
| USP2 | 9099 | -1.8723 | 0.7825346 | 1.00E-03 | 7.40E-03 |
| HTR1B | 3351 | -1.870134 | -0.224636 | 1.78E-03 | 1.17E-02 |
| EPPK1 | 83481 | -1.868741 | 1.9149972 | 1.05E-04 | 1.17E-03 |
| RBM43 | 375287 | -1.868515 | -0.308689 | 1.44E-02 | 5.92E-02 |
| HRH1 | 3269 | -1.859277 | 2.231544 | 7.99E-07 | 1.78E-05 |
| DLGAP2 | 9228 | -1.854582 | 1.7781643 | 6.05E-02 | 1.68E-01 |
| PDE8B | 8622 | -1.852656 | 1.8036492 | 3.93E-08 | 1.26E-06 |
| CFD | 1675 | -1.852497 | 2.0653773 | 2.51E-02 | 8.96E-02 |
| CDH9 | 1007 | -1.85102 | 3.3788694 | 4.49E-02 | 1.36E-01 |
| PCYT1B | 9468 | -1.846452 | 4.2627341 | 2.36E-20 | 5.05E-18 |
| CSRNP1 | 64651 | -1.843711 | 3.8391095 | 8.48E-05 | 9.83E-04 |
| GNA11 | 2767 | -1.842252 | 5.7625697 | 9.41E-19 | 1.68E-16 |
| THRB | 7068 | -1.839428 | 0.7914135 | 2.74E-04 | 2.57E-03 |
| SFXN2 | 118980 | -1.83454 | 1.3677153 | 1.06E-05 | 1.68E-04 |
| TRIM14 | 9830 | -1.832281 | 0.8751785 | 3.94E-03 | 2.20E-02 |
| CCDC152 | 100129792 | -1.830018 | 3.6424752 | 3.78E-08 | 1.22E-06 |
| H4C11 | 8363 | -1.82957 | 0.4953061 | 3.87E-03 | 2.18E-02 |
| ECHDC2 | 55268 | -1.826385 | 1.3203432 | 1.62E-04 | 1.67E-03 |
| DNASE2B | 58511 | -1.826371 | 0.581196 | 1.90E-02 | 7.30E-02 |
| LGALS1 | 3956 | -1.822656 | 5.0624959 | 2.23E-05 | 3.17E-04 |
| CNTN3 | 5067 | -1.821891 | 2.0468349 | 7.01E-03 | 3.47E-02 |
| BIRC3 | 330 | -1.821809 | 0.5876721 | 6.35E-02 | 1.74E-01 |
| FIBIN | 387758 | -1.82046 | 1.2782856 | 6.01E-03 | 3.10E-02 |
| ZC3H7B | 23264 | -1.817628 | 2.4066063 | 8.99E-08 | 2.60E-06 |
| FCSK | 197258 | -1.81698 | 1.8107543 | 6.92E-05 | 8.33E-04 |
| SCRT2 | 85508 | -1.816293 | 1.7818334 | 9.35E-02 | 2.28E-01 |
| VGF | 7425 | -1.812099 | 6.3574649 | 5.09E-04 | 4.28E-03 |
| PTPRZ1 | 5803 | -1.809383 | 5.5083273 | 4.98E-08 | 1.54E-06 |
| ABCA5 | 23461 | -1.807858 | 5.0124494 | 8.30E-06 | 1.36E-04 |
| JPH1 | 56704 | -1.807836 | 0.3627894 | 6.65E-04 | 5.33E-03 |
| OSBPL10 | 114884 | -1.806768 | 1.4782107 | 1.66E-04 | 1.70E-03 |
| PMEPA1 | 56937 | -1.805488 | 4.153781 | 1.18E-05 | 1.84E-04 |
| ANXA11 | 311 | -1.804003 | 4.5693873 | 1.16E-08 | 4.19E-07 |
| PDZRN3 | 23024 | -1.80274 | 3.0676795 | 9.59E-04 | 7.16E-03 |
| CAV1 | 857 | -1.799777 | 3.5530065 | 3.81E-02 | 1.21E-01 |
| IL10RA | 3587 | -1.797859 | 1.6576012 | 6.46E-03 | 3.27E-02 |
| LSAMP | 4045 | -1.794537 | 6.3791321 | 5.21E-07 | 1.20E-05 |
| HSPA2 | 3306 | -1.79403 | 2.8608666 | 2.99E-04 | 2.77E-03 |
| QRFPR | 84109 | -1.792578 | 0.6362031 | 2.65E-05 | 3.65E-04 |
| SV2C | 22987 | -1.791296 | 4.341885 | 1.78E-11 | 1.02E-09 |
| COBL | 23242 | -1.791295 | 0.0916225 | 1.26E-01 | 2.80E-01 |
| INAFM1 | 255783 | -1.786604 | 6.497208 | 1.65E-07 | 4.39E-06 |
| TACC3 | 10460 | -1.784971 | 1.8232323 | 2.23E-05 | 3.17E-04 |
| SEMA6B | 10501 | -1.782668 | 3.9554217 | 9.53E-03 | 4.35E-02 |
| JCAD | 57608 | -1.781857 | 0.9354265 | 9.13E-03 | 4.22E-02 |
| TTC9B | 148014 | -1.780889 | 7.0457119 | 6.97E-02 | 1.86E-01 |
| RDH12 | 145226 | -1.780772 | 1.091977 | 7.02E-02 | 1.87E-01 |
| ANO8 | 57719 | -1.779974 | 1.1877752 | 6.25E-03 | 3.19E-02 |
| PKDCC | 91461 | -1.773465 | -0.035274 | 3.96E-03 | 2.21E-02 |
| ADCY1 | 107 | -1.773247 | 6.0248364 | 7.91E-03 | 3.81E-02 |
| SNORC | 389084 | -1.771229 | 2.7339712 | 1.05E-01 | 2.48E-01 |
| BBOX1 | 8424 | -1.767906 | 0.2657696 | 3.66E-02 | 1.18E-01 |
| GBP2 | 2634 | -1.764852 | 1.1206997 | 4.07E-02 | 1.27E-01 |
| TMEM132E | 124842 | -1.764816 | 0.9602485 | 2.24E-07 | 5.75E-06 |
| UTRN | 7402 | -1.763987 | 3.4578704 | 4.26E-08 | 1.35E-06 |
| TDRD9 | 122402 | -1.763467 | 2.5858492 | 3.76E-06 | 6.77E-05 |
| COL14A1 | 7373 | -1.763262 | 0.5493321 | 1.77E-01 | 3.53E-01 |
| VCAN | 1462 | -1.761544 | 6.1349835 | 2.90E-15 | 3.20E-13 |
| SRPX | 8406 | -1.760578 | 2.4414205 | 3.83E-04 | 3.41E-03 |
| LATS2 | 26524 | -1.759008 | 2.092147 | 1.07E-03 | 7.83E-03 |
| MRM1 | 79922 | -1.757201 | 1.4179099 | 4.16E-04 | 3.66E-03 |
| SULT2B1 | 6820 | -1.756701 | 0.4143412 | 4.65E-02 | 1.40E-01 |
| FAM120C | 54954 | -1.754249 | 0.6476609 | 7.74E-04 | 6.03E-03 |
| PCBP3 | 54039 | -1.753439 | 3.2858094 | 5.30E-13 | 4.13E-11 |
| CYP2D6 | 107987479 | -1.749713 | 0.0235182 | 4.63E-02 | 1.39E-01 |
| RGN | 9104 | -1.749262 | 0.0112284 | 1.47E-01 | 3.10E-01 |
| GFPT2 | 9945 | -1.748571 | 4.2492131 | 4.52E-04 | 3.90E-03 |
| GABRD | 2563 | -1.748462 | 1.8602149 | 2.15E-04 | 2.11E-03 |
| ZNF804A | 91752 | -1.748378 | 5.3463689 | 8.14E-02 | 2.07E-01 |
| FGFR3 | 2261 | -1.747514 | 0.8237317 | 1.40E-01 | 3.01E-01 |
| IL1R1 | 3554 | -1.747048 | 1.5744535 | 5.28E-02 | 1.53E-01 |
| CNTNAP3B | 728577 | -1.746689 | 1.4320293 | 1.38E-02 | 5.74E-02 |
| SOX21 | 11166 | -1.745358 | -0.536048 | 1.46E-01 | 3.09E-01 |
| CNIH3 | 149111 | -1.745207 | 4.1535291 | 1.91E-07 | 4.98E-06 |
| NELL1 | 4745 | -1.743814 | 4.5533703 | 3.31E-04 | 3.00E-03 |
| KCNH1 | 3756 | -1.742415 | -0.452442 | 1.74E-02 | 6.83E-02 |
| ITPRIPL1 | 150771 | -1.742334 | -0.03272 | 1.25E-01 | 2.79E-01 |
| VSNL1 | 7447 | -1.740147 | 8.7278402 | 7.69E-02 | 1.99E-01 |
| CHRDL1 | 91851 | -1.738351 | 2.6011774 | 2.12E-03 | 1.34E-02 |
| EPHA5 | 2044 | -1.734258 | 4.4077718 | 1.06E-03 | 7.75E-03 |
| FEZF1 | 389549 | -1.731887 | 4.401824 | 9.45E-14 | 8.56E-12 |
| SHH | 6469 | -1.730431 | 0.0532734 | 4.73E-02 | 1.41E-01 |
| TTR | 7276 | -1.728183 | 0.9142581 | 3.49E-03 | 2.00E-02 |
| GPER1 | 2852 | -1.727429 | 1.1030341 | 1.42E-01 | 3.05E-01 |
| CLCF1 | 23529 | -1.723915 | 1.4238881 | 9.48E-02 | 2.30E-01 |
| COL1A2 | 1278 | -1.723083 | 5.7738724 | 4.36E-04 | 3.78E-03 |
| RGS8 | 85397 | -1.721211 | 2.1116689 | 1.46E-01 | 3.09E-01 |
| CEBPD | 1052 | -1.719474 | 3.7588025 | 1.34E-02 | 5.61E-02 |
| SEMA3E | 9723 | -1.719197 | 2.9228137 | 1.29E-08 | 4.61E-07 |
| SAT1 | 6303 | -1.717275 | 7.9838715 | 1.07E-05 | 1.70E-04 |
| H2AJ | 55766 | -1.7153 | 4.2594708 | 4.00E-14 | 3.89E-12 |
| ARHGEF6 | 9459 | -1.713909 | 0.7464818 | 5.10E-03 | 2.73E-02 |
| HS3ST1 | 9957 | -1.713342 | 5.1907598 | 1.08E-13 | 9.51E-12 |
| MT-ND4L | 4539 | -1.711432 | 8.6895663 | 1.74E-04 | 1.77E-03 |
| AHR | 196 | -1.711218 | 1.2348647 | 5.47E-02 | 1.57E-01 |
| DDX58 | 23586 | -1.70861 | -0.341244 | 1.88E-01 | 3.68E-01 |
| HRH2 | 3274 | -1.708243 | 3.8800182 | 2.60E-02 | 9.18E-02 |
| GLDN | 342035 | -1.705134 | -0.137288 | 6.56E-02 | 1.77E-01 |
| ST6GALNAC5 | 81849 | -1.702563 | 4.6162495 | 5.10E-10 | 2.39E-08 |
| RASA4 | 10156 | -1.701756 | 0.0487386 | 7.12E-02 | 1.89E-01 |
| FBXO17 | 115290 | -1.701606 | 2.3975365 | 5.08E-08 | 1.56E-06 |
| DOCK1 | 1793 | -1.69675 | 2.673344 | 6.33E-03 | 3.22E-02 |
| DGCR8 | 54487 | -1.695406 | 2.0439152 | 7.35E-04 | 5.78E-03 |
| FMN1 | 342184 | -1.692269 | 0.5963889 | 6.91E-02 | 1.85E-01 |
| PDGFA | 5154 | -1.689325 | 2.0566322 | 6.73E-05 | 8.13E-04 |
| MCRIP2 | 84331 | -1.688125 | 5.9221879 | 1.57E-14 | 1.62E-12 |
| PDE1B | 5153 | -1.687929 | 3.4763535 | 1.60E-02 | 6.41E-02 |
| CHCHD10 | 400916 | -1.687654 | 1.543166 | 1.68E-03 | 1.12E-02 |
| METRNL | 284207 | -1.687127 | 2.9852205 | 5.14E-05 | 6.45E-04 |
| TOX | 9760 | -1.686441 | 4.6376029 | 1.71E-08 | 5.89E-07 |
| EDNRA | 1909 | -1.684793 | 0.3179726 | 5.01E-02 | 1.46E-01 |
| ARHGEF37 | 389337 | -1.684524 | 1.74397 | 1.03E-05 | 1.65E-04 |
| KIAA1217 | 56243 | -1.682869 | 1.1669857 | 8.21E-03 | 3.90E-02 |
| NRP2 | 8828 | -1.682565 | 6.9014938 | 2.80E-07 | 7.04E-06 |
| TUBA4A | 7277 | -1.682357 | 5.2932324 | 1.11E-20 | 2.44E-18 |
| TACR1 | 6869 | -1.680267 | 0.8401492 | 1.37E-01 | 2.97E-01 |
| SMO | 6608 | -1.679557 | 0.70489 | 3.91E-02 | 1.24E-01 |
| ODAPH | 152816 | -1.679262 | 0.4738628 | 1.62E-01 | 3.33E-01 |
| KCNQ5 | 56479 | -1.677086 | 0.9530512 | 5.82E-02 | 1.64E-01 |
| CSF1 | 1435 | -1.67079 | 3.1159207 | 8.90E-03 | 4.15E-02 |
| FRMPD3 | 84443 | -1.669576 | 0.5859644 | 1.28E-02 | 5.41E-02 |
| GRM7 | 2917 | -1.669491 | 5.5336505 | 1.28E-02 | 5.42E-02 |
| JADE2 | 23338 | -1.668609 | 2.2405707 | 3.33E-04 | 3.01E-03 |
| SPR | 6697 | -1.668257 | 2.1826069 | 4.26E-03 | 2.35E-02 |
| PCOLCE2 | 26577 | -1.66645 | 2.7636992 | 5.10E-05 | 6.41E-04 |
| SORL1 | 6653 | -1.663342 | 2.826311 | 7.96E-05 | 9.30E-04 |
| SFRP4 | 6424 | -1.662276 | 2.9182109 | 3.64E-01 | 5.63E-01 |
| PDK4 | 5166 | -1.661252 | 0.5134719 | 1.61E-01 | 3.31E-01 |
| KRT25 | 147183 | -1.658607 | -0.036816 | 9.90E-03 | 4.48E-02 |
| SIPA1L1 | 26037 | -1.657537 | 3.4597971 | 1.23E-04 | 1.34E-03 |
| NAT8L | 339983 | -1.657387 | 2.5981105 | 3.62E-05 | 4.73E-04 |
| MINAR1 | 23251 | -1.656057 | 2.0455591 | 1.69E-04 | 1.72E-03 |
| TFAP2B | 7021 | -1.655318 | 3.14496 | 1.43E-01 | 3.05E-01 |
| SPX | 80763 | -1.655128 | 0.3337781 | 1.61E-04 | 1.66E-03 |
| LYPD6B | 130576 | -1.652947 | 1.9582505 | 1.90E-03 | 1.24E-02 |
| SLN | 6588 | -1.650486 | -0.148643 | 1.26E-01 | 2.81E-01 |
| VSTM2A | 222008 | -1.648712 | 5.962256 | 2.10E-02 | 7.85E-02 |
| TRPC3 | 7222 | -1.648467 | 0.4189092 | 9.49E-03 | 4.34E-02 |
| SEMA5B | 54437 | -1.647612 | 3.6482463 | 8.17E-07 | 1.81E-05 |
| METTL24 | 728464 | -1.647072 | 0.1201599 | 3.41E-02 | 1.12E-01 |
| MT-ND5 | 4540 | -1.647064 | 10.927131 | 1.36E-04 | 1.45E-03 |
| CHD5 | 26038 | -1.644369 | 4.1532052 | 2.76E-02 | 9.58E-02 |
| MORC4 | 79710 | -1.644141 | 3.8131232 | 3.62E-03 | 2.06E-02 |
| EFNB2 | 1948 | -1.643418 | 4.8383073 | 1.29E-05 | 1.98E-04 |
| MYOZ2 | 51778 | -1.643196 | 2.0358921 | 1.04E-01 | 2.46E-01 |
| IGLON5 | 402665 | -1.642859 | 1.6261959 | 6.65E-03 | 3.33E-02 |
| POTEE | 445582 | -1.64185 | 2.4639483 | 1.26E-05 | 1.95E-04 |
| CA8 | 767 | -1.641809 | 4.1135527 | 3.17E-03 | 1.85E-02 |
| XYLT1 | 64131 | -1.640239 | 2.2234329 | 9.25E-04 | 6.95E-03 |
| XKR7 | 343702 | -1.6396 | 2.3140642 | 7.67E-03 | 3.72E-02 |
| TSPAN5 | 10098 | -1.638943 | 7.7527898 | 7.95E-12 | 5.01E-10 |
| SNX29 | 92017 | -1.635293 | 4.1583771 | 1.90E-11 | 1.09E-09 |
| LEF1 | 51176 | -1.631488 | 0.8525864 | 7.41E-03 | 3.62E-02 |
| STRIP2 | 57464 | -1.631006 | 0.980898 | 1.26E-03 | 8.91E-03 |
| GPR143 | 4935 | -1.630499 | 2.6558775 | 1.10E-04 | 1.22E-03 |
| FAT1 | 2195 | -1.628352 | 4.3280651 | 1.58E-05 | 2.36E-04 |
| ROBO2 | 6092 | -1.625258 | 5.200272 | 4.38E-02 | 1.34E-01 |
| B4GALNT4 | 338707 | -1.622265 | 3.5250447 | 1.05E-07 | 2.97E-06 |
| NPL | 80896 | -1.61707 | 0.9339979 | 3.32E-02 | 1.10E-01 |
| SLITRK4 | 139065 | -1.616646 | 5.569654 | 7.56E-03 | 3.68E-02 |
| BSCL2 | 26580 | -1.614363 | 2.0992194 | 5.24E-06 | 9.04E-05 |
| PDE3A | 5139 | -1.612658 | 3.5661638 | 8.48E-06 | 1.39E-04 |
| DOCK9 | 23348 | -1.610995 | 1.9571035 | 8.91E-03 | 4.15E-02 |
| ADAM12 | 8038 | -1.610536 | 1.6549069 | 7.04E-02 | 1.87E-01 |
| PXMP2 | 5827 | -1.608935 | 3.1087867 | 1.47E-07 | 4.01E-06 |
| TM6SF1 | 53346 | -1.608287 | 0.1169204 | 4.60E-02 | 1.39E-01 |
| MAOB | 4129 | -1.60774 | 2.9219996 | 3.15E-06 | 5.81E-05 |
| CCDC110 | 256309 | -1.606972 | 1.2299859 | 7.71E-05 | 9.11E-04 |
| SNX32 | 254122 | -1.605934 | 3.0368647 | 1.07E-03 | 7.83E-03 |
| PLXNB3 | 5365 | -1.605927 | 0.1582475 | 1.65E-02 | 6.54E-02 |
| BEND7 | 222389 | -1.605051 | 3.3237594 | 1.06E-06 | 2.29E-05 |
| TTC39B | 158219 | -1.604233 | 1.8722731 | 2.56E-05 | 3.54E-04 |
| PRKCG | 5582 | -1.603724 | 2.3651234 | 2.59E-02 | 9.15E-02 |
| MCAM | 4162 | -1.603395 | 4.3811017 | 2.78E-04 | 2.59E-03 |
| CALHM6 | 441168 | -1.599638 | 0.8488866 | 1.61E-03 | 1.08E-02 |
| EGFL7 | 51162 | -1.598507 | 4.6122897 | 1.92E-13 | 1.62E-11 |
| WTIP | 126374 | -1.596683 | 0.9733953 | 1.15E-03 | 8.30E-03 |
| RAMP2 | 10266 | -1.593212 | 4.8284405 | 5.58E-02 | 1.59E-01 |
| NECTIN1 | 5818 | -1.592646 | 3.4325629 | 8.12E-03 | 3.87E-02 |
| GALK1 | 2584 | -1.592147 | 4.4020891 | 2.83E-10 | 1.39E-08 |
| CACNA1H | 8912 | -1.592013 | 2.5749678 | 6.33E-06 | 1.07E-04 |
| HNMT | 3176 | -1.591422 | 0.0600079 | 1.69E-02 | 6.68E-02 |
| WFDC2 | 10406 | -1.587524 | 3.949461 | 6.27E-02 | 1.72E-01 |
| RWDD2B | 10069 | -1.587009 | 3.3134752 | 4.30E-10 | 2.05E-08 |
| PGF | 5228 | -1.585769 | 0.7127386 | 5.88E-03 | 3.05E-02 |
| CHML | 1122 | -1.585447 | 5.1404198 | 1.13E-13 | 9.94E-12 |
| FBXO32 | 114907 | -1.582691 | 6.8487439 | 4.44E-02 | 1.35E-01 |
| SLC12A4 | 6560 | -1.5822 | 2.7070191 | 1.36E-01 | 2.96E-01 |
| LORICRIN | 4014 | -1.5819 | -0.255303 | 6.94E-02 | 1.85E-01 |
| IGFBP3 | 3486 | -1.580499 | 5.8104905 | 7.94E-02 | 2.03E-01 |
| ZADH2 | 284273 | -1.579187 | 3.4317071 | 3.94E-09 | 1.56E-07 |
| ELL2 | 22936 | -1.577799 | 3.0976549 | 1.44E-02 | 5.91E-02 |
| ARHGDIG | 398 | -1.577297 | 4.1172867 | 1.15E-02 | 5.01E-02 |
| SLC32A1 | 140679 | -1.576087 | 4.6807415 | 2.19E-01 | 4.07E-01 |
| GSDME | 1687 | -1.575042 | 5.4869156 | 4.40E-17 | 6.48E-15 |
| RIBC1 | 158787 | -1.572794 | 1.4080782 | 1.91E-04 | 1.92E-03 |
| QSOX2 | 169714 | -1.570866 | 2.6101685 | 4.95E-08 | 1.54E-06 |
| TOP1MT | 116447 | -1.569575 | 3.3065156 | 7.22E-06 | 1.21E-04 |
| LRRTM3 | 347731 | -1.567142 | 3.8947015 | 2.27E-01 | 4.17E-01 |
| ADGRL3 | 23284 | -1.564673 | 5.1529042 | 4.35E-07 | 1.02E-05 |
| FAM83G | 644815 | -1.56298 | 1.4328891 | 2.00E-02 | 7.59E-02 |
| ASNS | 440 | -1.562943 | 0.8093714 | 1.13E-04 | 1.25E-03 |
| ZNF536 | 9745 | -1.561257 | 4.337578 | 9.06E-08 | 2.61E-06 |
| PARP10 | 84875 | -1.561083 | -0.073397 | 2.46E-02 | 8.82E-02 |
| ACSS3 | 79611 | -1.560974 | 1.7797876 | 1.40E-02 | 5.81E-02 |
| LUZP1 | 7798 | -1.559769 | 5.6666816 | 2.60E-12 | 1.82E-10 |
| NOG | 9241 | -1.55674 | 0.9514977 | 2.01E-02 | 7.61E-02 |
| COL25A1 | 84570 | -1.556161 | 2.5338039 | 1.01E-02 | 4.54E-02 |
| SPATC1L | 84221 | -1.55384 | 4.5973908 | 4.80E-16 | 5.68E-14 |
| METTL7A | 25840 | -1.549469 | 0.325109 | 3.02E-02 | 1.02E-01 |
| HPCA | 3208 | -1.547911 | 2.516832 | 1.12E-01 | 2.59E-01 |
| PCP4 | 5121 | -1.546946 | 8.1852512 | 1.06E-05 | 1.68E-04 |
| TGIF2 | 60436 | -1.546582 | 1.8939049 | 6.90E-02 | 1.85E-01 |
| ESRRA | 2101 | -1.542703 | 3.7286328 | 1.92E-07 | 4.99E-06 |
| RFXANK | 8625 | -1.541863 | 3.8841538 | 2.99E-09 | 1.20E-07 |
| ENTPD3 | 956 | -1.541205 | 3.9210645 | 1.15E-04 | 1.27E-03 |
| ARMC7 | 79637 | -1.540953 | 1.2740974 | 3.42E-05 | 4.50E-04 |
| B3GAT2 | 135152 | -1.540086 | 3.8083665 | 2.65E-08 | 8.77E-07 |
| ATXN1 | 6310 | -1.538923 | 4.4639015 | 3.27E-08 | 1.07E-06 |
| ADAMTS9 | 56999 | -1.538047 | 1.7267854 | 4.28E-03 | 2.35E-02 |
| CSMD1 | 64478 | -1.53714 | 1.8444583 | 2.55E-03 | 1.56E-02 |
| MTHFD2L | 441024 | -1.535939 | 4.742997 | 1.18E-08 | 4.24E-07 |
| FNDC4 | 64838 | -1.535515 | 7.7677228 | 9.36E-09 | 3.42E-07 |
| SOX1 | 6656 | -1.533448 | 3.5217329 | 2.06E-09 | 8.67E-08 |
| LIMD1 | 8994 | -1.52801 | 0.6438818 | 1.70E-02 | 6.70E-02 |
| ADAMTS6 | 11174 | -1.526783 | 0.5248742 | 7.21E-02 | 1.91E-01 |
| SLC12A8 | 84561 | -1.526642 | 2.0824022 | 1.26E-03 | 8.91E-03 |
| GYPC | 2995 | -1.524828 | 2.9553187 | 5.36E-02 | 1.54E-01 |
| GABRG2 | 2566 | -1.524456 | 7.05166 | 1.90E-01 | 3.71E-01 |
| SLC7A4 | 6545 | -1.524276 | 1.3257768 | 1.30E-06 | 2.71E-05 |
| ARSA | 410 | -1.523643 | 3.8490298 | 3.19E-08 | 1.04E-06 |
| SLC27A2 | 11001 | -1.523291 | 4.285022 | 4.03E-05 | 5.19E-04 |
| FILIP1 | 27145 | -1.523009 | 1.4736883 | 5.29E-04 | 4.41E-03 |
| SH3RF1 | 57630 | -1.521685 | 4.5684172 | 4.85E-07 | 1.12E-05 |
| PDK3 | 5165 | -1.521175 | 6.6095888 | 4.47E-06 | 7.91E-05 |
| SLITRK3 | 22865 | -1.521106 | 3.4212054 | 4.50E-04 | 3.89E-03 |
| PEX6 | 5190 | -1.518361 | 2.9377037 | 1.68E-06 | 3.39E-05 |
| TC2N | 123036 | -1.516935 | 1.7363424 | 5.77E-03 | 3.01E-02 |
| ANKH | 56172 | -1.514778 | 4.3881399 | 4.72E-03 | 2.55E-02 |
| LGALS3 | 3958 | -1.51315 | 4.7014655 | 3.65E-03 | 2.08E-02 |
| MGST1 | 4257 | -1.512617 | 5.9757316 | 1.60E-04 | 1.65E-03 |
| NHSL1 | 57224 | -1.509647 | 2.2134577 | 3.84E-02 | 1.22E-01 |
| TMEM54 | 113452 | -1.509321 | 3.0266278 | 1.09E-04 | 1.22E-03 |
| CNOT6L | 246175 | -1.508837 | 3.7680144 | 1.89E-06 | 3.75E-05 |
| ERICH5 | 203111 | -1.507895 | 3.1178434 | 7.75E-08 | 2.29E-06 |
| PTCH2 | 8643 | -1.50717 | 2.0641948 | 1.01E-03 | 7.46E-03 |
| ANAPC2 | 29882 | -1.506092 | 1.1116095 | 1.56E-02 | 6.29E-02 |
| PI4K2B | 55300 | -1.505308 | 1.6727534 | 1.84E-02 | 7.10E-02 |
| APOO | 79135 | -1.504363 | 7.7193497 | 6.91E-10 | 3.20E-08 |
| CD247 | 919 | -1.504123 | 0.4332107 | 8.98E-02 | 2.21E-01 |
| SLC13A3 | 64849 | -1.503864 | 1.7864575 | 1.17E-05 | 1.83E-04 |
| BTN3A3 | 10384 | -1.502486 | 2.0354789 | 1.54E-02 | 6.24E-02 |
| NKX3-2 | 579 | -1.502143 | 0.1137791 | 1.24E-01 | 2.78E-01 |
| KIRREL2 | 84063 | -1.501643 | 0.3810689 | 9.91E-03 | 4.48E-02 |
| SLC16A13 | 201232 | -1.501612 | 1.8791388 | 1.38E-03 | 9.54E-03 |
| DLGAP1 | 9229 | -1.498244 | 3.8707676 | 1.41E-03 | 9.70E-03 |
| ROR1 | 4919 | -1.496916 | 2.4052413 | 1.12E-04 | 1.24E-03 |
| VSTM2L | 128434 | -1.495683 | 4.3525944 | 8.57E-03 | 4.04E-02 |
| THBS1 | 7057 | -1.49389 | 5.0958415 | 1.48E-01 | 3.12E-01 |
| BTBD19 | 149478 | -1.493217 | 0.1374843 | 2.73E-01 | 4.69E-01 |
| PCGF5 | 84333 | -1.489721 | 5.483478 | 1.10E-02 | 4.86E-02 |
| LTBP1 | 4052 | -1.48955 | 1.9765102 | 1.16E-02 | 5.04E-02 |
| LDB2 | 9079 | -1.488194 | 3.6131321 | 8.39E-03 | 3.97E-02 |
| NOP2 | 4839 | -1.486322 | 0.8028054 | 5.98E-03 | 3.09E-02 |
| PCSK7 | 9159 | -1.483433 | 3.7685803 | 1.63E-09 | 7.05E-08 |
| FIBCD1 | 84929 | -1.480273 | -0.048734 | 3.82E-02 | 1.22E-01 |
| GRID1 | 2894 | -1.478963 | 1.557974 | 1.80E-04 | 1.82E-03 |
| IL17RC | 84818 | -1.478875 | 1.8074004 | 1.32E-05 | 2.02E-04 |
| PTGFRN | 5738 | -1.477802 | 4.0993687 | 2.42E-03 | 1.49E-02 |
| CHTF18 | 63922 | -1.4721 | 0.6835172 | 7.61E-02 | 1.98E-01 |
| PPP1R12C | 54776 | -1.471774 | 3.5793296 | 5.07E-06 | 8.77E-05 |
| TMEM38A | 79041 | -1.468094 | 5.6405881 | 2.56E-09 | 1.05E-07 |
| ASCL1 | 429 | -1.466747 | 2.7122408 | 3.20E-03 | 1.86E-02 |
| LDLRAD4 | 753 | -1.466466 | 3.2926652 | 7.23E-04 | 5.71E-03 |
| APOA1 | 335 | -1.465485 | -0.261943 | 1.93E-01 | 3.75E-01 |
| RYR3 | 6263 | -1.463509 | 2.4389426 | 6.84E-06 | 1.15E-04 |
| TFAP2C | 7022 | -1.463465 | 4.1833563 | 6.12E-02 | 1.70E-01 |
| IRX6 | 79190 | -1.462986 | -0.186064 | 1.59E-02 | 6.39E-02 |
| TMEM117 | 84216 | -1.462938 | 3.204624 | 6.43E-04 | 5.18E-03 |
| PRDX4 | 10549 | -1.4624 | 7.5420683 | 4.02E-12 | 2.73E-10 |
| NACC2 | 138151 | -1.462357 | 2.8336115 | 3.45E-03 | 1.98E-02 |
| HCRTR2 | 3062 | -1.462119 | 4.1596125 | 1.47E-01 | 3.11E-01 |
| DSG2 | 1829 | -1.458095 | 2.996809 | 1.61E-03 | 1.08E-02 |
| MARCHF3 | 115123 | -1.457766 | 3.1720324 | 7.13E-09 | 2.66E-07 |
| EN2 | 2020 | -1.455192 | 3.3977646 | 9.06E-02 | 2.23E-01 |
| SEMA7A | 8482 | -1.45508 | 1.7249753 | 1.29E-01 | 2.86E-01 |
| TPBG | 7162 | -1.454627 | 4.4257229 | 4.91E-04 | 4.17E-03 |
| ELK4 | 2005 | -1.454437 | 1.8210766 | 1.57E-03 | 1.06E-02 |
| IGFBP4 | 3487 | -1.453796 | 4.4601514 | 3.76E-03 | 2.13E-02 |
| ANG | 283 | -1.451808 | 0.5197578 | 3.31E-02 | 1.10E-01 |
| CDH4 | 1002 | -1.449829 | 5.7530259 | 1.10E-10 | 5.76E-09 |
| KCNMA1 | 3778 | -1.449785 | 5.3917274 | 3.80E-04 | 3.38E-03 |
| CFAP300 | 85016 | -1.449468 | 2.9077773 | 2.40E-03 | 1.48E-02 |
| ALDH1A2 | 8854 | -1.449388 | -0.061403 | 1.90E-02 | 7.30E-02 |
| CD74 | 972 | -1.44881 | 5.1638832 | 1.63E-01 | 3.33E-01 |
| MT-ND1 | 4535 | -1.446446 | 10.190915 | 2.57E-04 | 2.43E-03 |
| SEMA3F | 6405 | -1.445397 | 0.9564932 | 1.12E-01 | 2.59E-01 |
| VEGFB | 7423 | -1.445027 | 5.451708 | 7.06E-09 | 2.64E-07 |
| SERF2 | 10169 | -1.4433 | 8.0812618 | 1.58E-11 | 9.20E-10 |
| COX5B | 1329 | -1.441319 | 9.5238923 | 4.20E-06 | 7.45E-05 |
| CRNDE | 643911 | -1.440595 | 4.2881301 | 1.54E-08 | 5.38E-07 |
| CENPM | 79019 | -1.439775 | 2.4949258 | 5.44E-04 | 4.52E-03 |
| JUNB | 3726 | -1.439585 | 3.7162876 | 9.32E-12 | 5.79E-10 |
| OPRM1 | 4988 | -1.438588 | 1.4531543 | 5.54E-02 | 1.58E-01 |
| NINL | 22981 | -1.438366 | 3.1578274 | 1.54E-08 | 5.37E-07 |
| NTS | 4922 | -1.437514 | 6.4185282 | 4.60E-02 | 1.39E-01 |
| TMEM91 | 641649 | -1.436572 | 3.0537976 | 2.60E-02 | 9.18E-02 |
| MGLL | 11343 | -1.436484 | 5.4013361 | 3.31E-03 | 1.91E-02 |
| IKBKB | 3551 | -1.435318 | 1.6320478 | 1.63E-03 | 1.09E-02 |
| S100A16 | 140576 | -1.434592 | 4.4533157 | 1.50E-03 | 1.02E-02 |
| BLM | 641 | -1.433157 | 0.4986304 | 6.45E-02 | 1.75E-01 |
| PCNX3 | 399909 | -1.432102 | 2.2144771 | 3.07E-03 | 1.81E-02 |
| QKI | 9444 | -1.431522 | 5.8432146 | 9.21E-07 | 2.02E-05 |
| SNTG2 | 54221 | -1.430132 | 0.0947868 | 1.40E-02 | 5.81E-02 |
| GALNT14 | 79623 | -1.429448 | 2.9567092 | 1.19E-01 | 2.69E-01 |
| ATF6B | 1388 | -1.429326 | 2.9146088 | 1.44E-05 | 2.19E-04 |
| CREB3L2 | 64764 | -1.429315 | 2.7913613 | 2.68E-03 | 1.63E-02 |
| TTPA | 7274 | -1.428814 | 0.3419082 | 2.74E-01 | 4.70E-01 |
| C8orf34 | 116328 | -1.428552 | 1.4171179 | 2.25E-01 | 4.14E-01 |
| USP44 | 84101 | -1.425059 | 1.5066628 | 3.54E-04 | 3.18E-03 |
| PNPLA6 | 10908 | -1.424958 | 2.0165852 | 8.55E-05 | 9.89E-04 |
| KCNS2 | 3788 | -1.424242 | 2.6000792 | 8.65E-04 | 6.57E-03 |
| MARCHF11 | 441061 | -1.4239 | 2.0556415 | 1.28E-01 | 2.84E-01 |
| CYYR1 | 116159 | -1.422145 | 2.0414963 | 1.43E-01 | 3.06E-01 |
| SLC9A5 | 6553 | -1.420635 | 1.3877288 | 9.97E-04 | 7.37E-03 |
| EFHC2 | 80258 | -1.418525 | 1.9123949 | 2.88E-03 | 1.72E-02 |
| RHOF | 54509 | -1.417143 | 0.5532323 | 1.47E-03 | 1.01E-02 |
| BAHD1 | 22893 | -1.41669 | 2.0689456 | 6.48E-06 | 1.09E-04 |
| CDH6 | 1004 | -1.414896 | 4.0124932 | 2.21E-04 | 2.16E-03 |
| KCNAB1 | 7881 | -1.413078 | 3.556721 | 2.05E-03 | 1.31E-02 |
| FOXM1 | 2305 | -1.411976 | -0.042144 | 1.43E-01 | 3.06E-01 |
| RDH10 | 157506 | -1.411374 | 2.3880032 | 8.00E-02 | 2.04E-01 |
| TRAF3 | 7187 | -1.410256 | 3.4919561 | 1.79E-05 | 2.63E-04 |
| FKBP10 | 60681 | -1.410237 | 1.6025657 | 1.46E-02 | 5.99E-02 |
| TRIM5 | 85363 | -1.409868 | 3.3482554 | 1.55E-02 | 6.25E-02 |
| NDUFA4 | 4697 | -1.40856 | 10.857234 | 6.16E-05 | 7.58E-04 |
| TMEM220 | 388335 | -1.408472 | 0.596792 | 2.45E-02 | 8.82E-02 |
| HACD4 | 401494 | -1.406309 | 0.2998996 | 2.06E-02 | 7.74E-02 |
| NUDT6 | 11162 | -1.404179 | 2.2214253 | 1.78E-03 | 1.17E-02 |
| MAP7D3 | 79649 | -1.404144 | 2.4341307 | 3.02E-02 | 1.02E-01 |
| DBF4B | 80174 | -1.403914 | 1.4392818 | 2.97E-05 | 4.03E-04 |
| ADRA2A | 150 | -1.403708 | 2.2547627 | 8.58E-02 | 2.14E-01 |
| HOMER2 | 9455 | -1.400452 | 4.3288546 | 8.28E-04 | 6.36E-03 |
| SYCE1L | 100130958 | -1.399728 | 1.931546 | 2.18E-02 | 8.08E-02 |
| SATB1 | 6304 | -1.398982 | 1.4306579 | 2.67E-03 | 1.62E-02 |
| RTKN2 | 219790 | -1.398874 | 1.0233859 | 2.70E-03 | 1.63E-02 |
| PSMG3 | 84262 | -1.397378 | 5.9323277 | 8.08E-10 | 3.65E-08 |
| UNC5D | 137970 | -1.396628 | 5.2447671 | 1.05E-01 | 2.48E-01 |
| ZNF687 | 57592 | -1.395691 | 0.1689476 | 2.03E-03 | 1.30E-02 |
| HOOK2 | 29911 | -1.394082 | 3.2883905 | 6.93E-03 | 3.44E-02 |
| NWD2 | 57495 | -1.392197 | 0.0852455 | 1.92E-02 | 7.34E-02 |
| NRSN1 | 140767 | -1.391219 | 6.3993679 | 3.36E-01 | 5.37E-01 |
| HS6ST2 | 90161 | -1.390008 | 5.9987479 | 5.17E-03 | 2.76E-02 |
| LHFPL5 | 222662 | -1.38968 | 1.053976 | 9.83E-02 | 2.36E-01 |
| CNFN | 84518 | -1.389072 | 1.0559676 | 4.72E-02 | 1.41E-01 |
| MT-ND2 | 4536 | -1.388732 | 11.720939 | 5.71E-04 | 4.70E-03 |
| GJC1 | 10052 | -1.387032 | 3.7102063 | 2.36E-06 | 4.52E-05 |
| PNCK | 139728 | -1.386792 | 1.9430196 | 1.61E-01 | 3.31E-01 |
| CRYL1 | 51084 | -1.386683 | 4.9716383 | 2.87E-08 | 9.46E-07 |
| ULK1 | 8408 | -1.386648 | 3.0197623 | 1.21E-05 | 1.89E-04 |
| ANTXR2 | 118429 | -1.383852 | 2.7486151 | 9.04E-03 | 4.19E-02 |
| ZDHHC11 | 79844 | -1.383631 | 0.6614686 | 1.45E-03 | 9.96E-03 |
| COX6A1 | 1337 | -1.383439 | 10.204103 | 1.33E-04 | 1.41E-03 |
| LRRTM1 | 347730 | -1.38284 | 3.1418921 | 1.11E-01 | 2.58E-01 |
| ZDHHC24 | 254359 | -1.382797 | 4.8610249 | 8.05E-06 | 1.33E-04 |
| ADCYAP1R1 | 117 | -1.38194 | 3.2269041 | 3.20E-02 | 1.07E-01 |
| CAMK2N2 | 94032 | -1.381385 | 3.2980221 | 5.90E-03 | 3.06E-02 |
| KIF26A | 26153 | -1.381173 | 0.8772659 | 4.54E-02 | 1.37E-01 |
| DDN | 23109 | -1.38058 | 2.6168574 | 1.85E-01 | 3.64E-01 |
| PRRG4 | 79056 | -1.38038 | 1.3436343 | 3.11E-03 | 1.82E-02 |
| AKNA | 80709 | -1.379879 | 0.3420567 | 3.15E-02 | 1.05E-01 |
| ZDHHC23 | 254887 | -1.378729 | 0.3831098 | 2.83E-02 | 9.73E-02 |
| FAM184B | 27146 | -1.377473 | 1.3144645 | 5.33E-04 | 4.44E-03 |
| LIPE | 3991 | -1.376346 | 0.3501515 | 4.41E-03 | 2.40E-02 |
| TPCN1 | 53373 | -1.376045 | 2.5406698 | 3.37E-02 | 1.11E-01 |
| KIT | 3815 | -1.37285 | 2.6307916 | 6.57E-03 | 3.30E-02 |
| LRRC56 | 115399 | -1.372683 | 0.5142861 | 2.64E-02 | 9.28E-02 |
| EBF3 | 253738 | -1.372205 | 2.1669587 | 1.04E-01 | 2.46E-01 |
| MEGF8 | 1954 | -1.371106 | 4.7349544 | 2.55E-10 | 1.26E-08 |
| TNMD | 64102 | -1.368605 | 2.732028 | 3.52E-01 | 5.52E-01 |
| NMRAL1 | 57407 | -1.367786 | 5.030821 | 1.17E-03 | 8.41E-03 |
| HIF3A | 64344 | -1.365417 | 2.0231494 | 2.95E-03 | 1.74E-02 |
| FAM107A | 11170 | -1.364898 | 2.9808741 | 1.55E-02 | 6.27E-02 |
| VWCE | 220001 | -1.364838 | 0.7100333 | 1.39E-03 | 9.60E-03 |
| CTSZ | 1522 | -1.364741 | 0.3456567 | 3.42E-02 | 1.12E-01 |
| EVC | 2121 | -1.3645 | 1.4174128 | 1.96E-05 | 2.84E-04 |
| C2CD2 | 25966 | -1.363733 | 2.5596331 | 4.80E-03 | 2.59E-02 |
| CITED2 | 10370 | -1.363573 | 3.2564141 | 1.02E-05 | 1.64E-04 |
| MACROD1 | 28992 | -1.363315 | 4.3471446 | 3.30E-07 | 8.09E-06 |
| TMSB10 | 9168 | -1.363254 | 12.653452 | 1.09E-02 | 4.82E-02 |
| KCNK1 | 3775 | -1.363145 | 2.8781657 | 7.03E-03 | 3.47E-02 |
| PTPRG | 5793 | -1.363085 | 4.1170869 | 2.94E-09 | 1.19E-07 |
| NDRG2 | 57447 | -1.36238 | 5.7209391 | 7.88E-03 | 3.79E-02 |
| TIMP1 | 7076 | -1.36017 | 5.6711032 | 2.54E-02 | 9.03E-02 |
| SSPN | 8082 | -1.36003 | 1.7428956 | 3.21E-02 | 1.07E-01 |
| DGKB | 1607 | -1.359748 | 1.8105831 | 3.65E-02 | 1.18E-01 |
| SPCS3 | 60559 | -1.359745 | 5.6742058 | 2.08E-08 | 7.00E-07 |
| MPST | 4357 | -1.3595 | 5.0811964 | 2.30E-10 | 1.15E-08 |
| FOXO1 | 2308 | -1.359412 | 1.9302661 | 1.62E-03 | 1.09E-02 |
| GABRA2 | 2555 | -1.357993 | 7.5000824 | 1.47E-01 | 3.11E-01 |
| ATP10A | 57194 | -1.356537 | 1.4048709 | 5.57E-02 | 1.59E-01 |
| BLOC1S1 | 2647 | -1.356399 | 4.068278 | 7.16E-05 | 8.55E-04 |
| PLEKHA7 | 144100 | -1.355445 | 2.8860646 | 3.61E-03 | 2.06E-02 |
| SPTLC3 | 55304 | -1.355227 | 0.2535007 | 8.34E-02 | 2.10E-01 |
| PREX1 | 57580 | -1.354131 | 2.794574 | 1.84E-02 | 7.12E-02 |
| PLCH1 | 23007 | -1.35284 | 2.7968425 | 1.29E-05 | 1.98E-04 |
| PIK3CD | 5293 | -1.348128 | 2.39287 | 4.75E-03 | 2.56E-02 |
| NTRK2 | 4915 | -1.347949 | 8.2032942 | 7.07E-04 | 5.62E-03 |
| PCSK1N | 27344 | -1.347878 | 9.7640957 | 4.47E-03 | 2.43E-02 |
| KIF5C | 3800 | -1.347415 | 8.5414278 | 4.77E-03 | 2.57E-02 |
| POU6F1 | 5463 | -1.346407 | 3.4995092 | 2.03E-04 | 2.01E-03 |
| TMEM47 | 83604 | -1.345365 | 6.0225974 | 1.89E-07 | 4.94E-06 |
| SMIM32 | 389332 | -1.343814 | 1.6368853 | 1.87E-01 | 3.67E-01 |
| INPPL1 | 3636 | -1.343747 | 0.9503658 | 1.31E-01 | 2.89E-01 |
| CYBA | 1535 | -1.342969 | 1.3615468 | 5.08E-02 | 1.48E-01 |
| NEDD4 | 4734 | -1.342785 | 1.2297239 | 7.04E-02 | 1.87E-01 |
| SMAD3 | 4088 | -1.342399 | 2.2294615 | 6.11E-02 | 1.70E-01 |
| PTCHD4 | 442213 | -1.341003 | 3.8961317 | 1.01E-02 | 4.55E-02 |
| SKIV2L | 6499 | -1.33938 | 2.0016102 | 3.10E-04 | 2.86E-03 |
| C4orf48 | 401115 | -1.338622 | 8.7379053 | 4.37E-03 | 2.39E-02 |
| OFD1 | 8481 | -1.335779 | 4.9828366 | 5.30E-08 | 1.62E-06 |
| SHC2 | 25759 | -1.334196 | 3.7561038 | 4.36E-05 | 5.56E-04 |
| PRKCA | 5578 | -1.333386 | 3.8681541 | 1.15E-05 | 1.81E-04 |
| AMZ1 | 155185 | -1.332144 | 0.0530728 | 2.10E-02 | 7.85E-02 |
| SMPD1 | 6609 | -1.330679 | 2.8142212 | 2.87E-04 | 2.67E-03 |
| MYOZ3 | 91977 | -1.329176 | 2.1688253 | 4.17E-03 | 2.31E-02 |
| CBFA2T3 | 863 | -1.328428 | 1.9198645 | 2.95E-03 | 1.74E-02 |
| COMTD1 | 118881 | -1.328359 | 5.5016565 | 9.73E-03 | 4.42E-02 |
| IGSF9B | 22997 | -1.328353 | 2.6992339 | 4.63E-06 | 8.16E-05 |
| ATP5MC1 | 516 | -1.327283 | 8.5415187 | 1.21E-04 | 1.32E-03 |
| ISOC2 | 79763 | -1.326219 | 5.0584153 | 1.01E-07 | 2.85E-06 |
| DNMBP | 23268 | -1.326137 | 1.7683347 | 4.78E-02 | 1.42E-01 |
| TNFRSF9 | 3604 | -1.324478 | -0.065776 | 2.06E-01 | 3.91E-01 |
| FURIN | 5045 | -1.324207 | 3.4667611 | 2.50E-03 | 1.54E-02 |
| PCDHB9 | 56127 | -1.324066 | 1.5863549 | 7.78E-03 | 3.76E-02 |
| TUBA1A | 7846 | -1.324035 | 14.683382 | 9.11E-03 | 4.21E-02 |
| RTL1 | 388015 | -1.323232 | 1.1463104 | 1.61E-02 | 6.42E-02 |
| CNTNAP1 | 8506 | -1.320297 | 0.4594758 | 4.07E-02 | 1.27E-01 |
| ISLR2 | 57611 | -1.319111 | 6.4094506 | 2.25E-01 | 4.14E-01 |
| IQCD | 115811 | -1.318815 | 2.9973916 | 7.76E-03 | 3.76E-02 |
| CATSPER2 | 117155 | -1.318207 | 0.2341691 | 2.31E-02 | 8.41E-02 |
| MBNL2 | 10150 | -1.317801 | 4.1001922 | 7.37E-03 | 3.60E-02 |
| ST6GAL2 | 84620 | -1.317047 | 5.1614705 | 2.67E-01 | 4.63E-01 |
| LHFPL2 | 10184 | -1.316208 | 2.2654434 | 5.74E-02 | 1.62E-01 |
| ADGRB2 | 576 | -1.315705 | 4.3746247 | 9.34E-07 | 2.04E-05 |
| FUT4 | 2526 | -1.314533 | 0.5171333 | 1.16E-02 | 5.06E-02 |
| CELSR2 | 1952 | -1.31367 | 4.6931066 | 6.26E-06 | 1.06E-04 |
| HOPX | 84525 | -1.313087 | 2.4272221 | 7.93E-03 | 3.81E-02 |
| PDXK | 105372824 | -1.312979 | 6.579495 | 1.99E-09 | 8.45E-08 |
| TEAD3 | 7005 | -1.310067 | 0.8403666 | 3.43E-01 | 5.43E-01 |
| MAP3K10 | 4294 | -1.308164 | 1.2511175 | 1.65E-02 | 6.54E-02 |
| DRAXIN | 374946 | -1.3073 | 5.2634662 | 1.15E-01 | 2.63E-01 |
| SERPINE2 | 5270 | -1.30679 | 7.5193186 | 2.13E-03 | 1.35E-02 |
| KIF7 | 374654 | -1.30635 | 1.4224077 | 1.95E-04 | 1.95E-03 |
| ACVR1B | 91 | -1.304814 | 3.7640435 | 5.09E-04 | 4.28E-03 |
| LPL | 4023 | -1.303011 | 3.2478935 | 1.81E-02 | 7.03E-02 |
| KLF2 | 10365 | -1.302817 | -0.059832 | 2.80E-02 | 9.67E-02 |
| STAP2 | 55620 | -1.301867 | 1.9972214 | 1.36E-04 | 1.44E-03 |
| ENC1 | 8507 | -1.300539 | 7.2984361 | 1.21E-04 | 1.32E-03 |
| AEBP1 | 165 | -1.300179 | 1.245776 | 2.74E-03 | 1.65E-02 |
| RABAC1 | 10567 | -1.299378 | 4.560493 | 9.76E-12 | 6.03E-10 |
| CEND1 | 51286 | -1.299262 | 6.7673437 | 2.81E-04 | 2.62E-03 |
| SLC6A9 | 6536 | -1.296843 | 3.0175865 | 1.13E-03 | 8.13E-03 |
| PRR16 | 51334 | -1.295792 | 1.7809452 | 4.67E-02 | 1.40E-01 |
| ABHD1 | 84696 | -1.295138 | 0.0004492 | 6.54E-02 | 1.77E-01 |
| GSTO1 | 9446 | -1.294855 | 6.6377174 | 9.78E-07 | 2.12E-05 |
| ZC3HAV1L | 92092 | -1.294462 | 2.1611992 | 5.84E-04 | 4.78E-03 |
| CDK16 | 5127 | -1.291622 | 7.0362181 | 8.92E-11 | 4.72E-09 |
| SYVN1 | 84447 | -1.291395 | 1.9225369 | 6.74E-04 | 5.39E-03 |
| ERICH3 | 127254 | -1.291208 | 0.7534967 | 1.53E-02 | 6.20E-02 |
| ADCY5 | 111 | -1.291046 | 4.2445464 | 3.19E-02 | 1.07E-01 |
| SKI | 6497 | -1.290054 | 1.5357707 | 1.96E-03 | 1.26E-02 |
| RNASEL | 6041 | -1.289835 | 1.4777479 | 9.95E-05 | 1.12E-03 |
| BOC | 91653 | -1.287479 | 3.0093891 | 9.60E-02 | 2.32E-01 |
| SOCS2 | 8835 | -1.286605 | 5.3303742 | 4.88E-08 | 1.53E-06 |
| MRPL38 | 64978 | -1.284846 | 0.0849686 | 2.78E-02 | 9.64E-02 |
| GABRA5 | 2558 | -1.284545 | 7.1471873 | 6.35E-02 | 1.74E-01 |
| SHISA2 | 387914 | -1.284038 | 1.4653912 | 1.92E-02 | 7.35E-02 |
| ZBTB20 | 26137 | -1.283473 | 4.6283302 | 9.65E-03 | 4.39E-02 |
| PDGFC | 56034 | -1.282788 | 1.5860541 | 1.39E-02 | 5.76E-02 |
| LSM7 | 51690 | -1.280281 | 7.2772892 | 1.77E-03 | 1.17E-02 |
| SLC45A4 | 57210 | -1.280012 | 1.7481345 | 6.50E-04 | 5.22E-03 |
| CTDSP1 | 58190 | -1.279124 | 0.2156063 | 2.55E-01 | 4.49E-01 |
| MMP16 | 4325 | -1.277873 | 3.7162215 | 3.50E-06 | 6.32E-05 |
| CORO2A | 7464 | -1.277202 | 3.5651456 | 1.49E-01 | 3.13E-01 |
| NIT2 | 56954 | -1.275278 | 5.7467478 | 7.98E-10 | 3.61E-08 |
| TPD52L1 | 7164 | -1.275018 | 6.5451604 | 1.40E-03 | 9.64E-03 |
| SMOC2 | 64094 | -1.273045 | 1.9657613 | 4.85E-02 | 1.44E-01 |
| C6orf141 | 135398 | -1.272377 | 0.446928 | 5.66E-03 | 2.96E-02 |
| TMEM141 | 85014 | -1.271844 | 3.8356537 | 6.90E-08 | 2.07E-06 |
| DENND3 | 22898 | -1.268706 | 1.2241637 | 3.43E-02 | 1.12E-01 |
| IGIP | 492311 | -1.267654 | 0.642771 | 2.50E-02 | 8.93E-02 |
| C3orf70 | 285382 | -1.266287 | 3.5763416 | 2.59E-02 | 9.15E-02 |
| STEAP1B | 256227 | -1.266248 | 0.3764572 | 1.61E-01 | 3.31E-01 |
| TCFL5 | 10732 | -1.264644 | 1.336081 | 5.81E-05 | 7.21E-04 |
| APOBEC3C | 27350 | -1.264625 | 2.2075281 | 1.87E-01 | 3.66E-01 |
| NKAIN2 | 154215 | -1.264594 | 3.6658937 | 4.95E-02 | 1.46E-01 |
| SERHL2 | 253190 | -1.264358 | 0.1525806 | 2.57E-02 | 9.11E-02 |
| TSPO | 706 | -1.264348 | 5.840743 | 1.17E-07 | 3.27E-06 |
| H4C3 | 8364 | -1.264311 | 8.9573555 | 4.56E-02 | 1.38E-01 |
| NPAS4 | 266743 | -1.263959 | 0.9915119 | 1.59E-02 | 6.37E-02 |
| RINL | 126432 | -1.263651 | 3.5157885 | 2.09E-03 | 1.33E-02 |
| TMEM160 | 54958 | -1.261685 | 7.3110344 | 2.33E-04 | 2.24E-03 |
| SLC22A17 | 51310 | -1.261631 | 7.9021037 | 2.03E-07 | 5.27E-06 |
| SFXN4 | 119559 | -1.260785 | 5.427388 | 8.77E-09 | 3.25E-07 |
| CAPS | 828 | -1.259131 | 0.8100936 | 1.65E-02 | 6.54E-02 |
| TRIM21 | 6737 | -1.258974 | 3.2182181 | 2.29E-03 | 1.42E-02 |
| KREMEN1 | 83999 | -1.258376 | 1.9862481 | 1.66E-01 | 3.37E-01 |
| VAV3 | 10451 | -1.25707 | 2.4258326 | 2.58E-02 | 9.14E-02 |
| MIDEAS | 91748 | -1.256712 | 3.4672831 | 2.79E-03 | 1.68E-02 |
| AGAP2 | 116986 | -1.256164 | 2.2601539 | 2.32E-01 | 4.24E-01 |
| TMEM256 | 254863 | -1.255074 | 3.1617785 | 1.32E-04 | 1.40E-03 |
| ASPDH | 554235 | -1.254614 | 1.7541842 | 2.33E-01 | 4.25E-01 |
| BMPR1B | 658 | -1.254569 | 0.2002711 | 2.63E-01 | 4.58E-01 |
| SH2D7 | 646892 | -1.254479 | -0.337232 | 5.58E-02 | 1.59E-01 |
| GLIS2 | 84662 | -1.253491 | 1.9327421 | 1.27E-03 | 8.93E-03 |
| ARID5A | 10865 | -1.253087 | 4.0557164 | 1.54E-04 | 1.60E-03 |
| GPR153 | 387509 | -1.25302 | 5.2879548 | 1.57E-02 | 6.32E-02 |
| IGFL4 | 444882 | -1.252537 | 0.1778037 | 3.13E-02 | 1.05E-01 |
| ATP1A2 | 477 | -1.252227 | 0.705899 | 1.21E-01 | 2.74E-01 |
| DAB1 | 1600 | -1.251976 | 4.3830403 | 3.99E-02 | 1.25E-01 |
| SLC9A2 | 6549 | -1.251872 | 0.1381245 | 5.64E-02 | 1.60E-01 |
| DNAH10 | 196385 | -1.251681 | 2.6250156 | 1.11E-02 | 4.90E-02 |
| OR1F1 | 4992 | -1.251313 | 0.4927863 | 2.50E-02 | 8.93E-02 |
| FABP6 | 2172 | -1.251289 | 1.9817862 | 1.60E-01 | 3.29E-01 |
| PLD1 | 5337 | -1.251075 | 0.3604078 | 6.34E-02 | 1.74E-01 |
| COL6A1 | 1291 | -1.250878 | 4.1599983 | 8.67E-03 | 4.07E-02 |
| TMEM177 | 80775 | -1.250247 | 4.6517522 | 3.35E-10 | 1.62E-08 |
| LRP1 | 4035 | -1.248898 | 5.4547125 | 1.69E-04 | 1.72E-03 |
| FTH1 | 2495 | -1.247918 | 11.418929 | 4.50E-04 | 3.89E-03 |
| SLC16A10 | 117247 | -1.247301 | 1.0852543 | 3.14E-03 | 1.83E-02 |
| STXBP6 | 29091 | -1.246663 | 2.9107017 | 1.34E-06 | 2.78E-05 |
| RFX1 | 5989 | -1.24572 | 0.8719753 | 1.60E-02 | 6.40E-02 |
| MZT2A | 653784 | -1.24537 | 7.1776896 | 4.15E-05 | 5.32E-04 |
| IGSF3 | 3321 | -1.245247 | 5.2233429 | 8.99E-05 | 1.03E-03 |
| MFAP3L | 9848 | -1.245027 | 2.9508171 | 1.66E-02 | 6.59E-02 |
| GPM6B | 2824 | -1.242736 | 8.6566153 | 1.11E-11 | 6.71E-10 |
| ITPKB | 3707 | -1.242041 | 3.1063147 | 3.02E-01 | 5.01E-01 |
| TMEM217 | 221468 | -1.242026 | 0.21648 | 5.48E-02 | 1.57E-01 |
| RNASEK | 440400 | -1.241185 | 1.5678434 | 2.24E-03 | 1.40E-02 |
| GLCCI1 | 113263 | -1.239917 | 5.318076 | 5.73E-04 | 4.71E-03 |
| ICAM5 | 7087 | -1.239818 | 0.7287395 | 2.15E-01 | 4.03E-01 |
| SEZ6L2 | 26470 | -1.239301 | 6.1771467 | 1.09E-01 | 2.55E-01 |
| ARHGAP19 | 84986 | -1.238871 | 0.8903377 | 2.90E-02 | 9.91E-02 |
| TENM3 | 55714 | -1.237944 | 4.4897528 | 2.29E-04 | 2.21E-03 |
| SLITRK1 | 114798 | -1.236857 | 1.3644873 | 1.25E-01 | 2.79E-01 |
| CCDC85B | 11007 | -1.236715 | 6.5276762 | 2.41E-10 | 1.20E-08 |
| ATP5F1D | 513 | -1.235779 | 8.0376279 | 6.39E-05 | 7.80E-04 |
| ASPHD1 | 253982 | -1.235664 | 3.6923668 | 3.23E-02 | 1.08E-01 |
| PLEKHG3 | 26030 | -1.234751 | 2.4265939 | 4.88E-03 | 2.62E-02 |
| CABP1 | 9478 | -1.232641 | 1.2321672 | 2.99E-02 | 1.02E-01 |
| NR1H2 | 7376 | -1.231556 | 5.6054799 | 1.17E-03 | 8.38E-03 |
| FOSL1 | 8061 | -1.231515 | 2.8342023 | 2.11E-01 | 3.97E-01 |
| ITGA7 | 3679 | -1.230932 | 1.9132801 | 8.27E-02 | 2.09E-01 |
| NFKBIA | 4792 | -1.229501 | 6.7922251 | 6.18E-12 | 4.01E-10 |
| MT-ND6 | 4541 | -1.228908 | 8.997485 | 2.25E-02 | 8.28E-02 |
| FAAP20 | 199990 | -1.227852 | 5.7316187 | 1.87E-09 | 8.02E-08 |
| DACH1 | 1602 | -1.227807 | 4.8488304 | 1.24E-03 | 8.80E-03 |
| LPCAT2 | 54947 | -1.227354 | 3.4501979 | 8.60E-03 | 4.05E-02 |
| SCUBE1 | 80274 | -1.227317 | 4.8263672 | 4.16E-02 | 1.29E-01 |
| TST | 7263 | -1.224245 | 2.2703785 | 5.38E-03 | 2.85E-02 |
| NDUFA3 | 4696 | -1.222364 | 7.0352728 | 3.01E-06 | 5.58E-05 |
| TRMT12 | 55039 | -1.222229 | 2.9904 | 9.23E-03 | 4.25E-02 |
| CCS | 9973 | -1.22066 | 4.6858589 | 5.17E-04 | 4.34E-03 |
| GABRR1 | 2569 | -1.219344 | 0.8306079 | 2.85E-01 | 4.83E-01 |
| RRP7A | 27341 | -1.219326 | 5.3207948 | 1.39E-11 | 8.11E-10 |
| GIPR | 2696 | -1.218976 | 0.8831619 | 1.12E-01 | 2.59E-01 |
| NLRC3 | 197358 | -1.21865 | 0.4582817 | 3.39E-02 | 1.12E-01 |
| CFL1 | 1072 | -1.217787 | 11.246936 | 1.34E-06 | 2.78E-05 |
| ZNF385A | 25946 | -1.217225 | 3.4976522 | 3.47E-06 | 6.28E-05 |
| XRCC2 | 7516 | -1.21715 | 0.3858605 | 9.13E-02 | 2.24E-01 |
| BACE2 | 25825 | -1.215481 | 2.6160208 | 2.11E-01 | 3.97E-01 |
| CP | 1356 | -1.215183 | 1.6676036 | 2.94E-01 | 4.93E-01 |
| ANGPT1 | 284 | -1.214674 | 1.5459739 | 7.11E-02 | 1.89E-01 |
| LYPD6 | 130574 | -1.211958 | 1.3651719 | 6.68E-03 | 3.34E-02 |
| TLCD2 | 727910 | -1.211688 | 0.4301791 | 8.08E-02 | 2.06E-01 |
| OCEL1 | 79629 | -1.211405 | 4.3558132 | 4.81E-10 | 2.27E-08 |
| C2orf88 | 84281 | -1.208105 | 1.1185602 | 1.90E-01 | 3.71E-01 |
| TPRN | 286262 | -1.207252 | 1.233634 | 2.09E-03 | 1.33E-02 |
| FBLN5 | 10516 | -1.206567 | 6.4462905 | 1.67E-02 | 6.60E-02 |
| KLHL13 | 90293 | -1.206114 | 6.4554302 | 3.91E-03 | 2.19E-02 |
| ADCK5 | 203054 | -1.205861 | 2.6150527 | 1.92E-04 | 1.92E-03 |
| MRPL41 | 64975 | -1.205837 | 7.190918 | 1.75E-05 | 2.58E-04 |
| EPB41L4B | 54566 | -1.201686 | 3.1026116 | 1.08E-04 | 1.20E-03 |
| SHANK2 | 22941 | -1.201411 | 3.4787662 | 7.40E-02 | 1.94E-01 |
| DGKH | 160851 | -1.201359 | 2.1776953 | 5.16E-03 | 2.76E-02 |
| NT5M | 56953 | -1.20129 | 4.2543803 | 4.60E-02 | 1.39E-01 |
| PLOD2 | 5352 | -1.201092 | 4.6147639 | 5.03E-02 | 1.47E-01 |
| IRX1 | 79192 | -1.201069 | 0.1318612 | 2.60E-01 | 4.55E-01 |
| MMP10 | 4319 | -1.20082 | 3.0960911 | 2.64E-02 | 9.28E-02 |
| GGT7 | 2686 | -1.200208 | 3.7265573 | 8.69E-06 | 1.42E-04 |
| CSAD | 51380 | -1.199884 | 2.5455639 | 1.47E-02 | 6.00E-02 |
| PPIC | 5480 | -1.19863 | 3.9613906 | 7.46E-03 | 3.63E-02 |
| MAN2B1 | 4125 | -1.198403 | 2.7610802 | 1.82E-02 | 7.06E-02 |
| ZFPM1 | 161882 | -1.198036 | 1.4517626 | 1.13E-02 | 4.95E-02 |
| SDCCAG8 | 10806 | -1.197584 | 5.0057386 | 1.28E-09 | 5.63E-08 |
| ATG2A | 23130 | -1.196868 | 1.6453411 | 5.76E-02 | 1.63E-01 |
| LRRC2 | 79442 | -1.194462 | 2.8901413 | 3.33E-01 | 5.34E-01 |
| AMOTL1 | 154810 | -1.194337 | 4.3074823 | 4.55E-03 | 2.47E-02 |
| RNASET2 | 8635 | -1.193935 | 3.7115922 | 1.49E-06 | 3.04E-05 |
| ZNF844 | 284391 | -1.19378 | 0.6186413 | 4.38E-02 | 1.34E-01 |
| LARGE1 | 9215 | -1.1937 | 3.4862467 | 1.95E-04 | 1.95E-03 |
| TNPO2 | 30000 | -1.192731 | 4.1106835 | 4.42E-05 | 5.63E-04 |
| PALD1 | 27143 | -1.191994 | 1.249931 | 1.81E-02 | 7.02E-02 |
| MCF2 | 4168 | -1.191307 | 2.7316335 | 1.75E-02 | 6.85E-02 |
| SAR1A | 56681 | -1.190733 | 6.3087423 | 9.46E-07 | 2.07E-05 |
| NRIP1 | 8204 | -1.190529 | 6.0478658 | 7.52E-05 | 8.92E-04 |
| TIMM29 | 90580 | -1.190102 | 4.4038804 | 4.38E-09 | 1.72E-07 |
| COX8A | 1351 | -1.189621 | 7.895933 | 1.48E-04 | 1.55E-03 |
| PSMB10 | 5699 | -1.189554 | 1.1745128 | 2.26E-02 | 8.30E-02 |
| AMMECR1 | 9949 | -1.188538 | 3.0147917 | 5.09E-02 | 1.48E-01 |
| COL4A2 | 1284 | -1.187635 | 6.3015368 | 3.30E-02 | 1.09E-01 |
| CALY | 50632 | -1.187239 | 8.5568405 | 1.05E-01 | 2.48E-01 |
| ADAMTS7 | 11173 | -1.187004 | 2.0175098 | 4.07E-03 | 2.26E-02 |
| RXRA | 6256 | -1.186842 | 2.4523096 | 1.01E-04 | 1.13E-03 |
| C1orf232 | 110806296 | -1.18672 | 0.3260386 | 1.97E-01 | 3.80E-01 |
| PKP4 | 8502 | -1.186405 | 3.8228555 | 2.18E-05 | 3.11E-04 |
| PFAS | 5198 | -1.186029 | 2.2243203 | 1.70E-03 | 1.13E-02 |
| GSTM2 | 2946 | -1.185776 | 5.0437517 | 3.66E-09 | 1.46E-07 |
| FNDC5 | 252995 | -1.185615 | 3.3795766 | 3.97E-02 | 1.25E-01 |
| ARPC1B | 10095 | -1.185216 | -0.025274 | 7.36E-02 | 1.93E-01 |
| PAMR1 | 25891 | -1.185025 | -0.119043 | 1.27E-01 | 2.83E-01 |
| GABBR2 | 9568 | -1.183708 | 6.2590551 | 9.94E-08 | 2.82E-06 |
| OBSCN | 84033 | -1.182822 | 1.756359 | 1.40E-03 | 9.67E-03 |
| TMEM87B | 84910 | -1.180857 | 1.6518865 | 1.76E-02 | 6.88E-02 |
| ABCA2 | 20 | -1.180679 | 2.3989424 | 5.56E-03 | 2.92E-02 |
| PAQR7 | 164091 | -1.180649 | -0.063979 | 1.01E-01 | 2.40E-01 |
| IRAK1BP1 | 134728 | -1.18015 | 3.181118 | 6.50E-05 | 7.90E-04 |
| IL4R | 3566 | -1.179737 | 0.9920686 | 3.57E-01 | 5.56E-01 |
| BAZ1A | 11177 | -1.178848 | 3.1560959 | 7.41E-04 | 5.83E-03 |
| NDE1 | 54820 | -1.177987 | 0.8577563 | 1.18E-01 | 2.69E-01 |
| TAF10 | 6881 | -1.176043 | 2.9610914 | 2.86E-05 | 3.91E-04 |
| ZBTB47 | 92999 | -1.17518 | 1.1086445 | 5.70E-03 | 2.98E-02 |
| SYDE2 | 84144 | -1.175058 | 1.3012074 | 3.12E-02 | 1.05E-01 |
| S1PR3 | 286223 | -1.174241 | 3.5308426 | 5.02E-02 | 1.47E-01 |
| OXR1 | 55074 | -1.174157 | 7.6282171 | 1.93E-03 | 1.25E-02 |
| FAM168A | 23201 | -1.173094 | 5.0829783 | 6.02E-03 | 3.11E-02 |
| LUM | 4060 | -1.172612 | 5.3696014 | 6.77E-06 | 1.14E-04 |
| B2M | 567 | -1.172418 | 8.2223332 | 6.00E-07 | 1.36E-05 |
| ZFP3 | 124961 | -1.171251 | 1.9129378 | 4.31E-02 | 1.33E-01 |
| MRPS12 | 6183 | -1.170868 | 6.3015163 | 6.00E-09 | 2.29E-07 |
| GRM5 | 2915 | -1.169842 | 2.7084534 | 1.19E-01 | 2.70E-01 |
| ZNF224 | 7767 | -1.168968 | 2.8811605 | 8.41E-04 | 6.42E-03 |
| EIF1AX | 1964 | -1.168597 | 7.3696242 | 4.99E-06 | 8.66E-05 |
| LRRC43 | 254050 | -1.16808 | 0.9887098 | 9.52E-02 | 2.31E-01 |
| KCNA2 | 3737 | -1.168052 | 1.9383201 | 4.50E-03 | 2.45E-02 |
| SLC66A3 | 130814 | -1.16683 | 3.4250736 | 4.67E-02 | 1.40E-01 |
| OR2W3 | 343171 | -1.165897 | 0.0584384 | 2.09E-01 | 3.95E-01 |
| LRRC45 | 201255 | -1.165824 | 2.0211208 | 6.06E-04 | 4.93E-03 |
| RTTN | 25914 | -1.165632 | 1.6464122 | 2.11E-02 | 7.87E-02 |
| ACTB | 60 | -1.163867 | 14.628782 | 2.23E-07 | 5.74E-06 |
| TNFRSF11B | 4982 | -1.162905 | 4.3711534 | 1.92E-01 | 3.74E-01 |
| PBX2 | 5089 | -1.160085 | -0.137783 | 4.08E-02 | 1.28E-01 |
| NPTX2 | 4885 | -1.160039 | 5.3236867 | 7.52E-04 | 5.90E-03 |
| GRIK3 | 2899 | -1.159324 | 2.4299811 | 3.15E-01 | 5.15E-01 |
| CCDC142 | 84865 | -1.158726 | 2.7260297 | 1.04E-03 | 7.63E-03 |
| ZFR2 | 23217 | -1.15871 | 5.837183 | 5.04E-02 | 1.47E-01 |
| NEUROD1 | 4760 | -1.157742 | 1.6864276 | 6.19E-02 | 1.71E-01 |
| ZEB2 | 9839 | -1.157741 | 3.176031 | 8.06E-04 | 6.23E-03 |
| BBS1 | 582 | -1.155726 | 0.2659691 | 1.14E-02 | 4.97E-02 |
| FUT9 | 10690 | -1.15551 | 4.5806211 | 2.38E-02 | 8.61E-02 |
| PSME4 | 23198 | -1.155227 | 3.707282 | 3.46E-07 | 8.42E-06 |
| TCP11L2 | 255394 | -1.155193 | 1.7163657 | 1.91E-02 | 7.33E-02 |
| PID1 | 55022 | -1.154867 | 5.2678534 | 3.79E-02 | 1.21E-01 |
| TGFBR3L | 100507588 | -1.154508 | 1.8440713 | 6.77E-03 | 3.38E-02 |
| MGST3 | 4259 | -1.154402 | 9.5238431 | 4.34E-04 | 3.77E-03 |
| SHOX2 | 6474 | -1.152844 | 1.2336267 | 2.18E-01 | 4.06E-01 |
| CHODL | 140578 | -1.152731 | 5.1346761 | 9.29E-03 | 4.27E-02 |
| TAB1 | 10454 | -1.152559 | 1.6860798 | 2.15E-03 | 1.36E-02 |
| NEK5 | 341676 | -1.151674 | 0.3535097 | 9.07E-02 | 2.23E-01 |
| PDE5A | 8654 | -1.151293 | 1.9661169 | 8.03E-03 | 3.84E-02 |
| SAMD1 | 90378 | -1.150047 | 4.9269354 | 6.69E-03 | 3.35E-02 |
| ADGRL2 | 23266 | -1.149628 | 3.8233506 | 3.10E-04 | 2.86E-03 |
| NPY1R | 4886 | -1.148713 | 4.49754 | 1.54E-01 | 3.21E-01 |
| KCND2 | 3751 | -1.147564 | 4.0142418 | 3.52E-01 | 5.52E-01 |
| TLE2 | 7089 | -1.14704 | 4.5242357 | 6.58E-02 | 1.78E-01 |
| SCAMP5 | 192683 | -1.14639 | 4.7717942 | 1.01E-02 | 4.55E-02 |
| EEF2K | 29904 | -1.146133 | 2.5672478 | 9.76E-04 | 7.26E-03 |
| TMTC1 | 83857 | -1.14578 | 4.1608165 | 1.63E-05 | 2.43E-04 |
| CRLS1 | 54675 | -1.145376 | 2.7806158 | 5.78E-05 | 7.18E-04 |
| ECRG4 | 84417 | -1.145271 | 0.5393586 | 2.55E-01 | 4.49E-01 |
| SOX11 | 6664 | -1.145097 | 7.0336219 | 7.00E-02 | 1.86E-01 |
| RESP18 | 389075 | -1.143105 | 0.7010363 | 2.46E-01 | 4.39E-01 |
| ROPN1B | 152015 | -1.142192 | 1.6854108 | 1.61E-01 | 3.30E-01 |
| NPM2 | 10361 | -1.141653 | 3.9734609 | 1.32E-01 | 2.90E-01 |
| PPIB | 5479 | -1.140729 | 7.2346552 | 4.35E-07 | 1.02E-05 |
[truncated: 239,432 more chars]
