# Supplementary material for: Carvedilol suppresses ryanodine receptor-dependent Ca2+ bursts in human neurons bearing PSEN1 variants found in early onset Alzheimer’s disease
Source: PLoS One. 2024 Aug 22;19(8):e0291887. doi: 10.1371/journal.pone.0291887 (PMC11341060; doi:10.1371/journal.pone.0291887)
Supplement: Attachment — Submitted filename: Hori_PLoS_R1_Answers_V9.0_03272024.docx [file pone.0291887.s006.docx]

**Responses to the comments**

PONE-D-23-23711

Carvedilol suppresses ryanodine receptor-dependent Ca2+ bursts in human neurons bearing PSEN1 variants found in early onset Alzheimer’s disease

PLOS ONE

Dear Dr. Ai,

Thank you for submitting your manuscript to PLOS ONE. After careful consideration, we feel that it has merit but does not fully meet PLOS ONE’s publication criteria as it currently stands. Therefore, we invite you to submit a revised version of the manuscript that addresses the points raised during the review process.

If you would like to make changes to your financial disclosure, please include your updated statement in your cover letter. Guidelines for resubmitting your figure files are available below the reviewer comments at the end of this letter.

If applicable, we recommend that you deposit your laboratory protocols in [protocols.io](http://protocols.io/) to enhance the reproducibility of your results. Protocols.io assigns your protocol its own identifier (DOI) so that it can be cited independently in the future. For instructions see: <https://journals.plos.org/plosone/s/submission-guidelines#loc-laboratory-protocols>. Additionally, PLOS ONE offers an option for publishing peer-reviewed Lab Protocol articles, which describe protocols hosted on [protocols.io](http://protocols.io/). Read more information on sharing protocols at <https://plos.org/protocols?utm_medium=editorial-email&utm_source=authorletters&utm_campaign=protocols>.

We look forward to receiving your revised manuscript.

Kind regards,

Pan Li, PhD
Academic Editor
PLOS ONE

**Journal Requirements:**

When submitting your revision, we need you to address these additional requirements.

1. Please ensure that your manuscript meets PLOS ONE's style requirements, including those for file naming. The PLOS ONE style templates can be found at 
[https://journals.plos.org/plosone/s/file?id=wjVg/PLOSOne_formatting_sample_main _body.pdf](https://journals.plos.org/plosone/s/file?id=wjVg/PLOSOne_formatting_sample_main%20%20%20%20_body.pdf) and 
<https://journals.plos.org/plosone/s/file?id=ba62/PLOSOne_formatting_sample_title_authors_affiliations.pdf>

2.  Note from Emily Chenette, Editor in Chief of PLOS ONE, and Iain Hrynaszkiewicz, Director of Open Research Solutions at PLOS: Did you know that depositing data in a repository is associated with up to a 25% citation advantage (<https://doi.org/10.1371/journal.pone.0230416>)? If you’ve not already done so, consider depositing your raw data in a repository to ensure your work is read, appreciated and cited by the largest possible audience. You’ll also earn an Accessible Data icon on your published paper if you deposit your data in any participating repository (<https://plos.org/open-science/open-data/#accessible-data>).

3. Thank you for stating the following financial disclosure: 
"Japan Society for the Promotion of Science (JSPS) grant 20K16532 (AH)
JSPS grant 18K08900 and 21K08135 (TA)"

Please state what role the funders took in the study.  If the funders had no role, please state: "The funders had no role in study design, data collection and analysis, decision to publish, or preparation of the manuscript." 
If this statement is not correct you must amend it as needed. 
Please include this amended Role of Funder statement in your cover letter; we will change the online submission form on your behalf.

**Response**: We would like to amend the statement as follows: "This study was supported by the Japan Society for the Promotion of Science (JSPS) grant 20K16532 (AH) and JSPS grant 18K08900 and partially supported by the JSPS grant 21K08135 (TA). All materials such as cells and reagents were purchased using these grants".

4. We note that you have stated that you will provide repository information for your data at acceptance. Should your manuscript be accepted for publication, we will hold it until you provide the relevant accession numbers or DOIs necessary to access your data. If you wish to make changes to your Data Availability statement, please describe these changes in your cover letter and we will update your Data Availability statement to reflect the information you provide.

**Response**: We apologize for providing an incorrect statement. We would like to state that all data information is included in the manuscript. Both reviewers have stated that all necessary data are available in the manuscript.

[Note: HTML markup is below. Please do not edit.]

Reviewers' comments:

Reviewer's Responses to Questions

**Comments to the Author**

1. Is the manuscript technically sound, and do the data support the conclusions?

The manuscript must describe a technically sound piece of scientific research with data that supports the conclusions. Experiments must have been conducted rigorously, with appropriate controls, replication, and sample sizes. The conclusions must be drawn appropriately based on the data presented.

Reviewer #1: Partly

Reviewer #2: Partly

2. Has the statistical analysis been performed appropriately and rigorously?

Reviewer #1: Yes

Reviewer #2: I Don't Know

3. Have the authors made all data underlying the findings in their manuscript fully available?

The [PLOS Data policy](http://www.plosone.org/static/policies.action#sharing) requires authors to make all data underlying the findings described in their manuscript fully available without restriction, with rare exception (please refer to the Data Availability Statement in the manuscript PDF file). The data should be provided as part of the manuscript or its supporting information, or deposited to a public repository. For example, in addition to summary statistics, the data points behind means, medians and variance measures should be available. If there are restrictions on publicly sharing data—e.g. participant privacy or use of data from a third party—those must be specified.

Reviewer #1: Yes

Reviewer #2: Yes

4. Is the manuscript presented in an intelligible fashion and written in standard English?

PLOS ONE does not copyedit accepted manuscripts, so the language in submitted articles must be clear, correct, and unambiguous. Any typographical or grammatical errors should be corrected at revision, so please note any specific errors here.

Reviewer #1: Yes

Reviewer #2: No

**5. Review Comments to the Author**

Please use the space provided to explain your answers to the questions above. You may also include additional comments for the author, including concerns about dual publication, research ethics, or publication ethics. (Please upload your review as an attachment if it exceeds 20,000 characters)

**Reviewer #1:** The manuscript of Hori et al. entitled:”Carvedilol suppresses ryanodine receptor-dependent Ca2+ bursts in human neurons bearing PSEN1 variants found in early onset Alzheimer’s disease” attempts to support the importance of amyloid β (Aβ)-independent mechanisms in the pathogenesis of early-onset Alzheimer’s disease (AD). The authors focused on a potential role of Ca2+ dysregulation in neuronal cells derived from early-onset AD patients harboring three variants of Presenilin-1 (A246E, L286V, and M146L). I agree that much attention has been given to the Aβ hypothesis of AD, however, an increasing body of evidence suggests that dysregulation of neuronal Ca2+ homeostasis may play a fundamental role in the pathogenesis of AD, at least in a sporadic form. Thus, it is feasible to investigate whether neuronal Ca2+ dysregulation might be implicated also in early-onset AD. Reading the manuscript, I found some major and minor issues that need to be addressed by the authors.

Response 1: We appreciate your time and efforts to review our manuscript and providing us with constructive feedback.

MAJOR issues
1. Fig. 4: I was quite surprised that dantrolene did not have any effect on Ca2+ oscillations. For a long time, dantrolene has been consider a selective inhibitor of the RyR1 and RyR3 channels. However, it seems that dantrolene is able to inhibit also the RyR2 channel. This interaction is very likely conditioned by phosphorylation of the channel and/or the presence of associated protein, FKBP12 (DOI: 10.1016/j.jjcc.2019.08.020; 10.1016/s1388-9842(99)00017-3; 10.1016/j.hroo.2020.03.004; 10.1371/journal.pone.0125366; 10.1085/jgp.202213277). And indeed, increased phosphorylation of the RyR2 channel in the AD brains has been reported (DOI:10.1007/s00401-017-1733-7). Thus, I would expect a strong effect of dantrolene. This issue has to be addressed by the authors and mentioned works should be cited.

**Response**: We are aware that the effects of dantrolene on the RyR2 channels are controversial. However, the aim of this study is not to investigate the inconsistent results and opinions among the researchers in this field.

The proposal by Kobayashi et al. is a clinical trial to examine whether dantrolene can suppress cardiac arrhythmias [PMID: 31866190]. In another study by Meissner, in human papillary muscles resected from failing hearts dantrolene improved the myocardium-responsiveness to the β-stimulation which did not show direct effects on RyR2 [PMID: 10937928]. Nofi et al. reported that dantrolene attenuated cardiac dysfunction and reduced atrial fibrillation inducibility in a post-myocardial infarction heart failure rat model [PMID: 34113867]. Weel et al. claimed the role of FKBP12.6 in inhibition of RyR2 by dantrolene [PMID: 37279522]. This experiment was done using artificial lipid bilayers, which is a different environment from actual human cells. In addition, the association of FKBP12.6 and RyR2 is controversial [PMID: 17921453]. Furthermore, the role of phosphorylation of RyR2 in heart failure is also controversial [PMID: 24723657; PMID: 22302785].

In contrast, many studies showed that dantrolene does not affect the RyR2 or cardiac functions though these experiments were also performed using animal models or cell lines [e.g., PMID: 966155; PMID: 1015907; PMID: 29674523].

Our data showed the fact that dantrolene did not affect the Ca^2+^-bursts in human iPSC-induced neurons bearing *PSEN1* variants. At this moment, we do not know what the underlying mechanism is. However, even if dantrolene can suppress the Ca^2+^-bursts by some interventions in our systems, it will not affect our conclusion that carvedilol suppresses the Ca^2+^-bursts. We added descriptions regarding these issues in the discussion section (Page 20).

2. Fig.5: To assess whether protein amounts for the RyR2 channel and PSEN1 were changed in PSEN1-A246E cells comparison to WT control is more appropriate to use standard Western blotting, especially in the case of the RyR2 channel. This is because the anti-RyR antibody used also labels a 130 kDa band, which is not the RyR channel. This non-RyR binding interferes with the RyR-related signal in confocal images. Furthermore, I have doubts about the RyR2-specificity of the antibody, as I did not find satisfactory information on the supplier's website.

**Response**: Fig. 5 was replaced by the Western blot data. We used a different antibody made by our coauthors [PMID: 12805094] in the new experiments. The description was rewritten (Pages 15 and 16).

3. In connection to Fig.5: The amount of the RyR2 channel in PSEN1-A246E cells versus WT control was not changed when analyzed from confocal images, however, RNA-Seq showed downregulation of RYR2 and RYR3 gens in PSEN1-A246E cells. How could be this finding interpreted?

**Response**: Persistently high intracellular Ca^2+^ may downregulate the expression of *RYR2* and *RYR3* although we do not know the exact mechanisms. Additionally, gene expressions do not always co-relate with protein expressions. We added a description in Discussion (Page 19).

4. The authors mentioned in the Introduction and Discussion sections that PSEN1 is a Ca2+ pore. However, they did not cite the most relevant papers, which directly showed conductive properties of PSEN1 using the BLM method (DOI: 10.1074/jbc.M111.243063; 10.1016/j.Cell.2006.06). These works should be cited.

**Response**: The manuscript [PMID: 21531718; DOI: 10.1074/jbc.M111.243063] was cited in the introduction. I was wondering what the other one would be. Did the reviewer mean PMID: 16923388 (DOI: 10.1016/j.cell.2006.06.044)?

MINOR issues
1. Fig. 2: I recommend to show also representative images of Cal-520 filled cells.

**Response**: The cell images filled with Cal-520 were added in the figure (New Fig 1).

2. Fig. 6: The resolution of this figure has to be enhanced because labelling is not legible in the submitted version.

**Response**: We revised Fig 6, and Fig 6B was removed based on the comments by Reviewer 2 (Page 16).

**Reviewer #2:** This work evaluated the role of ryanodine and IP3 receptors in the generation of calcium signals generated in cell lines derived from patients with familial forms of Alzheimer's disease with a mutation in presenilin 1. Although the results are of interest and can contribute to the discussion involving these receptors in Alzheimer's disease, several issues should be addressed before publication:

**Response**: We appreciate your time and efforts to review our manuscript and providing us with your valuable comments to improve our manuscript.

1) For example, the authors mention that" human neurons express RyR1 and RyR2" and ignore the RyR3 isoform, which is a brain isoform. They use inhibitors for RyR1 and RyR2 and conclude that RyR2 is very important, but they did not discuss the possible implications of RyR3. Moreover, in the hippocampal neurons, a brain region centrally involved in AD, low levels of RyR1 are present, while RyR3 is highly expressed.

**Response**: As the reviewer pointed out, RyR3 is expressed in human brains [https://www.ncbi.nlm.nih.gov/gene/6263]. However, dantrolene inhibits RyR3 as well as RyR1 [PMID: 11278295; PMID: 36982484]. Though this does not change our conclusion, the descriptions were added (Pages 4 and 19).

2) They also mentioned a study using dantrolene, an inhibitor of RyR1, as capable of eliminating both Aß42 and Ca2+ signals. However, they dismiss these findings, mentioning that these data still support the classical amyloid theory, which may not be the primary pathological mechanism. It is important to note that although a discussion has been made on the centrality of amyloid to AD pathogenesis, the amyloid cascade is still the leading hypothesis to explain the disease.

**Response**: The sentence, “These data still support the classical amyloid theory”, was removed.

3) Still on this matter, the authors omit a critical paper (<https://doi.org/10.3389/fnmol.2017.00115>) showing that Ca2+ release mediated by the RyR2 isoform causes the deleterious effects of AβOs on mitochondrial function in hippocampal neurons. Knockdown of RyR2 decreased by 40% Ca2+ release induced by the RyR agonist 4-chloro-m-cresol and significantly reduced the cytoplasmic and mitochondrial Ca2+ signals and the mitochondrial fragmentation induced by AβOs. Also, in another work from the same group (10.3389/fnagi.2018.00399), it was shown that AβOs injections directly into the hippocampus decreased RyR2 protein content but increased single RyR2 channel activation by Ca2+ and caused considerable spatial memory deficits. These articles should be cited and discussed correctly, together with Yao'paaper, in the manuscript.

**Response**: The aim of this study is to investigate whether *PSEN1* variants found in early-onset AD can promote abnormal Ca^2+^-signals in human iPSC-induced neuronal cells. In other types of AD, AβOs can increase intracellular Ca^2+^ leading to neuronal damage. We modified the discussion section (Page 18-19).

3) Figure 1 is a characterization of the ability to generate neurons from cell lines with previously described protocols, so it should be considered a supplementary figure. The figure legends do not specify the rum or the statistical test used.

**Response**: The initial Fig 1 was changed as Fig S1. These are representative neuronal images, and statistical methods were not used.

4) Regarding Figure 2, the authors mention that Ca2+-bursts were significantly higher in neurons bearing PSEN1 variants (p.A246E and p.M146L) compared to the WT neurons, suggesting that these two variants may cause more severe phenotypes than p.L286V. Although they show a quantification, no statistical analysis was presented to conclude that Ca2+ bursts were higher in those cases than the others.

**Response**: Statistical analysis was performed and shown as follows:

Comparisons of the rates of Ca^2+^-oscillations and Ca^2+^-bursts in neurons bearing PSEN1 variants were performed between p.A246E and the WT neurons, between p.M146L and the WT neurons, and between p.L286V and the WT neurons. Regarding Ca^2+^-oscillations, the 99% confidence intervals (CI) of these comparisons were all significant. (Table S1) Comparisons of the rates of Ca^2+^-bursts were [99%CI: -0.483, -0.338] (p.A246E and WT) and [99%CI: -0.224, -0.020] (p.M146L and WT), respectively, both of which were significant (p<0.01). Meanwhile, a comparison of the rates of Ca^2+^-bursts in neurons with PSEN1 variants between p.L286V and the WT neurons showed [95%CI: -0.100, 0.019] which was nonsignificant. (Table S2) Therefore, we suggested that the two variants with p.A246E and p.M146L may cause more severe phenotypes than p.L286V (Page 12).

Accordingly, the Fig 2 was revised as shown below:

**p<0.01, NS: not significant

**Table S1**

| Ca oscillation | WT | A246E | M146L | L286V |
| --- | --- | --- | --- | --- |
| n | 592 | 353 | 155 | 363 |
| Ratio (%) | 9.1 | 34.6 | 52.9 | 59.5 |

| PSEN1  variants | PSEN1 variants | rates1 | rates 2 | difference | significance | 95%CI  LL | 95%CI  UL | 95%CI  LL | 95%CI  UL |
| --- | --- | --- | --- | --- | --- | --- | --- | --- | --- |
| WT | A246E | 0.091 | 0.346 | 9.717 | ** | -0.314 | -0.195 | -0.329 | -0.180 |
| WT | M146L | 0.091 | 0.529 | 11.452 | ** | -0.532 | -0.344 | -0.553 | -0.323 |
| WT | L286V | 0.091 | 0.595 | 16.149 | ** | -0.584 | -0.424 | -0.601 | -0.407 |
| A246E | M146L | 0.346 | 0.529 | 4.008 | ** | -0.290 | -0.076 | -0.318 | -0.049 |
| A246E | L286V | 0.346 | 0.595 | 6.842 | ** | -0.341 | -0.158 | -0.362 | -0.137 |
| M146L | L286V | 0.529 | 0.595 | 1.393 | NS | -0.173 | 0.041 | -0.201 | 0.069 |

NS: not significant, **p<0.01, CI: confidence interval, LL: lower limit, UL: upper limit

**Table S2**

| Ca burst | WT | A246E | M146L | L286V |
| --- | --- | --- | --- | --- |
| n | 592 | 353 | 155 | 363 |
| Ratio (%) | 2.0 | 43.1 | 14.2 | 6.1 |

| PSEN1  variants | PSEN1 variants | rates1 | rates 2 | difference | significance | 95%CI  LL | 95%CI  UL | 95%CI  LL | 95%CI  UL |
| --- | --- | --- | --- | --- | --- | --- | --- | --- | --- |
| WT | A246E | 0.020 | 0.431 | -0.410 | ** | -0.468 | -0.353 | -0.483 | -0.338 |
| WT | M146L | 0.020 | 0.142 | -0.122 | ** | -0.205 | -0.039 | -0.224 | -0.020 |
| WT | L286V | 0.020 | 0.061 | -0.040 | NS | -0.100 | 0.019 | -0.113 | 0.032 |
| A246E | M146L | 0.431 | 0.142 | 0.289 | ** | 0.185 | 0.392 | 0.159 | 0.419 |
| A246E | L286V | 0.431 | 0.061 | 0.370 | ** | 0.293 | 0.447 | 0.276 | 0.464 |
| M146L | L286V | 0.142 | 0.061 | 0.081 | ** | 0.021 | 0.142 | 0.005 | 0.157 |

NS: not significant, **p<0.01, CI: confidence interval, LL: lower limit, UL: upper limit

Supplementary tables, Table S1 and S2, were added to the supplementary materials as shown above.

5) In Figure 3, the results are coherent, but what were the controls to check that BAPTA and CPA did not kill the cells after their appliance to the cultures?

**Response**: In the revised **Fig 3**, we added the traces obtained from the same cells after washout of BAPTA and CPA. As you can see, those cells showed Ca^2+^-bursts after washout (Page 13-14).

6) In the figure 4, they used carvedilol to block RyR2. Using genetic tools to imply RyR2 in the Ca2+ signals would be relevant. Also, although n is shown for each condition in the figure legend, statistical analysis needs to be mentioned, which is needed to make conclusions about the role of RyR2 as the "main facilitator of the abnormal Ca2+-bursts in the cells bearing PSEN1 variant.". Moreover, significant differences exist among the N used in the different conditions. They mention N = 21 in B, N = 98i C, and N = 18 in D. Given this disparity and the elevated values for N, it is necessary to describe what the authors consider an N.

**Response**: We randomly chose neuronal cells showing Ca^2+^-bursts, and the effects of the reagents were consistent in all cells (i.e., all-or-none). We did not intentionally emphasize the role of RyR2 based on these numbers. In general, approximately 20 consistent observations are thought to be a true phenomenon (i.e., not experimental error). Please see some of our previous papers for examples [PMID: 12208804; PMID: 15767295]. Descriptions were added on Page 14.

7) The authors should describe how they performed quantifying the immunofluorescence images in Figure 5.

**Response**: Based on the other reviewer, this data was replaced with a Western blot data. Please see the revised methods and results section (Page 15).

8) RyR3 was omitted until Figure 6, where it appears to be decreased just as RyR2, but without any comment. Also, the scheme shown in Figure 6B is speculative and can not be supported by the results presented in this work. Moreover, the resolution is inacceptable in this figure.

**Response**: Fig 6B was removed based on the reviewer’s comment and Fig 6A was revised (New Fig 6).
